# Emission solvatochromic, solid-state and aggregation-induced emissive α-pyrones and emission-tuneable 1*H*-pyridines by Michael addition–cyclocondensation sequences

**DOI:** 10.3762/bjoc.15.262

**Published:** 2019-11-12

**Authors:** Natascha Breuer, Irina Gruber, Christoph Janiak, Thomas J J Müller

**Affiliations:** 1 Heinrich-Heine Universität Düsseldorf, Institut für Organische Chemie und Makromolekulare Chemie, Universitätsstraße 1, D-40225 Düsseldorf, Germany; 2 Heinrich-Heine Universität Düsseldorf, Institut für Anorganische Chemie und Strukturchemie, Universitätsstraße 1, D-40225 Düsseldorf, Germany

**Keywords:** cyclocondensation, DFT calculations, fluorescence, heterocycles, 1*H*-pyridines, α-pyrones

## Abstract

Starting from substituted alkynones, α-pyrones and/or 1*H*-pyridines were generated in a Michael addition–cyclocondensation with ethyl cyanoacetate. The peculiar product formation depends on the reaction conditions as well as on the electronic substitution pattern of the alkynone. While electron-donating groups furnish α-pyrones as main products, electron-withdrawing groups predominantly give the corresponding 1*H*-pyridines. Both heterocycle classes fluoresce in solution and in the solid state. In particular, dimethylamino-substituted α-pyrones, as donor–acceptor systems, display remarkable photophysical properties, such as strongly red-shifted absorption and emission maxima with daylight fluorescence and fluorescence quantum yields up to 99% in solution and around 11% in the solid state, as well as pronounced emission solvatochromism. Also a donor-substituted α-pyrone shows pronounced aggregation-induced emission enhancement.

## Introduction

A high sensitivity and precise tuneability of fluorescence colors are prerequisites for the application of fluorescent substances in chemistry, medicine and materials science [1]. With this respect emissive small molecules [2], fluorescent proteins [3], and quantum dots have received considerable attention and remarkable progress in their synthesis and photophysics has been achieved [4]. Small molecule organic fluorophores are particularly advantageous due to the potential of a tailored fine-tuning of their photophysical properties through synthetic modifications [5]. Based on their structural features, functionalized organic chromophores, containing N-, O- or S-atoms, are increasingly used in OLEDs [6–10] and LCDs [11–13] of mobile phones [14]. Fluorescent compounds often intensively emit in solution but only weakly or not in the solid state [15]. Dyes which fluoresce both in the solid state and in solution are still relatively rare, due to the fact that often molecular aggregation in the solid state causes fluorescence quenching [16].

In recent years, we have coined diversity-oriented syntheses of functional chromophores by multicomponent strategies [17–18], opening accesses to substance libraries for systematic studies of structure–property relationships on fluorophores [19], in particular on aggregation-induced emissive polar dyes [20]. Conceptually, many of these consecutive multicomponent syntheses rely on transition-metal-catalyzed heterocyclic syntheses [21]. By virtue of catalytic generation of alkynones [22] we have recently disclosed consecutive alkynylation–Michael addition–cyclocondensation (AMAC) multicomponent syntheses of α-pyrones [23].

While most α-pyrones neither fluoresce in solution nor in the solid state specific substitution patterns have been identified for fluorophore design for this heterocyclic family. Tominaga and co-workers synthesized a series of α-pyrone derivatives with emission maxima between 400 and 675 nm in the solid state and between 486 and 542 nm in chloroform [16,24–26], including fluorescence quantum yields as high as 95% in solution and 58% in the solid state [16,24]. While these fluorophores were synthesized by cyclocondensation with ketene dithioacetals and substituted acetophenones other cyano-containing derivatives became accessible by desymmetrizing cyclocondensation of 1,2-diaroylacetylenes with ethyl cyanoacetate [27], similar to related studies with dialkyl malonates [28]. Here, we report on effects of base and temperature on Michael addition–cyclocondensation sequences in the formation of α-pyrones and/or 1*H*-pyridines starting from diversely substituted alkynones and cyanoethylacetate. This bifurcating domino process furnishes small chromophore libraries which were characterized by photophysical studies (absorption and emission spectroscopy) and the studies on the electronic structure were accompanied by TD-DFT calculations for assigning the dominant longest-wavelength absorption bands.

## Results and Discussion

### Synthesis and tentative mechanism

**Scheme 1:**
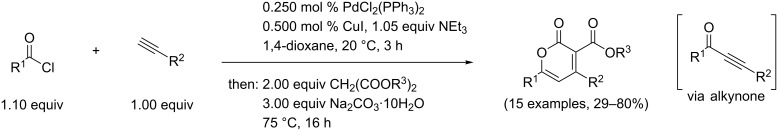
Consecutive three-component alkynylation–Michael addition–cyclocondensation (AMAC) synthesis of α-pyrones from acid chlorides, terminal alkynes and dialkyl malonates.

Recently, we reported a straightforward access to α-pyrones through a consecutive alkynylation–Michael addition–cyclocondensation (AMAC) multicomponent synthesis [23]. The reaction can be rationalized by a Sonogashira coupling between an acid chloride and a terminal alkyne furnishing an alkynone, which is transformed without isolation by addition of dialkyl malonates in a Michael addition–cyclocondensation to form α-pyrones (Scheme 1).

**Scheme 2:**
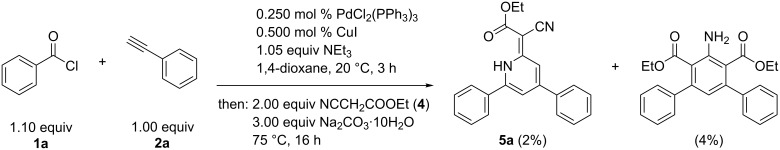
Consecutive pseudo-four-component alkynylation–Michael addition–cyclocondensation (AMAC) synthesis of 1*H*-pyridines **5a** and an aniline derivative.

With this sequence in hand, we envisioned the variation of CH-acidic esters to generate differently 3-substituted α-pyrones. For introducing a cyano substituent we employed benzoyl chloride (**1a**), phenylacetylene (**2a**), and ethyl cyanoacetate (**4**) within the AMAC sequence (Scheme 2). Surprisingly, the desired α-pyrone was not isolated, but two other compounds were detected. On the one hand a 1*H*-pyridine derivative **5a** (2% yield) and on the other hand an aniline derivative with two ester groups (4% yield). Both compounds indicate that two molecules of ethyl cyanoacetate (**4**) were incorporated in the final structure.

**Scheme 3:**
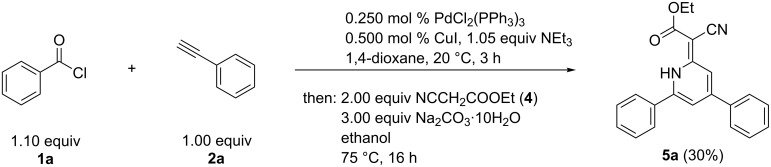
Consecutive pseudo-four-component alkynylation–Michael addition–cyclocondensation (AMAC) synthesis of 1*H*-pyridines **5a** from acid chlorides **1**, terminal alkynes **2** and ethyl cyanoacetate (**4**).

With an increased amount of ethyl cyanoacetate the yield of both products could be increased. By the addition of ethanol as a cosolvent in the second step of the sequence, 1*H*-pyridine **5a** could be isolated in 30% yield, while the aniline derivative was not formed (Scheme 3).

There are only a few known methods for the synthesis of this kind of 1*H*-pyridines. In a cyclocondensation, starting from 1,3-dicarbonyl compounds, Elnagdi and co-workers synthesized 1*H*-pyridines with an additional cyano substituent in the 3-position [29]. Most syntheses generating 1*H*-pyridines make use of ethyl cyanoactate as a starting material. It can react with itself and forms a dimer by selfcondensation, catalyzed by transition metals [30–31].

**Scheme 4:**
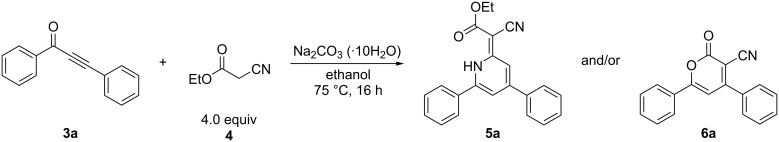
Model system for the optimization of the Michael addition–cyclocondensation reaction step to 1*H*-pyridine **5a** or/and α-pyrone **6a**.

Intrigued by the unusual pseudo-four-component AMAC synthesis we investigated the reaction conditions of the terminal Michael addition–cyclocondensation step starting from alkynone **3a**. By varying the amount of the base we could observe the formation of 1*H*-pyridine **5a**, but also of α-pyrone **6a** (Scheme 4, Table 1), similarly to the reaction of compound **4** with 1,2-diaroylacetylenes [28].

**Table 1:** Optimization of the cyclization step of 1,5-diacyl-5-hydroxypyrazoline **5b**.^a^

Entry	Base (equiv)	Compound **5a** (yield)^b^	Compound **6a** (yield)^b^

1	Na_2_CO_3_∙10H_2_O (0.80)	20%	50%
2	Na_2_CO_3_∙10H_2_O (1.0)	8%	64%
3	Na_2_CO_3_∙10H_2_O (1.5)	32%	–
4	Na_2_CO_3_∙10H_2_O (2.0)	26%	–
5	Na_2_CO_3_ (0.80)	3%	41%
6^c^	Na_2_CO_3_ (0.80)	22%	52%
7^b^	Na_2_CO_3_ (1.4)	9%	66%

^a^All reactions were carried out on a 0.500 mmol scale (*c*_0_(**3a**) = 0.50 M, *c*_0_(**4**) = 2.0 M; ^b^all yields refer to isolated and purified products; ^c^additional water (5.6 equiv).

With either 0.8 or 1.0 equiv of Na_2_CO_3_·10H_2_O α-pyrone **6a** is formed as the main product (50–64%), while 1*H*-pyridine **5a** can also be isolated in around 15% yield (Table 1, entries 1 and 2). By increasing the amount of Na_2_CO_3_·10H_2_O, exclusively 1*H*-pyridine **5a** can be isolated in low yield (Table 1, entries 3 and 4). Using anhydrous sodium carbonate α-pyrone **6a** is again formed as the main product in 41% yield, but the yield of 1*H*-pyridine **5a** drops to 3% (Table 1, entry 5). By the addition of water, the yield of **6a** could be increased (Table 1, entries 6 and 7).

Next we evaluated the use of a mixture of two bases, sodium carbonate and sodium acetate, and water (Table 2). With 0.80 equiv of sodium carbonate, 0.60 equiv of sodium acetate and 5.6 equiv of water 1*H*-pyridine **5a** could be isolated in 56% yield. Decreasing the amount of ethyl cyanoacetate (**4**) the yields drops (Table 2, entry 2), however, increasing the amount of substrate **4** does not improve the yield (Table 2, entry 3). The exclusion of water only causes a decrease in yield (Table 2, entry 4). With sodium acetate as the only base, both 1*H*-pyridine **5a** and α-pyrone **6a** are formed in ca. 25% yield each (Table 2, entry 5). Sodium acetate with additional water gives 1*H*-pyridine **5a** in 23% yield (Table 2, entry 6). It seems to be important that both bases and water are present, but neither a reduction nor an increase of the amount of water increased the yields of 1*H*-pyridine **5a** (Table 2, entries 7–9). The increase of neither sodium carbonate (Table 2, entry 10) nor sodium acetate (Table 2, entry 11) caused an increase in yields.

**Table 2:** Optimization of the formation of 1*H*-pyridine **5a** or/and α-pyrone **6a** in the Michael addition–cyclocondensation reaction with Na_2_CO_3_, NaOAc and water.^a^

Entry	Na_2_CO_3_ [equiv]	NaOAc [equiv]	H_2_O [equiv]	Compound **5a** (yield)^b^	Compound **6a** (yield)^b^

1	0.80	0.60	5.6.	56%	–
2^c^	0.80	0.60	5.6	26%	–
3^d^	0.80	0.60	5.6	56%	–
4	0.80	0.60	–	44%	–
5	–	0.60	–	26%	28%
6	–	0.60	5.6	23%	–
7	0.80	0.60	2.8	40%	–
8	0.80	0.60	8.4	40%	20%
9	0.80	0.60	11	38%	28%
10	1.0	0.60	5.6	45%	–
11	0.80	0.80	5.6	43%	–

^a^All reactions were carried out on a 0.500 mmol scale (*c*_0_(**3a**) = 0.50 M, *c*_0_(**4**) = 2.0 M; ^b^all yields refer to isolated and purified products; ^c^*c*_0_(**4**) = 1.0 M; ^d^additional 4.0 equiv of ethyl cyanoacetate (**4**) after 2 h.

**Scheme 5:**
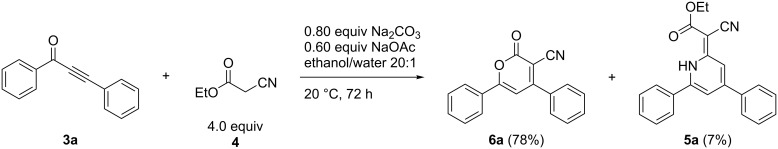
Formation of α-pyrone **6a** and 1*H*-pyridine **5a** at 20 °C.

Only lowering the reaction temperature to 20 °C α-pyrone **6a** was isolated as the main product in 78% yield and 1*H*-pyridine **5a** was obtained in only 7% yield (Scheme 5).

**Scheme 6:**
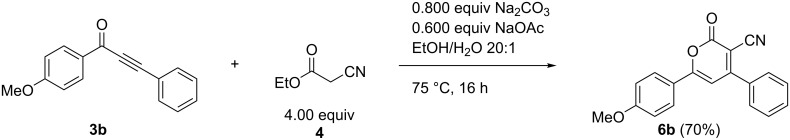
Formation of α-pyrone **6a** starting from alkynone **3b** having an electron-donating substituent.

Since base(s) and reaction temperature exert a significant impact on which heterocyclic compound is formed, we also tried to change the electronic nature of the starting material. Therefore, an electron-donating substituent was introduced in the alkynone **3b** and the reaction was performed at 75 °C. To our surprise, we only could isolate α-pyrone **6b** (Scheme 6).

**Scheme 7:**
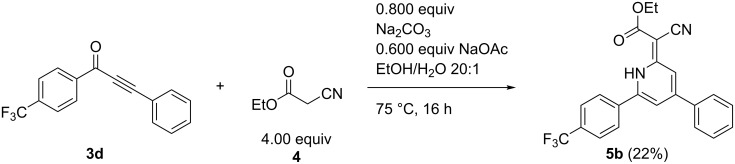
Formation of 1*H*-pyridine **5b** starting from alkynone **3d** having an electron-withdrawing substituent.

However, when we introduced an electron-withdrawing group 1*H*-pyridine **5b** was the only product (Scheme 7).

**Scheme 8:**
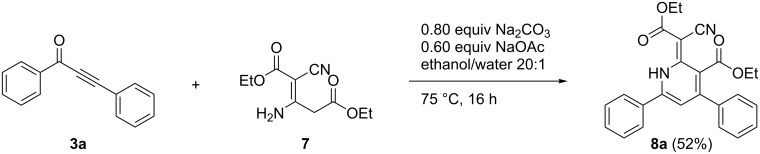
Formation of 1*H*-pyridine **8a** by Michael addition–cyclocondensation reaction.

For elucidating whether 1*H*-pyridine **5a** is formed from α-pyrone **6a** and ethyl cyanoacetate (**4**) a reaction between α-pyrone **6a** and ethyl cyanoacetate (**4**) under the same reaction conditions as for the 1*H*-pyridine from alkynone **3a** was conducted, but only starting material could be isolated. Another option for the formation of the 1*H*-pyridine **5a** was envisioned by an in situ generation of a dimer of ethyl cyanoacetate (**4**). The dimer **7** can be synthesized by iridium catalysis [30]. With dimer **7** in hand, we performed the reaction at 75 °C for 16 h, but we only could isolate 1*H*-pyridine **8a**, which still contains an ester group (Scheme 8). Therefore, the in situ formation of the dimer starting from the alkynone **3a** and ethyl cyanoacetate (**4**) was excluded for the formation of the 1*H*-pyridine **5a**.

While the in situ generation of dimer **7** does not happen during the formation of 1*H*-pyridine **5a**, we examined the reaction between ethyl cyanoacetate (**4**) and the optimized base system by adding alkynone **3a** to the reaction after different times (Table 3).

**Table 3:** Influence of the reaction time on the self-condensation of ethyl cyanoacetate (**4**) in the presence of the optimized base system.^a^

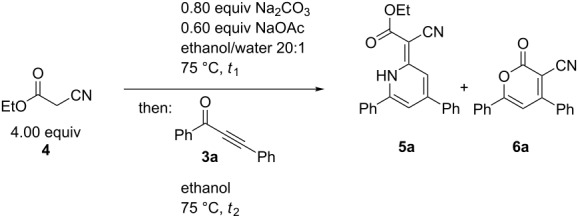				

Entry	*t*_1_	*t*_2_	Compound **5a** (yield)^b^	Compound **6a** (yield)^b^

1	2 h	16 h	53%	–
2	6 h	16 h	5%	46%
3	24 h	16 h	3%	2%

^a^All reactions were carried out on a 0.500 mmol scale (*c*_0_(**3a**) = 0.50 M, *c*_0_(**4**) = 2.0 M; ^b^all yields refer to isolated and purified products.

In the first attempt, alkynone **3a** was added after 2 h. 1*H*-Pyridine **5a** was isolated in 53% yield (Table 3, entry 1), indicating that the time of addition of the alkynone is not relevant within the first two hours of the reaction. However, if alkynone **3a** was added after 6 h α-pyrone **6a** was the main product and 1*H*-pyridine **5a** could only be isolated in 5% yield (Table 3, entry 2). Upon the addition of alkynone **3a** after 24 h, both 1*H*-pyridine **5a** and α-pyrone **6a** were isolated in only around 3% yield. This finding supports that within the first two hours ethyl cyanoacetate (**4**) is consumed and thereafter the ethyl cyanoacetate concentration is just too low for the formation of 1*H*–pyridine **5a**, therefore α-pyrone **6a** is formed. At longer initial reaction times (6 and 24 h) there is no ethyl cyanoacetate (**4**) left for the formation of any product. Also, ethyl cyanoacetate (**4**) probably does not form dimer **7** because in that case under these conditions 1*H*-pyridine **8a** would have been detected.

**Scheme 9:**
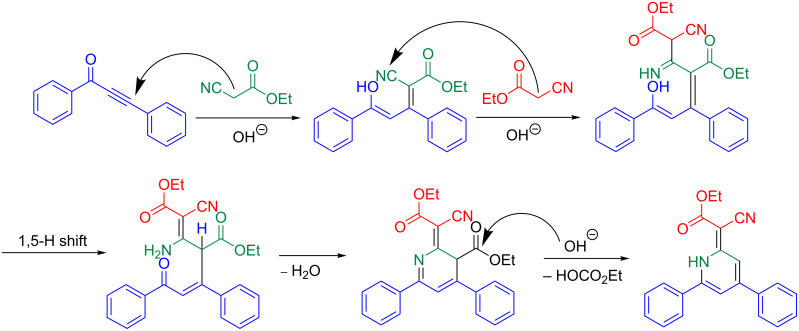
Mechanistic rationale for the formation of the 1*H*-pyridine **5a**.

Therefore, the tentative mechanistic rationale takes into account that the formation of 1*H*-pyridine **5a** rather proceeds via stepwise condensation of alkynone **3** with two equivalents of ethyl cyanoacetate (**4**) than by reaction with dimer **7** (Scheme 9).

First, a molecule of ethyl cyanoacetate (**4**) attacks the alkynone **3a** in a Michael addition. A second molecule **4** then attacks the cyano substituent and an imine is formed. The ester substituent of the initially reacted more electrophilic ethyl cyanoacetate (**4**) is presumably cleaved by a base-mediated acyl cleavage furnishing directly 1*H*-pyridine **5a** after protonation.

For examining the influence of the electronic nature of the alkynone **3** on the product formation, a range of differently substituted alkynones **3** (for experimental details on their preparation, see chapters 2.1 and 2.2 in Supporting Information File 1) bearing electron-donating and/or electron-withdrawing substituents were synthesized and employed in the cyclocondensation step under the optimized reaction conditions [32–34]. Alkynones **3b–e** with only one electron-donating substituent furnish the corresponding α-pyrones **6b–e**, while the alkynone with a single electron-withdrawing substituent furnishes 1*H*-pyridines **5b–e**. Interestingly, the position of substitution on the alkynone does not affect the outcome (Table 4, entries 2 and 6). Also, for alkynone **3j** bearing an electron-donating substituent on either aryl ring, α-pyrone **6f** is formed likewise (Table 4, entry 10). For electronically unsymmetrically substituted alkynones **3** the product formation depends rather on the strength of the employed electron-donating group. Whereas the *p*-anisyl substituent leads to the formation of 1*H*-pyridine (Table 4, entries 11 and 12), the *N*,*N*-dimethylaminophenyl substituent furnishes α-pyrone **6g** (Table 4, entry 13).

**Table 4:** Michael addition–cyclocondensation synthesis of 1*H*-pyridine **5** or α-pyrone **6**.

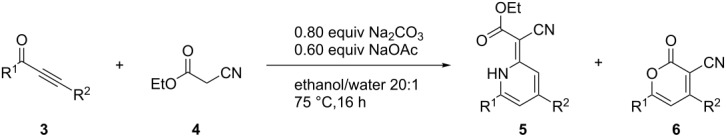			

Entry	Alkynone **3**	1*H*-Pyridine **5**^a^	α-Pyrone **6**^a^

1	**3a** (R^1^ = Ph, R^2^ = Ph)	**5a** (R^1^ = Ph, R^2^ = Ph, 56%)	–
2	**3b** (R^1^ = *p*-MeOC_6_H_4_, R^2^ = Ph)	–	**6b** (R^1^ = *p*-MeOC_6_H_4_, R^2^ = Ph; 70%)
3	**3c** (R^1^ = *p*-Me_2_NC_6_H_4_, R^2^ = Ph)	–	**6c** (R^1^ = *p*-Me_2_NC_6_H_4_, R^2^ = Ph, 12%)
4	**3d** (R^1^ = *p*-F_3_CC_6_H_4_, R^2^ = Ph)	**5b** (R^1^ = *p*-F_3_CC_6_H_4_, R^2^ = Ph, 22%)	–
5	**3e** (R^1^ = *p*-NCC_6_H_4_, R^2^ = Ph)	**5c** (R^1^ = *p*-NCC_6_H_4_, R^2^ = Ph, 20%)	–
6	**3f** (R^1^ = Ph, R^2^ = *p*-MeOC_6_H_4_)	–	**6d** (R^1^ = Ph, R^2^ = *p*-MeOC_6_H_4_, 82%)
7	**3g** (R^1^ = Ph, R^2^ = *p*-Me_2_NC_6_H_4_)	–	**6e** (R^1^ = Ph, R^2^ = *p*-Me_2_NC_6_H_4_, 62%)
8	**3h** (R^1^ = Ph, R^2^ = *p*-F_3_CC_6_H_4_)	**5d** (R^1^ = Ph, R^2^ = *p*-F_3_CC_6_H_4_, 25%)	–
9	**3i** (R^1^ = Ph, R^2^ = *p*-NCC_6_H_4_)	**5e** (R^1^ = Ph, R^2^ = *p*-NCC_6_H_4_, 2%)	**–**
10	**3j** (R^1^ = *p*-MeOC_6_H_4_, R^2^ = *p*-MeOC_6_H_4_)	–	**6f** (R^1^ = *p*-MeOC_6_H_4_, R^2^ = *p*-MeOC_6_H_4_, 45%)
11	**3k** (R^1^ = *p*-MeOC_6_H_4_, R^2^ = *p*-F_3_CC_6_H_4_)	**5f** (R^1^ = *p*-MeOC_6_H_4_, R^2^ = *p*-F_3_CC_6_H_4_, 37%)	–
12	**3l** (R^1^ = *p*-F_3_CC_6_H_4_, R^2^ = *p*-MeOC_6_H_4_)	**5g** (R^1^ = *p*-F_3_CC_6_H_4_, R^2^ = *p*-MeOC_6_H_4_,, 40%)	–
13	**3m** (R^1^ = *p*-F_3_CC_6_H_4_, R^2^ = *p*-Me_2_NC_6_H_4_)	–	**6g** (R^1^ = *p*-F_3_CC_6_H_4_, R^2^ = *p*-Me_2_NC_6_H_4_, 71%)
14	**3n** (R^1^ = 2-thienyl, R^2^ = Ph)	**5h** (R^1^ = 2-thienyl, R^2^ = Ph, 51%)	–

^a^All yields refer to isolated and purified products.

**Scheme 10:**
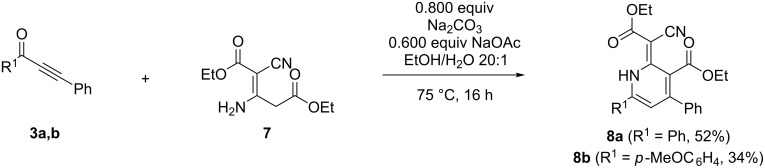
Formation of 1*H*-pyridine **8a** from alkynone **3b** and dimer **7**.

For synthesizing 1*H*-pyridine derivatives **8** with an electron-donating group we employed the isolated dimer **7** and were able to isolate 1*H*-pyridines **8** in 52 and 34% yield (Scheme 10).

### Crystal structure of 1*H*-pyridine **5a**

**Figure 1:**
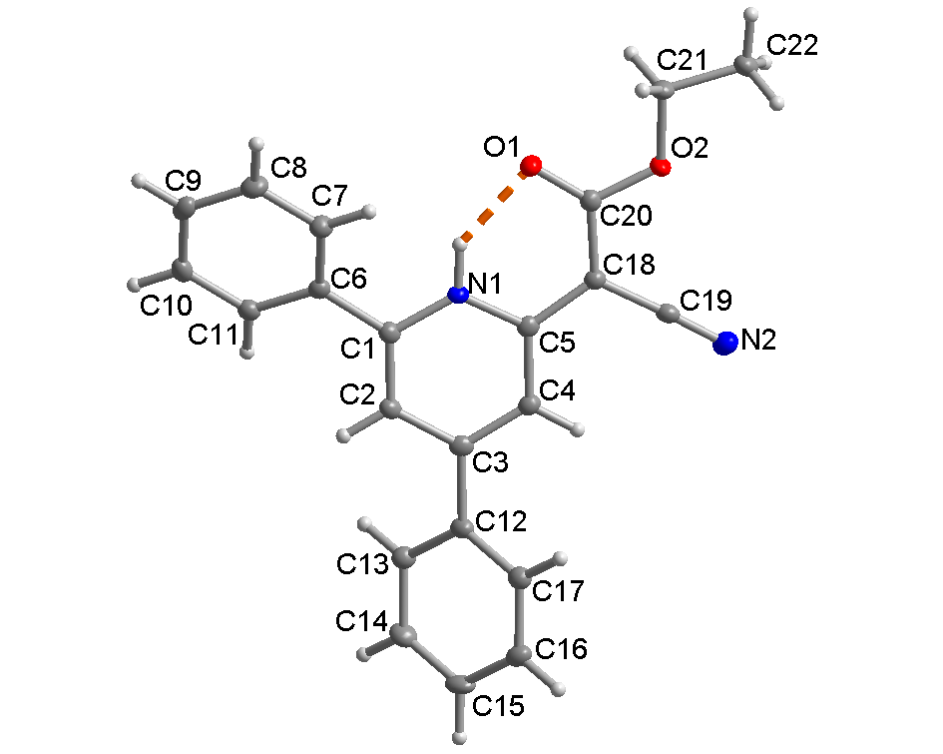
Molecular structure of 1*H*-pyridine **5a** (50% thermal ellipsoids), showing the intramolecular N–H···O bond as dashed orange line. H-bond details N1–H 0.90(2) Å, H···O1 1.87(2) Å, N1···O2 2.624(2) Å, O1–H···O2 140(1)°.

**Figure 2:**
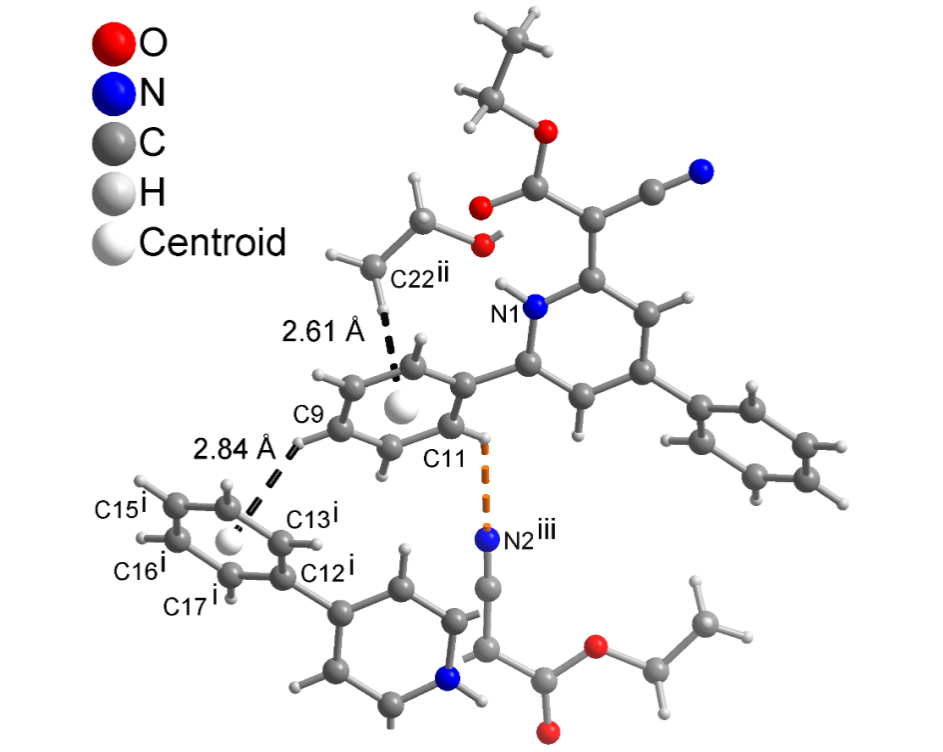
Supramolecular C–H···N [36–39] and C–H···π [40–49] interactions around the 6-positioned phenyl ring in **5a**. Details of C–H···N bond (dashed orange line) C11–H 0.95 Å, H···N2 2.61 Å, C11···N2 3.263(2) Å, C11–H···N2 127°. Symmetry transformations are i = 1−x, 1−y, 1−z; ii = x, 3/2−y, −1/2+z, iii = 1−x, −1/2+y, −1/2−z.

The structure of 1*H*-pyridines **5** was further corroborated by a single crystal X-ray structure determination of compound **5a** (Figure 1) [35]. In the single crystal the carboxyl ester group is oriented to the N–H and via a hydrogen bond. Solid-state torsional/dihedral angles between the 4- and 6-positioned aryl rings differ especially for the 6-positioned phenyl ring with 27° in the X-ray structure and 38° from calculation (for comparison to the DFT calculated ground state structure of 1*H*-pyridine **5a**, see chapter 12.3 in Supporting Information File 1). This is probably due to packing constraints from the involvement of the 6-phenyl ring in C-H···N [36–39] and C-H···π [40–49] interactions (Figure 2, for details, see Supporting Information File 1). It is noteworthy to mention that there are no significant π···π interactions in the solid-state structure of **5a** (for details, see Supporting Information File 1) [50–57].

### Photophysical properties

#### Photophysical properties of 1*H*-pyridines **5** and **8**

**Figure 3:**
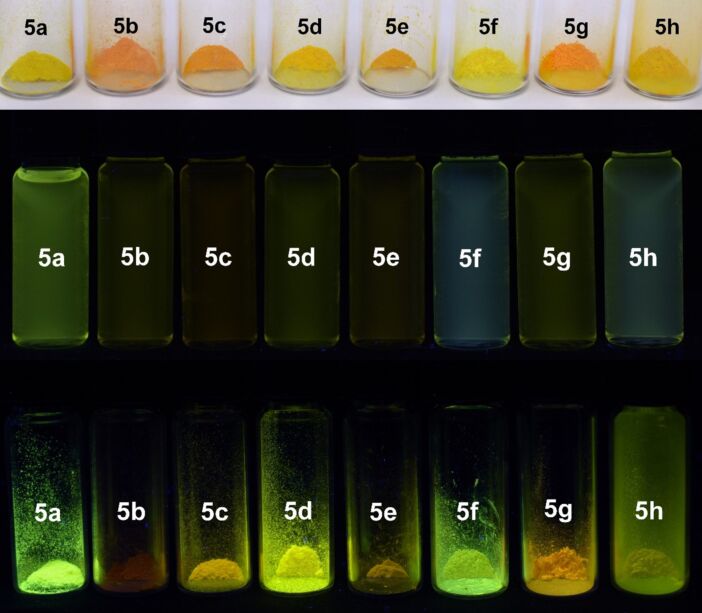
1*H*-Pyridine derivatives **5** as solids under daylight (top), under UV light (λ_exc_ = 365 nm, *c*(**5**) = 10^−4^ M) in dichloromethane solution (center), and under UV light (λ_exc_ = 365 nm) in the solid state (bottom).

**Figure 4:**
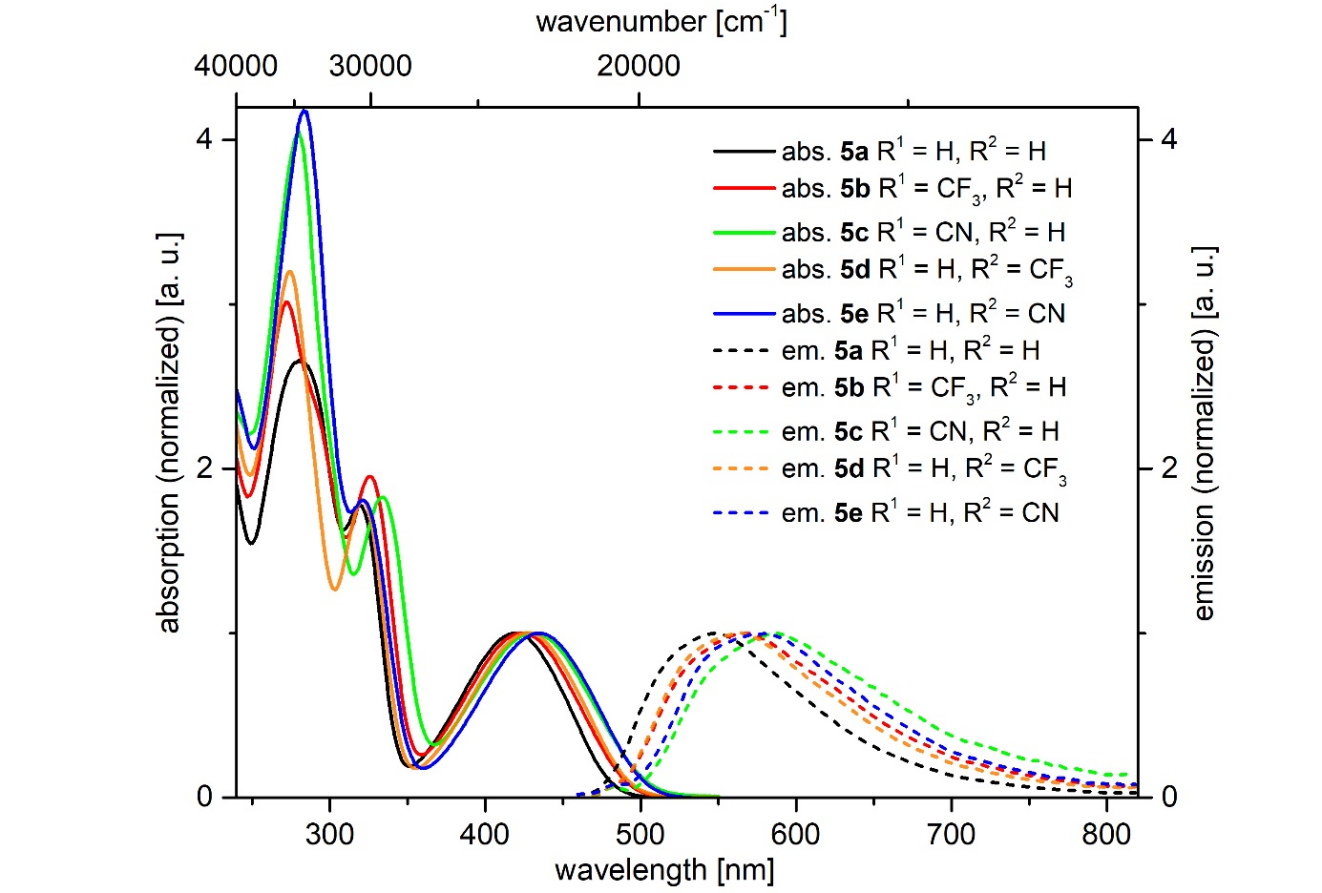
Selected normalized absorption (solid lines) and emission (dashed lines) spectra of 1*H*-pyridines **5a–e** (recorded in dichloromethane at *T* = 298 K).

1*H*-Pyridine derivatives **5** are yellow or orange compounds under daylight (Figure 3, top) and fluoresce in solution (Figure 3, center) and in the solid state (Figure 3, bottom). Therefore, the photophysical properties were studied by absorption and emission spectroscopy (Figure 4, Table 5).

**Table 5:** Photophysical properties of 1*H*-pyridines **5**.

Entry	Compound	R^1^	R^2^	λ_max,abs_ [nm]^a^ (*ε* [L·mol^−1^·cm^−1^])	λ_max,em_ [nm]^b^ (Φ_f_)^c^	Stokes shift Δν̃ [cm^−1^]

1	**5a**	Ph	Ph	281 (26100), 319 (17400), 418 (9800)	545 (0.02)	5600
2	**5b**	*p*-F_3_CC_6_H_4_	Ph	272 (27000), 326 (17500), 424 (8900)	565 (0.01)	5600
3	**5c**	*p*-NCC_6_H_4_	Ph	280 (37600), 333 (17300), 431 (9500)	585 (0.01)	6100
4	**5d**	Ph	*p*-F_3_CC_6_H_4_	274 (32000), 321 (18100), 428 (10000)	565 (0.01)	5700
5	**5e**	Ph	*p*-NCC_6_H_4_	283 (36000), 322 (15600), 434 (8600)	579 (0.01)	5800
6	**5f**	*p*-MeOC_6_H_4_	*p*-F_3_CC_6_H_4_	261 (26200), 306 (28400), 429 (10400)	557 (0.02)	5400
7	**5g**	*p*-F_3_CC_6_H_4_	*p*-MeOC_6_H_4_	260 (20300), 324 (43800), 420 (10000)	562 (0.02)	6000
8	**5h**	2-thienyl	Ph	273 (21800), 308 (26700), 433 (9200)	560 (0.03)	5200

^a^Recorded in dichloromethane, *T* = 293 K, *c*(**5**) = 10^−6^ M; ^b^recorded in dichloromethane, *T* = 293 K, *c*(**5**) = 10^−7^ M; ^c^fluorescence quantum yields were determined relative to coumarin153 (Φ_f_ = 0.54) as a standard in ethanol [58].

All compounds show three absorption maxima at around 275, 320 and 430 nm, where the longest wavelength absorption maxima exhibit extinction coefficients of around 9500 L·mol^−1^·cm^−1^ (Table 5). Upon introducing electron-withdrawing substituents on the aryl rings the longest wavelength maxima shift bathochromically (Table 5, entries 2–5). The redshift qualitatively corresponds with the strength of the acceptor group (Table 5, entries 3 and 5). However, as can be seen from entries 2–5 (Table 5), the placement of the acceptor group at the 4 or 6-aryl substituent does not affect the absorption energies. This situation changes to a minor extent upon placing an additional donor substituent at the remaining phenyl substituent (Table 5, entries 6 and 7). A thienyl substituent instead of a phenyl substituent causes a redshift of the longest wavelength absorption maximum (Table 5, entries 1 and 8).

Upon excitation at the longest wavelength absorption band dichloromethane solutions of all compounds **5** fluoresce with emission maxima at around 565 nm (Table 5, entries 2–5). Upon the introduction of electron-withdrawing substituents the maxima are shifted bathochromically, similarly as the absorption maxima, and the shift is qualitatively correlated with the acceptor strength. In comparison, the introduction of another electron-donating substituent does not significantly change the luminescence characteristics (Table 5, entries 2, 4, 6 and 7). The Stokes shifts fall in a range between 5000 and 6100 cm^−1^ and the fluorescence quantum yields of the 1*H*-pyridines **5** account between 1 and 3%.

**Figure 5:**
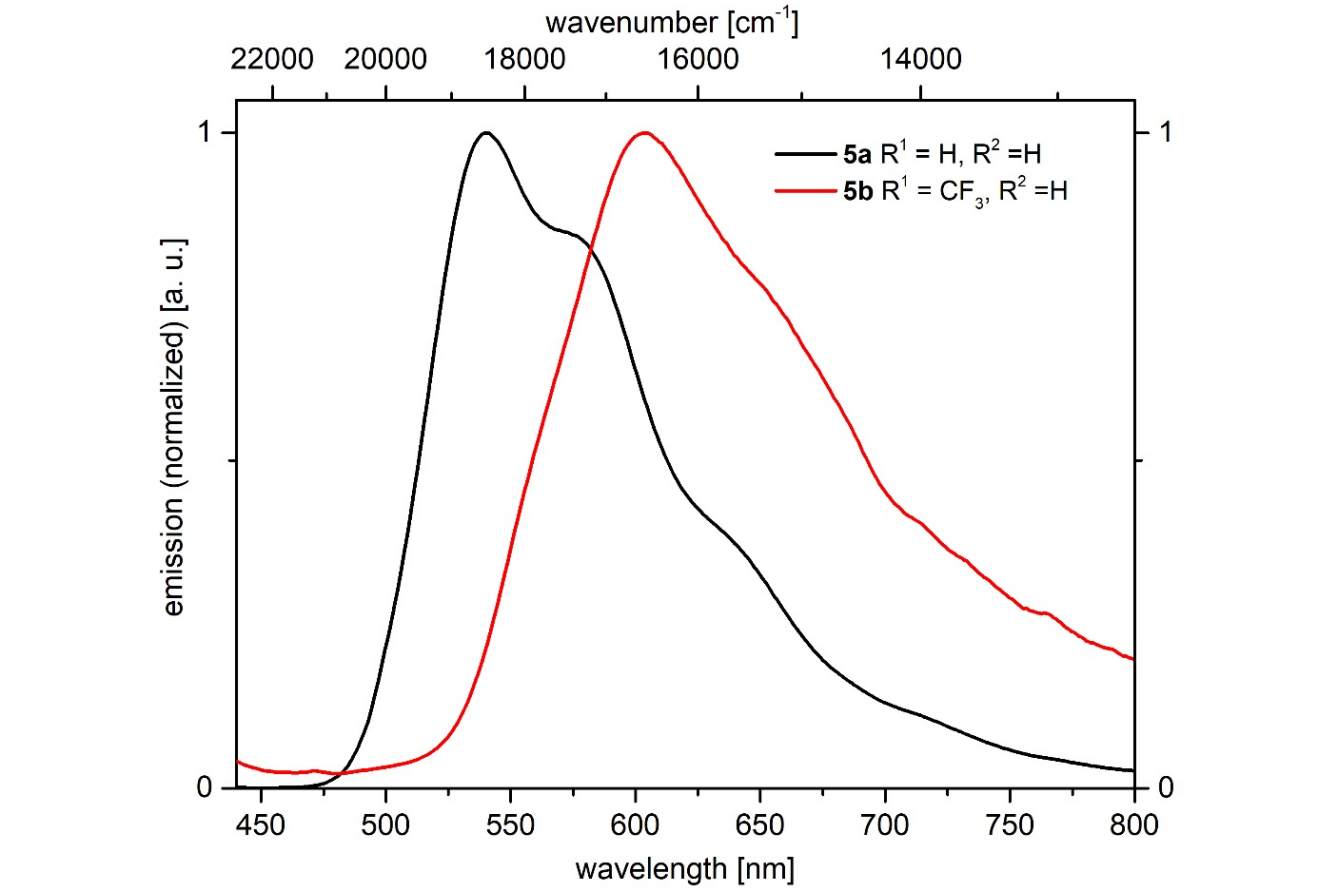
Selected normalized emission spectra of 1*H*-pyridine **5a** and **5b** in the solid state at *T* = 298 K.

Besides solution fluorescence all 1*H*-pyridines **5** also luminesce in the solid state (Figure 3, bottom). The emission maxima of two selected 1*H*-pyridines **5** were determined (Figure 5, Table 6), showing a similar behavior in the solid state as in solution. The emission maximum of the unsubstituted 1*H*-pyridine **5a** appears at 540 nm, while the CF_3_-substituted 1*H*-pyridine **5b** emits bathochromically shifted at 604 nm.

**Table 6:** Photophysical properties of 1*H*-pyridines **5a** and **5b** in the solid state.

Compound	R^1^	R^2^	λ_max,em_ [nm]^a^

**5a**	H	H	540
**5b**	CF_3_	H	604

^a^λ_exc_ = 420 nm.

**Figure 6:**
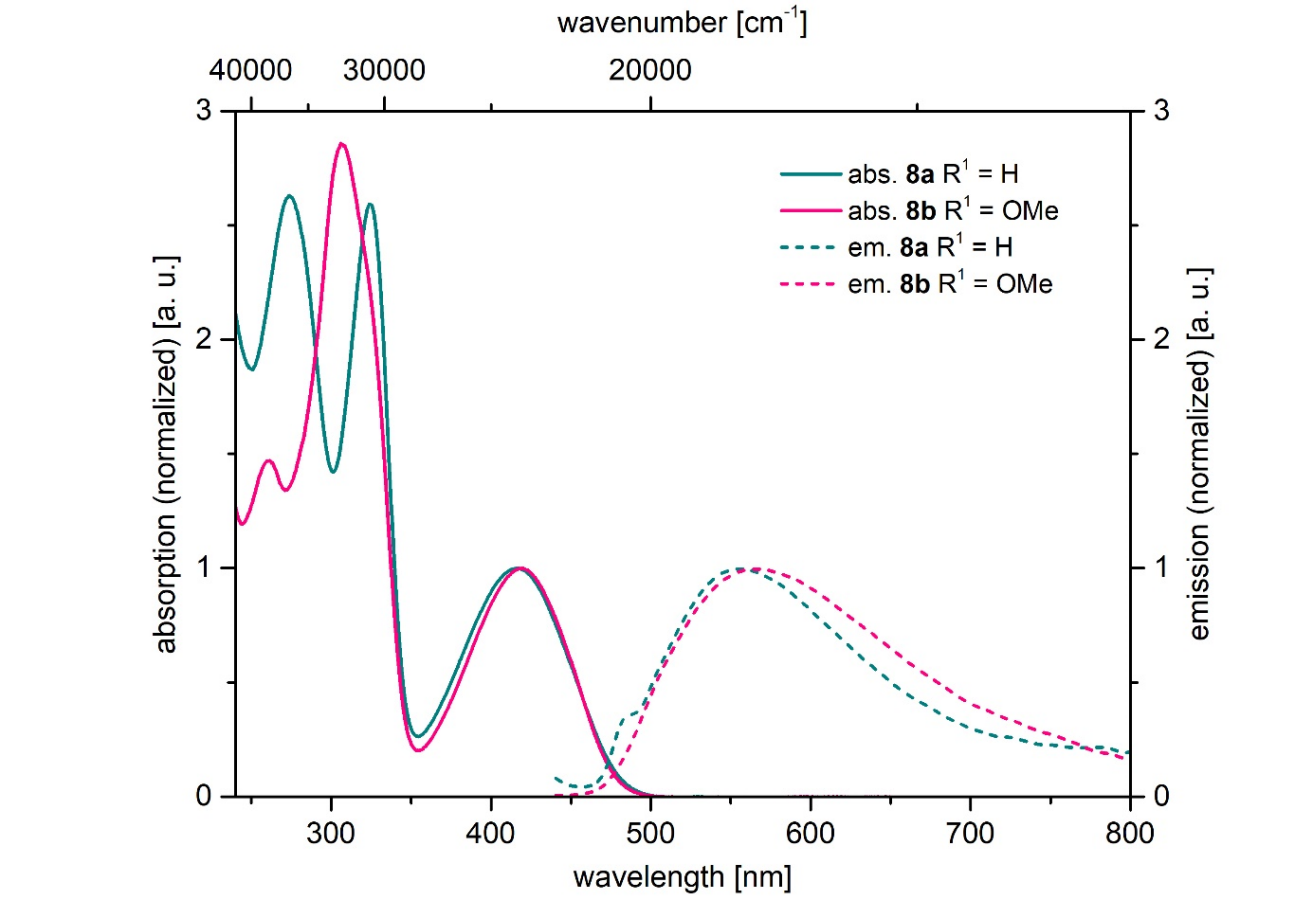
Selected normalized absorption (solid lines) and emission (dashed lines) spectra of 1*H*-pyridines **8a** and **8b** (recorded in dichloromethane at *T* = 298 K).

In addition, both ester-substituted 1*H*-pyridines **8a** and **8b** also possess interesting photophysical properties (Figure 6, Table 7). Under daylight they are yellow and they fluoresce in solution and in the solid state. The three absorption maxima are found at around 270, 315 and 415 nm. The methoxy group in the spectrum of compound **8b** only has a minor influence on the absorption maximum, however, slightly more on the emission maximum. The fluorescence quantum yields Φ_f_ of both compounds are lower than 1%.

**Table 7:** Photophysical properties of 1*H*-pyridines **8**.

Compound	R^1^	R^2^	λ_max,abs_ [nm]^a^ (ε [L·mol^−1^·cm^−1^])	λ_max,em_ [nm]^b^ (Φ_f_)^c^	Stokes shift Δ ν̃ [cm^−1^]

**8a**	Ph	Ph	274 (20300), 324 (20100), 417 (7700)	557 (<0.01)	6000
**8b**	*p*-MeOC_6_H_4_	Ph	261 (16200), 307 (31300), 419 (11000)	565 (<0.01)	6200

^a^Recorded in dichloromethane, *T* = 293 K, *c*(**8**) = 10^−6^ M; ^b^recorded in dichloromethane, *T* = 293 K, *c*(**8**) = 10^−7^ M (λ_exc_ = 420 nm); ^c^fluorescence quantum yields were determined relative to coumarin153 (Φ_f_ = 0.54) as a standard in ethanol [58].

**Figure 7:**
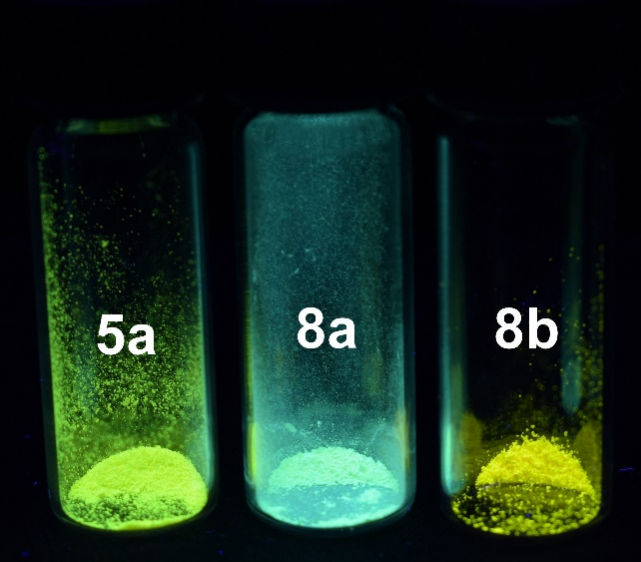
Solid-state luminescence of 1*H*-pyridines **5a**, **8a** and **8b** (λ_exc_ = 365 nm).

With the addition of the second ester group in the 3-position to 1*H*-pyridine **8a** the fluorescence in the solid state appears to shift to blue. If the phenyl substituent in the 4-position bears an additional methoxy substituent the fluorescence of the 1*H*-pyridine **8b** appears yellow again (Figure 7).

#### Photophysical properties of α-pyrones **6**

**Figure 8:**
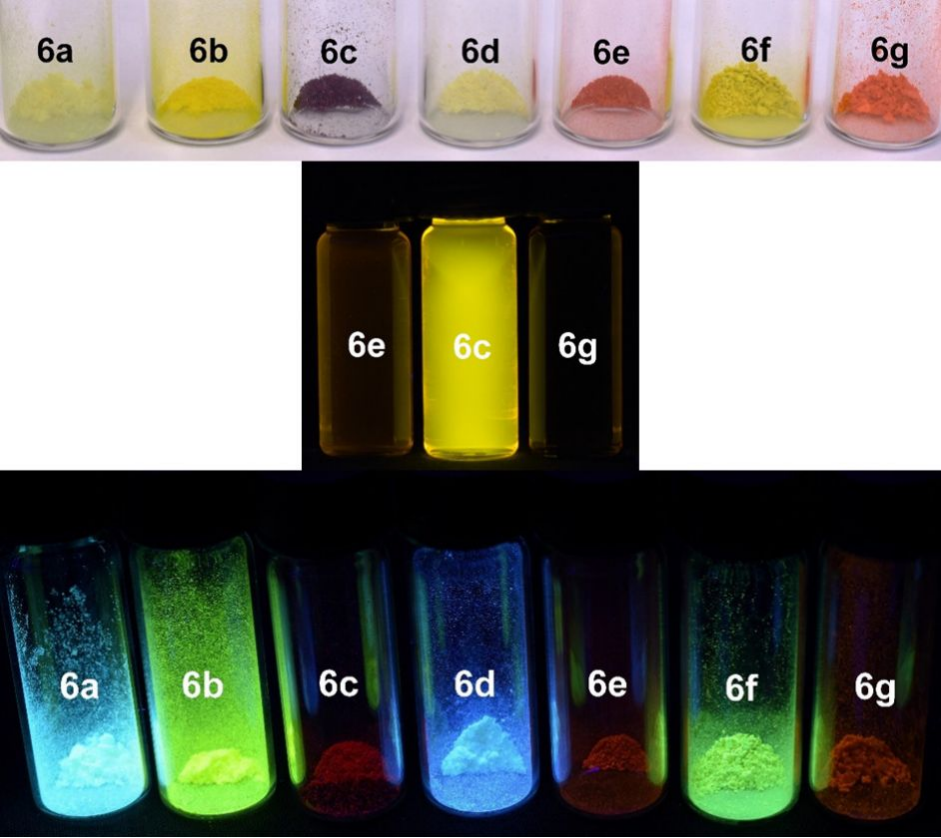
α-Pyrones **6** as solids under daylight (top), selected derivatives under UV light (λ_exc_ = 365 nm, *c*(**6**) = 10^−4^ M) in dichloromethane solution (center), and under UV light (λ_exc_ = 365 nm) in the solid state (bottom).

**Figure 9:**
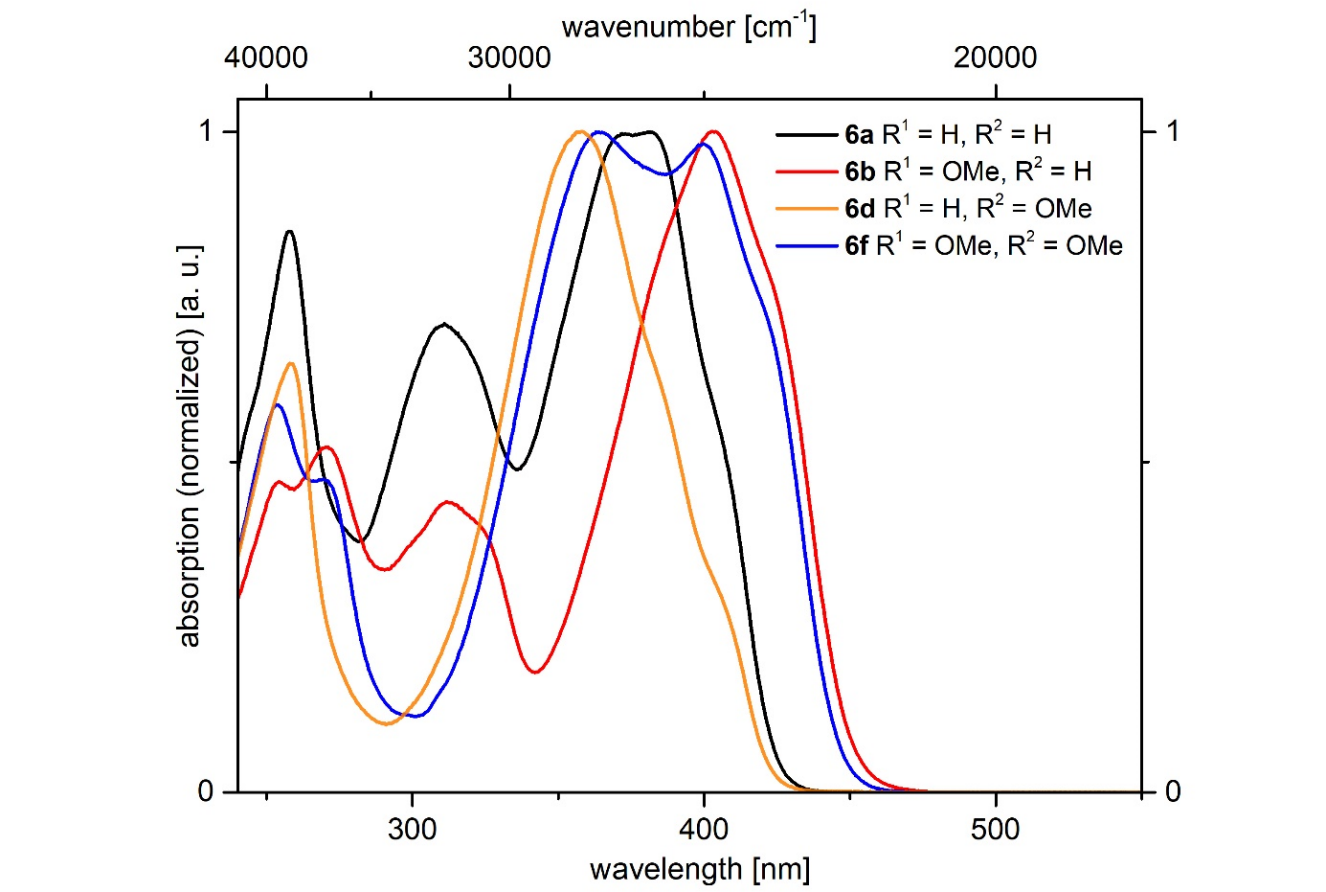
Selected normalized absorption spectra of α-pyrones **6a**, **6b**, **6d**, and **6e** recorded in dichloromethane at *T* = 298 K.

All α-pyrone derivatives **6** are yellow or red under daylight (Figure 8, top) and some of them fluoresce in solution (Figure 8, center) and in the solid state (Figure 8, bottom). Therefore, the photophysical properties were studied by absorption and emission spectroscopy (Figure 9, Table 8).

**Table 8:** Photophysical properties of α-pyrones **6**.

Entry	Compound	R^1^	R^2^	λ_max,abs_ [nm]^a^ (ε [L·mol^−1^·cm^−1^])	λ_max,em_ [nm]^b^ (Φ_f_)^c^	Stokes shift Δν̃ [cm^−1^]

1	**6a**	Ph	Ph	258 (15300), 311 (12800), 381 (18000)	–	–
2	**6b**	*p*-MeOC_6_H_4_	Ph	254 (10300), 271 (11500), 312 (9600), 404 (21900)	–	–
3	**6c**	*p*-Me_2_NC_6_H_4_	Ph	294 (18800), 482 (47300)	567 (0.99)	3100
4	**6d**	Ph	*p*-MeOC_6_H_4_	258 (19700), 358 (30400)	–	–
5	**6e**	Ph	*p*-Me_2_NC_6_H_4_	255 (25600), 289 (23300), 375 (39600), 453 (40600)	634 (0.01)	6300
6	**6f**	*p*-MeOC_6_H_4_	*p*-MeOC_6_H_4_	254 (15000), 364 (25600), 400 (25100)	–	–
7	**6g**	*p*-F_3_CC_6_H_4_	*p*-Me_2_NC_6_H_4_	251 (16200), 309 (13900), 372 (20200), 465 (21600)	673 (<0.01)	6600

^a^Recorded in dichloromethane, *T* = 293 K, *c*(**6**) = 10^−6^ M; ^b^recorded in dichloromethane, *T* = 293 K, *c*(**6**) = 10^−7^ M (λ_exc_ = 465 nm); ^c^fluorescence quantum yields were determined relative to DCM (Φ_f_ = 0.435) as a standard in ethanol [58].

All compounds show 2–4 absorption maxima and the shortest wavelength maxima appear at around 255 nm. The unsubstituted α-pyrone **6a** exhibits its longest wavelength maximum at 381 nm (Table 8, entry 1). A *p*-methoxyphenyl substituent in the 6-position causes a bathochromic shift (Table 8, entry 2), whereas the same substituent in 4-position leads to a hypsochromic shift (Table 8, entry 4). Interestingly, *p*-methoxyphenyl substituents at positions 4 and 6 split the longest absorption band into two maxima at 358 nm (arising from the *p*-methoxyphenyl substituent in the 4-position and at 400 nm arising from the *p*-methoxyphenyl substituent in the 6-position) (Table 8, entry 6). The introduction of a more strongly electron-donating substituent, such as *N*,*N*-dimethylaminophenyl, causes a significant bathochromic shift (Table 8, entries 3 and 5). Donor–acceptor substitution in positions 4 and 6 causes a further bathochromic shift (Table 8, entry 7).

**Figure 10:**
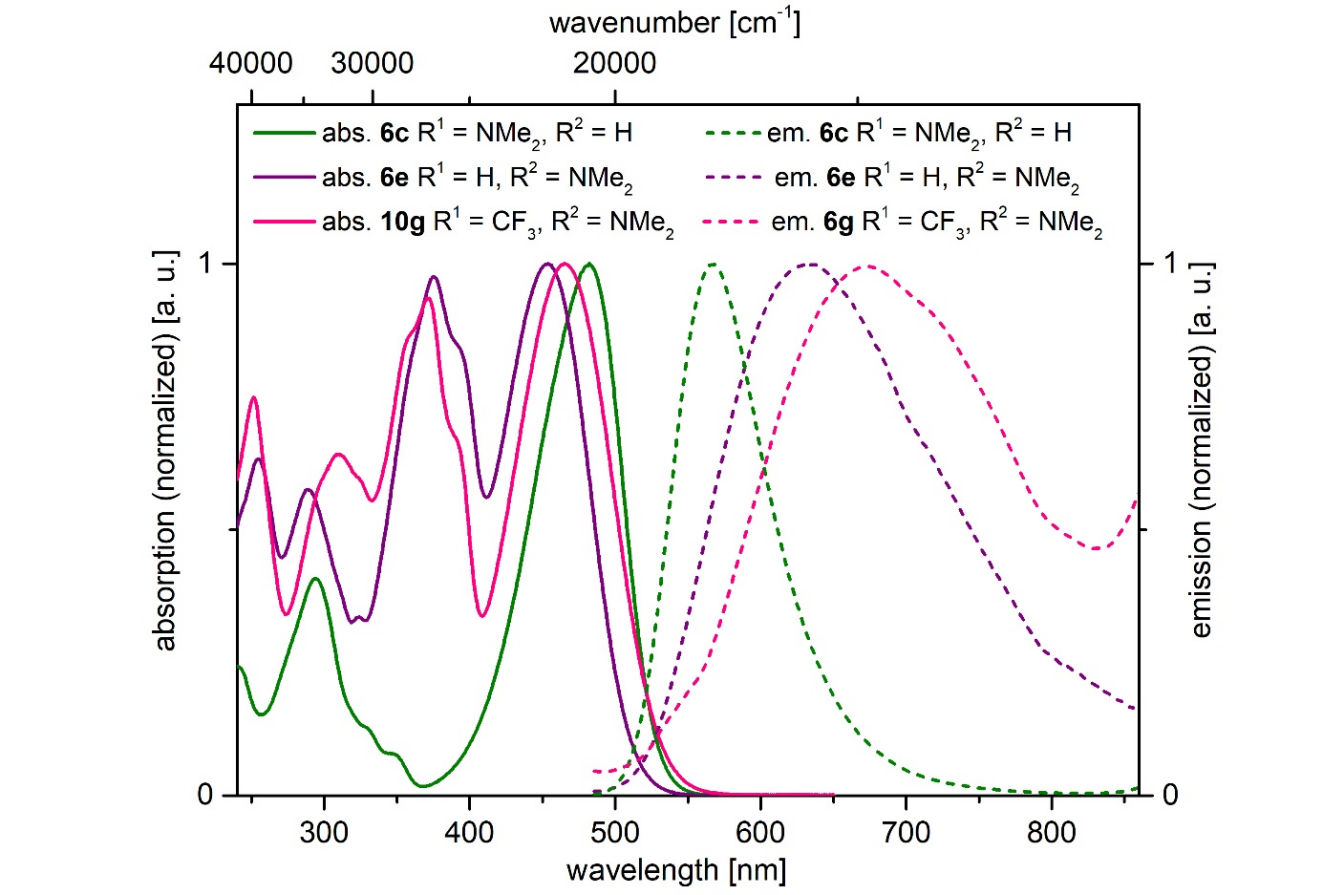
Selected normalized absorption (solid lines) and emission (dashed lines) spectra of α-pyrones **6c**, **6e**, and **6g** recorded in dichloromethane at *T* = 298 K.

In solution only *N*,*N*-dimethylaminophenyl-substituted derivatives fluoresce (Figure 10). While the 6-substituted α-pyrone **6c** has a fluorescence maximum at 567 nm, the one for the regioisomer **6e** is shifted bathochromically to 634 nm (Table 8, entries 3 and 5). Donor–acceptor substitution in positions 4 and 6 causes a further bathochromic shift to 673 nm (Table 8, entry 7). Most remarkably, the regioisomers **6c** and **6e** differ quite significantly with respect to their fluorescence quantum yields Φ_f_. While chromophore **6e** only emits with an efficiency of 1%, the regioisomer **6c** accounts for an extraordinarily high relative quantum yield of 99%.

**Figure 11:**
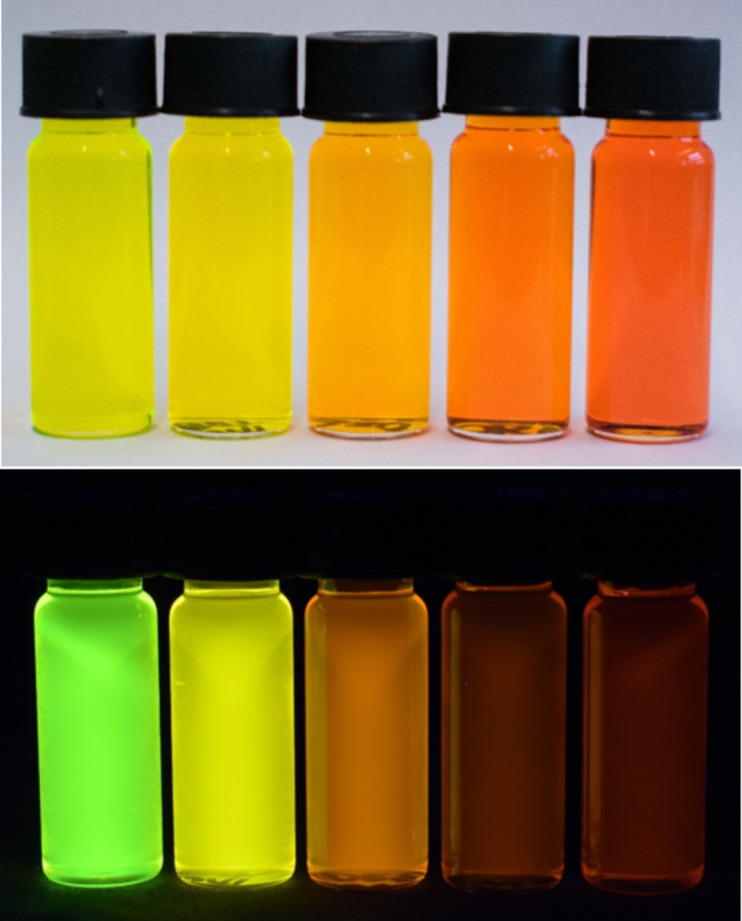
Absorption (top) and fluorescence (bottom) of compound **6c** with variable solvent polarity (left to the right: toluene, ethyl acetate, acetone, DMF and DMSO, *c*(**6c**) = 10^−4^ M; λ_exc_ = 365 nm, handheld UV lamp).

**Figure 12:**
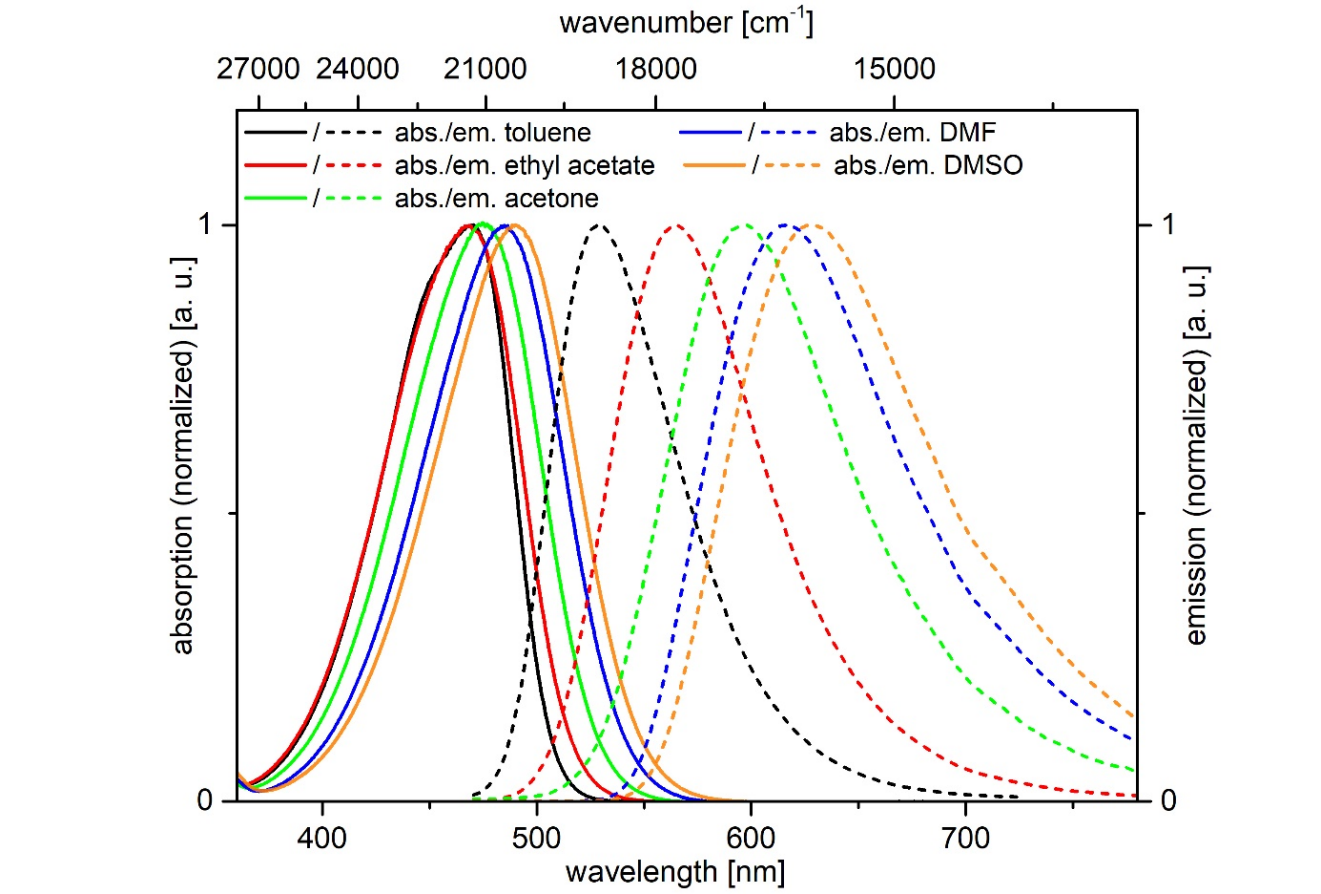
Absorption (solid lines) and emission (dashed lines) spectra of α-pyrone **6c** in five solvents of different polarity (recorded at *T* = 298 K).

**Figure 13:**
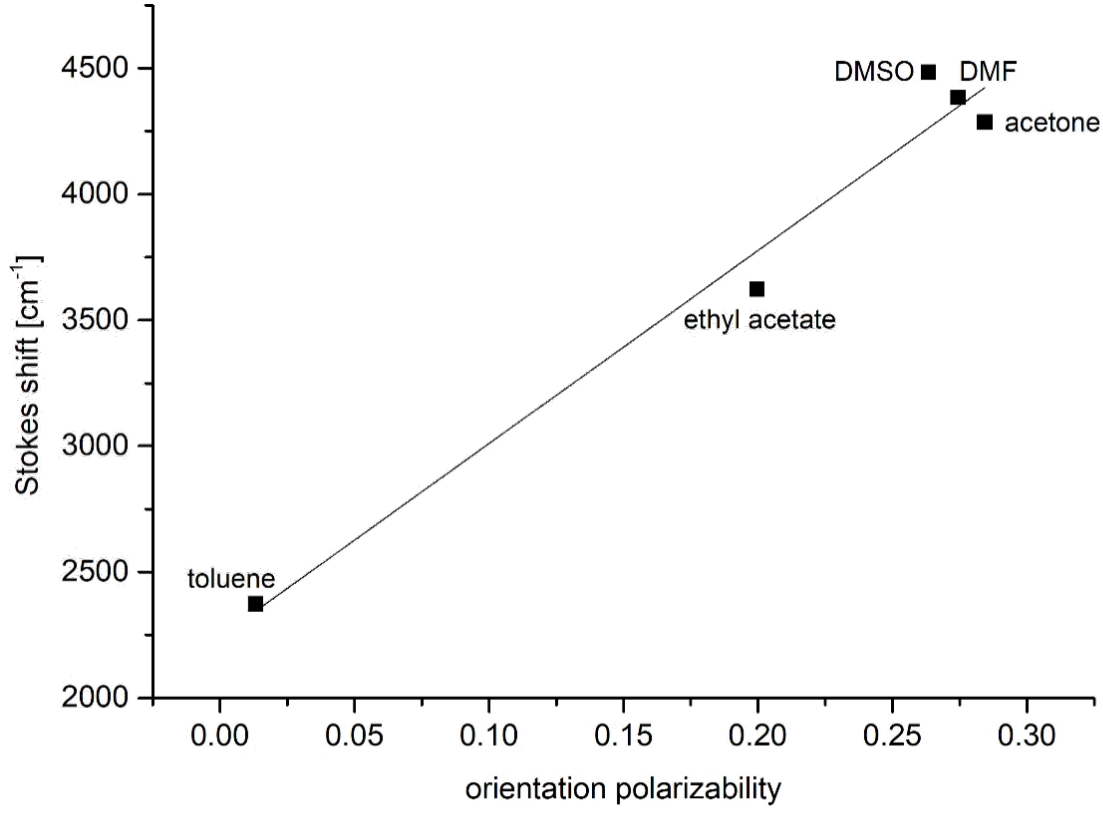
Lippert plot for α-pyrone **6c** (*n* = x, r^2^ = 0.970).

Furthermore, the *N*,*N*-dimethylaminophenyl-derivative **6c** shows a pronounced emission solvatochromism (Figure 11, Table 9). While the polarity effect on the absorption maximum is only minor within a range of the longest wavelength maximum between 469 and 490 nm, the emission maximum is shifted bathochromically with increasing solvent polarity in a range from green fluorescence (529 nm) in toluene to red fluorescence in DMSO (638 nm) (Figure 12). The observed positive emission solvatochromism is a consequence of a significant change in the dipole moment from the electronic ground to the vibrationally relaxed excited state [59]. Plotting Stokes shifts Δν̃ against the orientation polarizabilities Δƒ of the respective solvents (Lippert plot) [60] gives a reasonable linear correlation with a moderate fit of *r^2^* = 0.970 (Figure 13). The orientation polarizabilities Δƒ were calculated according to Equation 1

[1]
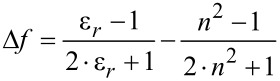


where ε_r_ is the relative permittivity and *n* the optical refractive index of the respective solvent.

The change in dipole from the ground to the excited state can be calculated according to the Lippert–Mataga equation (Equation 2)

[2]
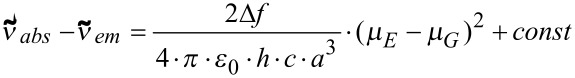


where ν̃_abs_ represents the absorption and ν̃_em_ the emission maxima (in m^−1^), µ_E_ and µ_G_ are the dipole moments in the excited and ground state (in C·m), ε_0_ (8.8542·10^−12^ A·s/V·m) is the vacuum permittivity constant, *h* (6.6256·10^−34^ J·s) is the Planck’s constant, *c* (2.9979·10^10^ cm/s) is the speed of light and *a* is the radius of the solvent cavity occupied by the molecules (in m). The Onsager radius *a*, assuming a spherical dipole to approximate the molecular volume of the molecule in solution, was estimated from the optimized ground-state structure of compound **6c** obtained by DFT calculations. With an *a* value of 5.46 Å, the change in dipole moment was calculated to 11.6 D (3.87·10^−29^ C·m).

**Figure 14:**
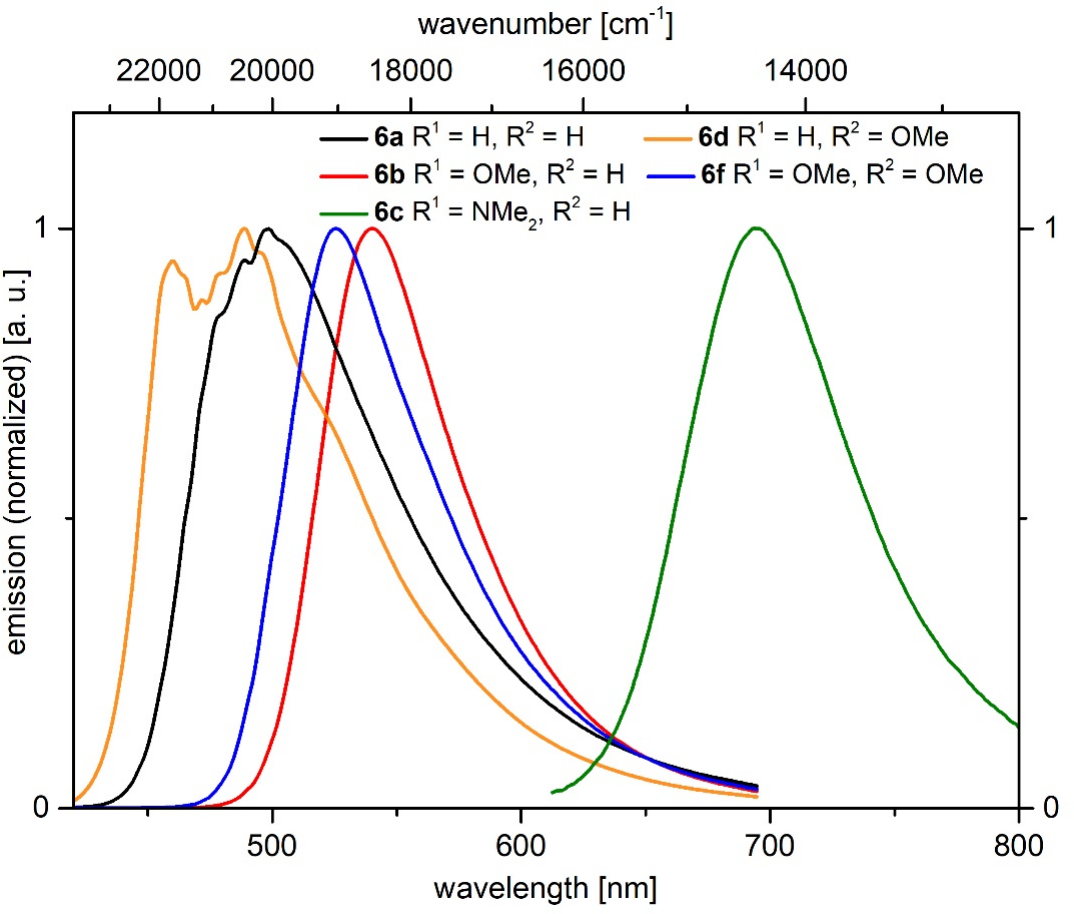
Normalized emission spectra of selected α-pyrones **6a–d**,**f** in the solid state at *T* = 298 K.

All α-pyrones **6** fluoresce in the solid state (Figure 8, bottom) and for five selected α-pyrones **6** the emission maxima were determined (Figure 14, Table 9). The fluorescence maximum of unsubstituted α-pyrone **6a** lies at 499 nm and the maxima of the monomethoxy-substituted regioisomers **6b** (540 nm) and **6d** (489 nm) appear at quite different energies, similar to their corresponding absorption maxima in solution. In comparison to α-pyrone **6a** the introduction of two methoxy substituents in derivative **6f** results in a bathochromic shift to 526 nm. The solid-state emission of *N*,*N*-dimethylaminophenyl derivative **6c** shows an enormous redshift to 694 nm. The solid-state fluorescence quantum yield Φ_f_ of compound **6c** was determined to 11%.

**Table 9:** Photophysical properties of selected α-pyrones **6** in the solid state.

Compound	R^1^	R^2^	λ_max,em_ [nm]^a^

**6a**	Ph	Ph	499
**6b**	*p*-MeOC_6_H_4_	Ph	540
**6c**	*p*-Me_2_NC_6_H_4_	Ph	694^b^
**6d**	Ph	*p*-MeOC_6_H_4_	489
**6f**	*p*-MeOC_6_H_4_	*p*-MeOC_6_H_4_	526

^a^λ_exc_ = 380 nm; ^b^λ_exc_ = 480 nm.

Interestingly, the α-pyrone **6e** with the *N*,*N*-dimethylaminophenyl substituent in 4-position only fluoresces weakly in solution but shows a strong fluorescence in the solid state. This finding suggests that by restricting intramolecular motion and vibration, which enables radiation-less deactivation of the excited state [61], an AIE (aggregation‐induced emission) or AIEE (aggregation-induced enhanced emission), might become operative [62–64].

**Figure 15:**
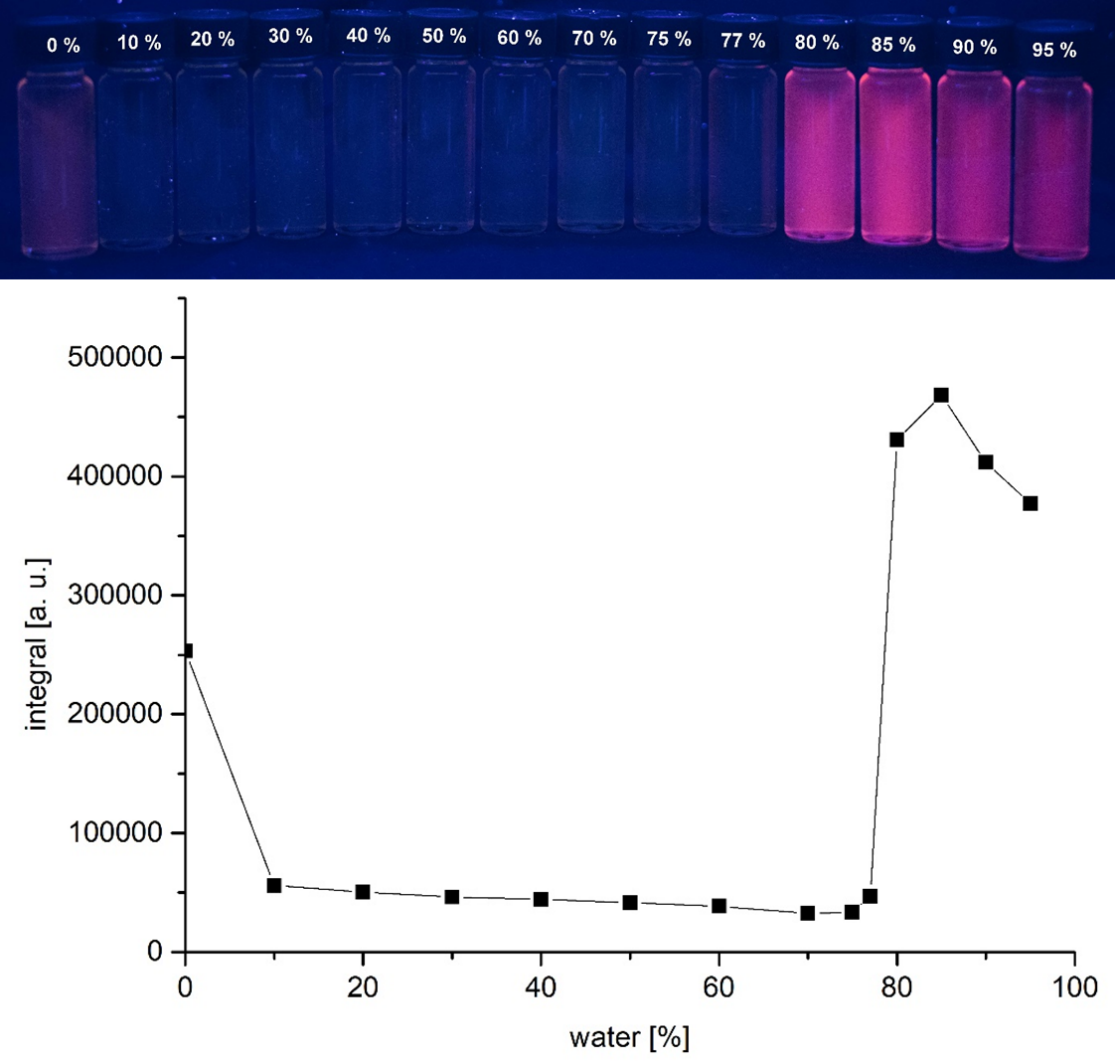
Fluorescence of compound **6e** in different THF/water fractions (top, λ_exc_ = 365 nm, handheld UV lamp) and *I*/*I*_0_ vs %H_2_O of α-pyrone **6e** in THF/water mixtures containing different water fractions (bottom, recorded at *T* = 298 K).

The AIE or AIEE effect was assessed by measuring the emission spectra of α-pyrone **6e** in THF/water at variable ratios (Figure 15). In pure THF α-pyrone **6e** displays an emission maximum at 644 nm with a relative intensity of 54. The addition of water first quenches the fluorescence and at a water/THF ratio of 80% aggregates are formed and the emission maximum is shifted to 632 nm. The maximal relative intensity of 130 is reached for a ratio of 85%, which is more intense than in pure THF, therefore, an AIEE effect occurs. Further increasing the water content slightly quenches the emission.

### Computational studies

#### Computational studies on 1*H*-pyridines **5** and **8**

For a further elucidation of the electronic structure the geometries of the electronical ground-state structures of the 1*H*-pyridines **5** and **8** were optimized using Gaussian 09 with the B3LYP functional [65–68] and the Pople 6-311G** basis set [69], applying vacuum calculations as well as the polarizable continuum model (PCM) with dichloromethane as a solvent [70] (for details of the DFT calculations, see Supporting Information File 1). The optimized geometries were verified by frequency analyses of the local minima. The electronic absorptions of the 1*H*-pyridines **5** and **8** were calculated on the level of TDDFT theory employing the B3LYP functional and the Pople 6-311G** basis set. The calculated absorption maxima are in accordance with the experimentally determined maxima (for details, see Tables S7 and S8 in Supporting Information File 1). Most characteristically, for all 1*H*-pyridines **5** and **8** the longest wavelength maxima representing the Franck–Condon S_1_ states are characterized by HOMO–LUMO transitions and S_2_ states are represented by HOMO–LUMO+1 transitions.

**Figure 16:**
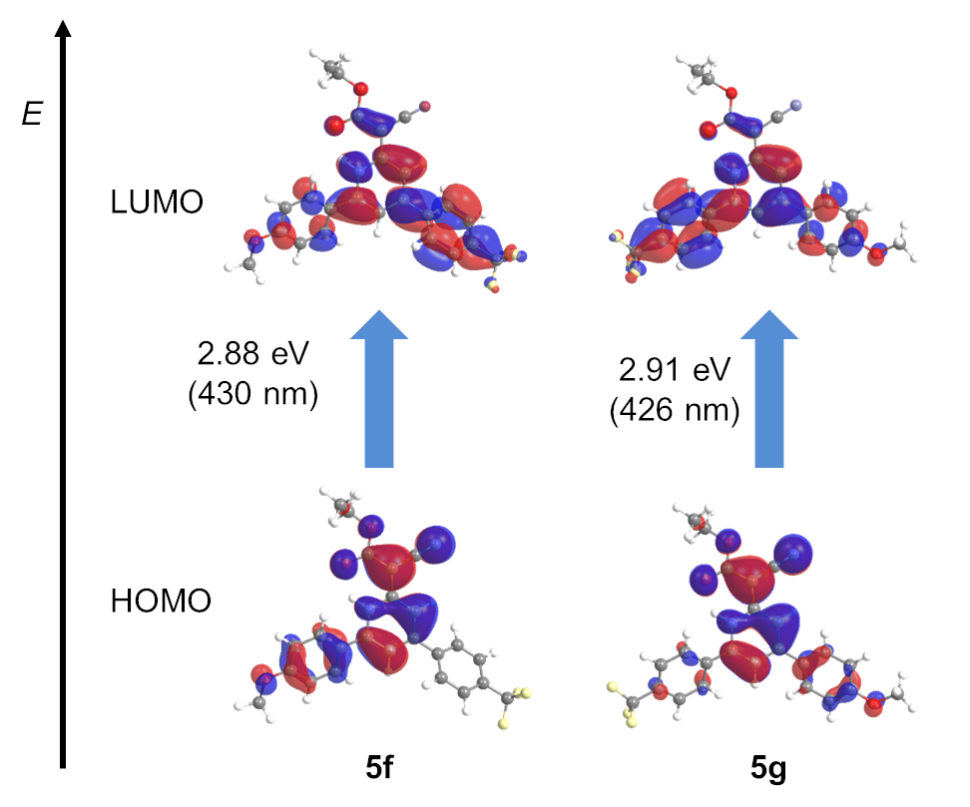
Selected DFT-computed (B3LYP 6-311G**) Kohn–Sham FMOs for 1*H*-pyridines **5f** and **5g** representing contributions of the longest wavelength Franck–Condon absorption bands.

The computed Kohn–Sham frontier molecular orbitals show that the coefficient density of the HOMO of the 1*H*-pyridines **5f** and **5g** with an electron-withdrawing and an electron-donating substituent is located on the 1*H*-pyridine core, the ester and cyano substituents and also on the electron-rich aryl substituent. For the LUMO, the coefficient density is spread over the whole scaffold (Figure 16).

#### Computational studies on α-pyrones **6**

**Figure 17:**
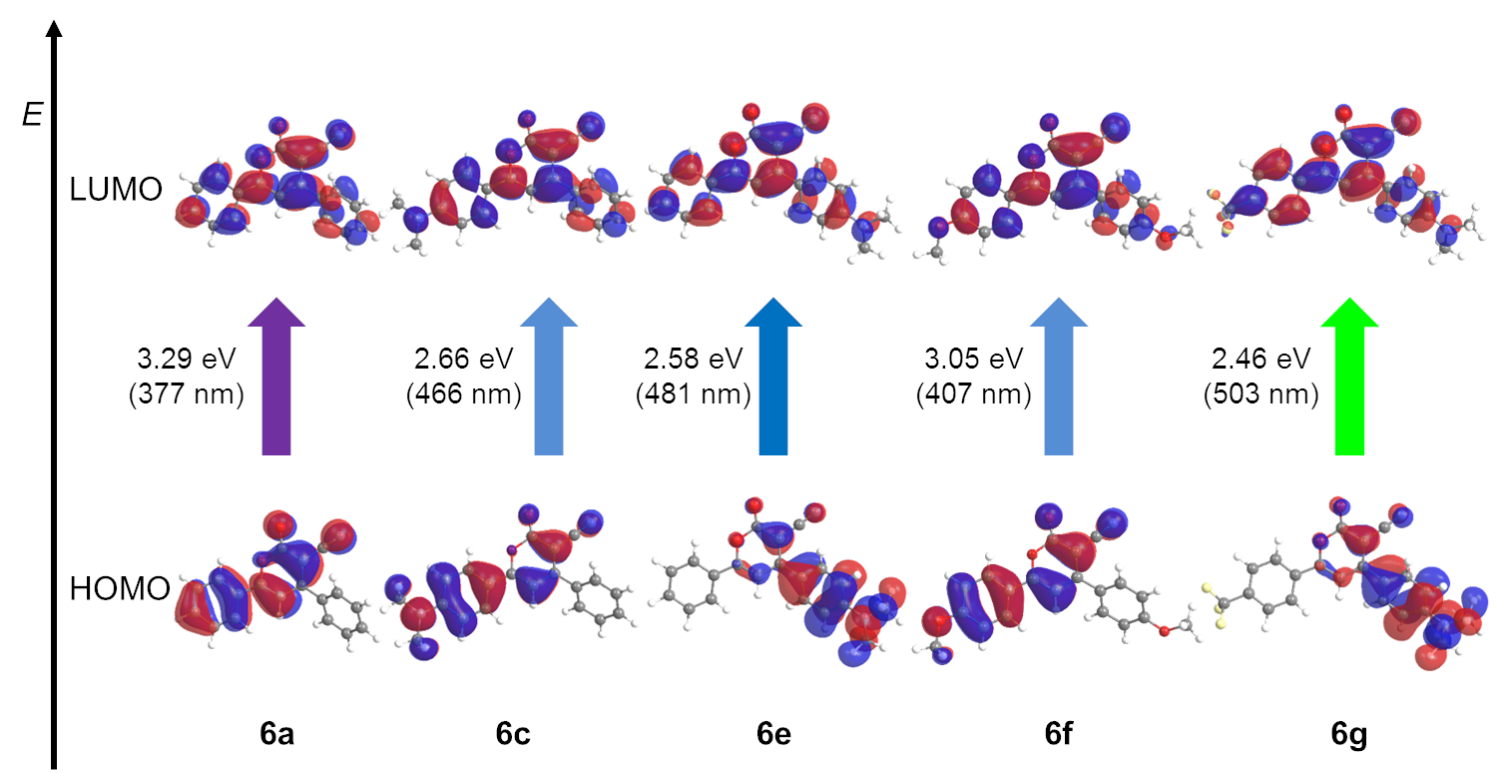
Selected DFT-computed (B3LYP 6-311G**) Kohn–Sham FMOs for 1*H*-pyridines **6a**, **6c**, **6e**, **6f**, and **6g** and representing contributions of the longest wavelength Franck-Condon absorption bands.

For further elucidation of the electronic structure the geometries of the electronical ground-state structures of the α-pyrones **6** were optimized using Gaussian 09 with the B3LYP functional [65–68] and the Pople 6-311G** basis set [69], applying vacuum calculations as well as the polarizable continuum model (PCM) with dichloromethane as a solvent [70] (for details on the DFT calculations, see Supporting Information File 1). The optimized geometries were verified by frequency analyses of the local minima. The electronic absorptions of the α-pyrones **6** were calculated on the level of TDDFT theory employing the B3LYP functional and the Pople 6-311G** basis set. The calculated absorption maxima are in accordance with the experimentally determined maxima (for details, see Table S10 in Supporting Information File 1). For all α-pyrones **6** the longest wavelength maxima are characterized by Franck–Condon S_1_ states representing HOMO–LUMO transitions (Figure 17).

The computed Kohn–Sham frontier molecular orbitals show that the coefficient density of the HOMO in the parent α-pyrone **6a** is localized on the α-pyrone core and on the phenyl substituent in the 6-position. For an *N*,*N*-dimethylaminophenyl substituent, there is no difference, but for an *N*,*N*-dimethylaminophenyl substituent in the 4-position the coefficient density is shifted towards this substituent. With electron-donating substituents in the 4- and 6-position, the coefficient density is again located on the core and the phenyl substituent in 6-position. Donor substituents in 4-position and acceptor substituents in 6-position cause a coefficient density shift towards the 4-substituent. The coefficient density in the LUMO in all compounds is spread over the whole scaffold.

## Conclusion

The cyclocondensation of alkynones and ethyl cyanoacetate, depending on the reaction conditions, the type of base, and the reaction temperature, as well as the electronic nature of the alkynone **3** furnishes either 1*H*-pyridines or α-pyrones. Optimized reaction conditions finally give rise to 8 examples of 1*H*-pyridines and 6 examples of α-pyrones. While the presence of electron-withdrawing substituents mainly furnish 1*H*-pyridines and electron-donating groups lead to the formation of α-pyrones. The strongly electron-donating *p*-*N*,*N*-dimethylaminophenyl group furnishes α-pyrones.

1*H*-Pyridines absorb and emit intensively in solution and in the solid state. While the absorption behavior is not affected by the substitution pattern the emission maxima are shifted bathochromically with increasing acceptor strength. The same trend manifests for the solid-state emission.

For α-pyrones the photophysical properties are considerably depending on the substituent pattern. The absorption and emission maxima are shifted bathochromically with increasing donor strength. α-Pyrones are only weakly fluorescent in solution. However, with distinct *p*-*N*,*N*-dimethylaminophenyl substitution in 6-position, an extraordinarily high fluorescence quantum yield of 99% in solution and 11% in the solid state was achieved. Interestingly, the isomeric *p*-*N*,*N*-dimethylaminophenyl substitution in 6-position represents a system with aggregation-induced emission enhancement. These design principles of luminescent 1*H*-pyridines and α-pyrones as polarity sensitive tunable luminophores and the observed aggregation-induced emission enhancement are currently under further investigation.

## Experimental

### Typical procedure for the cyclocondensation synthesis of compound **5d**

1-Phenyl-3-[4-(trifluoromethyl)phenyl]prop-2-yn-1-one (**3h**, 1.40 g, 5.00 mmol), was placed in a dry Schlenk tube and ethanol (10 mL) was added. Sodium carbonate (430 mg, 4.00 mmol), sodium acetate (250 mg, 3.00 mmol), water (5 mL), and ethyl cyanoacetate (**4**, 2.31 g, 20.0 mmol) were added and the mixture was stirred at 75 °C for 16 h. After the addition of CH_2_Cl_2_ (5.00 mL) and NaOH/FeSO_4_ solution (5.00 mL), the solution was extracted with CH_2_Cl_2_ (3 × 50.0 mL). The combined organic layers were dried (anhydrous MgSO_4_) and the solvent was removed in vacuo. The residue was purified by flash chromatography on silica gel (*n*-hexane/EtOAc 12:1 to 5:1 to 0:1) and washed with hot ethanol (5 mL) to give compound **5d** (513 mg, 25%) as orange solid. Mp 223–233 °C; ^1^H NMR (300 MHz, CDCl_3_) δ 1.38 (t, *J* = 7.1 Hz, 3H), 4.30 (q, *J* = 7.1 Hz, 2H), 7.12 (dd, *J* = 1.5, 1.6 Hz, 1H), 7.44 (dd, *J* = 1.5, 1.6 Hz, 1H), 7.56–7.63 (m, 3H), 7.78–7.84 (m, 6H), 14.57 (s, 1H); ^13^C NMR (150 MHz, CDCl_3_) δ 14.7 (CH_3_), 60.6 (CH_2_), 63.5 (C_quat_), 109.2 (CH), 116.0 (CH), 119.5 (C_quat_), 123.9 (q, *J*_C–F_ = 273 Hz, C_quat_), 126.1 (CH), 126.2–126.7 (m, CH), 127.8 (CH), 130.1 (CH), 131.6 (CH), 132.3 (C_quat_), 132.4 (q, *J*_C–F_ = 32.9 Hz, C_quat_), 140.6 (C_quat_), 146.6 (C_quat_), 151.2 (C_quat_), 156.0 (C_quat_), 171.0 (C_quat_); EIMS (70 eV, *m/z* (%)): 410 ([M]^+^, 24), 366 (24), 365 ([M − C_2_H_5_O]^+^, 100), 339 (12), 338 ([M − C_3_H_5_O_2_]^+^, 53), 337 (38), 308 (8), 240 (23), 149 ([M − C_16_H_12_F_3_]^+^, 11); IR (ATR) ν̃ [cm^−1^]: 3092 (w), 2992 (w), 2963 (w), 2943 (w), 2876 (w), 2806 (w), 2193 (m), 1625 (m), 1620 (m), 1597 (m), 1577 (m), 1506 (w), 1466 (w), 1413 (w), 1396 (w), 1369 (w), 1308 (m), 1300 (m), 1283 (s), 1258 (m), 1206 (w), 1165 (m), 1115 (s), 1092 (m), 1082 (m), 1071 (m), 1045 (s), 1030 (m), 1015 (m), 980 (m), 976 (m), 968 (w), 920 (w), 885 (w), 874 (w), 837 (s), 829 (m), 764 (s), 745 (w), 727 (w), 685 (m), 662 (w), 655 (w), 650 (w); UV–vis (CH_2_Cl_2_) λ_max_ [nm] (ε [L·mol^−1^·cm^−1^]): 274 (32000), 321 (18100), 428 (10000); emission (CH_2_Cl_2_) λ_max_ [nm] (Stokes shift [cm^−1^]): 565 (5700); quantum yield (CH_2_Cl_2_) Φ_f_: 0.01; Anal. calcd for C_23_H_17_F_3_N_2_O_2_ (410.1): C, 67.31; H, 4.18; N, 6.83; found: C, 67.50; H, 4.32; N, 6.70.

### Typical procedure for the cyclocondensation synthesis of compound **6c**

1-[4-(Dimethylamino)phenyl]-3-phenylprop-2-yn-1-one (**3c**, 249 mg, 1.00 mmol) was placed in a dry Schlenk tube and ethanol (2 mL) was added. Sodium carbonate (86.0 mg, 0.80 mmol), sodium acetate (50.0 mg, 0.60 mmol), water (1 mL), and ethyl cyanoacetate (**4**, 462 mg, 4.00 mmol) were added and the mixture was stirred at 75 °C for 16 h. After the addition of CH_2_Cl_2_ (5.00 mL) and NaOH/FeSO_4_ solution (5.00 mL), the solution was extracted with CH_2_Cl_2_ (3 × 50.0 mL). The combined organic layers were dried (anhydrous MgSO_4_) and the solvent was removed in vacuo. The residue was purified by flash chromatography on silica gel (*n*-hexane/EtOAc 5:1 to 1:1 to 0:1) and washed with hot ethanol (2.00 mL) and compound **6c** (37.0 mg, 12%) was obtained as deep purple solid. Mp 224–253 °C; ^1^H NMR (300 MHz, CDCl_3_) δ 3.10 (s, 6H), 6.69 (s, 1H), 6.69–6.75 (m, 2H), 7.51–7.58 (m, 3H), 7.67–7.73 (m, 2H), 7.78–7.85 (m, 2 H); ^13^C NMR (75 MHz, CDCl_3_) δ 40.2 (CH_3_), 91.1 (C_quat_), 100.1 (CH), 111.8 (CH), 115.7 (C_quat_), 116.5 (C_quat_), 128.0 (CH), 128.8 (CH), 129.3 (CH), 131.6 (CH), 135.3 (C_quat_), 153.4 (C_quat_), 160.3 (C_quat_), 164.1 (C_quat_), 164.9 (C_quat_). EIMS (70 eV, *m/z* (%)): 317 (15), 316 ([M]^+^, 66), 293 (11), 289 ([M − CN]^+^, 11), 288 ([M − CO]^+^, 51), 287 (19), 167 ([M − C_9_H_11_NO]^+^, 18), 150 (11), 149 ([M − C_11_H_5_NO]^+^, 100), 148 (20), 144 (13), 127 ([M − C_11_H_11_NO_2_]^+^, 12), 85 (13), 71 (22), 57 (18), 43 ([M − C_18_H_11_NO_2_]^+^, 13); IR (ATR) ν̃ [cm^−1^]: 3092 (w), 3048 (w), 2901 (w), 2864 (w), 2812 (w), 2739 (w), 2212 (w), 1708 (m), 1706 (m), 1609 (m), 1589 (m), 1570 (m), 1530 (m), 1497 (m), 1491 (m), 1482 (m), 1478 (m), 1473 (m), 1467 (m), 1458 (m), 1433 (m), 1375 (m), 1360 (m), 1333 (m), 1252 (m), 1209 (m), 1171 (m), 1159 (m), 1125 (m), 1111 (m), 1082 (m), 1059 (m), 1020 (m), 995 (m), 953 (m), 945 (m), 924 (w), 853 (m), 818 (s), 795 (m), 750 (m), 748 (m), 692 (s), 669 (m), 640 (m); UV–vis (CH_2_Cl_2_) λ_max_ [nm] (ε [L·mol^−1^·cm^−1^]): 294 (18800), 482 (47300); emission (CH_2_Cl_2_) λ_max_ [nm] (Stokes-shift [cm^−1^]): 567 (3100); quantum yield (CH_2_Cl_2_) Φ_f_: 0.99; emission (solid) λ_max_ [nm]: 694; quantum yield (solid) Φ_f_: 0.11; Anal. calcd for C_20_H_16_N_2_O_2_ (316.1): C, 75.93; H, 5.10; N, 8.86; found: C, 75.74; H, 5.19; N, 8.56.

### Typical procedure for the cyclocondensation synthesis of compound **8a**

1,3-Diphenylprop-2-yn-1-one (**3a**, 103 mg, 0.50 mmol) was placed in a dry Schlenk tube and ethanol (1.00 mL) was added. Sodium carbonate (43.0 mg, 0.40 mmol), sodium acetate (25.0 mg, 0.30 mmol), water (50.0 µL) and diethyl (*Z*)-3-amino-2-cyanopent-2-endioate (**7**, 226 mg, 1.00 mmol) were added and the mixture was stirred at 75 °C for 16 h. After the addition of CH_2_Cl_2_ (5.00 mL) and NaOH/FeSO_4_ solution (5.00 mL), the solution was extracted with CH_2_Cl_2_ (3 × 50.0 mL). The combined organic layers were dried (anhydrous MgSO_4_) and the solvent was removed in vacuo. The residue was purified by flash chromatography on silica gel (*n*-hexane/EtOAc 5:1 to 1:1 to 0:1) and washed with hot ethanol (5.00 mL) to furnish compound **8a** (108 mg, 52%) as yellow solid. Mp 140–146 °C; ^1^H NMR (300 MHz, CDCl_3_) δ 1.08 (t, *J* = 7.2 Hz, 3H), 1.37 (t, *J* = 7.1 Hz, 3H), 4.22 (q, *J* = 7.2 Hz, 2H), 4.31 (q, *J* = 7.1 Hz, 2H), 6.94 (d, *J* = 1.9 Hz, 1H), 7.40–7.48 (m, 5H), 7.55–7.62 (m, 3H), 7.75–7.84 (m, 2H), 15.60 (s, 1H); ^13^C NMR (75 MHz, CDCl_3_) δ 13.7 (CH_3_), 14.7 (CH_3_), 60.9 (CH_2_), 62.2 (CH_2_), 62.7 (C_quat_), 112.2 (CH), 118.2 (C_quat_), 122.8 (C_quat_), 126.3 (CH), 127.9 (CH), 128.8 (CH), 129.6 (CH), 130.1 (CH), 131.77 (CH), 131.84 (C_quat_), 137.4 (C_quat_), 146.1 (C_quat_), 151.9 (C_quat_), 153.0 (C_quat_), 165.1 (C_quat_), 172.1 (C_quat_); EIMS (70 eV, *m/z* (%)): 415 ([M + H]^+^, 26), 414 ([M]^+^, 97), 369 ([M − C_2_H_5_O]^+^, 18), 342 (28), 341 ([M − C_3_H_5_O]^+^, 27), 340 ([M − C_3_H_6_O]^+^, 12), 314 (20), 313 ([M − C_8_H_5_]^+^, 57), 298 (24), 297 ([M − C_5_H_9_O_3_]^+^, 27), 296 ([M − C_5_H_10_O_3_]^+^, 33), 287 (10), 286 (25), 271 (24), 270 ([M − C_6_H_8_O_4_]^+^, 100), 269 (23), 268 ([M − C_6_H_10_O_4_]^+^, 21), 266 (12), 258 ([M − C_7_H_10_NO_3_]^+^, 14), 245 (18), 241 (13), 240 (26), 231 (15), 230 ([M − C_8_H_10_NO_4_]^+^, 34), 203 (19), 202 (31), 164 (13); IR (ATR) ν̃ [cm^−1^]: 2978 (w), 2895 (w), 2197 (m), 1722 (m), 1636 (m), 1593 (s), 1578 (m), 1501 (m), 1489 (w), 1462 (w), 1441 (w), 1420 (w), 1364 (w), 1308 (m), 1288 (m), 1248 (s), 1188 (w), 1169 (m), 1134 (m), 1113 (s), 1092 (m), 1067 (m), 1047 (m), 1028 (w), 1001 (w), 885 (m), 854 (m), 847 (w), 775 (w), 758 (s), 746 (m), 694 (m), 658 (w); UV–vis (CH_2_Cl_2_) λ_max_ [nm] (ε [L·mol^−1^·cm^−1^]): 274 (20300), 324 (20100), 417 (7700); emission (CH_2_Cl_2_) λ_max_ [nm] (Stokes shift [cm^−1^]): 557 (6000); quantum yield (CH_2_Cl_2_) Φ_f_ = < 0.01; Anal. calcd for C_25_H_22_N_2_O_4_ (414.2): C, 72.45; H, 5.35; N, 6.76; found: C, 71.97; H, 5.45; N, 6.52.

## Supporting Information

For experimental details of the synthesis and analytical data of compounds **3**, **5**, **6**, and **8**, ^1^H and ^13^C NMR, and absorption and emission spectra of compounds **5**, **6**, and **8**, solid state emission spectra of compounds **5** and **6**, X-ray structural data of compound **5a**, and DFT/TDDFT calculations of compounds **5** and **6**, see below.

File 1 Additional experimental and calculated data.

## Statement

The reported results have been summarized in the inaugural dissertation "Diversitätsorientierte Synthese von Heterocyclen über Cyclokondensationen von Alkinonen und CH-aciden Estern" by Dr. Natascha Breuer, Heinrich Heine University Düsseldorf, 2018.
