# Supplementary material for: Emission solvatochromic, solid-state and aggregation-induced emissive α-pyrones and emission-tuneable 1H-pyridines by Michael addition–cyclocondensation sequences
Source: Beilstein J Org Chem. 2019 Nov 12;15:2684–703. doi: 10.3762/bjoc.15.262 (PMC6880829; doi:10.3762/bjoc.15.262)
Supplement: File 1 — Additional experimental and calculated data. [file Beilstein_J_Org_Chem-15-2684-s001.pdf]

BEILSTEIN JOURNAL OF ORGANIC CHEMISTRY

Supporting Information

for

Emission solvatochromic, solid-state and aggregation-induced emissive α-pyrones and emission-tuneable 1*H*-pyridines by Michael addition–cyclocondensation sequences

Natascha Breuer, Irina Gruber, Christoph Janiak and Thomas J. J. Müller

Beilstein J. Org. Chem. **2019,** *15,* 2684–2703. doi:10.3762/bjoc.15.262

Additional experimental and calculated data

License and Terms: This is a supporting information file under the terms of the Creative Commons Attribution License (http://creativecommons.org/licenses/by/4.0). Please note that the reuse, redistribution and reproduction in particular requires that the authors and source are credited.
The license is subject to the *Beilstein Journal of Organic Chemistry* terms and conditions: (https://www.beilstein-journals.org/bjoc)

Table of Contents

1 General considerations S5

2 Synthesis and analytical data of compounds **3**, **5**, **6**, and **8** S6

2.1 Sonogashira synthesis of alkynones **3** S6

2.2 Kumada–Sonogashira synthesis of alkynones **3** S10

2.3 One-pot synthesis of 3-ethyl (*Z*)-2-cyano-2-(4,6-diphenyl-1*H*-pyridin-2-ylidene)acetate (**5a**) S14

2.4 Synthesis of 2-oxo-4,6-diphenyl-2*H*-pyran-3-carbonitrile (**6a**) S15

2.5 General procedure for the synthesis of 1*H*-pyridines **5** and α-pyrones **6** (GP3) S16

2.5.1 Ethyl (*Z*)-2-cyano-2-(4,6-diphenyl-1*H*-pyridin-2-ylidene)acetate (**5a**) S16

2.5.2 Ethyl (*Z*)-2-cyano-2-{4-phenyl-6-[4-(trifluoromethyl)phenyl]-1*H*-pyridin-2-ylidene}-acetate (**5b**) S16

2.5.3 Ethyl (*Z*)-2-cyano-2-[6-(4-cyanophenyl)-4-phenyl-1*H*-pyridin-2-ylidene]acetate (**5c**) S17

2.5.4 Ethyl (*Z*)-2-cyano-2-(6-phenyl-4-(4-(trifluoromethyl)phenyl)pyridin-2(1*H*)-ylidene)acetate (**5d**) S17

2.5.5 Ethyl (*Z*)-2-cyano-2-(4-(4-cyanophenyl)-6-phenylpyridin-2(1*H*)-ylidene)acetate (**5e**) S18

2.5.6 Ethyl (*Z*)-2-cyano-2-[6-(4-methoxyphenyl]-4-[4-(trifluoromethyl)phenyl]pyridin-2(1*H*)-ylidene)acetate (**5f**) S19

2.5.7 Ethyl (*Z*)-2-cyano-2-{4-(4-methoxyphenyl)-6-[4-(trifluoromethyl)phenyl]pyridin-2(1*H*)-ylidene}acetate (**5g**) S19

2.5.8 Ethyl (*Z*)-2-cyano-2-[4-phenyl-6-(thiophen-2-yl)pyridin-2(1*H*)-ylidene]acetate (**5h**) S20

2.5.9 6-(4-Methoxyphenyl)-2-oxo-4-phenyl-2*H*-pyran-3-carbonitrile (**6b**) S21

2.5.10 6-[4-(Dimethylamino)phenyl]-2-oxo-4-phenyl-2*H*-pyran-3-carbonitrile (**6c**) S21

2.5.11 4-(4-Methoxyphenyl)-2-oxo-6-phenyl-2*H*-pyran-3-carbonitrile (**6d**) S22

2.5.12 4-[4-(Dimethylamino)phenyl]-2-oxo-6-phenyl-2*H*-pyran-3-carbonitrile (**6e**) S22

2.5.13 4,6-Bis(4-methoxyphenyl)-2-oxo-2*H*-pyran-3-carbonitrile (**6f**) S23

2.5.14 4-[4-(Dimethylamino)phenyl]-2-oxo-6-[4-(trifluormethyl)pheny]-2*H*-pyran-3-carbonitrile (**6g**) S23

2.6 General procedure for the synthesis of 1*H*-pyridines **8** (GP4) S24

2.6.1 Ethyl (*Z*)-2-(1-cyano-2-ethoxy-2-oxoethylidene)-4,6-diphenyl-1,2-dihydropyridine-3-carboxylate (**8a**) S24

2.6.2 Ethyl (*Z*)-2-(1-cyano-2-ethoxy-2-oxoethylidene)-6-(4-methoxyphenyl)-4-phenyl-1,2-dihydropyridine-3-carboxylate (**8b**) S25

3 ^{1}H and ^{13}C NMR spectra of 1*H*-pyridines **5** S26

3.1 Ethyl (*Z*)-2-cyano-2-(4,6-diphenyl-1*H*-pyridin-2-ylidene)acetate (**5a**) S26

3.2 Ethyl (Z)-2-cyano-2-{4-phenyl-6-[4-(trifluoromethyl)phenyl]-1H-pyridin-2-ylidene}acetate (**5b**) S27

3.3 Ethyl (Z)-2-Cyano-2-[6-(4-cyanophenyl)-4-phenyl-1H-pyridin-2-ylidene]acetate (**5c**) S28

3.4 Ethyl (*Z*)-2-Cyano-2-{4-phenyl-6-[4-(trifluoromethyl)phenyl]-1*H*-pyridin-2-ylid-ene}acetate (**5d**) ... S29
3.5 Ethyl (*Z*)-2-cyano-2-[6-(4-cyanophenyl)-4-phenyl-1*H*-pyridin-2-ylidene]acetate (**5e**) ... S30
3.6 Ethyl (Z)-2-cyano-2-[4-(4-methoxyphenyl]-6-[4-(trifluoromethyl)phenyl]pyridin-2(1*H*)-ylidene)acetate (**5f**) ... S31
3.7 Ethyl (*Z*)-2-cyano-2-{4-(4-methoxyphenyl)-6-[4-(trifluoromethyl)phenyl]pyridin-2(1*H*)-ylidene}acetate (**5g**) ... S32
3.8 Ethyl (*Z*)-2-cyano-2-[4-phenyl-6-(thiophen-2-yl)pyridin-2(1*H*)-ylidene]acetate (**5h**) ... S33
4 ^{1}H and ^{13}C NMR spectra of α-pyrones **6** ... S34
4.1 2-Oxo-4,6-diphenyl-2*H*-pyran-3-carbonitrile (**6a**) ... S34
4.2 6-(4-Methoxyphenyl)-2-oxo-4-phenyl-2*H*-pyran-3-carbonitrile (**6b**) ... S35
4.3 6-[4-(Dimethylamino)phenyl]-2-oxo-4-phenyl-2*H*-pyran-3-carbonitrile (**6c**) ... S36
4.4 4-(4-Methoxyphenyl)-2-oxo-6-phenyl-2*H*-pyran-3-carbonitrile (**6d**) ... S37
4.5 4-[4-(Dimethylamino)phenyl]-2-oxo-6-phenyl-2*H*-pyran-3-carbonitrile (**6e**) ... S38
4.6 4,6-Bis(4-methoxyphenyl)-2-oxo-2*H*-pyran-3-carbonitrile (**6f**) ... S39
4.7 4-[4-(Dimethylamino)phenyl]-2-oxo-6-[4-(trifluormethyl)pheny]-2*H*-pyran-3-carbo-nitrile (**6g**) ... S40
5 ^{1}H and ^{13}C NMR spectra of 1*H*-pyridines **8** ... S41
5.1 Ethyl (*Z*)-2-(1-cyano-2-ethoxy-2-oxoethylidene)-4,6-diphenyl-1,2-dihydropyridin-3-carboxylate (**8a**) ... S41
5.2 Ethyl (*Z*)-2-(1-cyano-2-ethoxy-2-oxoethyliden)-6-(4-methoxyphenyl)-4-phenyl-1,2-dihydropyridine-3-carboxylate (**8b**) ... S42
6 Absorption and emission spectra of 1*H*-pyridine **5** ... S43
6.1 Ethyl (*Z*)-2-cyano-2-(4,6-diphenyl-1*H*-pyridin-2-ylidene)acetate (**5a**) ... S43
6.2 Ethyl (*Z*)-2-cyano-2-{4-phenyl-6-[4-(trifluoromethyl)phenyl]-1*H*-pyridin-2-ylid-ene}acetate (**5b**) ... S43
6.3 Ethyl (*Z*)-2-cyano-2-[6-(4-cyanophenyl)-4-phenyl-1*H*-pyridin-2-ylidene]acetate (**5c**) ... S44
6.4 Ethyl (*Z*)-2-cyano-2-{4-phenyl-6-[4-(trifluoromethyl)phenyl]-1*H*-pyridin-2-ylid-ene}acetate (**5d**) ... S44
6.5 Ethyl (*Z*)-2-cyano-2-[6-(4-cyanophenyl)-4-phenyl-1*H*-pyridin-2-ylidene]acetate (**5e**) ... S45
6.6 Ethyl (*Z*)-2-cyano-2-[4-(4-methoxyphenyl]-6-[4-(trifluoromethyl)phenyl]pyridin-2(1*H*)-ylidene)acetate (**5f**) ... S45
6.7 Ethyl (*Z*)-2-cyano-2-{4-(4-methoxyphenyl)-6-[4-(trifluoromethyl)phenyl]pyridin-2(1*H*)-ylidene}acetate (**5g**) ... S46
6.8 Ethyl (*Z*)-2-cyano-2-[4-phenyl-6-(thiophen-2-yl)pyridin-2(1*H*)-ylidene]acetate (**5h**) ... S46
7 Absorption and emission spectra of α-pyrones **6** ... S47
7.1 2-Oxo-4,6-diphenyl-2*H*-pyran-3-carbonitrile (**6a**) ... S47
7.2 6-(4-Methoxyphenyl)-2-oxo-4-phenyl-2*H*-pyran-3-carbonitrile (**6b**) ... S47
7.3 6-[4-(Dimethylamino)phenyl]-2-oxo-4-phenyl-2*H*-pyran-3-carbonitrile (**6c**) ... S48
7.4 4-(4-Methoxyphenyl)-2-oxo-6-phenyl-2*H*-pyran-3-carbonitrile (**6d**) ... S48
7.5 4-[4-(Dimethylamino)phenyl]-2-oxo-6-phenyl-2*H*-pyran-3-carbonitrile (**6e**) ... S49
7.6 4,6-Bis(4-methoxyphenyl)-2-oxo-2*H*-pyran-3-carbonitrile (**6f**) ... S49

7.7 4-[4-(Dimethylamino)phenyl]-2-oxo-6-[4-(trifluormethyl)pheny]-2*H*-pyran-3-carbonitrile (**6g**) S50

7.8 Solvatochromism of 6-[4-(dimethylamino)phenyl]-2-oxo-4-phenyl-2*H*-pyran-3-carbonitrile (**6c**) S50

7.9 Aggregation-induced enhanced emission of 4-[4-(dimethylamino)phenyl]-2-oxo-6-phenyl-2*H*-pyran-3-carbonitrile (**6e**) S51

8 Absorption and emission spectra of 1*H*-pyridine **8** S52

8.1 Ethyl (*Z*)-2-(1-cyano-2-ethoxy-2-oxoethylidene)-4,6-diphenyl-1,2-dihydropyridine-3-carboxylate (**8a**) S52

8.2 Ethyl (*Z*)-2-(1-cyano-2-ethoxy-2-oxoethylidene)-6-(4-methoxyphenyl)-4-phenyl-1,2-dihydropyridine-3-carboxylate (**8b**) S52

9 Emission spectra of 1*H*-pyridines **5** in the solid state S53

9.1 Ethyl (*Z*)-2-cyano-2-(4,6-diphenyl-1*H*-pyridin-2-ylidene)acetate (**5a**) S53

9.2 Ethyl (*Z*)-2-cyano-2-{4-phenyl-6-[4-(trifluoromethyl)phenyl]-1*H*-pyridin-2-ylidene}acetate (**5b**) S53

10 Emission spectra of α-pyrones **6** in the solid state S54

10.1 2-Oxo-4,6-diphenyl-2*H*-pyran-3-carbonitrile (**6a**) S54

10.2 6-(4-Methoxyphenyl)-2-oxo-4-phenyl-2*H*-pyran-3-carbonitrile (**6b**) S54

10.3 6-[4-(Dimethylamino)phenyl]-2-oxo-4-phenyl-2*H*-pyran-3-carbonitrile (**6c**) S55

10.4 4-(4-Methoxyphenyl)-2-oxo-6-phenyl-2*H*-pyran-3-carbonitrile (**6d**) S55

10.5 4,6-Bis(4-methoxyphenyl)-2-oxo-2*H*-pyran-3-carbonitrile (**6f**) S56

11 X-ray structural data of compound **5a** S57

12 Quantum chemical calculations S62

12.1 Computed xyz-coordinates of 1*H*-pyridines **5** S62

12.1.1 Ethyl (*Z*)-2-cyano-2-(4,6-diphenyl-1*H*-pyridin-2-ylidene)acetate (**5a**) (B3LYP) S64

12.1.2 Ethyl (*Z*)-2-cyano-2-{4-phenyl-6-[4-(trifluoromethyl)phenyl]-1*H*-pyridin-2-ylidene}acetate (**5b**) (B3LYP) S65

12.1.3 Ethyl (*Z*)-2-cyano-2-[6-(4-cyanophenyl)-4-phenyl-1*H*-pyridin-2-ylidene]acetate (**5c**) (B3LYP) S66

12.1.4 Ethyl (*Z*)-2-cyano-2-{4-phenyl-6-[4-(trifluoromethyl)phenyl]-1*H*-pyridin-2-ylidene}acetate (**5d**) (B3LYP) S67

12.1.5 Ethyl (*Z*)-2-cyano-2-[6-(4-cyanophenyl)-4-phenyl-1*H*-pyridin-2-ylidene]acetate (**5e**) (B3LYP) S68

12.1.6 Ethyl (*Z*)-2-cyano-2-[4-(4-methoxyphenyl]-6-[4-(trifluoromethyl)phenyl]pyridin-2(1*H*)-ylidene)acetate (**5f**) (B3LYP) S69

12.1.7 Ethyl (*Z*)-2-cyano-2-{4-(4-methoxyphenyl)-6-[4-(trifluoromethyl)phenyl]pyridin-2(1*H*)-ylidene}acetate (**5g**) (B3LYP) S70

12.2 Computed xyz-coordinates of 1*H*-pyridines **8** S72

12.2.1 Ethyl (*Z*)-2-(1-cyano-2-ethoxy-2-oxoethylidene)-4,6-diphenyl-1,2-dihydropyridine-3-carboxylate (**8a**) (B3LYP) S72

12.2.2 Ethyl (*Z*)-2-(1-cyano-2-ethoxy-2-oxoethylidene)-6-(4-methoxyphenyl)-4-phenyl-1,2-dihydropyridine-3-carboxylate (**8b**) (B3LYP) S73

12.3 Calculated equilibrium ground-state structures of 1*H*-pyridines **5a** and **8a** S75

12.4 Computed xyz-coordinates of α-pyrones **6** S76
12.4.1 2-Oxo-4,6-diphenyl-2*H*-pyran-3-carbonitrile (**6a**) (B3LYP) S78
12.4.2 6-(4-Methoxyphenyl)-2-oxo-4-phenyl-2*H*-pyran-3-carbonitrile (**6b**) (B3LYP) S78
12.4.3 6-[4-(Dimethylamino)phenyl]-2-oxo-4-phenyl-2*H*-pyran-3-carbonitrile (**6c**) (B3LYP) .. S79
12.4.4 4-(4-Methoxyphenyl)-2-oxo-6-phenyl-2*H*-pyran-3-carbonitrile (**6d**) (B3LYP) S80
12.4.5 4-[4-(Dimethylamino)phenyl]-2-oxo-6-phenyl-2*H*-pyran-3-carbonitrile (**6e**) (B3LYP) .. S81
12.4.6 4,6-Bis(4-methoxyphenyl)-2-oxo-2*H*-pyran-3-carbonitrile (**6f**) (B3LYP) S82
12.4.7 4-[4-(Dimethylamino)phenyl]-2-oxo-6-[4-(trifluormethyl)pheny]-2*H*-pyran-3-carbonitrile (**6g**) (B3LYP) S83
12.5 Calculated equilibrium ground-state structures of α-pyrones **6** S84
13 References S85

1 General considerations

All reactions were performed in dried Schlenk tubes. Thin layer chromatography was used to monitor the reaction progress qualitatively, using silica-gel-layered aluminum foil (60, F_{254} Merck, Darmstadt). For detection, UV light of wavelengths 254 and 366 nm was employed. Commercially available chemicals were used as received without any further purification. All commercially available chemicals were purchased from ABCR GmbH & Co. KG (Karlsruhe, Deutschland), Acros Organics (Geel, Belgien), Alfa-Aesar GmbH & Co. KG (Karlsruhe, Deutschland), Carl Roth GmbH & Co. KG (Karlsruhe, Deutschland), J&K Scientific GmbH (Beijing, China), Merck KGaA (Darmstadt, Deutschland), Riedel-de Haën (Seelze, Deutschland), Sigma-Aldrich Co. LLC. (St. Louis, USA) und Tokyo Chemical Industry Co., LTD. (Tokio, Japan) ^{1}H, ^{13}C and DEPT NMR spectra were recorded in $CDCl_3$ and CD_2Cl_2 on a 300 MHz (Bruker Avance III - 300) or 600 MHz (Bruker Avance III - 600) NMR spectrometer. Chemical shifts are referenced to the internal solvent signal: $CDCl_3$ (^{1}H δ 7.26, ^{13}C δ 77.2) and CD_2Cl_2 (^{1}H δ 5.32, ^{13}C δ 54.0). Multiplicities are stated as s (singlet), d (doublet), dd (doublet of doublet), t (triplet), q (quatet), m (multiplet). Coupling constants (*J*) are given in hertz. The assignment of primary (CH_3), secondary (CH_2), tertiary (CH), and quaternary carbon nuclei (C_{quat}) was made using DEPT-135 spectra. Mass spectroscopic measurements were conducted on a quadrupole (EI) analyzer (TSQ 7000, Finnigan MAT) in the Department of Mass Spectrometry of the Institute of Inorganic and Structural Chemistry, Heinrich-Heine-Universität Düsseldorf. IR spectra were measured using ATR technique (Shimadzu IR Affinity-1). The intensities of the IR bands are abbreviated as w (weak), m (medium), s (strong) and vs (very strong). Elemental analyses were carried out on a Perkin Elmer Series II Analyser 2400 in the microanalytical laboratory of the Pharmazeutisches Institut of the Heinrich-Heine-Universität Düsseldorf. Uncorrected melting points and decomposition temperature were determined with a Reichert Thermovar melting point microscope (heating unit: PeakTech 6000A DC Power Supply; thermometry: Norma D2400 (digital)). Absorption spectra were recorded in various spectroscopy grade solvents at 293 K using a PerkinElmer UV–vis–NIR Lambda 19 spectrometer. Emission spectra in solution were recorded at room temperature on a LS55 spectrometer (Perkin Elmer). Fluorescence quantum yields Φ_f were determined relative to a fluorescence standard (measurements at five concentrations, Coumarin 153 in EtOH, λ_{exc} = 420 nm, Φ_f = 0.45 and DCM in EtOH λ_{exc} = 465 nm, Φ_f = 0.435) [1]. The areas under the emission bands were extracted from the spectra using the program OriginPro 9.0G. Quantum chemical calculations were carried out utilizing the HPC-Cluster Ivybridge of the Zentrum für Informations- und Medientechnologie (ZIM) at the Heinrich-Heine-University Düsseldorf.

2 Synthesis and analytical data of compounds 3, 5, 6, and 8

2.1 Sonogashira synthesis of alkynones 3

General procedure 1 (GP1) according to literature [2]

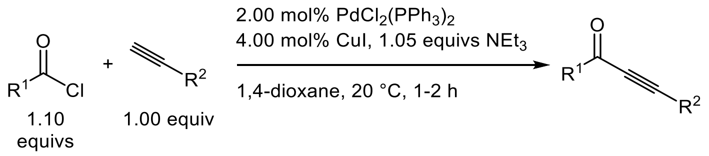

In a Schlenk tube with magnetic stirring bar were placed $PdCl_2(PPh_3)_2$ (2.00 mol %) and CuI (4.00 mol %) and evacuated and flushed with a nitrogen atmosphere three times (for experimental details, see Table S1). Then, 1,4-dioxane (1.00 M), the acid chloride (1.10 equiv), the terminal alkyne (1.00 equiv), and triethylamine (1.05 equiv) were successively added and the mixture stirred at room temp for 2 h (monitored by TLC). The crude product was adsorbed on celite® and purified by flash chromatography on silica gel (*n*-hexane/EtOAc) to give the analytically pure alkynones **3**.

Table S1. Experimental details for the synthesis of alkynones **3** starting from acid chlorides and alkynes.

entry	acid chloride	terminal alkyne	$PdCl_2(PPh_3)_2$ / CuI	triethyl-amine	alkynone **3**
1	1.26 g (8.87 mmol) of benzoyl chloride	830 mg (8.04 mmol) of phenylacetylene	112 mg (160 µmol) / 61.0 mg (320 µmol)	848 mg (8.40 mmol)	1.53 g (93%) of **3a**
2	569 mg (3.30 mmol) of 4-methoxybenzoyl chloride	310 mg (3.00 mmol) of phenylacetylene	42.0 mg (60.0 µmol) / 22.8 mg (120 µmol)	318 mg (3.15 mmol)	655 mg (92%) of **3b**
3	418 mg (2.21 mmol) of 4-dimethylamino-benzoyl chloride	207 mg (2.01 mmol) of phenylacetylene	28.0 mg (40.0 µmol) / 15.2 mg (80.0 µmol)	212 mg (2.10 mmol)	116 mg (23%) of **3c**
4	470 mg (2.21 mmol) of 4-(trifluoromethyl)-benzoyl chloride	208 mg (2.02 mmol) of phenylacetylene	28.0 mg (40.0 µmol) / 15.2 mg (80.0 µmol)	212 mg (2.10 mmol)	490 mg (89%) of **3d**
5	372 mg (2.20 mmol) of 4-cyanobenzoyl chloride	209 mg (2.03 mmol) of phenylacetylene	28.0 mg (40.0 µmol) / 15.2 mg (80.0 µmol	212 mg (2.10 mmol)	297 mg (64%) of **3e**
6	470 mg (3.31 mmol) of benzoyl chloride	393 mg (3.00 mmol) of 4-ethynylbenzonitrile	42.0 mg (60.0 µmol) / 22.8 mg (120 µmol)	318 mg (3.15 mmol)	547 mg (79%) of **3g**
7	331 mg (2.21 mmol) of 2-thienylcarbonylchloride	208 mg (2.02 mmol) of phenylacetylene	28.0 mg (40.0 µmol) / 15.2 mg (80.0 µmol)/	212 mg (2.10 mmol)	338 mg (80%) of **3n**

1,3-Diphenylprop-2-yn-1-one (3a) [2]

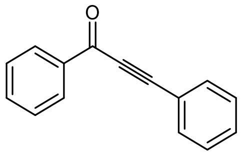

$C_{15}H_{10}O$
206.24 g/mol

According to GP1 and column chromatography on silica gel (*n*-hexane/EtOAc 40:1) 1.53 g (93%) of compound **3a** was obtained as an orange oil, R_f (*n*-hexane/EtOAc 5:1) = 0.47.

^{1}H NMR (300 MHz, $CDCl_3$): δ 7.35-7.56 (m, 5 H), 7.58-7.75 (m, 3 H), 8.18-8.27 (m, 2 H). ^{13}C NMR (75 MHz, $CDCl_3$): δ 87.0 (C_{quat}), 93.2 (C_{quat}), 120.3 (C_{quat}), 128.75 (CH), 128.81 (CH), 129.7 (CH), 130.9 (CH), 133.2 (CH), 134.2 (CH), 137.0 (C_{quat}), 178.1 (C_{quat}). EI-MS (70 eV, *m/z* (%)): 206 ($[M]^+$, 48), 178 ($[M-CO]^+$, 100), 129 ($[M-C_6H_5]^+$, 73), 105 ($[M-C_8H_5]^+$, 4), 101 ($[M-C_7H_5O]^+$, 9), 89 ($[M-C_8H_6O]^+$, 6), 77 ($[M-C_9H_5O]^+$, 10).

1-(4-Methoxyphenyl)-3-phenylprop-2-yn-1-one (3b) [2]

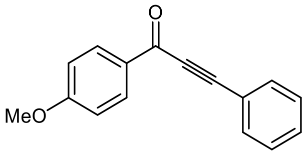

$C_{16}H_{12}O_2$
236.27 g/mol

According to GP1 and column chromatography on silica gel (*n*-hexane/EtOAc 20:1 to 10:1) 655 mg (92%) of compound **3b** was obtained as a beige solid, Mp 225-230 °C, R_f (*n*-hexane/EtOAc 20:1) = 0.12.

^{1}H NMR (300 MHz, $CDCl_3$): δ 3.90 (s, 3 H), 6.94-7.02 (m, 2 H), 7.36-7.52 (m, 3 H), 7.62-7.71 (m, 2 H), 8.16-8.24 (m, 2 H). ^{13}C NMR (75 MHz, $CDCl_3$): δ 55.7 (CH_3), 87.1 (C_{quat}), 92.4 (C_{quat}), 114.0 (CH), 120.5 (C_{quat}), 128.8 (CH), 130.5 (C_{quat}), 130.7 (CH), 132.1 (CH), 133.1 (CH), 164.6 (C_{quat}), 176.8 (C_{quat}). EI-MS (70 eV, *m/z* (%)): 237 (15), 236 ($[M]^+$, 89), 209 (16), 208 ($[M-CO]^+$, 100), 194 (13), 193 ($[M-C_2H_3O]^+$, 86), 165 (47), 164 (13), 163 (8), 135 ($[M-C_8H_5]^+$, 8), 129 (46), 101 ($[M-C_8H_7O_2]^+$, 8), 77 ($[M-C_{10}H_7O_2]^+$, 9).

1-[4-(Dimethylamino)phenyl]-3-phenylprop-2-yn-1-one (3c) [3]

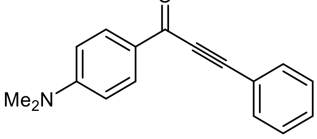

$C_{17}H_{15}NO$
249.31 g/mol

According to GP1 and column chromatography on silica gel (*n*-hexane/EtOAc 5:1 to 103:1) and recrystallization from ethanol (26 mL) 116 mg (23%) of compound **3c** was obtained as a yellow solid, Mp 153 °C, R_f (*n*-hexane/EtOAc 5:1) = 0.21.

^{1}H NMR (600 MHz, $CDCl_3$): δ 3.09 (s, 6 H), 6.64-6.74 (m, 2 H), 7.36-7.47 (m, 3 H), 7.62-7.68 (m, 2 H), 8.08-8.14 (m, 2 H). ^{13}C NMR (151 MHz, $CDCl_3$): δ 40.2 (CH_3), 87.5 (C_{quat}), 91.2 (C_{quat}), 110.8 (CH), 121.0 (C_{quat}), 125.7 (C_{quat}), 128.7 (CH), 130.3 (CH), 132.1 (CH), 132.9 (CH), 154.2 (C_{quat}), 176.1 (C_{quat}). EI-MS (70 eV, *m/z* (%)): 249 ($[M]^+$, 100), 221 ($[M-CO]^+$, 72), 220 (67), 205 ($[M-C_2H_6N]^+$, 18), 178 (10), 176 (10), 129 ($M-C_9H_5O]^+$, 28), 110 (23).

3-Phenyl-1-[4-(trifluoromethyl)phenyl]prop-2-yn-1-one (3d) [4]

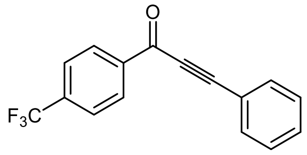

$C_{16}H_9F_3O$
274.24 g/mol

According to GP1 and column chromatography on silica gel (*n*-hexane/EtOAc 20:1) 490 mg (89%) of compound **3d** was obtained as a light brown solid, Mp 87 °C, R_f (*n*-hexane/EtOAc 20:1) = 0.11.

^{1}H NMR (300 MHz, $CDCl_3$): δ 7.40-7.57 (m, 3 H), 7.66-7.74 (m, 2 H), 7.75-7.82 (m, 2 H), 8.28-8.36 (m, 2 H). ^{13}C NMR (75 MHz, $CDCl_3$): δ 86.7 (C_{quat}), 94.6 (C_{quat}), 119.8 (C_{quat}), 123.7 (q, J_{C-F} = 272.8 Hz, C_{quat}), 125.9 (q, J_{C-F} = 3.8 Hz, CH), 129.0 (CH), 130.0 (CH), 131.4 (CH), 133.4 (CH), 135.3 (q, J_{C-F} = 32.8 Hz, C_{quat}), 139.5 (C_{quat}), 176.9 (C_{quat}). EI-MS (70 eV, *m/z* (%)): 275 (9), 274 ($[M]^+$, 55), 247 (11), 246 ($[M-CO]^+$, 71), 145 ($[M-C_9H_5O]^+$, 5), 130 (10), 129 ($[M-C_7H_4F_3]^+$, 100), 101 ($[M-C_8H_4F_3O]^+$, 9), 98 (10), 75 ($[M-C_{10}H_6F_3O]^+$, 11).

4-(3-Phenylpropioloyl)benzonitrile (3e) [5]

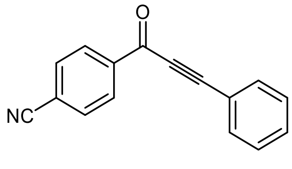

$C_{16}H_9NO$
231.25 g/mol

According to GP1 and column chromatography on silica gel (*n*-hexane/EtOAc 20:1 to 10:1) 297 mg (64%) of compound **3e** was obtained as a beige solid, Mp 133 °C, R_f (*n*-hexane/EtOAc 5:1) = 0.38.

^{1}H NMR (300 MHz, $CDCl_3$): δ 7.41-7.58 (m, 3 H), 7.66-7.73 (m, 2 H), 7.79-7.87 (m, 2 H), 8.26-8.34 (m, 2 H). ^{13}C NMR (75 MHz, $CDCl_3$): δ 86.6 (C_{quat}), 95.3 (C_{quat}), 117.3 (C_{quat}), 118.0 (C_{quat}), 119.6 (C_{quat}), 129.0 (CH), 129.9 (CH), 131.5 (CH), 132.6 (CH), 133.4 (CH), 139.8 (C_{quat}),

176.3 (C_{quat}). EI-MS (70 eV, *m/z* (%)): 231 ($[M]^+$, 48), 204 (10), 203 ($[M-CO]^+$, 63), 130 (10), 129 ($[M-C_7H_4N]^+$, 100), 101 ($[M-C_8H_4NO]^+$, 15), 75 ($[M-C_{10}H_6ON]^+$, 14).

4-(3-Oxo-3-phenylprop-1-yn-1-yl)benzonitrile (3i) [6]

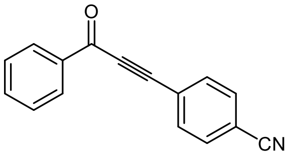

$C_{16}H_9NO$
231.25 g/mol

According to GP1 and column chromatography on silica gel (*n*-hexane/EtOAc 10:1) and recrystallization from EtOAc (12 mL) 547 mg (79%) of compound **3i** was obtained as a colorless solid, Mp 145 °C, R_f (*n*-hexane/EtOAc 5:1) = 0.26.

^{1}H NMR (600 MHz, $CDCl_3$): δ 7.51-7.56 (m, 2 H), 7.65-7.69 (m, 1 H), 7.71-7.74 (m, 2 H), 7.75-7.79 (m, 2 H), 8.18-8.21 (m, 2 H). ^{13}C NMR (151 MHz, $CDCl_3$): δ 89.5 (C_{quat}), 89.8 (C_{quat}), 114.2 (C_{quat}), 118.0 (C_{quat}), 125.1 (C_{quat}), 128.9 (CH), 129.8 (CH), 132.5 (CH), 133.4 (CH), 134.7 (CH), 136.6 (C_{quat}), 177.6 (C_{quat}). EI-MS (70 eV, *m/z* (%)): 231 ($[M]^+$, 37), 204 (16), 203 ($[M-CO]^+$, 100), 154 ($[M-C_6H_5]^+$, 51), 126 ($[M-C_7H_5O]^+$, 6), 105 ($[M-C_9H_4N]^+$, 8), 77 ($[M-C_{10}H_4NO]^+$, 10).

3-Phenyl-1-(thiophen-2-yl)prop-2-yn-1-one (3n) [3]

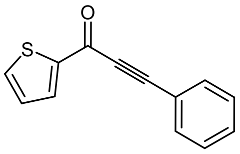

$C_{13}H_8OS$
212.27 g/mol

According to GP1 and column chromatography on silica gel (*n*-hexane/EtOAc 40:1) 338 mg (80%) of compound **3n** was obtained as a light orange solid, Mp 56 °C, R_f (*n*-hexane/EtOAc 20:1) = 0.18.

^{1}H NMR (300 MHz, $CDCl_3$): δ 7.19 (dd, J = 4.9, 3.8 Hz, 1 H), 7.38-7.53 (m, 3 H), 7.63-7.70 (m, 2 H), 7.73 (dd, J = 4.9, 1.2 Hz, 1 H), 8.01 (dd, J = 3.8, 1.2 Hz, 1 H). ^{13}C NMR (75 MHz, $CDCl_3$): δ 86.6 (C_{quat}), 91.9 (C_{quat}), 120.1 (C_{quat}), 128.5 (CH), 128.8 (CH), 131.0 (CH), 133.2 (CH), 135.2 (CH), 135.4 (CH), 145.1 (C_{quat}), 169.9 (C_{quat}). EI-MS (70 eV, *m/z* (%)): 212 ($[M]^+$, 62), 185 (14), 184 ($[M-CO]^+$, 100), 152 (20), 139 (15), 129 ($[M-C_4H_3S]^+$, 41), 101 ($[M-C_5H_3OS]^+$, 7), 83 ($[M-C_9H_5O]^+$, 2), 75 (10).

2.2 Kumada–Sonogashira synthesis of alkynones 3

General procedure 2 (GP2) according to literature [7]

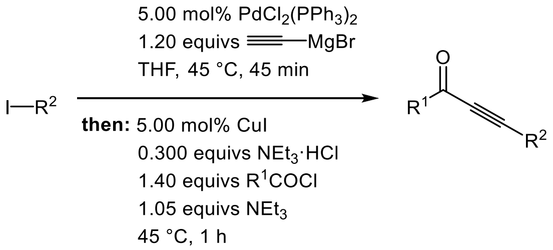

In a Schlenk tube with magnetic stirring bar were placed $PdCl_2(PPh_3)_2$ (5.00 mol %) and, if a solid, aryl iodide (1.00 equiv), and evacuated and flushed with a nitrogen atmosphere three times (for experimental details, see Table S2). Then, ethynylmagnesium bromide in THF (0.500 M, 1.20 equiv) and, if a liquid, aryl iodide (1.00 equiv), was added. The mixture was stirred at 45 °C (oil bath) for 45 min (monitored by TLC) to full conversion. After cooling to room temp triethylamine hydrochloride (0.300 equiv) was added to the reaction mixture. After 5 min triethylamine (1.05 equiv), acid chloride (1.40 equiv), and CuI (5.00 mol %) were added and the mixture stirred at 45 °C (oil bath) for 1 h. After cooling to room temp the reaction was stopped by adding a saturated aqueous NaCl solution (5.00 mL). The aqueous phase was extracted with dichloromethane (3 × 50 mL). The combined organic phases were dried (anhydrous magnesium sulfate). After filtration the crude product was adsorbed on celite® and purified by flash chromatography on silica gel (*n*-hexane/EtOAc) to give the analytically pure alkynones **3**.

Table S2. Experimental details for the synthesis of alkynones 3 starting from aryl iodides.

Entry	aryliodide	ethynylmagnesium bromide	acid chloride	$PdCl_2(PPh_3)_2$ / CuI	$NEt_3 \cdot HCl$ / NEt_3	alkynone **3**
1	242 mg (1.01 mmol) of 4-iodoanisole	2.40 mL (1.20 mmol)	201 mg (1.42 mmol) of benzoyl chloride	35.1 mg (50.0 µmol) / 9.50 mg (50.0 µmol)	43.1 mg (500 µmol) / 106 mg (1.05 mmol)	205 mg (87%) of **3f**
2	175 mg (710 µmol) of *N*,*N*-dimethyl-4-iodoaniline	1.70 mL (850 µmol)	143 mg (1.01 mmol) of benzoyl chloride	25.0 mg (35.5 µmol) / 6.74 mg (35.5 µmol)	29.3 mg (210 µmol) / 75.0 mg (740 µmol)	139 mg (78 %) of **3g**
3	279 mg (990 µmol) of 4-iodobenzotrifluoride	2.40 mL (1.20 mmol)	200 mg (1.41 mmol) of benzoyl chloride	35.1 mg (50.0 µmol) / 9.50 mg (50.0 µmol)	43.1 mg (500 µmol) / 106 mg (1.05 mmol)	130 mg (47%) of **3h**
4	1.40 g (6.00 mmol) of 4-iodoanisole	14.4 mL (7.20 mmol)	1.45 g (8.40 mmol) of 4-methoxybenzoyl chloride	211 mg (300 µmol) / 57.0 mg (300 µmol)	248 mg (1.80 mmol) / 636 mg (6.30 mg)	1.03 g (65%) of **3j**

5	840 mg (3.00 mmol) of 4-iodobenzotri-fluoride	7.20 mL (3.60 mmol)	723 mg (4.20 mmol) of 4-methoxybenzoyl chloride	105 mg (150 µmol) / 28.5 mg (150 µmol)	124 mg (900 µmol) 318 mg (3.15 mmol)	596 mg (65%) of **3k**
6	717 mg (3.00 mmol) of 4-iodoanisole	7.20 mL (3.60 mmol)	894 mg (4.20 mmol) of 4-(trifluoro-methyl)benzoyl chloride	105 mg (150 µmol) / 28.5 mg (150 µmol)	124 mg (900 µmol) 318 mg (3.15 mmol)	598 mg (66%) of **3l**
7	247 mg (1.00 mmol) of *N*,*N*-di-methyl-4-iodoaniline	2.40 mL (1.20 mmol)	299 mg (1.40 mmol) of 4-(trifluoro-methyl)benzoyl chloride	35.1 mg (50.0 µmol) / 9.50 mg (50.0 µmol)	43.1 mg (500 µmol) / 106 mg (1.05 mmol)	273 mg (86%) of **3m**

3-(4-Methoxyphenyl)-1-phenylprop-2-yn-1-one (3f) [3]

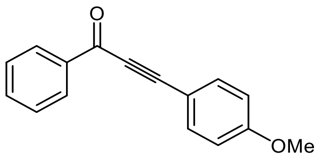

$C_{16}H_{12}O_2$
236.27 g/mol

According to GP2 and column chromatography on silica gel (*n*-hexane/EtOAc 20:1 to 10:1) 205 mg (87%) of compound **3f** was obtained as a beige solid, Mp 72 °C, R_f (*n*-hexane/EtOAc 20:1) = 0.21.

^{1}H NMR (300 MHz, $CDCl_3$): δ 3.86 (s, 3 H), 6.89-6.97 (m, 2 H), 7.45-7.56 (m, 2 H), 7.59-7.68 (m, 3 H), 8.16-8.25 (m, 2 H). ^{13}C NMR (75 MHz, $CDCl_3$): δ 55.6 (CH_3), 87.0 (C_{quat}), 94.5 (C_{quat}), 112.1 (C_{quat}), 114.6 (CH), 128.7 (CH), 129.6 (CH), 134.0 (CH), 135.3 (CH), 137.2 (C_{quat}), 161.9 (C_{quat}), 178.2 (C_{quat}). EI-MS (70 eV, *m/z* (%)): 237 (17), 236 ([M]$^+$, 100), 209 (8), 208 ([M-CO]$^+$, 51), 205 ([M-CH_3O]$^+$, 5), 194 (9), 193 ([M-C_2H_3O]$^+$, 62), 165 (44), 164 (11), 160 (10), 159 ([M-C_6H_5]$^+$, 97), 144 ([M-C_7H_8]$^+$, 16), 131 ([M-C_7H_5O]$^+$, 5), 116 ([M-C_8H_8O]$^+$, 15), 88 (12), 77 ([M-$C_{10}H_7O_2$]$^+$, 16).

3-[4-(Dimethylamino)phenyl]-1-phenylprop-2-yn-1-one (3g) [7]

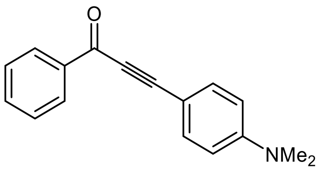

$C_{17}H_{15}NO$
249.31 g/mol

According to GP2 and column chromatography on silica gel (*n*-hexane/EtOAc 19:1) 139 mg (78%) of compound **3g** was obtained as a beige solid, Mp 124 °C, R_f (*n*-hexane/EtOAc 5:1) = 0.30.

^{1}H NMR (300 MHz, $CDCl_3$): δ 3.05 (s, 6 H), 6.63-6.71 (m, 2 H), 7.47-7.54 (m, 2 H), 7.54-7.64 (m, 3 H), 8.20-8.27 (m, 2 H). ^{13}C NMR (75 MHz, $CDCl_3$): δ 40.2 (CH_3), 88.0 (C_{quat}), 97.7 (C_{quat}), 105.8 (C_{quat}), 111.7 (CH), 128.6 (CH), 129.5 (CH), 133.6 (CH), 135.3 (CH), 137.6 (C_{quat}), 151.9 (C_{quat}), 178.1 (C_{quat}). EI-MS (70 eV, *m/z* (%)): 250 (13), 249 ($[M]^+$, 71), 248 (22), 221 ($[M-CO]^+$, 13), 205 ($[M-C_2H_6N]^+$, 11), 172 ($[M-C_6H_5]^+$, 35), 167 (10), 166 (100), 165 (25), 144 ($[M-C_7H_5O]^+$, 17), 120 ($[M-C_9H_5O]^+$, 10), 119 (31), 118 (10), 105 ($[M-C_{10}H_{10}N]^+$, 15), 104 (13), 91 (10), 77 ($[M-C_{11}H_{10}NO]^+$, 21), 42 ($[M-C_{15}H_9O]^+$, 13).

1-Phenyl-3-[4-(trifluormethyl)phenyl]prop-2-yn-1-one (3h) [8]

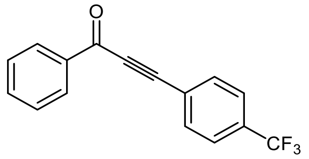

$C_{16}H_9F_3O$
274.24 g/mol

According to GP2 and column chromatography on silica gel (*n*-hexane/EtOAc 20:1) 130 mg (47%) of compound **3h** was obtained as a beige solid, Mp 80 °C, R_f (*n*-hexane/EtOAc 20:1) = 0.29.

^{1}H NMR (300 MHz, $CDCl_3$): δ 7.50-7.58 (m, 2 H), 7.62-7.73 (m, 3 H), 7.76-7.83 (m, 2 H), 8.18-8.25 (m, 2 H). ^{13}C NMR (75 MHz, $CDCl_3$): δ 88.2 (C_{quat}), 90.6 (C_{quat}), 123.7 (q, J_{C-F} = 272.6 Hz, C_{quat}), 124.1 (q, J_{C-F} = 1.8 Hz, C_{quat}), 125.8 (q, J_{C-F} = 3.8 Hz, CH), 128.9 (CH), 129.8 (CH), 132.4 (q, J_{C-F} = 32.9 Hz, C_{quat}), 133.3 (CH), 134.6 (CH), 136.7 (C_{quat}), 177.8 (C_{quat}). EI-MS (70 eV, *m/z* (%)): 275 (8), 274 ($[M]^+$, 47), 247 (17), 246 ($[M-CO]^+$, 100), 245 (7), 197 ($[M-C_6H_5]^+$, 60), 98 (12), 77 ($[M-C_{10}H_4F_3O]^+$, 8).

1,3-Bis(4-methoxyphenyl)prop-2-yn-1-one (3j) [9]

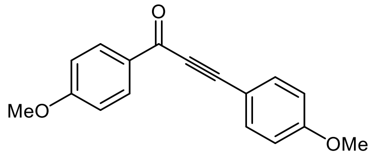

$C_{17}H_{14}O_3$
266.30 g/mol

According to GP2 and column chromatography on silica gel (*n*-hexane/EtOAc 20:1 to 10:1) 1.03 g (65%) of compound **3j** was obtained as a beige solid, Mp 72 °C, R_f (*n*-hexane/EtOAc 20:1) = 0.17.

^{1}H NMR (300 MHz, $CDCl_3$): δ 3.86 (s, 3 H), 3.90 (s, 3 H), 6.90-6.95 (m, 2 H), 6.96-7.01 (m, 2 H), 7.58-7.67 (m, 2 H), 8.15-8.25 (m, 2 H). ^{13}C NMR (75 MHz, $CDCl_3$): δ 55.6 (CH_3), 55.7 (CH_3), 86.9 (C_{quat}), 93.6 (C_{quat}), 112.3 (C_{quat}), 114.0 (CH), 114.5 (CH), 130.6 (C_{quat}), 132.0 (CH), 135.1 (CH), 161.7 (C_{quat}), 164.5 (C_{quat}), 176.9 (C_{quat}). EI-MS (70 eV, *m/z* (%)): 267 (18), 266

([M]$^+$, 100), 238 ([M-CO]$^+$, 56), 224 (11), 223 (63), 195 (13), 159 ([M-C_7H_7O]$^+$, 41), 119 ([M-$C_{10}H_9O_2$]$^+$, 14).

1-(4-Methoxyphenyl)-3-[4-(trifluoromethyl)phenyl]prop-2-yn-1-one (3k) [10]

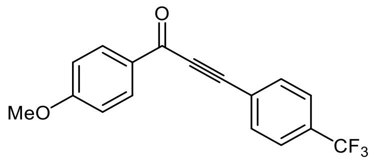

$C_{17}H_{11}F_3O_2$
304.27 g/mol

According to GP2 and column chromatography on silica gel (*n*-hexane/EtOAc 10:1) and recrystallization from *n*-hexane (11 mL) 596 mg (65%) of compound **3j** was obtained as a beige solid, Mp 105 °C, R_f (*n*-hexane/EtOAc 20:1) = 0.16.

^{1}H NMR (300 MHz, $CDCl_3$): δ 3.90 (s, 3 H), 6.94-7.01 (m, 2 H), 7.62-7.82 (m, 4 H), 8.11-8.22 (m, 2 H). ^{13}C NMR (75 MHz, $CDCl_3$): δ 55.8 (CH_3), 88.4 (C_{quat}), 89.9 (C_{quat}), 114.2 (CH), 121.9 (C_{quat}), 124.3 (C_{quat}), 125.7 (q, $J_{C\text{-}F}$ = 3.8 Hz, CH), 130.1 (C_{quat}), 132.2 (CH), 133.2 (CH), 164.9 (C_{quat}), 176.4 (C_{quat}). C_{quat} of the CF_3 group is superpositioned by other signals. EI-MS (70 eV, *m/z* (%)): 305 (13), 304 ([M]$^+$, 76), 277 (17), 276 ([M-CO]$^+$, 100), 261 ([M-C_2H_3O]$^+$, 60), 233 (40), 232 (14), 197 ([M-C_7H_7O]$^+$, 48), 183 (13), 169 ([M-$C_8H_7O_2$]$^+$, 7), 138 (11), 135 ([M-$C_9H_4F_3$]$^+$, 31), 92 (14), 77 ([M-$C_{11}H_6F_3O_2$]$^+$, 13).

3-(4-Methoxyphenyl)-1-[4-(trifluoromethyl)phenyl]prop-2-yn-1-one (3l) [10]

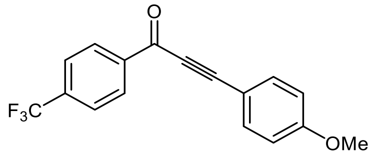

$C_{17}H_{11}F_3O_2$
304.27 g/mol

According to GP2 and column chromatography on silica gel (*n*-hexane/EtOAc 10:1) and recrystallization from *n*-hexane (26 mL) 598 mg (66%) of compound **3l** was obtained as a beige solid, Mp 118-120 °C, R_f (*n*-hexane/EtOAc 20:1) = 0.27.

^{1}H NMR (300 MHz, $CDCl_3$): δ 3.87 (s, 3 H), 6.90-7.01 (m, 2 H), 7.61-7.70 (m, 2 H), 7.74-7.81 (m, 2 H), 8.32 (m, 2 H). ^{13}C NMR (75 MHz, $CDCl_3$): δ 55.6 (CH_3), 86.9 (C_{quat}), 96.0 (C_{quat}), 111.5 (C_{quat}), 114.7 (CH), 123.7 (d, $J_{C\text{-}F}$ = 272.8 Hz, C_{quat}), 125.8 (q, $J_{C\text{-}F}$ = 3.8 Hz, CH), 129.9 (CH), 135.1 (q, $J_{C\text{-}F}$ = 32.7 Hz, C_{quat}), 135.5 (CH), 139.7 (C_{quat}), 162.2 (C_{quat}), 176.8 (C_{quat}). EI-MS (70 eV, *m/z* (%)): 305 (12), 304 ([M]$^+$, 59), 276 (16), 261 ([M-C_2H_3O]$^+$, 12), 159 ([M-$C_7H_4F_3$]$^+$, 100), 145 ([M-$C_{10}H_7O_2$]$^+$, 7), 144 (12).

3-[4-(Dimethylamino)phenyl]-1-[4-(trifluormethyl)phenyl]prop-2-yn-1-one (3m) [7]

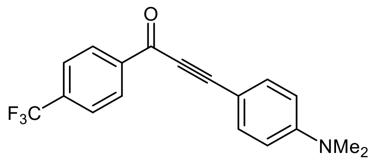

$C_{18}H_{14}F_3NO$
317.31 g/mol

According to GP2 and column chromatography on silica gel (*n*-hexane/EtOAc 20:1) 273 mg (86%) of compound **3m** was obtained as a red solid, Mp 118 °C, R_f (*n*-hexane/EtOAc 5:1) = 0.33.

^{1}H NMR (300 MHz, $CDCl_3$): δ 3.07 (s, 6 H), 6.63-6.71 (m, 2 H), 7.53-7.62 (m, 2 H), 7.72-7.82 (m, 2 H), 8.29-8.35 (m, 2 H). ^{13}C NMR (75 MHz, $CDCl_3$): δ 40.2 (CH_3), 88.2 (C_{quat}), 99.5 (C_{quat}), 105.2 (C_{quat}), 111.7 (CH), 125.7 (q, J_{C-F} = 3.8 Hz, CH), 129.7 (CH), 134.7 (q, J_{C-F} = 32.5 Hz, C_{quat}), 135.5 (CH), 140.2 (C_{quat}), 152.2 (C_{quat}), 176.6 (C_{quat}). C_{quat} of the CF_3 group is superpositioned by other signals. EI-MS (70 eV, *m/z* (%)): 317 ($[M]^+$, 100), 316 (44), 289 ($[M-CO]^+$, 13), 288 (27), 273 ($[M-C_2H_6N]^+$, 10), 173 ($[M-C_{10}H_{10}N]^+$, 12), 172 (54), 145 ($[M-C_{11}H_{10}NO]^+$, 100), 144 (35).

2.3 One-pot synthesis of 3-ethyl (*Z*)-2-cyano-2-(4,6-diphenyl-1*H*-pyridin-2-ylidene)acetate (5a)

In a manner similar to our method [11] $PdCl_2(PPh_3)_2$ (3.50 mg, 5.00 µmol, 0.250 mol %) and CuI (1.90 mg, 10.0 µmol, 0.50 mol %) were placed in a dry Schlenk tube under a nitrogen atmosphere and anhydrous 1,4-dioxane (2.00 mL) was added. Then, benzoyl chloride (**1a**, 312 mg, 2.20 mmol), phenylacetylene (**2a**, 206 mg, 2.00 mmol) and NEt_3 (212 mg, 2.10 mmol) were added and the mixture was stirred at 20 °C for 3 h until complete conversion (monitored by TLC). Afterwards, ethanol (2.00 mL), $Na_2CO_3\cdot 10H_2O$ (1.72 g, 6.00 mmol) and ethyl cyanoacetate (**4**, 914 mg, 8.00 mmol) were added and stirring at 75 °C was continued for 16 h. After the addition of CH_2Cl_2 (5.00 mL) and $NaOH/FeSO_4$ solution (5.00 mL), the solution was extracted with CH_2Cl_2 (3 × 50.0 mL). The combined organic layers were dried (anhydrous $MgSO_4$) and the solvent was removed in vacuo. The residue was purified by flash chromatography on silica gel (*n*-hexane-EtOAc, 25:1 → 5:1 → 1:1) and washed with hot ethanol (2.00 mL). 207 mg (0.600 mmol, 30%) of compound **5a** was obtained as bright yellow solid. Mp 178-180 °C.

^{1}H NMR (300 MHz, $CDCl_3$): δ 1.37 (t, J = 7.1 Hz, 3 H), 4.29 (q, J = 7.1 Hz, 2H), 7.17 (dd, J = 1.5, 1.6 Hz, 1 H), 7.45 (dd, J = 1.5, 1.6 Hz, 1 H), 7.48-7.54 (m, 3 H), 7.55-7.61 (m, 3 H), 7.66-7.74 (m, 2 H), 7.77-7.84 (m, 2 H), 14.50 (s, 1 H). ^{13}C NMR (75 MHz, $CDCl_3$): δ 14.8 (CH_3), 60.4 (CH_2), 62.8 (C_{quat}), 109.6 (CH), 115.4 (CH), 119.9 (C_{quat}), 126.1 (CH), 127.3 (CH), 129.4 (CH), 130.0 (CH), 130.6 (CH), 131.4 (CH), 132.5 (C_{quat}), 136.9 (C_{quat}), 146.1 (C_{quat}), 152.8 (C_{quat}),

155.9 (C_{quat}), 171.1 (C_{quat}). EI-MS (70 eV, *m/z* (%)): 342 ($[M]^+$, 100), 297 ($[M - C_2H_5O]^+$, 12), 273 ($[M - C_3H_7NO]^+$, 97), 270 ($[M - C_3H_5O_2]^+$, 35), 245 ($[M - C_4H_5NO_2]^+$, 100), 231 ($[M - C_5H_5NO_2]^+$, 2), 217 ($[M - C_5H_6N_2O_2]^+$, 11), 191 ($[M - C_7H_7N_2O_2]^+$, 33), 140 ($[M - C_{16}H_{12}]^+$, 17), 105 ($[M - C_{15}H_{12}NO_2]^+$, 32), 77 ($[M - C_{16}H_{13}N_2O_2]^+$, 30). IR (ATR): $\tilde{\nu}$ [cm^{-1}] 3096 (w), 3059 (w), 2984 (w), 2951 (w), 2903 (w), 2864 (w), 2768 (w), 2195 (m), 1624 (s), 1605 (m), 1593 (m), 1578 (m), 1481 (w), 1458 (w), 1439 (w), 1420 (w), 1395 (w), 1368 (m), 1308 (m), 1279 (s), 1256 (m), 1167 (m), 1157 (m), 1094 (s), 1082 (m), 1049 (m), 1028 (w), 982 (m), 968 (w), 882 (m), 856 (s), 828 (w), 762 (s), 733 (w), 696 (s), 635 (w). UV/Vis (CH_2Cl_2): λ_{max} [nm] (ε [$L\cdot mol^{-1}\cdot cm^{-1}$]) = 281 (26100), 319 (17400), 418 (9800). Emission (CH_2Cl_2): λ_{max} [nm] (Stokes shift [cm^{-1}]) = 545 (5600); quantum yield (CH_2Cl_2): Φ_f = 0.02; emission (solid): λ_{max} [nm] = 540. Anal. calcd. for $C_{22}H_{18}N_2O_2$ (342.1): C 77.17, H 5.30, N 8.18; Found: C 76.88, H 5.24, N 8.13.

2.4 Synthesis of 2-oxo-4,6-diphenyl-2*H*-pyran-3-carbonitrile (6a) [12]

Alkynone **3a** (103 mg, 0.500 mmol) was placed in a dry Schlenk tube and ethanol (1.00 mL) was added. Sodium carbonate (43.0 mg, 400 µmol), sodium acetate (25.0 mg, 300 µmol), water (50.0 µL, 2.80 mmol) and ethyl cyanoacetate (**4**, 231 mg, 2.00 mmol) were added and the mixture was stirred at 20 °C for 72 h. After the addition of CH_2Cl_2 (5.00 mL) and $NaOH/FeSO_4$ solution (5.00 mL), the solution was extracted with CH_2Cl_2 (3 × 50.0 mL). The combined organic layers were dried (anhydrous $MgSO_4$) and the solvent was removed in vacuo. The residue was purified by flash chromatography on silica gel (*n*-hexane/EtOAc 5:1 to 1:1) and washed with hot ethanol (2.00 mL). 107 mg (0.390 mmol, 78%) of compound **6a** were obtained as yellow solid. Mp 194 °C.

^{1}H NMR (300 MHz, $CDCl_3$): δ 6.94 (s, 1 H), 7.44-7.66 (m, 6 H), 7.69-7.77 (m, 2 H), 7.89-7.97 (m, 2 H). ^{13}C NMR (75 MHz, $CDCl_3$): δ 95.7 (C_{quat}), 103.3 (CH), 114.6 (C_{quat}), 126.7 (CH), 128.1 (CH), 129.48 (CH), 129.50 (CH), 130.1 (C_{quat}), 132.2 (CH), 132.9 (CH), 134.4 (C_{quat}), 159.2 (C_{quat}), 163.4 (C_{quat}), 164.1 (C_{quat}). EI-MS (70 eV, *m/z* (%)): 273 ($[M]^+$, 100), 246 (19), 245 ($[M - CO]^+$, 100), 191 ($[M - C_3NO_2]^+$, 4), 140 ($[M - C_8H_5O_2]^+$, 17), 105 ($[M - C_{11}H_6NO]^+$, 32), 77 ($[M - C_{12}H_6NO_2]^+$, 29). IR (ATR): $\tilde{\nu}$ [cm^{-1}] 3061 (w), 3038 (w), 2328 (w), 2228 (w), 1913 (w), 1722 (s), 1721 (s), 1684 (m), 1607 (m), 1578 (m), 1558 (w), 1508 (s), 1493 (s), 1452 (m), 1441 (m), 1381 (m), 1350 (m), 1342 (m), 1292 (w), 1240 (m), 1209 (w), 1190 (w), 1155 (w), 1101 (w), 1076 (w), 1053 (m), 1015 (w), 947 (m), 924 (w), 833 (m), 777 (m), 758 (s), 698 (s), 685 (s), 667 (m), 640 (m), 615 (w). UV/Vis (CH_2Cl_2): λ_{max} [nm] (ε [$L\cdot mol^{-1}\cdot cm^{-1}$]) = 258 (15300), 311 (12800), 381 (18000). Emission (solid): λ_{max} [nm] = 499. Anal. calcd. for $C_{18}H_{11}NO_2$ (273.1): C 79.11, H 4.06, N 5.13; Found C 79.12, H 3.89, N 5.02.

2.5 General procedure for the synthesis of 1*H*-pyridines 5 and α-pyrones 6 (GP3)

Alkynone **3a** (1.00 equiv) was placed in a dry Schlenk tube and ethanol (0.500 M) was added. Sodium carbonate (0.800 equiv), sodium acetate (0.600 equiv), water (5.60 equiv) and ethyl cyanoacetate (**4**, 4.00 equiv) were added and the mixture was stirred at 75 °C for 16 h. After the addition of CH_2Cl_2 (5.00 mL) and NaOH/$FeSO_4$ solution (5.00 mL), the solution was extracted with CH_2Cl_2 (3 × 50.0 mL). The combined organic layers were dried (anhydrous $MgSO_4$) and the solvent was removed in vacuo. The residue was purified by flash chromatography on silica gel and washed with hot ethanol.

2.5.1 Ethyl (*Z*)-2-cyano-2-(4,6-diphenyl-1*H*-pyridin-2-ylidene)acetate (5a)

According to GP3 using 1,3-diphenylprop-2-yn-1-one (**3a**) (103 mg, 500 µmol), sodium carbonate (43.0 mg, 400 µmol), sodium acetate (25.0 mg, 300 µmol) and ethyl cyanoacetate (**4**) (231 mg, 2.00 mmol). After flash chromatography on silica gel (*n*-hexane/EtOAc 5:1 to 1:1) and washing with hot ethanol (2.00 mL), compound **5a** (95.0 mg, 278 µmol, 56%) was obtained as a bright yellow solid (for analytical data, see 2.3).

2.5.2 Ethyl (*Z*)-2-cyano-2-{4-phenyl-6-[4-(trifluoromethyl)phenyl]-1*H*-pyridin-2-ylidene}-acetate (5b)

According to GP3 using 3-phenyl-1-[4-(trifluoromethyl)phenyl]prop-2-yn-1-one (**3d**, 823 mg, 3.00 mmol), sodium carbonate (254 mg, 2.40 mmol), sodium acetate (149 mg, 1.80 mmol) and ethyl cyanoacetate (**4**, 1.39 g, 12.0 mmol) and after flash chromatography on silica gel (*n*-hexane/EtOAc 5:1 to 1:1) and washing with hot ethanol (4.00 mL), compound **5b** (270 mg, 660 µmol, 22%) was obtained as orange solid. Mp 170 °C.

^{1}H NMR (300 MHz, $CDCl_3$): δ 1.37 (t, J = 7.1 Hz, 3 H), 4.29 (q, J = 7.1 Hz, 2 H), 7.18 (dd, J = 1.5, 1.6 Hz, 1 H), 7.49 (dd, J = 1.5, 1.6 Hz, 1 H), 7.51-7.57 (m, 3 H), 7.65-7.75 (m, 2 H), 7.81-7.88 (m, 2 H), 7.89-7.97 (m, 2 H), 14.62 (s, 1 H). ^{13}C NMR (75 MHz, $CDCl_3$): δ 14.7 (CH_3), 60.7 (CH_2), 63.4 (C_{quat}), 110.2 (CH), 116.3 (CH), 119.4 (C_{quat}), 123.6 (q, $J_{C\text{-}F}$ = 273 Hz, C_{quat}), 126.6 (CH), 127.0 (q, $J_{C\text{-}F}$ = 3.7 Hz, CH), 127.3 (CH), 129.5 (CH), 130.8 (CH), 133.1 (q, $J_{C\text{-}F}$ = 33.0 Hz, C_{quat}), 136.0 (C_{quat}), 136.6 (C_{quat}), 144.3 (C_{quat}), 152.6 (C_{quat}), 156.0 (C_{quat}), 171.1 (C_{quat}). EI-MS (70 eV, *m/z* (%)): 410 ([M]$^+$, 8), 365 ([M - C_2H_5O]$^+$, 3), 339 (22), 338 ([M - $C_3H_4O_2$]$^+$, 100), 337 (17), 299 ([M - $C_5H_5NO_2$]$^+$, 4), 149 ([M - $C_{16}H_{12}F_3$]$^+$, 11), 77 ([M - $C_{17}H_{12}F_3N_2O_2$]$^+$, 4). IR (ATR): $\tilde{\nu}$ [cm^{-1}] 3049 (w), 2994 (w), 2941 (w), 2872 (w), 2203 (m), 1626 (m), 1599 (m), 1574 (m), 1504 (w), 1445 (w), 1395 (w), 1368 (m), 1321 (s), 1304 (s), 1283 (s), 1177 (m), 1126 (s), 1090 (m), 1074 (s), 1043 (m), 1030 (w), 1016 (m), 884 (m), 854 (m), 841 (m), 764 (s), 743 (w), 700 (m), 667 (w), 642 (w). UV/Vis (CH_2Cl_2): λ_{max} [nm] (ε [L·mol^{-1}·cm^{-1}]) = 272 (27000), 326 (17500), 424 (8900). Emission (CH_2Cl_2): λ_{max} [nm] (Stokes shift [cm^{-1}]) = 565 (5600);

quantum yield (CH_2Cl_2): Φ_f = 0.01; emission (solid): λ_{max} [nm] = 604. Anal. calcd. for $C_{23}H_{17}F_3N_2O_2$ (410.1): C 67.31, H 4.18, N 6.83; Found: C 67.39, H 4.28, N 6.66.

2.5.3 Ethyl (*Z*)-2-cyano-2-[6-(4-cyanophenyl)-4-phenyl-1*H*-pyridin-2-ylidene]acetate (5c)

According to GP3 using 4-(3-phenylpropioloyl)benzonitrile (**3e**, 463 mg, 2.00 mmol), sodium carbonate (172 mg, 1.60 mmol), sodium acetate (100 mg, 1.20 mmol) and ethyl cyanoacetate (**4**, 924 mg, 8.00 mmol) and after flash chromatography on silica gel (*n*-hexane/EtOAc 5:1 to 1:1) and washing with hot ethanol (4.00 mL), compound **5c** (147 mg, 400 µmol, 22%) was obtained as orange solid. Mp 225-228 °C.

^{1}H NMR (300 MHz, $CDCl_3$): δ 1.37 (t, *J* = 7.1 Hz, 3 H), 4.30 (q, *J* = 7.1 Hz, 2 H), 7.18 (dd, *J* = 1.5, 1.8 Hz, 1 H), 7.51 (dd, *J* = 1.5, 1.8 Hz, 1 H), 7.52-7.57 (m, 3 H), 7.64-7.73 (m, 2 H), 7.84-7.97 (m, 4 H), 14.64 (s, 1 H). ^{13}C NMR (75 MHz, $CDCl_3$): δ 14.7 (CH_3), 60.8 (CH_2), 63.7 (C_{quat}), 110.5 (CH), 114.9 (C_{quat}), 116.8 (CH), 117.8 (C_{quat}), 119.2 (C_{quat}), 126.8 (CH), 127.3 (CH), 129.6 (CH), 130.9 (CH), 133.7 (CH), 136.5 (C_{quat}), 136.6 (C_{quat}), 143.6 (C_{quat}), 152.6 (C_{quat}), 156.2 (C_{quat}), 171.2 (C_{quat}). EI-MS (70 eV, *m/z* (%)): 368 (10), 367 ([M]$^+$, 36), 322 ([M - C_2H_5O]$^+$, 12), 321 (15), 296 ([M - C_3H_5NO]$^+$, 29), 295 (100), 294 ([M - $C_3H_5O_2$]$^+$, 14), 293 (13), 270 (11), 265 ([M - C_7H_4N]$^+$, 8), 77 ([M - $C_{17}H_{12}N_3O_2$]$^+$, 4). IR (ATR): $\tilde{\nu}$ [cm^{-1}] 3080 (w), 3003 (w), 2918 (w), 2849 (w), 2228 (w), 2189 (m), 1624 (m), 1589 (m), 1578 (m), 1560 (m), 1510 (w), 1508 (m), 1489 (m), 1466 (w), 1439 (m), 1391 (w), 1371 (m), 1344 (w), 1312 (m), 1285 (s), 1260 (m), 1219 (w), 1177 (m), 1155 (m), 1136 (w), 1090 (m), 1082 (m), 1042 (m), 1016 (w), 980 (m), 949 (w), 920 (w), 883 (m), 862 (s), 841 (s), 829 (m), 760 (s), 758 (s), 712 (w), 692 (s), 638 (m), 625 (m), 615 (m). UV/Vis (CH_2Cl_2): λ_{max} [nm] (ε [L·mol^{-1}·cm^{-1}]) = 280 (37600), 333 (17300), 431 (9500). Emission (CH_2Cl_2): λ_{max} [nm] (Stokes shift [cm^{-1}]) = 585 (6100); quantum yield (CH_2Cl_2): Φ_f = 0.01. Anal. calcd. for $C_{23}H_{17}N_3O_2$ (367.1): C 75.19, H 4.66, N 11.44; Found: C 75.02, H 4.64, N 11.15.

2.5.4 Ethyl (*Z*)-2-cyano-2-(6-phenyl-4-(4-(trifluoromethyl)phenyl)pyridin-2(1*H*)-ylidene)acetate (5d)

According to GP3 using 1-phenyl-3-[4-(trifluoromethyl)phenyl]prop-2-yn-1-one (**3h**, 1.40 g, 5.00 mmol), sodium carbonate (430 mg, 4.00 mmol), sodium acetate (250 mg, 3.00 mmol) and ethyl cyanoacetate (**4**, 2.31 g, 20.0 mmol) and after flash chromatography on silica gel (*n*-hexane/EtOAc 12:1 to 5:1 to 0:1) and washing with hot ethanol (5.00 mL), compound **5d** (513 mg, 1.25 mmol, 25%) was obtained as orange solid. Mp 223-233 °C.

^{1}H NMR (300 MHz, $CDCl_3$): δ 1.38 (t, *J* = 7.1 Hz, 3 H), 4.30 (q, *J* = 7.1 Hz, 2 H), 7.12 (dd, *J* = 1.5, 1.6 Hz, 1 H), 7.44 (dd, *J* = 1.5, 1.6 Hz, 1 H), 7.56-7.63 (m, 3 H), 7.78-7.84 (m, 6 H), 14.57 (s, 1 H). ^{13}C NMR (150 MHz, $CDCl_3$): δ 14.7 (CH_3), 60.6 (CH_2), 63.5 (C_{quat}), 109.2 (CH), 116.0

(CH), 119.5 (C_{quat}), 123.9 (q, J_{C-F} = 273 Hz, C_{quat}), 126.1 (CH), 126.2-126.7 (m, CH), 127.8 (CH), 130.1 (CH), 131.6 (CH), 132.3 (C_{quat}), 132.4 (q, J_{C-F} = 32.9 Hz, C_{quat}), 140.6 (C_{quat}), 146.6 (C_{quat}), 151.2 (C_{quat}), 156.0 (C_{quat}), 171.0 (C_{quat}). EI-MS (70 eV, *m/z* (%)): 410 ($[M]^+$, 24), 366 (24), 365 ($[M - C_2H_5O]^+$, 100), 339 (12), 338 ($[M - C_3H_5O_2]^+$, 53), 337 (38), 308 (8), 240 (23), 149 ($[M - C_{16}H_{12}F_3]^+$, 11). IR (ATR): $\tilde{\nu}$ [cm^{-1}] 3092 (w), 2992 (w), 2963 (w), 2943 (w), 2876 (w), 2806 (w), 2193 (m), 1625 (m), 1620 (m), 1597 (m), 1577 (m), 1506 (w), 1466 (w), 1413 (w), 1396 (w), 1369 (w), 1308 (m), 1300 (m), 1283 (s), 1258 (m), 1206 (w), 1165 (m), 1115 (s), 1092 (m), 1082 (m), 1071 (m), 1045 (s), 1030 (m), 1015 (m), 980 (m), 976 (m), 968 (w), 920 (w), 885 (w), 874 (w), 837 (s), 829 (m), 764 (s), 745 (w), 727 (w), 685 (m), 662 (w), 655 (w), 650 (w). UV/Vis (CH_2Cl_2): λ_{max} [nm] (ε [$L\cdot mol^{-1}\cdot cm^{-1}$]) = 274 (32000), 321 (18100), 428 (10000). Emission (CH_2Cl_2): λ_{max} [nm] (Stokes shift [cm^{-1}]) = 565 (5700); quantum yield (CH_2Cl_2): Φ_f = 0.01. Anal. calcd. for $C_{23}H_{17}F_3N_2O_2$ (410.1): C 67.31, H 4.18, N 6.83; Found: C 67.50, H 4.32, N 6.70.

2.5.5 Ethyl (*Z*)-2-cyano-2-(4-(4-cyanophenyl)-6-phenylpyridin-2(1*H*)-ylidene)acetate (5e)

According to GP3 using 4-(3-oxo-3-phenylprop-1-yn-1-yl)benzonitrile (**3i**, 463 mg, 2.00 mmol), sodium carbonate (172 mg, 1.60 mmol), sodium acetate (100 mg, 1.20 mmol) and ethyl cyano-acetate (**4**, 924 mg, 8.00 mmol) and after flash chromatography on silica gel (*n*-hexane/EtOAc 5:1 to 1:1 to 0:1) and washing with hot ethanol (2.00 mL), compound **5e** (18.0 mg, 50.0 µmol, 2%) was obtained as orange solid. Mp 230-234 °C.

^{1}H NMR (300 MHz, $CDCl_3$): δ 1.38 (t, *J* = 7.1 Hz, 3 H), 4.30 (q, *J* = 7.1 Hz, 2 H), 7.08 (dd, *J* = 1.6, 1.8 Hz, 1 H), 7.42 (dd, *J* = 1.6, 1.8 Hz, 1 H), 7.56-7.64 (m, 3 H), 7.76-7.85 (m, 6 H), 14.57 (s, 1 H). ^{13}C NMR (75 MHz, $CDCl_3$): δ 14.7 (CH_3), 60.7 (CH_2), 63.8 (C_{quat}), 108.8 (CH), 114.2 (C_{quat}), 116.2 (CH), 118.2 (C_{quat}), 119.4 (C_{quat}), 126.1 (CH), 128.0 (CH), 130.1 (CH), 131.7 (CH), 132.2 (C_{quat}), 133.2 (CH), 141.4 (C_{quat}), 146.8 (C_{quat}), 150.6 (C_{quat}), 156.0 (C_{quat}), 170.9 (C_{quat}). EI-MS (70 eV, *m/z* (%)): 368 (12), 367 ($[M]^+$, 48), 296 (28), 295 ($[M - C_3H_4O_2]^+$, 294 ($[M - C_3H_5O_2]^+$, 293 (16), 270 (12), 265 ($[M - C_7H_4N]^+$, 12). IR (ATR): $\tilde{\nu}$ [cm^{-1}] 3092 (w), 3042 (w), 2988 (w), 2922 (w), 2851 (w), 2229 (w), 2210 (m), 2197(m), 1624 (m), 1597 (s), 1578 (m), 1501 (m), 1489 (m), 1462 (w), 1410 (m), 1393 (m), 1366 (m), 1310 (s), 1292 (s), 1281 (m), 1258 (m), 1173 (m), 1092 (m), 1078 (m), 1047 (m), 1032 (m), 1003 (m), 982 (m), 874 (m), 849 (m), 826 (m), 762 (s), 733 (w), 681 (m), 637 (m). UV/Vis (CH_2Cl_2): λ_{max} [nm] (ε [$L\cdot mol^{-1}\cdot cm^{-1}$]) = 283 (36000), 322 (15600), 434 (8600). Emission (CH_2Cl_2): λ_{max} [nm] (Stokes shift [cm^{-1}]) = 579 (5800); quantum yield (CH_2Cl_2): Φ_f = 0.01. HRMS (ESI): *m/z* calcd for [$C_{23}H_{17}N_3O_2^+$]: 368.1399; Found: 368.1399.

2.5.6 Ethyl (*Z*)-2-cyano-2-[6-(4-methoxyphenyl]-4-[4-(trifluoromethyl)phenyl]pyridin-2(1*H*)-ylidene)acetate (5f)

According to GP3 using 1-(4-methoxyphenyl)-3-[4-(trifluoromethyl)phenyl]prop-2-yn-1-one (**3k**, 456 mg, 1.50 mmol), sodium carbonate (129 mg, 1.20 mmol), sodium acetate (75.0 mg, 90.0 µmol) and ethyl cyanoacetate (**4**, 693 mg, 6.00 mmol) and after flash chromatography on silica gel (*n*-hexane/EtOAc 5:1 to 1:1) and washing with hot ethanol (10.0 mL), compound **5f** (242 mg, 550 µmol, 37%) was obtained as yellow solid. Mp 214-226 °C.

^{1}H NMR (300 MHz, CD_2Cl_2): δ 1.38 (t, *J* = 7.1 Hz, 3 H), 3.90 (s, 3 H), 4.30 (q, *J* = 7.1 Hz, 2 H), 7.06 (dd, *J* = 1.6, 1.7 Hz, 1 H), 7.07-7.17 (m, 2 H), 7.37 (dd, *J* = 1.6, 1.7 Hz, 1 H), 7.72-7.87 (m, 6 H), 14.52 (s, 1 H). ^{13}C NMR (150 MHz, CD_2Cl_2): δ 14.8 (CH_3), 55.8 (CH_3), 60.5 (CH_2), 63.1 (C_{quat}), 108.3 (CH), 115.0 (CH), 115.5 (CH), 119.7 (C_{quat}), 123.9 (q, $J_{C\text{-}F}$ = 272.5 Hz, C_{quat}), 124.4 (C_{quat}), 126.4 (q, $J_{C\text{-}F}$ = 3.5 Hz, CH), 127.6 (CH), 127.8 (CH), 132.3 (q, $J_{C\text{-}F}$ = 32.7 Hz, C_{quat}), 140.8 (C_{quat}), 146.4 (C_{quat}), 151.3 (C_{quat}), 155.8 (C_{quat}), 162.4 (C_{quat}), 171.0 (C_{quat}). EI-MS (70 eV, *m/z* (%)): 441 (24), 440 ([M]$^+$, 100), 417 (21), 406 (12), 395 ([M - C_2H_5O]$^+$, 24), 394 (55), 372 (16), 369 (32), 368 ([M - $C_3H_4O_2$]$^+$, 79), 351 (15), 345 ([M - $C_3H_8O_2F$]$^+$, 28), 344 (11), 343 (36), 325 ([M - $C_3H_6OF_3$]$^+$, 11), 323 (22). IR (ATR): $\tilde{\nu}$ [cm^{-1}] 3102 (w), 2986 (w), 2945 (w), 2911 (w), 2193 (m), 1722 (w), 1610 (m), 1597 (m), 1574 (m), 1518 (m), 1493 (w), 1416 (w), 1395 (w), 1368 (w), 1325 (m), 1310 (m), 1285 (s), 1261 (w), 1246 (m), 1198 (m), 1171 (m), 1105 (s), 1096 (s), 1070 (s), 1043 (m), 1024 (m), 1015 (m), 988 (m), 878 (w), 831 (s), 826 (s), 800 (m), 764 (w), 725 (w), 638 (w). UV/Vis (CH_2Cl_2): λ_{max} [nm] (ε [L·mol^{-1}·cm^{-1}]) = 261 (26200), 306 (28400), 429 (10400). Emission (CH_2Cl_2): λ_{max} [nm] (Stokes shift [cm^{-1}]) = 557 (5400); quantum yield (CH_2Cl_2): Φ_f = 0.02. Anal. calcd. for $C_{24}H_{19}F_3N_2O_3$ (440.1): C 65.45, H 4.35, N 6.36; Found: C 65.41, H 4.44, N 6.00.

2.5.7 Ethyl (*Z*)-2-cyano-2-{4-(4-methoxyphenyl)-6-[4-(trifluoromethyl)phenyl]pyridin-2(1*H*)-ylidene}acetate (5g)

According to GP3 using 3-(4-methoxyphenyl)-1-[4-(trifluoromethyl)phenyl]prop-2-yn-1-one (**3l**, 456 mg, 1.50 mmol), sodium carbonate (129 mg, 1.20 mmol), sodium acetate (75.0 mg, 90.0 µmol) and ethyl cyanoacetate (**4**, 693 mg, 6.00 mmol) and after flash chromatography on silica gel (*n*-hexane/EtOAc 5:1 to 1:1) and washing with hot ethanol (7.00 mL), compound **5g** (263 mg, 600 µmol, 40%) was obtained as yellow solid. Mp 200-202 °C.

^{1}H NMR (300 MHz, CD_2Cl_2): δ 1.37 (t, *J* = 7.1 Hz, 3 H), 3.89 (s, 3 H), 4.29 (q, *J* = 7.1 Hz, 2 H), 6.99-7.07 (m, 2 H), 7.16 (dd, *J* = 1.6, 1.8 Hz, 1 H), 7.45 (dd, *J* = 1.6, 1.8 Hz, 1 H), 7.64-7.71 (m, 2 H), 7.81-7.95 (m, 4 H), 14.54 (s, 1 H). ^{13}C NMR (75 MHz, CD_2Cl_2): δ 14.7 (CH_3), 55.7 (CH_3), 60.6 (CH_2), 63.0 (C_{quat}), 109.8 (CH), 114.9 (CH), 115.1 (CH), 119.7 (C_{quat}), 126.6 (CH), 127.0 (q, $J_{C\text{-}F}$ = 3.7 Hz, CH), 128.6 (CH), 128.8 (C_{quat}), 133.3 (C_{quat}), 136.2 (C_{quat}), 144.2 (C_{quat}),

152.0 (C_{quat}), 156.0 (C_{quat}), 162.0 (C_{quat}), 171.2 (C_{quat}). The C_{quat} signal of the CF_3 group is superimposed by other signals. EI-MS (70 eV, *m/z* (%)): 441 (14), 440 ($[M]^+$, 51), 395 ($[M - C_2H_5O]^+$, 14), 394 (20), 369 (27), 368 ($[M - C_3H_4O_2]^+$, 100), 343 (19), 325 (12), 323 (12), 152 ($[M - C_{17}H_{11}F_3O]^+$, 11), 132 ($[M - C_{15}H_{11}F_3N_2O_2]^+$, 10). IR (ATR): $\tilde{\nu}$ [cm^{-1}] 3030 (w), 2941 (w), 2845 (w), 2187 (m), 1628 (m), 1601 (m), 1578 (m), 1521 (m), 1485 (w), 1441 (w), 1400 (w), 1331 (m), 1300 (m), 1287 (m), 1244 (m), 1173 (m), 1126 (m), 1098 (m), 1072 (m), 1042 (m), 1032 (w), 1017 (w), 988 (w), 861 (w), 826 (s), 768 (w). UV/Vis (CH_2Cl_2): λ_{max} [nm] (ε [$L \cdot mol^{-1} \cdot cm^{-1}$]) = 260 (20300), 324 (43800), 420 (10000). Emission (CH_2Cl_2): λ_{max} [nm] (Stokes shift [cm^{-1}]) = 562 (6000); quantum yield (CH_2Cl_2): Φ_f = 0.02. Anal. calcd. for $C_{24}H_{19}F_3N_2O_3$ (440.1): C 65.45, H 4.35, N 6.36; Found: C 65.64, H 4.46, N 6.24.

2.5.8 Ethyl (*Z*)-2-cyano-2-[4-phenyl-6-(thiophen-2-yl)pyridin-2(1*H*)-ylidene]acetate (5h)

According to GP3 using 3-phenyl-1-(thiophen-2-yl)prop-2-in-1-one (**3n**, 193 mg, 910 µmol), sodium carbonate (78.0 mg, 730 µmol), sodium acetate (46.0 mg, 550 µmol) and ethyl cyano-acetate (**4**, 420 mg, 3.64 mmol) and after flash chromatography on silica gel (*n*-hexane/EtOAc 5:1 to 1:1 to 0:1) and washing with hot ethanol (10.0 mL), compound **5h** (161 mg, 460 µmol, 51%) was obtained as orange solid. Mp 192-201 °C.

^1H NMR (300 MHz, $CDCl_3$): δ 1.36 (t, *J* = 7.1 Hz, 3 H), 4.29 (q, *J* = 7.1 Hz, 2 H), 7.14 (t, *J* = 1.8 Hz, 1 H), 7.25 (dd, *J* = 5.0, 3.7 Hz, 1 H), 7.33 (t, *J* = 1.6 Hz, 1 H), 7.50-7.55 (m, 3 H), 7.60 (dd, *J* = 5.1, 1.1 Hz, 1 H), 7.70 (dd, *J* = 3.9, 1.1 Hz, 1 H), 7.71-7.75 (m, 2 H), 14.55 (s, 1 H). ^{13}C NMR (75 MHz, $CDCl_3$): δ 15.0 (CH_3), 60.9 (CH_2), 62.9 (C_{quat}), 109.1 (CH), 115.3 (CH), 119.6 (C_{quat}), 127.4 (CH), 127.7 (CH), 129.5 (CH), 129.8 (CH), 129.9 (CH), 131.0 (CH), 135.9 (C_{quat}), 137.3 (C_{quat}), 140.7 (C_{quat}), 153.1 (C_{quat}), 156.0 (C_{quat}), 171.4 (C_{quat}). EI-MS (70 eV, *m/z* (%)): 349 (21), 348 ($[M]^+$, 84), 303 ($[M-C_2H_5O]^+$, 26), 302 (51), 277 ($[M-C_3H_5NO]^+$, 31), 276 (100), 275 ($[M-C_3H_5O_2]^+$, 17), 274 (15), 251 (16), 248 (12), 246 ($[M-C_8H_6]^+$, 84), 164 (9). IR (ATR): $\tilde{\nu}$ [cm^{-1}] 3111 (w), 2984 (w), 2924 (w), 2907 (w), 2872 (w), 2195 (m), 1622 (m), 1585 (s), 1578 (s), 1558 (w), 1501 (w), 1489 (m), 1443 (m), 1427 (w), 1404 (w), 1391 (w), 1366 (m), 1308 (s), 1279 (s), 1261 (m), 1250 (m), 1175 (m), 1161 (m), 1098 (s), 1082 (m), 1063 (m), 1038 (m), 995 (w), 980 (m), 934 (w), 901 (w), 874 (w), 856 (m), 841 (w), 829 (w), 795 (w), 768 (s), 750 (m), 720 (s), 696 (m), 615 (w). UV/Vis (CH_2Cl_2): λ_{max} [nm] (ε [$L \cdot mol^{-1} \cdot cm^{-1}$]) = 273 (21800), 308 (26700), 433 (9200). Emission (CH_2Cl_2): λ_{max} [nm] (Stokes-shift [cm^{-1}]) = 560 (5200); quantum yield (CH_2Cl_2): Φ_f = 0.03. Anal. calcd. for $C_{20}H_{16}N_2O_2S$ (348.1): C 68.95, H 4.63, N 8.04, S 9.20; Found: C 68.82, H 4.79, N 7.85, S 9.05.

2.5.9 6-(4-Methoxyphenyl)-2-oxo-4-phenyl-2*H*-pyran-3-carbonitrile (6b)

According to GP3 using 1-(4-methoxyphenyl)-3-phenylprop-2-yn-1-one (**3b**, 118 mg, 500 µmol), sodium carbonate (43.0 mg, 400 µmol), sodium acetate (25.0 mg, 300 µmol) and ethyl cyanoacetate (**4**, 231 mg, 2.00 mmol) and after flash chromatography on silica gel (*n*-hexane/EtOAc 5:1 to 1:1) and washing with hot ethanol (2.00 mL), compound **6b** (106 mg, 350 µmol, 70%) was obtained as bright yellow solid. Mp 225-230 °C.

^{1}H NMR (300 MHz, $CDCl_3$): δ 3.90 (s, 3 H), 6.81 (s, 1 H), 6.97-7.04 (m, 2 H), 7.51-7.62 (m, 3 H), 7.69-7.75 (m, 2 H), 7.85-7.94 (m, 2 H). ^{13}C NMR (75 MHz, $CDCl_3$): δ 56.2 (CH_3), 94.5 (C_{quat}), 103.7 (CH), 115.2 (CH), 115.8 (C_{quat}), 126.9 (C_{quat}), 127.0 (CH), 129.8 (CH), 130.7 (CH), 130.8 (C_{quat}), 133.1 (CH), 160.1 (C_{quat}), 163.2 (C_{quat}), 163.5 (C_{quat}), 163.7 (C_{quat}). EI-MS (70 eV, *m/z* (%)): 304 (19), 303 ([M]$^+$, 82), 300 (17), 276 (20), 275 ([M - CO]$^+$, 100), 260 ([M - CO_2]$^+$, 20), 232 (19), 221 ([M - C_3NO_2]$^+$, 14), 135 (36), 107 ([M - $C_{12}H_6NO_2$]$^+$, 11), 77 ([M - $C_{13}H_8NO_3$]$^+$, 15). IR (ATR): $\tilde{\nu}$ [cm^{-1}] 2965 (w), 2901 (w), 2868 (w), 2839 (w), 2361 (w), 2311 (w), 2220 (w), 1717 (m), 1622 (w), 1603 (m), 1574 (m), 1522 (m), 1496 (m), 1494 (m), 1456 (m), 1443 (w), 1423 (w), 1385 (w), 1352 (w), 1303 (m), 1261 (m), 1248 (m), 1190 (m), 1157 (m), 1123 (w), 1080 (m), 1055 (m), 1032 (m), 1016 (m), 1001 (w), 947 (w), 835 (s), 814 (w), 775 (s), 745 (w), 692 (s). UV/Vis (CH_2Cl_2): λ_{max} [nm] (ε [L·mol^{-1}·cm^{-1}]) = 254 (10300), 271 (11500), 312 (9600), 404 (21900). Emission (solid): λ_{max} [nm] = 540. Anal. calcd. for $C_{19}H_{13}NO_3$ (303.1): C 75.24, H 4.32, N 4.62; Found: C 75.29, H 4.27, N 4.59.

2.5.10 6-[4-(Dimethylamino)phenyl]-2-oxo-4-phenyl-2*H*-pyran-3-carbonitrile (6c)

According to GP3 using 1-[4-(dimethylamino)phenyl]-3-phenylprop-2-yn-1-one (**3c**, 249 mg, 1.00 mmol), sodium carbonate (86.0 mg, 800 µmol), sodium acetate (50.0 mg, 600 µmol) and ethyl cyanoacetate (**4**, 462 mg, 4.00 mmol) and after flash chromatography on silica gel (*n*-hexane/EtOAc 5:1 to 1:1 to 0:1) and washing with hot ethanol (2.00 mL), compound **6c** (37.0 mg, 120 µmol, 12%) was obtained as deep purple solid. Mp 224-253 °C.

^{1}H NMR (300 MHz, $CDCl_3$): δ 3.10 (s, 6 H), 6.69 (s, 1 H), 6.69-6.75 (m, 2 H), 7.51-7.58 (m, 3 H), 7.67-7.73 (m, 2 H), 7.78-7.85 (m, 2 H). ^{13}C NMR (75 MHz, $CDCl_3$): δ 40.2 (CH_3), 91.1 (C_{quat}), 100.1 (CH), 111.8 (CH), 115.7 (C_{quat}), 116.5 (C_{quat}), 128.0 (CH), 128.8 (CH), 129.3 (CH), 131.6 (CH), 135.3 (C_{quat}), 153.4 (C_{quat}), 160.3 (C_{quat}), 164.1 (C_{quat}), 164.9 (C_{quat}). EI-MS (70 eV, *m/z* (%)): 317 (15), 316 ([M]$^+$, 66), 293 (11), 289 ([M - CN]$^+$, 11), 288 ([M - CO]$^+$, 51), 287 (19), 167 ([M - $C_9H_{11}NO$]$^+$, 18), 150 (11), 149 ([M - $C_{11}H_5NO$]$^+$, 100), 148 (20), 144 (13), 127 ([M - $C_{11}H_{11}NO_2$]$^+$, 12), 85 (13), 71 (22), 57 (18), 43 ([M - $C_{18}H_{11}NO_2$]$^+$, 13). IR (ATR): $\tilde{\nu}$ [cm^{-1}] 3092 (w), 3048 (w), 2901 (w), 2864 (w), 2812 (w), 2739 (w), 2212 (w), 1708 (m), 1706 (m), 1609 (m), 1589 (m), 1570 (m), 1530 (m), 1497 (m), 1491 (m), 1482 (m), 1478 (m), 1473 (m), 1467 (m), 1458 (m), 1433 (m), 1375 (m), 1360 (m), 1333 (m), 1252 (m), 1209 (m), 1171 (m), 1159

(m), 1125 (m), 1111 (m), 1082 (m), 1059 (m), 1020 (m), 995 (m), 953 (m), 945 (m), 924 (w), 853 (m), 818 (s), 795 (m), 750 (m), 748 (m), 692 (s), 669 (m), 640 (m). UV/Vis (CH_2Cl_2): λ_{max} [nm] (ε [L·mol^{-1}·cm^{-1}]) = 294 (18800), 482 (47300). Emission (CH_2Cl_2): λ_{max} [nm] (Stokes-shift [cm^{-1}]) = 567 (3100); quantum yield (CH_2Cl_2): Φ_f = 0.99; emission (solid): λ_{max} [nm] = 694; quantum yield (solid): Φ_f = 0.11. Anal. calcd. for $C_{20}H_{16}N_2O_2$ (316.1): C 75.93, H 5.10, N 8.86; Found: C 75.74, H 5.19, N 8.56.

2.5.11 4-(4-Methoxyphenyl)-2-oxo-6-phenyl-2*H*-pyran-3-carbonitrile (6d)

According to GP3 using 3-(4-methoxyphenyl)-1-phenylprop-2-yn-1-one (**3f**, 945 mg, 4.00 mmol), sodium carbonate (344 mg, 3.20 mmol), sodium acetate (200 mg, 2.40 mmol) and ethyl cyanoacetate (**4**, 1.85 g, 16.0 mmol) and after flash chromatography on silica gel (*n*-hexane/EtOAc 5:1 to 0:1) and washing with hot ethanol (20.0 mL), compound **6d** (993 mg, 3.72 mmol, 82%) was obtained as yellow solid. Mp 227-238 °C.
^{1}H NMR (300 MHz, $CDCl_3$): δ 3.91 (s, 3 H), 6.93 (s, 1 H), 7.04-7.10 (m, 2 H), 7.48-7.60 (m, 3 H), 7.74-7.79 (m, 2 H), 7.90-7.94 (m, 2 H). ^{13}C NMR (75 MHz, $CDCl_3$): δ 55.8 (CH_3), 94.1 (C_{quat}), 103.0 (CH), 114.9 (CH), 115.3 (C_{quat}), 126.4 (C_{quat}), 126.7 (CH), 129.5 (CH), 130.3 (CH), 132.7 (CH), 159.6 (C_{quat}), 162.9 (C_{quat}), 163.1 (C_{quat}), 163.2 (C_{quat}). EI-MS (70 eV, *m/z* (%)): 304 (22), 303 ([M]$^+$, 83), 276 (22), 275 ([M - CO]$^+$, 100), 232 (15), 221 ([M - C_3NO_2]$^+$, 19), 204 (11), 170 (12), 127 (7), 105 (53), 77 ([M - $C_{13}H_8NO_3$]$^+$, 34). IR (ATR): $\tilde{\nu}$ [cm^{-1}] 2988 (w), 2972 (w), 2922 (w), 2901 (w), 2884 (w), 2849 (w), 2220 (w), 2197 (w), 1720 (m), 1717 (m), 1595 (m), 1580 (m), 1492 (s), 1489 (s), 1472 (m), 1456 (m), 1443 (m), 1431 (m), 1408 (w), 1385 (m), 1356 (m), 1317 (m), 1304 (m), 1271 (s), 1246 (m), 1206 (m), 1184 (s), 1167 (m), 1121 (m), 1076 (m), 1049 (m), 1022 (m), 999 (w), 988 (w), 961 (w), 949 (w), 930 (w), 891 (w), 839 (m), 826 (s), 758 (s), 731 (w), 692 (s), 673 (m), 642 (m). UV/Vis (CH_2Cl_2): λ_{max} [nm] (ε [L·mol^{-1}·cm^{-1}]) = 258 (19700), 358 (30400); emission (solid): λ_{max} [nm] = 489. Anal. calcd. for $C_{19}H_{13}NO_3$ (303.1): C 75.24, H 4.32, N 4.62; Found: C 74.98, H 4.29, N 4.44.

2.5.12 4-[4-(Dimethylamino)phenyl]-2-oxo-6-phenyl-2*H*-pyran-3-carbonitrile (6e)

According to GP3 using 3-[4-(dimethylamino)phenyl]-1-phenylprop-2-yn-1-one (**3g**, 324 mg, 1.30 mmol), sodium carbonate (112 mg, 1.04 mmol), sodium acetate (65.0 mg, 780 µmol) and ethyl cyanoacetate (**5**, 600 mg, 5.20 mmol) and after flash chromatography on silica gel (*n*-hexane/EtOAc 1:1 to 0:1) and washing with hot ethanol (5.00 mL), compound **6e** (255 mg, 810 µmol, 62%) was obtained as red solid. Mp 190-200 °C.
^{1}H NMR (300 MHz, $CDCl_3$): δ 3.11 (s, 6 H), 6.82-6.91 (m, 2 H), 6.95 (s, 1 H), 7.45-7.59 (m, 3 H), 7.76-7.84 (m, 2 H), 7.87-7.97 (m, 2 H). ^{13}C NMR (75 MHz, $CDCl_3$): δ 40.6 (CH_3), 91.4 (C_{quat}), 102.6 (CH), 112.5 (CH), 116.3 (C_{quat}), 121.4 (C_{quat}), 126.5 (CH), 129.4 (CH), 130.4 (CH),

130.6 (C_{quat}), 132.3 (CH), 152.7 (C_{quat}), 160.4 (C_{quat}), 161.9 (C_{quat}), 162.2 (C_{quat}). EI-MS (70 eV, *m/z* (%)): 317 (22), 316 ($[M]^+$, 100), 215 (21), 288 ($[M - CO]^+$, 20), 287 (14), 144 (11), 105 (48), 77 ($[M - C_{14}H_{11}N_2O_2]^+$, 21). IR (ATR): $\tilde{\nu}$ [cm^{-1}] 2980 (w), 2903 (w), 2191 (m), 1638 (m), 1624 (s), 1599 (m), 1578 (m), 1566 (m), 1495 (m), 1477 (w), 1410 (w), 1389 (m), 1368 (m), 1308 (s), 1283 (s), 1252 (m), 1167 (m), 1157 (m), 1093 (m), 1080 (m), 1045 (m), 1032 (m), 1003 (m), 984 (m), 968 (m), 901 (w), 876 (w), 860 (w), 824 (s), 768 (s), 737 (m), 691 (s), 637 (m), 617 (w). UV/Vis (CH_2Cl_2): λ_{max} [nm] (ε [$L \cdot mol^{-1} \cdot cm^{-1}$]) = 255 (25600), 289 (23300), 375 (39600), 453 (40600). Emission (CH_2Cl_2): λ_{max} [nm] (Stokes-shift [cm^{-1}]) = 634 (6300); quantum yield (CH_2Cl_2): Φ_f = 0.01. Anal. calcd. for $C_{20}H_{16}N_2O_2$ (316.1): C 75.93, H 5.10, N 8.86; Found: C 76.08, H 4.89, N 8.56.

2.5.13 4,6-Bis(4-methoxyphenyl)-2-oxo-2*H*-pyran-3-carbonitrile (6f)

According to GP3 using 1,3-bis(4-methoxyphenyl)prop-2-yn-1-one (**3j**, 932 mg, 3.50 mmol), sodium carbonate (301 mg, 2.80 mmol), sodium acetate (175 mg, 2.10 mmol) and ethyl cyanoacetate (**4**, 1.62 g, 14.0 mmol) and after flash chromatography on silica gel (*n*-hexane/EtOAc 2:1 to 1:1) and washing with hot ethanol (50.0 mL), compound **6f** (530 mg, 1.59 mmol, 45%) was obtained as yellow solid. Mp 236 °C.

^1H NMR (300 MHz, $CDCl_3$): δ 3.89 (s, 3 H), 3.90 (s, 3 H), 6.81 (s, 1 H), 6.79 -7.03 (m, 2 H), 7.04-7.09 (m, 2 H), 7.71-7.78 (m, 2 H), 7.85-7.92 (m, 2 H). ^{13}C NMR (75 MHz, CD_2Cl_2): δ 55.8 (CH_3), 92.7 (C_{quat}), 101.6 (CH), 114.85 (CH), 114.93 (CH), 115.5 (C_{quat}), 122.9 (C_{quat}), 126.7 (C_{quat}), 128.7 (CH), 130.2 (CH), 159.9 (C_{quat}), 162.9 (C_{quat}), 163.1 (C_{quat}), 163.36 (C_{quat}), 163.44 (C_{quat}). EI-MS (70 eV, *m/z* (%)): 334 (11), 333 ($[M]^+$, 47), 306 (16), 305 ($[M - CO]^+$,77), 262 ($[M - C_2HNO_2]^+$, 14), 135 ($[M - C_{12}H_8NO_2]^+$, 100), 107 ($[M - C_{13}H_8NO_3]^+$, 13), 92 ($[M - C_{14}H_{11}NO_3]^+$, 21), 77 ($[M - C_{14}H_{10}NO_4]^+$, 23). IR (ATR): $\tilde{\nu}$ [cm^{-1}] 3100 (w), 3080 (w), 3053 (w), 2938 (w), 2837 (w), 2220 (w), 1720 (s), 1717 (s), 1597 (s), 1578 (m), 1495 (s), 1489 (s), 1449 (m), 1418 (m), 1391 (m), 1360 (m), 1319 (w), 1302 (m), 1271 (s), 1246 (s), 1209 (m), 1182 (s), 1126 (m), 1051 (w), 1032 (s), 945 (w), 843 (m), 822 (s), 766 (m), 735 (w), 695 (w), 675 (m). UV/Vis (CH_2Cl_2): λ_{max} [nm] (ε [$L \cdot mol^{-1} \cdot cm^{-1}$]) = 254 (15000), 364 (25600), 400 (25100); emission (solid): λ_{max} [nm] = 526. Anal. calcd. for $C_{20}H_{15}NO_4$ (333.1): C 72.06, H 4.54, N 4.20; Found: C 71.98, H 4.57, N 4.11.

2.5.14 4-[4-(Dimethylamino)phenyl]-2-oxo-6-[4-(trifluormethyl)pheny]-2*H*-pyran-3-carbonitrile (6g)

According to GP1 using 3-[4-(dimethylamino)phenyl]-1-[4-(trifluoromethyl)phenyl]prop-2-yn-1-one (**3m**, 69.0 mg, 200 µmol), sodium carbonate (18.0 mg, 160 µmol), sodium acetate (10.0 mg, 120 µmol) and ethyl cyanoacetate (**4**, 93.0 mg, 800 µmol) and after chromatography

on silica gel (*n*-hexane/EtOAc 15:1 to 5:1 to 0:1) and washing with hot ethanol (2.00 mL), compound **6g** (54.6 mg, 140 µmol, 71%) was obtained as red solid. Mp 246-265 °C.

^{1}H NMR (300 MHz, $CDCl_3$): δ 3.11 (s, 6 H), 6.74-6.82 (m, 2 H), 7.02 (s, 1 H), 7.73-7.79 (m, 2 H), 7.79-7.86 (m, 2 H), 7.98-8.06 (m, 2 H). ^{13}C NMR (75 MHz, $CDCl_3$): δ 40.2 (CH_3), 91.8 (C_{quat}), 103.9 (CH), 111.8 (CH), 116.2 (C_{quat}), 119.8 (C_{quat}), 123.6 (q, $J_{C\text{-}F}$ = 273 Hz, C_{quat}), 126.3-126.6 (m, CH), 126.8 (CH), 130.6 (CH), 133.6 (q, $J_{C\text{-}F}$ = 33.0 Hz, C_{quat}), 133.9 (C_{quat}), 153.4 (C_{quat}), 159.5 (C_{quat}), 160.0 (C_{quat}), 161.7 (C_{quat}). EI-MS (70 eV, *m/z* (%)): 384 ($[M]^+$, 100), 383 (32), 183 ($[M\text{-}C_{11}H_{10}N_2O_2]^+$, 11), 173 (25), 145 ($[M\text{-}C_{14}H_{11}N_2O_2]^+$, 18). IR (ATR): $\tilde{\nu}$ [cm^{-1}] 3088 (w), 2922 (w), 2878 (w), 2864 (w), 2833 (w), 2814 (w), 2218 (w), 1701 (s), 1601 (m), 1574 (m), 1547 (m), 1516 (w), 1489 (m), 1466 (w), 1427 (w), 1414 (w), 1383 (m), 1352 (m), 1321 (s), 1258 (m), 1242 (m), 1223 (m), 1211 (m), 1196 (m), 1163 (s), 1138 (m), 1111 (s), 1069 (s), 1051 (m), 1034 (m), 1011 (m), 999 (m), 953 (m), 855 (m), 826 (s), 814 (s), 791 (w), 764 (m), 745 (m), 731 (w), 708 (w), 650 (w), 631 (w). UV/Vis (CH_2Cl_2): λ_{max} [nm] (ε [$L\cdot mol^{-1}\cdot cm^{-1}$]) = 251 (16200), 309 (13900), 372 (20200), 465 (21600). Emission (CH_2Cl_2): λ_{max} [nm] (Stokes shift [cm^{-1}]) = 673 (6600); quantum yield (CH_2Cl_2): Φ_f = <0.01. Anal. calcd. for $C_{21}H_{15}F_3N_2O_2$ (384.1): C 65.62, H 3.93, N 7.29; Found: C 65.45, H 3.90, N 7.18.

2.6 General procedure for the synthesis of 1*H*-pyridines 8 (GP4)

Alkynone **3** (1.00 equiv) was placed in a dry Schlenk tube and ethanol (1.00 mL, 0.500 M) was added. Sodium carbonate (43.0 mg, 400 µmol), sodium acetate (25.0 mg, 300 µmol), water (50.0 µL) and diethyl (*Z*)-3-amino-2-cyanopent-2-endioate (**7**, 226 mg, 1.00 mmol) were added and the mixture was stirred at 75 °C for 16 h. After the addition of CH_2Cl_2 (5.00 mL) and NaOH/$FeSO_4$ solution (5.00 mL), the solution was extracted with CH_2Cl_2 (3 × 50.0 mL). The combined organic layers were dried (anhydrous $MgSO_4$) and the solvent was removed in vacuo. The residue was purified by flash chromatography on silica gel (*n*-hexane/EtOAc 5:1 to 1:1 to 0:1) and washed with hot ethanol (5.00 mL).

2.6.1 Ethyl (*Z*)-2-(1-cyano-2-ethoxy-2-oxoethylidene)-4,6-diphenyl-1,2-dihydropyridine-3-carboxylate (8a)

According to GP4 using 1,3-diphenylprop-2-yn-1-one (**3a**, 103 mg, 500 µmol), compound **8a** (108 mg, 261 µmol, 52%) was obtained as yellow solid. Mp 140-146 °C.

^{1}H NMR (300 MHz, $CDCl_3$): δ 1.08 (t, *J* = 7.2 Hz, 3 H), 1.37 (t, *J* = 7.1 Hz, 3 H), 4.22 (q, *J* = 7.2 Hz, 2 H), 4.31 (q, *J* = 7.1 Hz, 2 H), 6.94 (d, *J* = 1.9 Hz, 1 H), 7.40-7.48 (m, 5 H), 7.55-7.62 (m, 3 H), 7.75-7.84 (m, 2 H), 15.60 (s, 1 H). ^{13}C NMR (75 MHz, $CDCl_3$): δ 13.7 (CH_3), 14.7 (CH_3), 60.9 (CH_2), 62.2 (CH_2), 62.7 (C_{quat}), 112.2 (CH), 118.2 (C_{quat}), 122.8 (C_{quat}), 126.3 (CH), 127.9 (CH), 128.8 (CH), 129.6 (CH), 130.1 (CH), 131.77 (CH), 131.84 (C_{quat}), 137.4 (C_{quat}), 146.1 (C_{quat}), 151.9 (C_{quat}), 153.0 (C_{quat}), 165.1 (C_{quat}), 172.1 (C_{quat}). EI-MS (70 eV, *m/z* (%)): 415 (26),

414 ([M]$^+$, 97), 369 ([M - C_2H_5O]$^+$, 18), 342 (28), 341 ([M - C_3H_5O]$^+$, 27), 340 ([M - C_3H_6O]$^+$, 12), 314 (20), 313 ([M - C_8H_5]$^+$, 57), 298 (24), 297 ([M - $C_5H_9O_3$]$^+$, 27), 296 ([M - $C_5H_{10}O_3$]$^+$, 33), 287 (10), 286 (25), 271 (24), 270 ([M - $C_6H_8O_4$]$^+$, 100), 269 (23), 268 ([M - $C_6H_{10}O_4$]$^+$, 21), 266 (12), 258 ([M - $C_7H_{10}NO_3$]$^+$, 14), 245 (18), 241 (13), 240 (26), 231 (15), 230 ([M - $C_8H_{10}NO_4$]$^+$, 34), 203 (19), 202 (31), 164 (13). IR (ATR): $\tilde{\nu}$ [cm^{-1}] 2978 (w), 2895 (w), 2197 (m), 1722 (m), 1636 (m), 1593 (s), 1578 (m), 1501 (m), 1489 (w), 1462 (w), 1441 (w), 1420 (w), 1364 (w), 1308 (m), 1288 (m), 1248 (s), 1188 (w), 1169 (m), 1134 (m), 1113 (s), 1092 (m), 1067 (m), 1047 (m), 1028 (w), 1001 (w), 885 (m), 854 (m), 847 (w), 775 (w), 758 (s), 746 (m), 694 (m), 658 (w). UV/Vis (CH_2Cl_2): λ_{max} [nm] (ε [L·mol^{-1}·cm^{-1}]) = 274 (20300), 324 (20100), 417 (7700). Emission (CH_2Cl_2): λ_{max} [nm] (Stokes shift [cm^{-1}]) = 557 (6000), quantum yield (CH_2Cl_2): Φ_f = <0.01. Anal. calcd. for $C_{25}H_{22}N_2O_4$ (414.2): C 72.45, H 5.35, N 6.76; Found: C 71.97, H 5.45, N 6.52.

2.6.2 Ethyl (*Z*)-2-(1-cyano-2-ethoxy-2-oxoethylidene)-6-(4-methoxyphenyl)-4-phenyl-1,2-dihydropyridine-3-carboxylate (8b)

According to GP2 using 1-(4-methoxyphenyl)-3-phenylprop-2-yn-1-one (**3b**, 118 mg, 500 µmol), compound **8b** (77.0 mg, 172 µmol, 34%) was obtained as yellow solid. Mp 115-120 °C.

^{1}H NMR (300 MHz, $CDCl_3$): δ 1.07 (t, *J* = 7.2 Hz, 3 H), 1.36 (t, *J* = 7.1 Hz, 3 H), 3.89 (s, 3 H), 4.20 (q, *J* = 7.2 Hz, 2 H), 4.30 (q, *J* = 7.1 Hz, 2 H), 6.88 (d, *J* = 1.9 Hz, 1 H), 7.03-7.12 (m, 2 H), 7.37-7.49 (m, 5 H), 7.72-7.78 (m, 2 H), 15.53 (s, 1 H). ^{13}C NMR (75 MHz, $CDCl_3$): δ 13.7 (CH_3), 14.7 (CH_3), 55.7 (CH_3), 60.9 (CH_2), 62.1 (CH_2), 62.3 (C_{quat}), 111.3 (CH), 115.5 (CH), 118.4 (C_{quat}), 121.8 (C_{quat}), 123.9 (C_{quat}), 127.8 (CH), 127.9 (CH), 128.7 (CH), 129.5 (CH), 137.6 (C_{quat}), 145.9 (C_{quat}), 151.6 (C_{quat}), 153.1 (C_{quat}), 162.5 (C_{quat}), 165.3 (C_{quat}), 172.2 (C_{quat}). EI-MS (70 eV, *m/z* (%)): 445 (27), 444 ([M]$^+$, 100), 399 ([M-C_2H_5O]$^+$, 16), 372 ([M-$C_3H_4O_2$]$^+$, 22), 371 ([M-$C_3H_5NO_4$]$^+$, 14), 370 ([M-$C_3H_7O_2$]$^+$, 14), 347 (12), 344 (12), 343 (43), 328 (20), 327 ([M-$C_{10}H_9NO_4$]$^+$, 24), 326 ([M-$C_{10}H_{10}NO_4$]$^+$, 44), 316 (17), 301 (25), 300 (94), 288 ([M-$C_7H_{10}NO_3$]$^+$, 12), 283 (13), 275 (27), 260 ([M-$C_8H_{10}O_2$]$^+$, 27), 256 (14), 228 (16), 189 (13). IR (ATR): $\tilde{\nu}$ [cm^{-1}] 2990 (w), 2980 (w), 2941 (w), 2906 (w), 2845 (w), 2191 (m), 1734 (m), 1639 (w), 1594 (m), 1585 (m), 1574 (m), 1520 (m), 1501 (m), 1487 (w), 1468 (w), 1443 (w), 1396 (w), 1369 (w), 1306 (s), 1294 (m), 1248 (s), 1236 (m), 1188 (m), 1152 (m), 1138 (m), 1109 (m), 1096 (m), 1043 (m), 1016 (m), 891 (m), 824 (m), 783 (m), 762 (m), 704 (m). UV/Vis (CH_2Cl_2): λ_{max} [nm] (ε [L·mol^{-1}·cm^{-1}]) = 261 (16200), 307 (31300), 419 (11000). Emission (CH_2Cl_2): λ_{max} [nm] (Stokes-shift [cm^{-1}]) = 565 (6200); quantum yield (CH_2Cl_2): Φ_f = <0.01. Anal. calcd. for $C_{26}H_{24}N_2O_5$ (444.2): C 70.26, H 5.44, N 6.30; Found: C 70.02, H 5.49, N 6.30.

3 ^{1}H and ^{13}C NMR spectra of 1*H*-pyridines 5

3.1 Ethyl (*Z*)-2-cyano-2-(4,6-diphenyl-1*H*-pyridin-2-ylidene)acetate (5a)

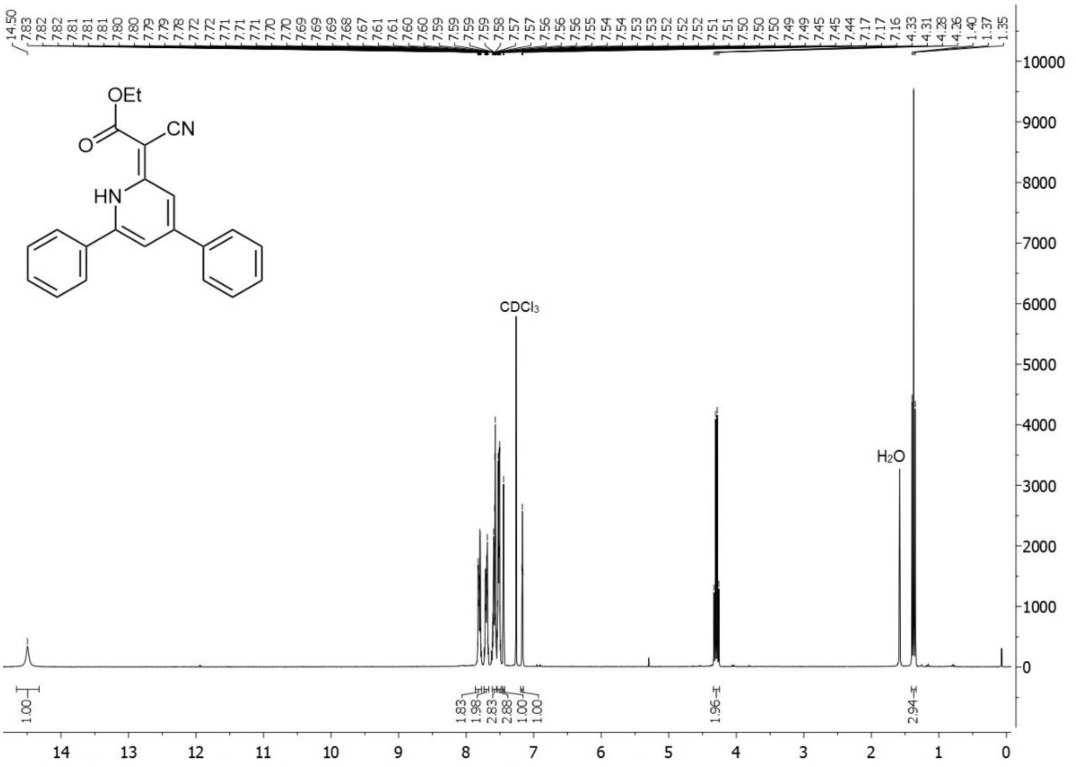

^{1}H NMR spectrum of compound **5a** ($CDCl_3$, 300 MHz, 293 K).

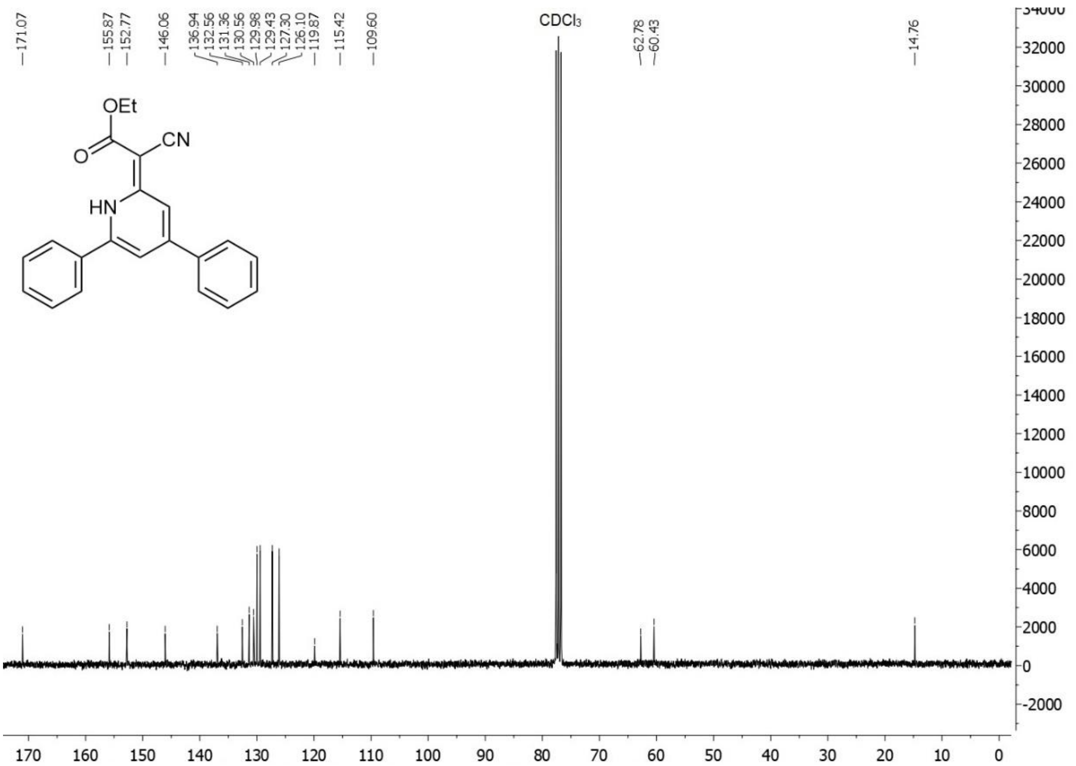

^{13}C NMR spectrum of compound **5a** ($CDCl_3$, 75 MHz, 293 K).

3.2 Ethyl (Z)-2-cyano-2-{4-phenyl-6-[4-(trifluoromethyl)phenyl]-1H-pyridin-2-ylidene}acetate (5b)

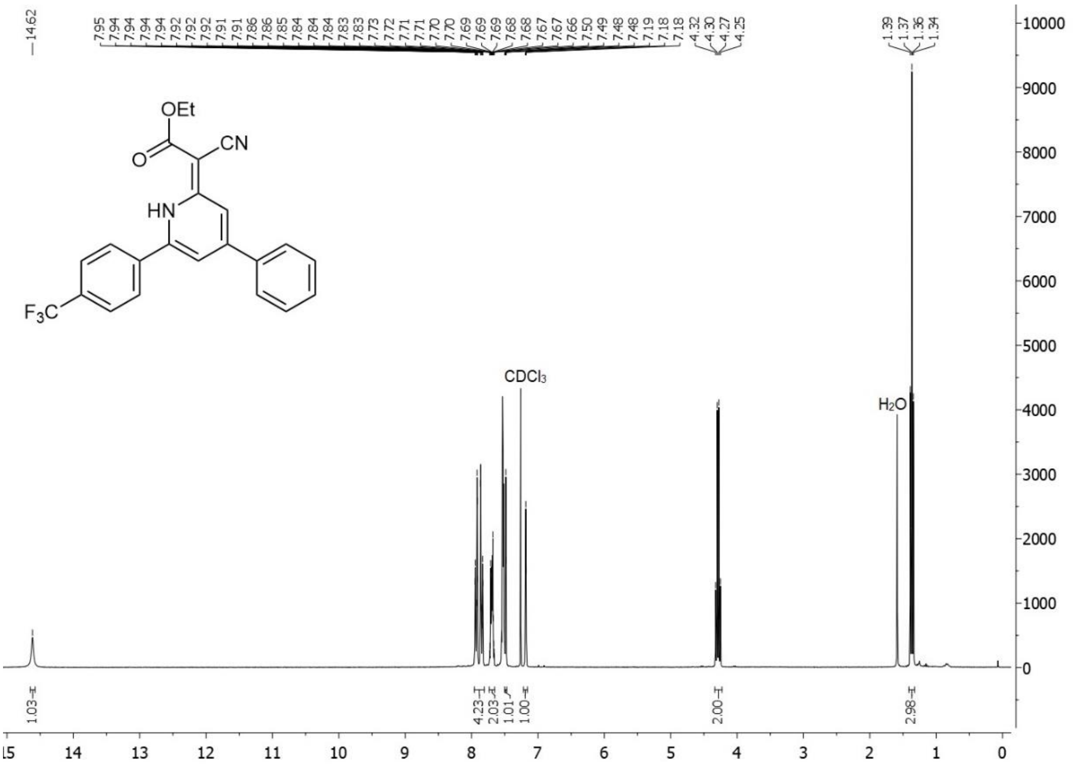

^{1}H NMR spectrum of compound **5b** ($CDCl_3$, 300 MHz, 293 K).

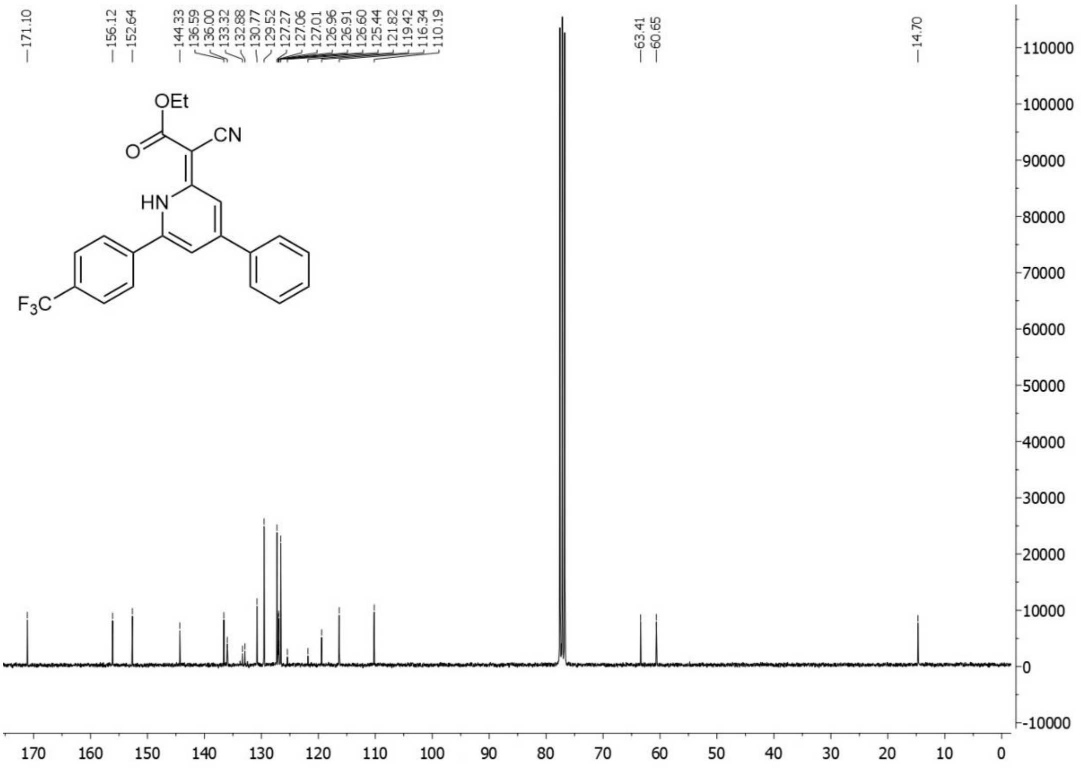

^{13}C NMR spectrum of compound **5b** ($CDCl_3$, 75 MHz, 293 K).

3.3 Ethyl (Z)-2-Cyano-2-[6-(4-cyanophenyl)-4-phenyl-1H-pyridin-2-ylidene]acetate (5c)

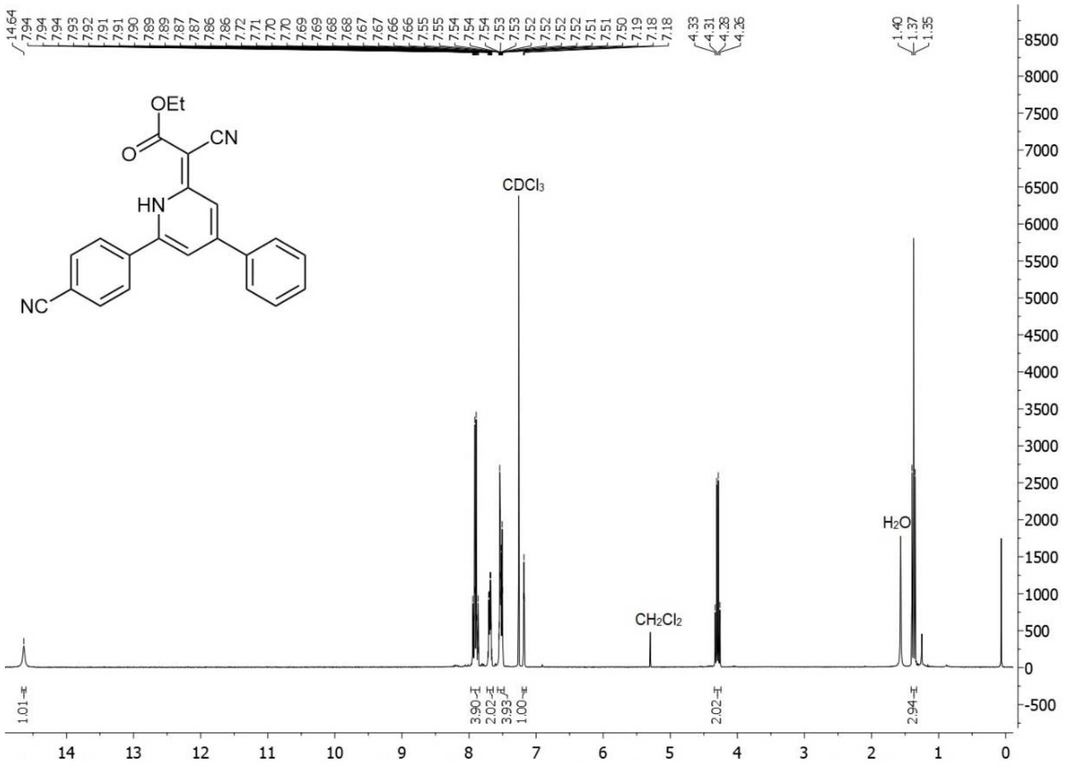

^{1}H NMR spectrum of compound **5c** ($CDCl_3$, 300 MHz, 293 K).

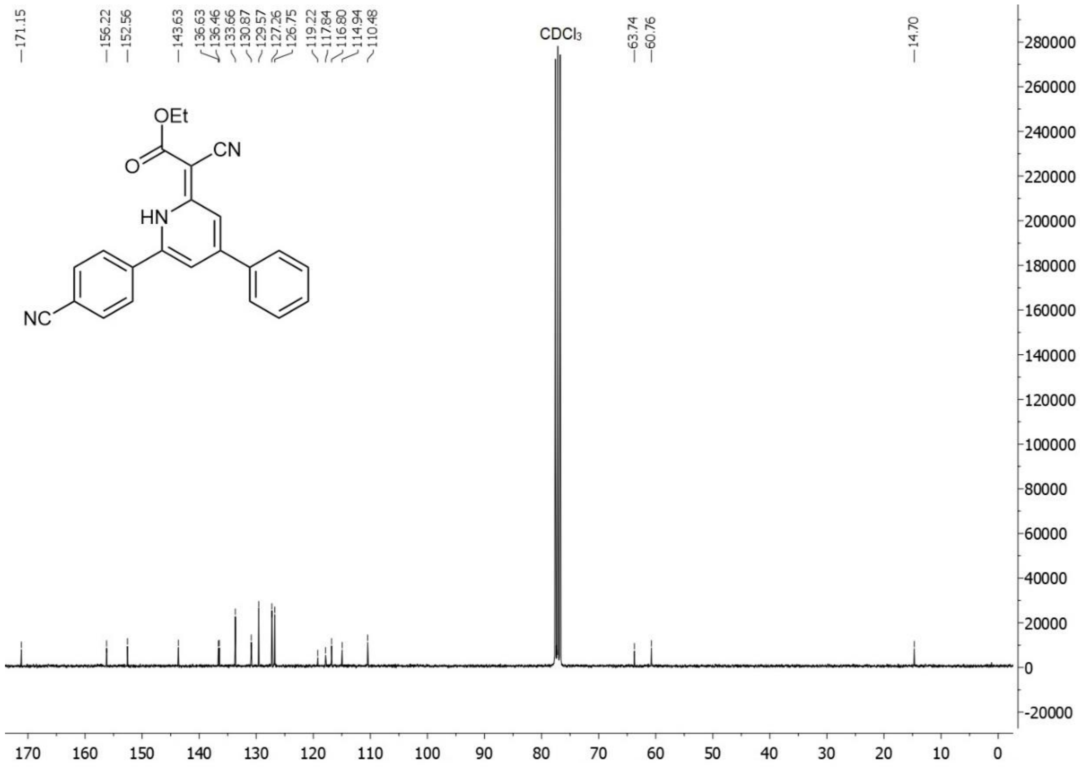

^{13}C NMR spectrum of compound **5c** ($CDCl_3$, 75 MHz, 293 K).

3.4 Ethyl (*Z*)-2-Cyano-2-{4-phenyl-6-[4-(trifluoromethyl)phenyl]-1*H*-pyridin-2-ylidene}acetate (5d)

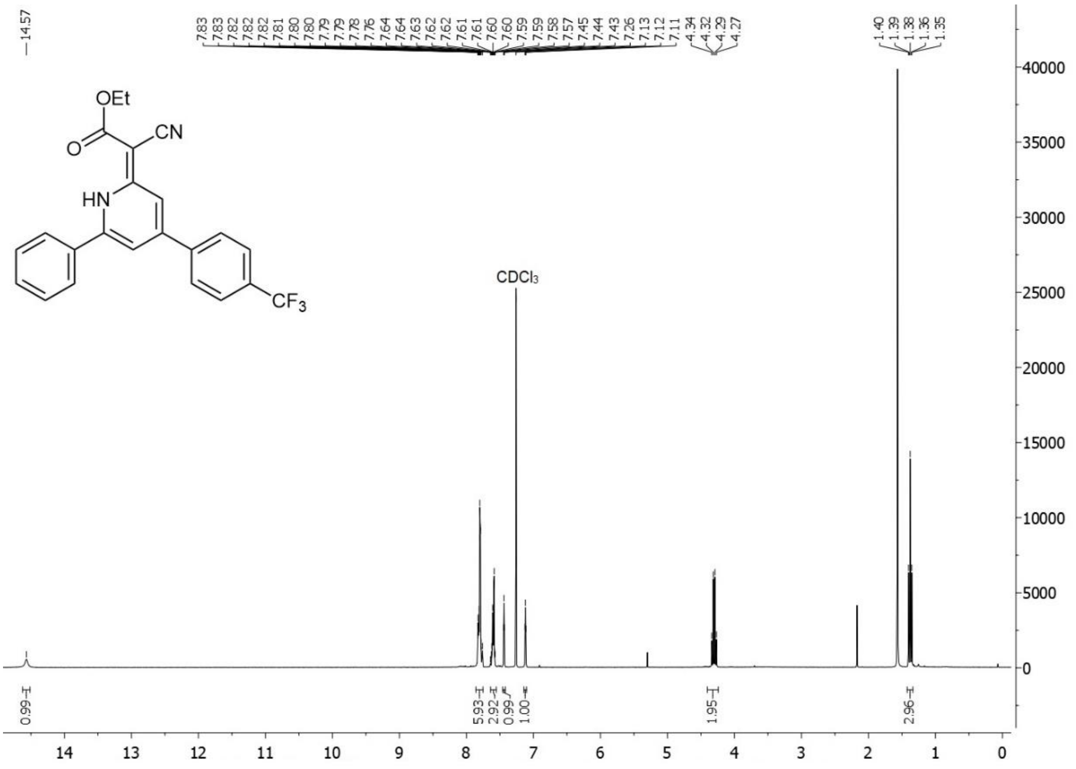

^{1}H NMR spectrum of compound **5d** ($CDCl_3$, 300 MHz, 293 K).

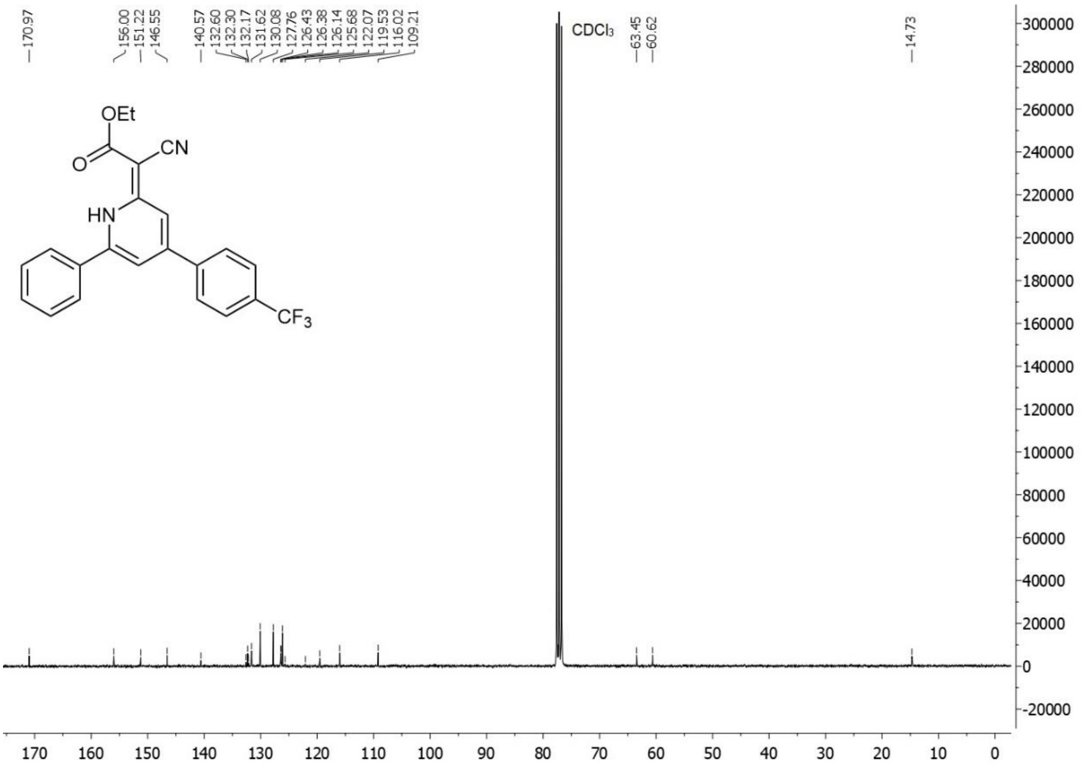

^{13}C NMR spectrum of compound **5d** ($CDCl_3$, 75 MHz, 293 K).

3.5 Ethyl (*Z*)-2-cyano-2-[6-(4-cyanophenyl)-4-phenyl-1*H*-pyridin-2-ylidene]acetate (5e)

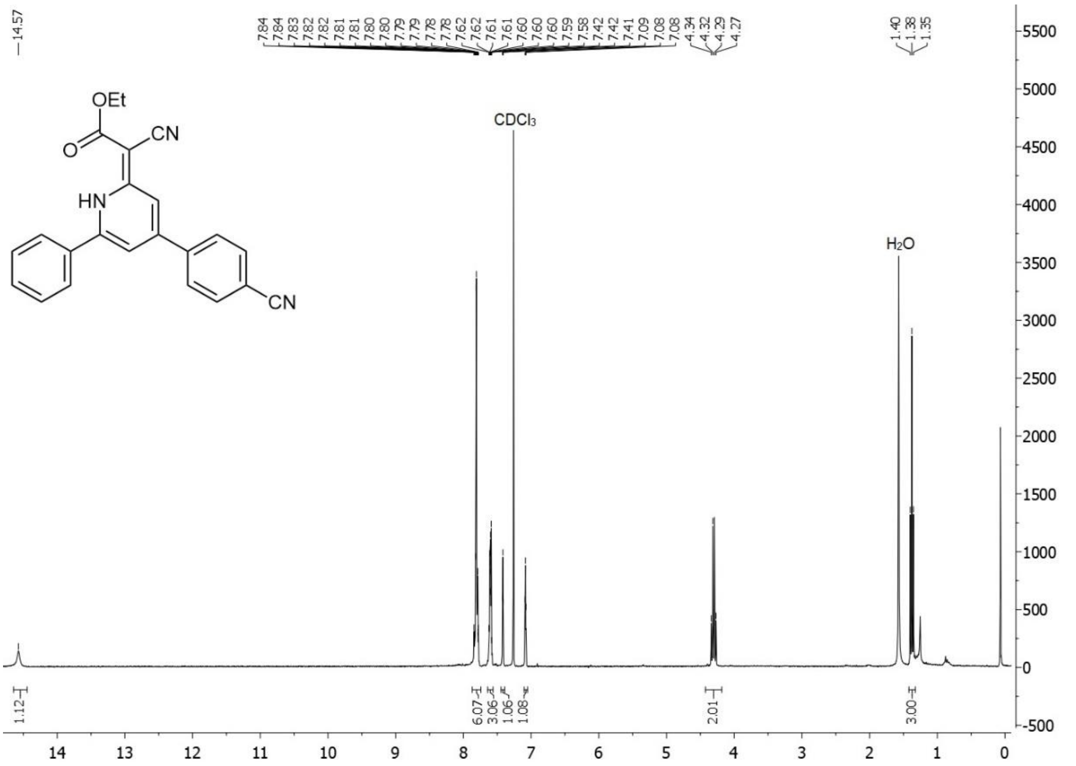

^{1}H NMR spectrum of compound **5e** ($CDCl_3$, 300 MHz, 293 K).

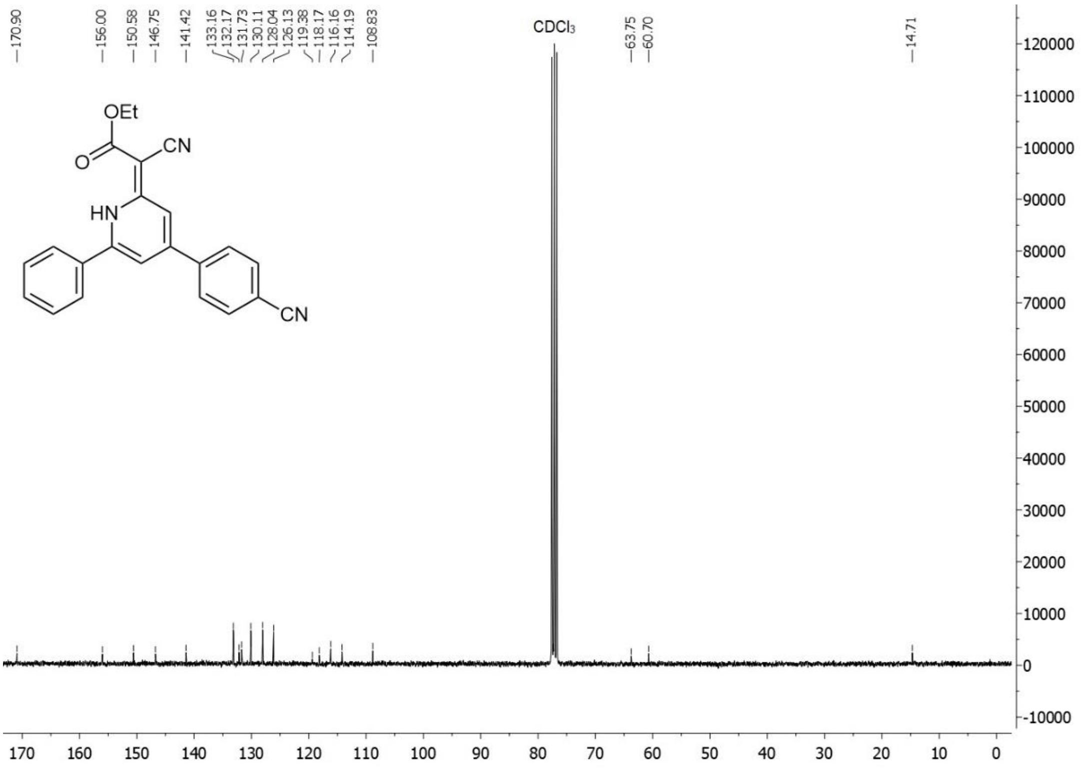

^{13}C NMR spectrum of compound **5e** ($CDCl_3$, 75 MHz, 293 K).

3.6 Ethyl (Z)-2-cyano-2-[4-(4-methoxyphenyl]-6-[4-(trifluoromethyl)phenyl]pyridin-2(1*H*)-ylidene)acetate (5f)

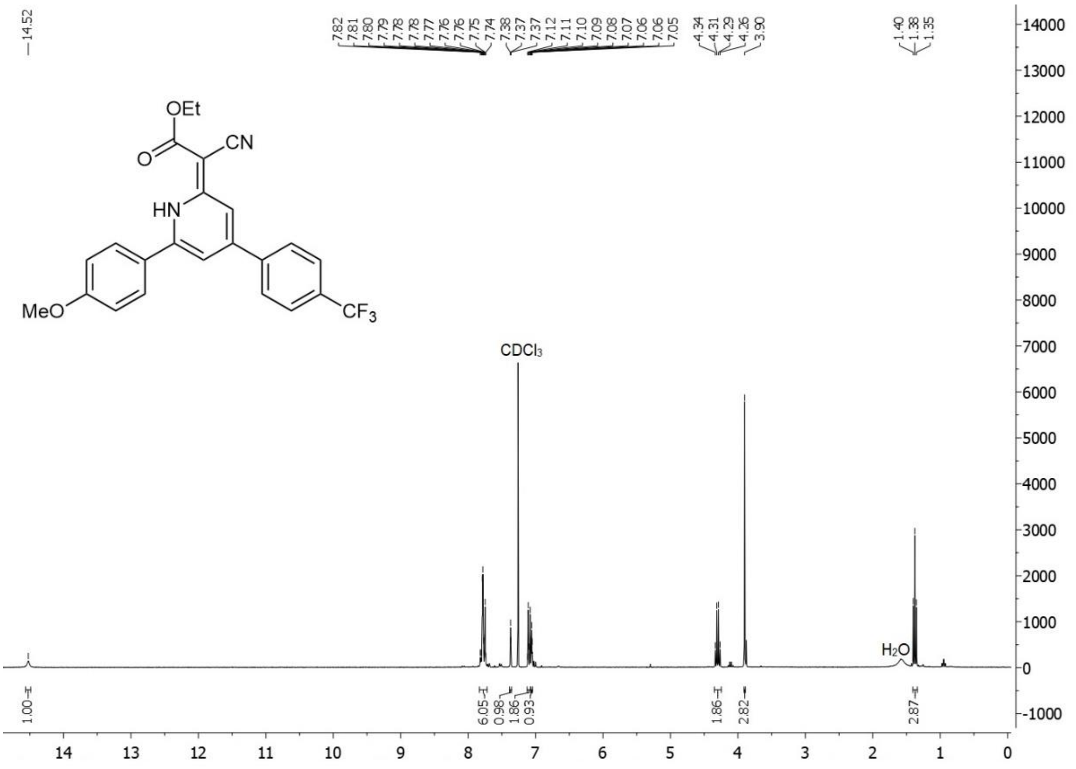

^{1}H NMR spectrum of compound **5f** ($CDCl_3$, 300 MHz, 293 K).

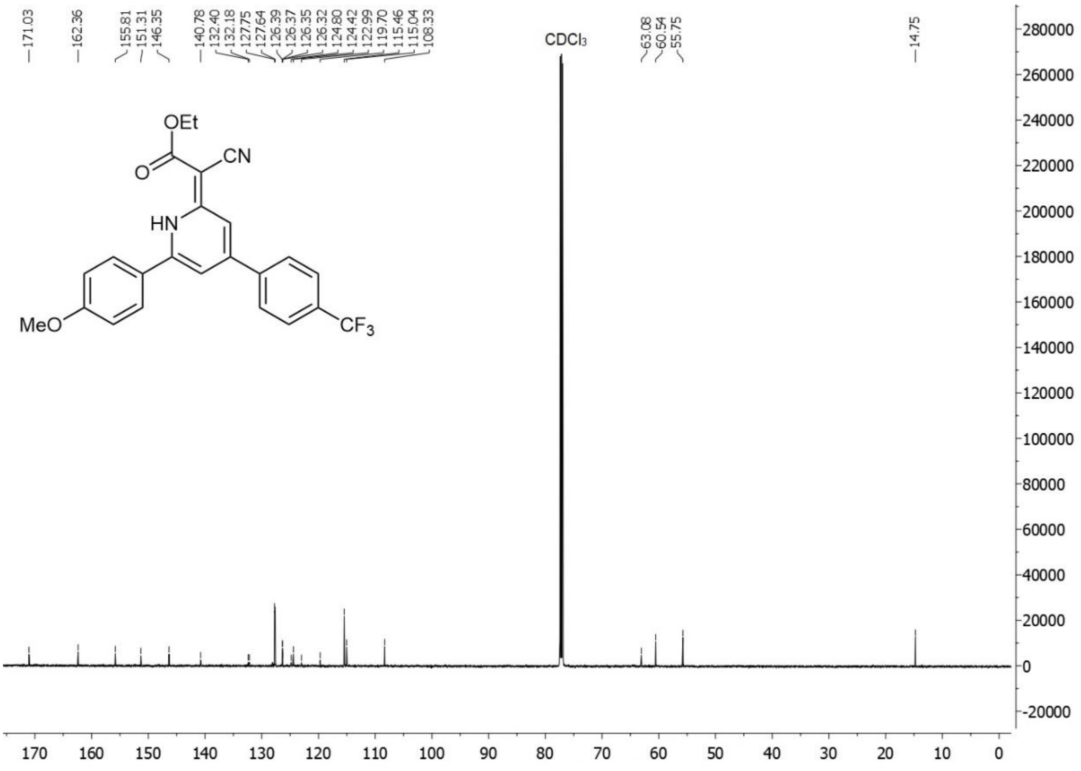

^{13}C NMR spectrum of compound **5f** ($CDCl_3$, 75 MHz, 293 K).

3.7 Ethyl (*Z*)-2-cyano-2-{4-(4-methoxyphenyl)-6-[4-(trifluoromethyl)phenyl]pyridin-2(1*H*)-ylidene}acetate (5g)

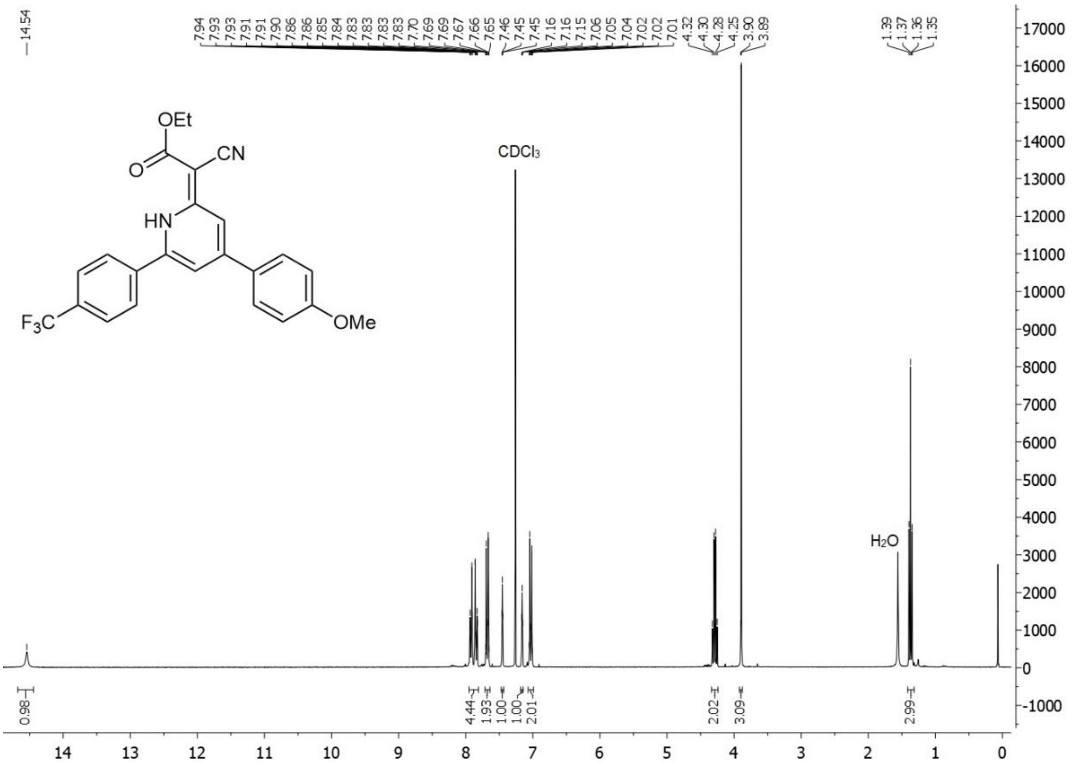

^{1}H NMR spectrum of compound **5g** ($CDCl_3$, 300 MHz, 293 K).

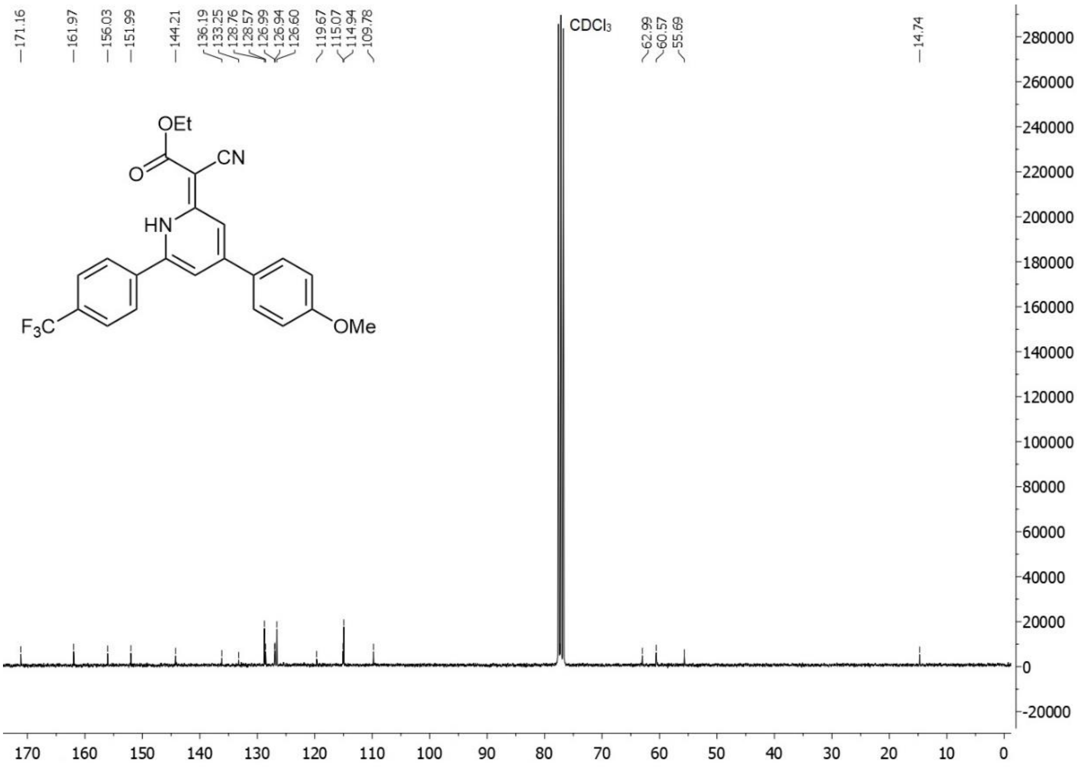

^{13}C NMR spectrum of compound **5g** ($CDCl_3$, 75 MHz, 293 K).

3.8 Ethyl (*Z*)-2-cyano-2-[4-phenyl-6-(thiophen-2-yl)pyridin-2(1*H*)-ylidene]acetate (5h)

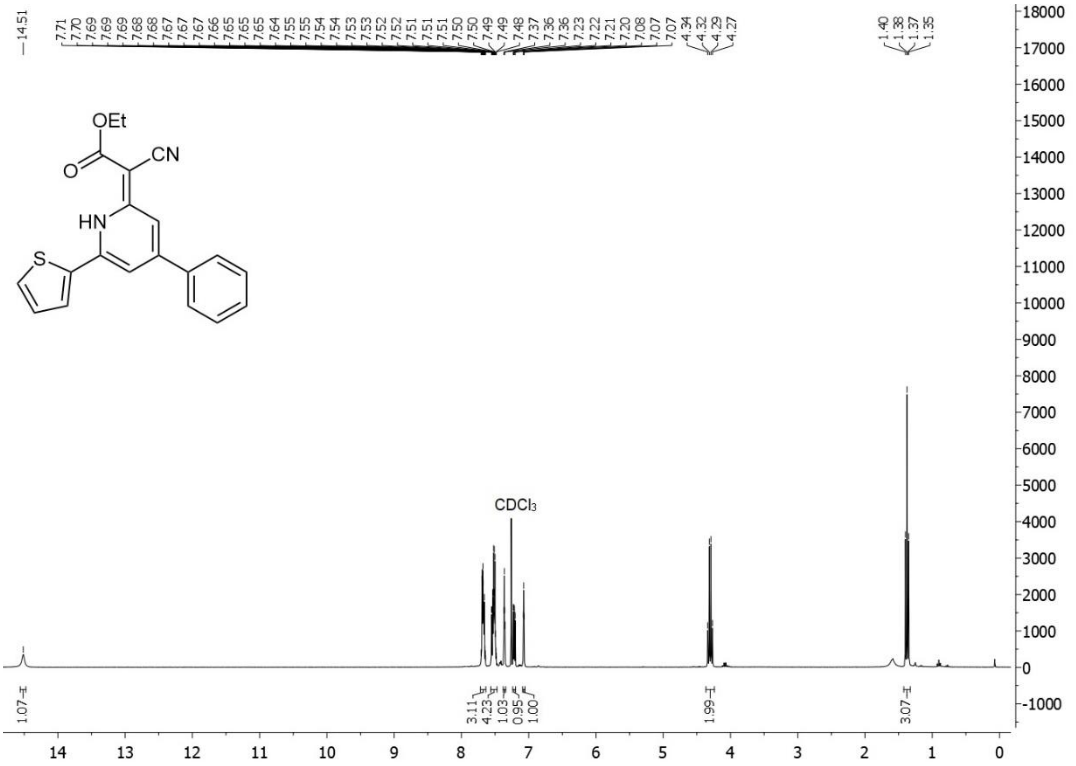

^{1}H NMR spectrum of compound **5h** ($CDCl_3$, 300 MHz, 293 K).

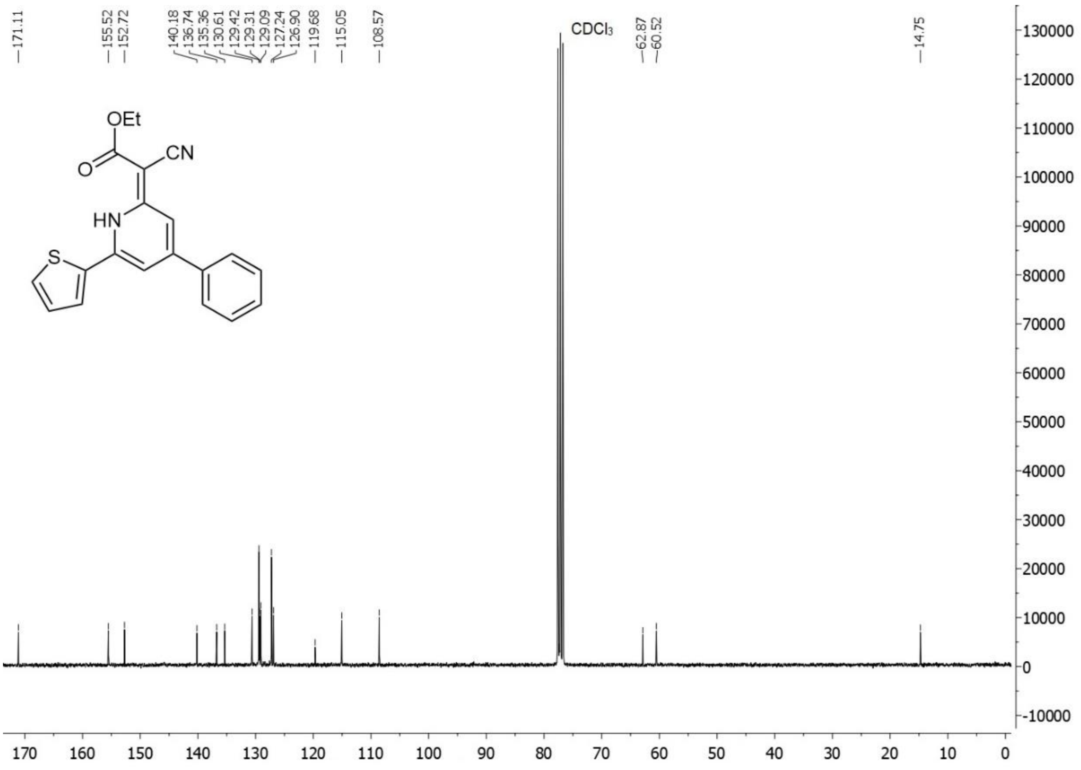

^{13}C NMR spectrum of compound **5h** ($CDCl_3$, 75 MHz, 293 K).

4 ^{1}H and ^{13}C NMR spectra of α-pyrones 6

4.1 2-Oxo-4,6-diphenyl-2*H*-pyran-3-carbonitrile (6a)

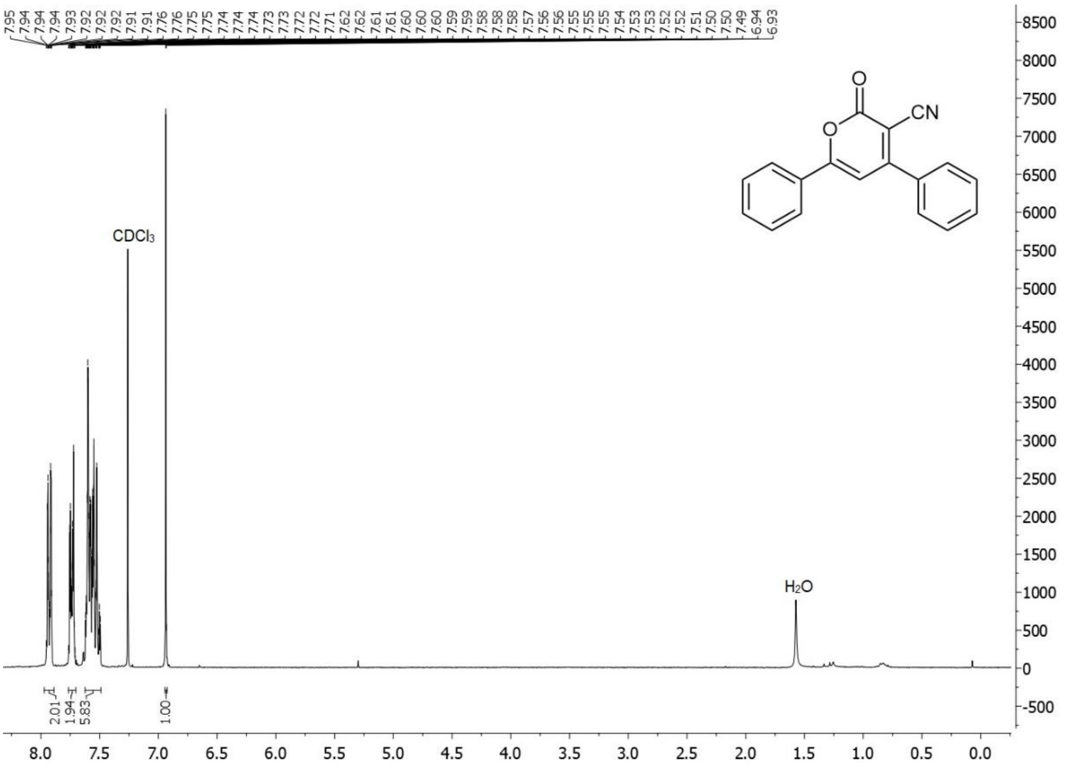

^{1}H NMR spectrum of compound **6a** ($CDCl_3$, 300 MHz, 293 K).

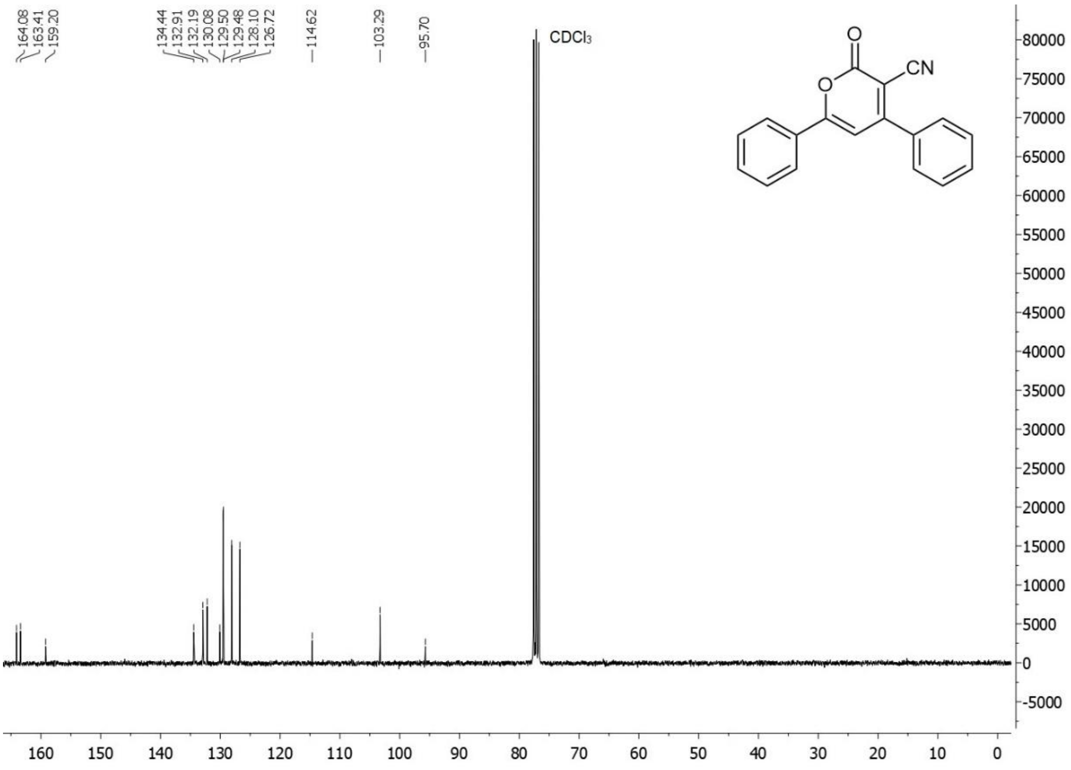

^{13}C NMR spectrum of compound **6a** ($CDCl_3$, 75 MHz, 293 K).

4.2 6-(4-Methoxyphenyl)-2-oxo-4-phenyl-2*H*-pyran-3-carbonitrile (6b)

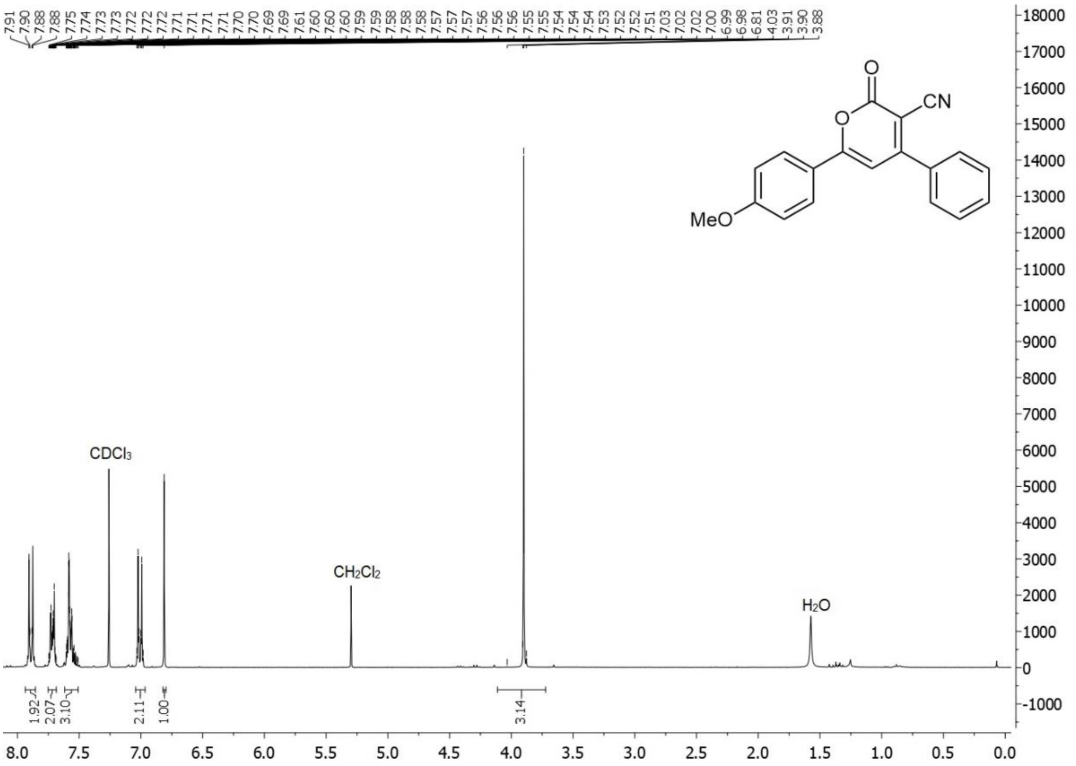

^{1}H NMR spectrum of compound **6b** ($CDCl_3$, 300 MHz, 293 K).

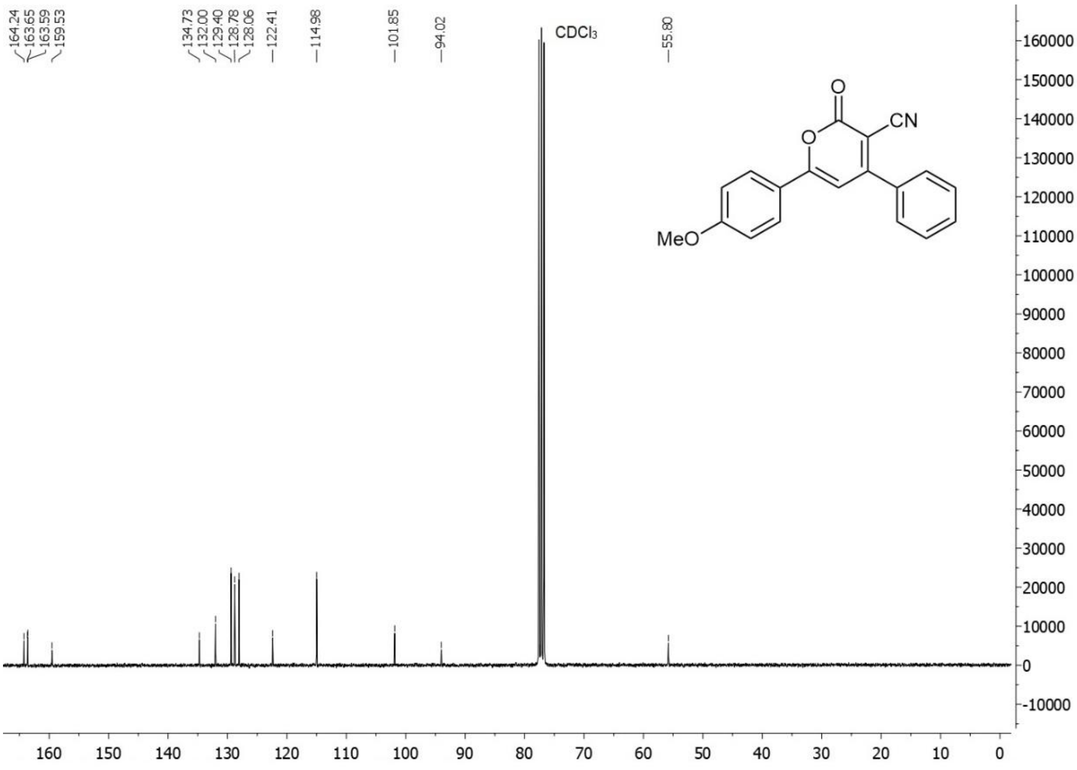

^{13}C NMR spectrum of compound **6b** ($CDCl_3$, 75 MHz, 293 K).

4.3 6-[4-(Dimethylamino)phenyl]-2-oxo-4-phenyl-2*H*-pyran-3-carbonitrile (6c)

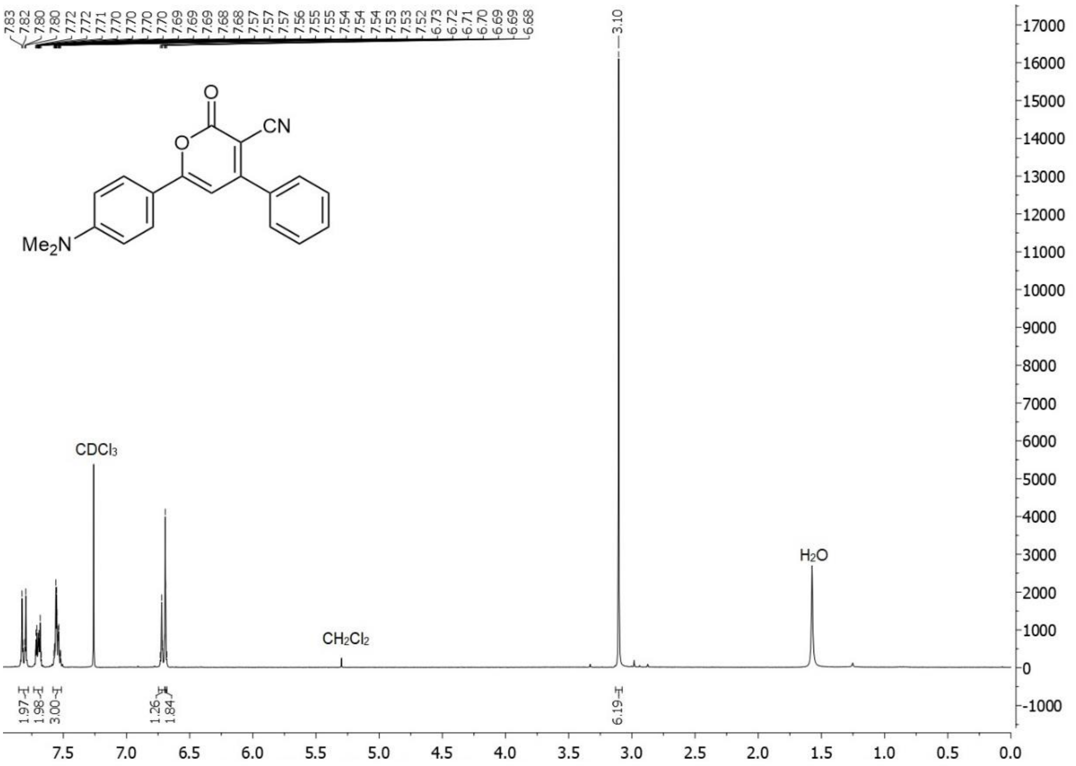

^{1}H NMR spectrum of compound **6c** ($CDCl_3$, 300 MHz, 293 K).

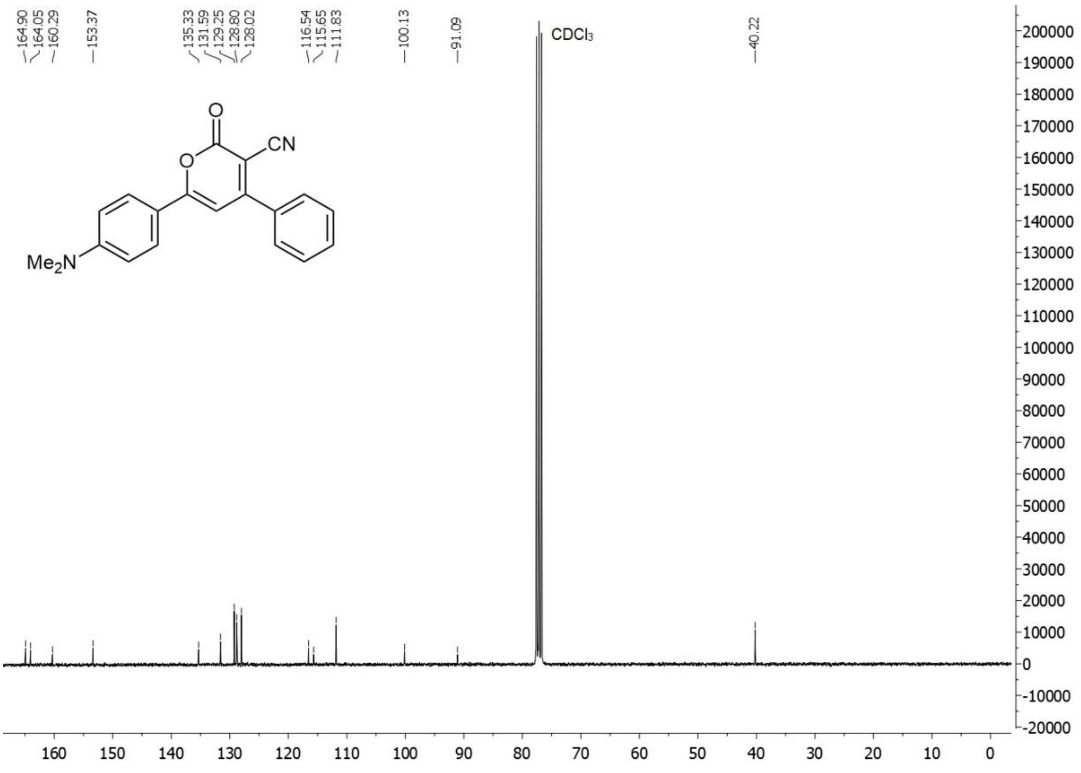

^{13}C NMR spectrum of compound **6c** ($CDCl_3$, 75 MHz, 293 K).

4.4 4-(4-Methoxyphenyl)-2-oxo-6-phenyl-2*H*-pyran-3-carbonitrile (6d)

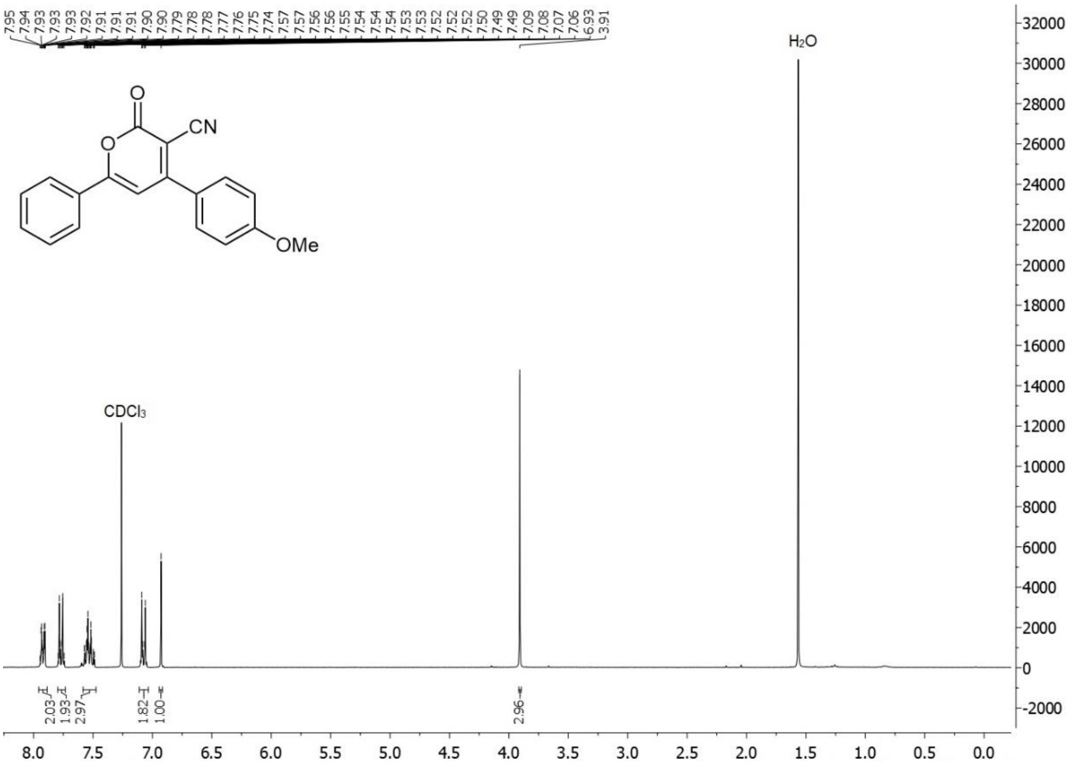

^{1}H NMR spectrum of compound **6d** ($CDCl_3$, 300 MHz, 293 K).

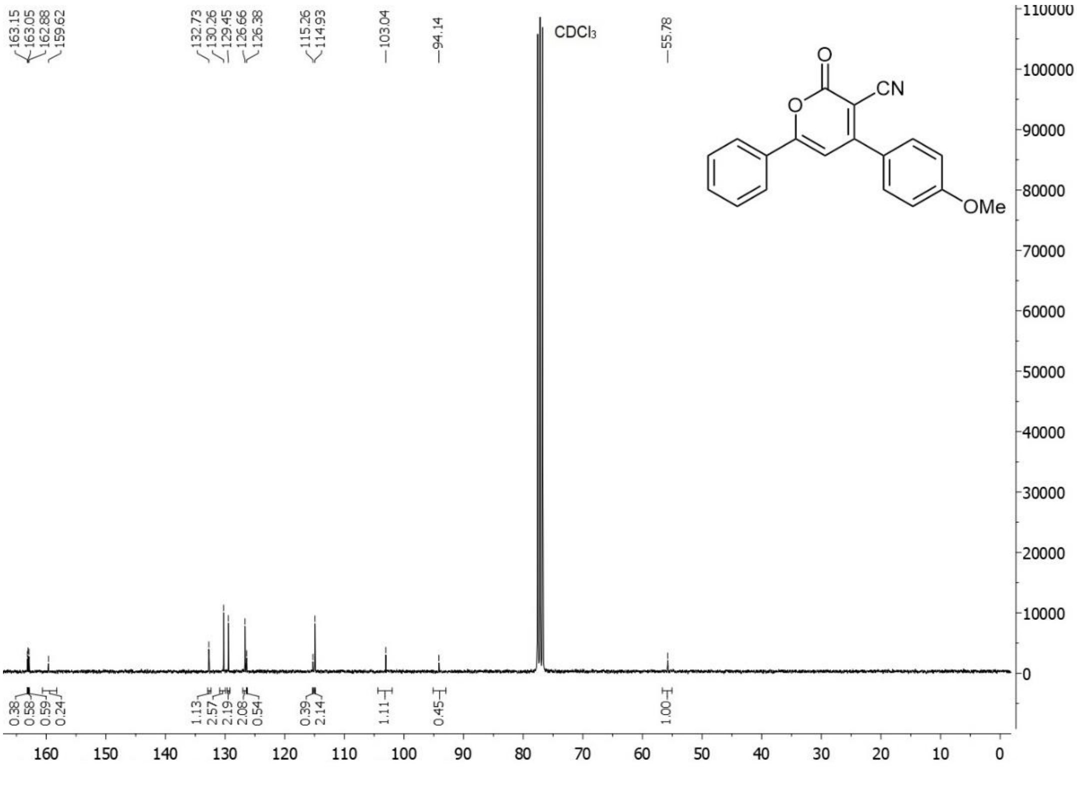

^{13}C NMR spectrum of compound **6d** ($CDCl_3$, 75 MHz, 293 K).

4.5 4-[4-(Dimethylamino)phenyl]-2-oxo-6-phenyl-2*H*-pyran-3-carbonitrile (6e)

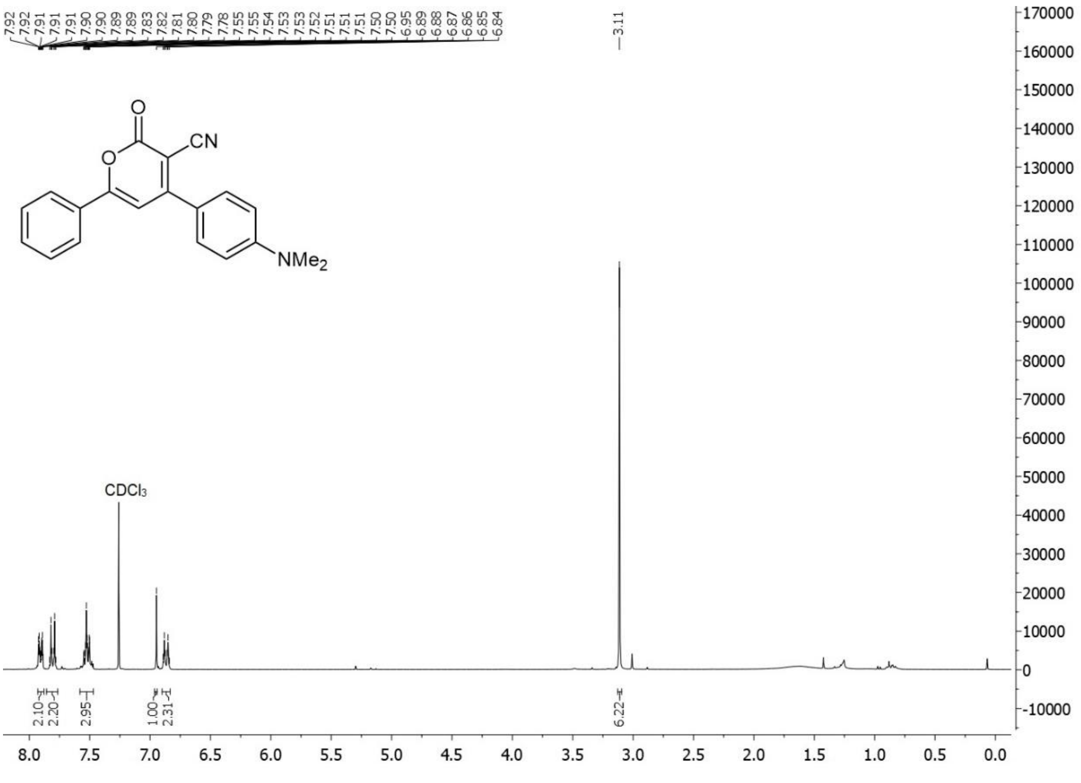

^{1}H NMR spectrum of compound **6e** ($CDCl_3$, 300 MHz, 293 K).

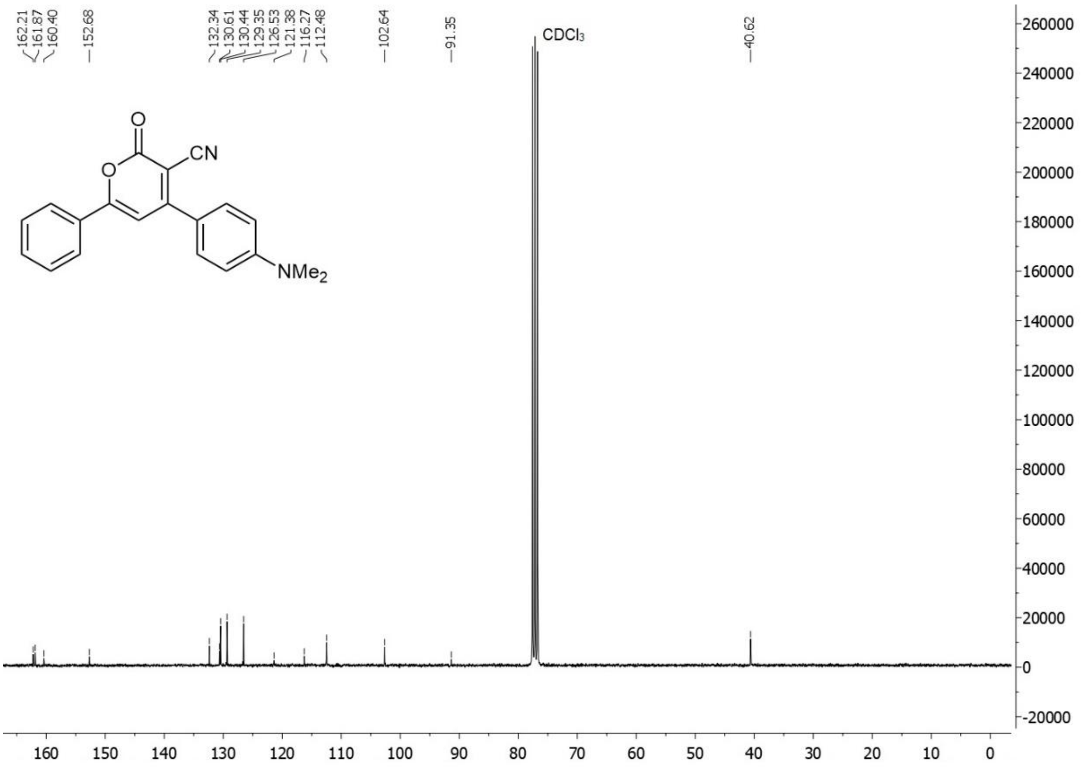

^{13}C NMR spectrum of compound **6e** ($CDCl_3$, 75 MHz, 293 K).

4.6 4,6-Bis(4-methoxyphenyl)-2-oxo-2*H*-pyran-3-carbonitrile (6f)

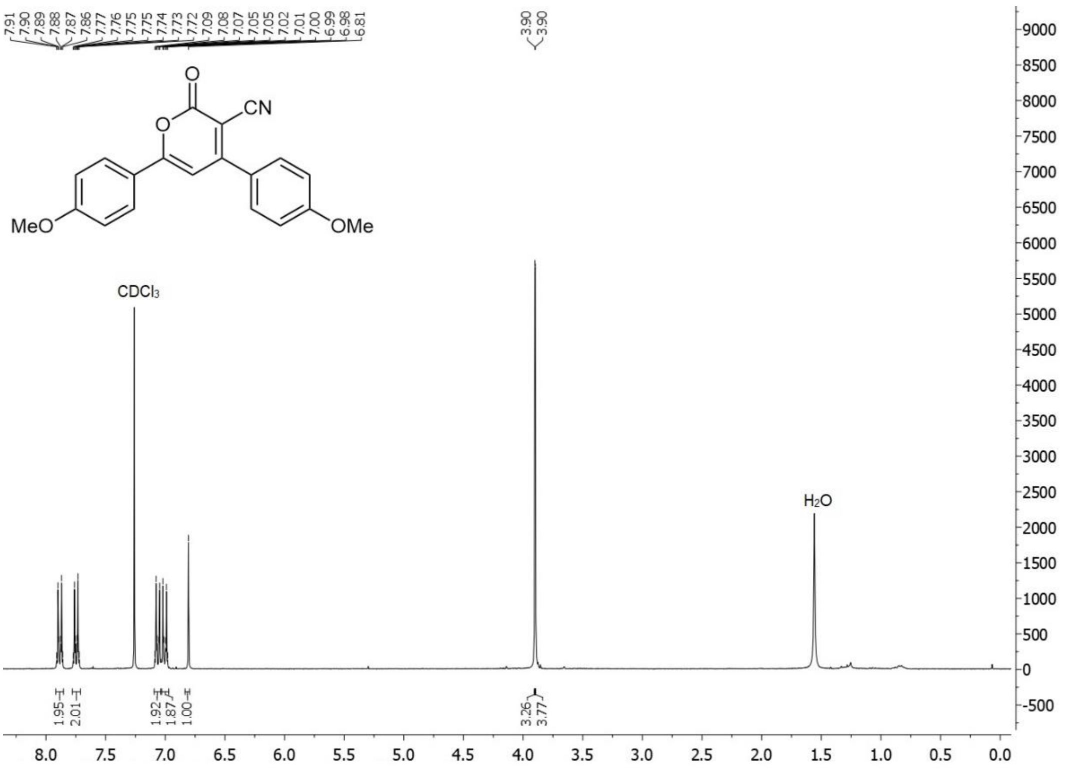

^1H NMR spectrum of compound **6f** ($CDCl_3$, 300 MHz, 293 K).

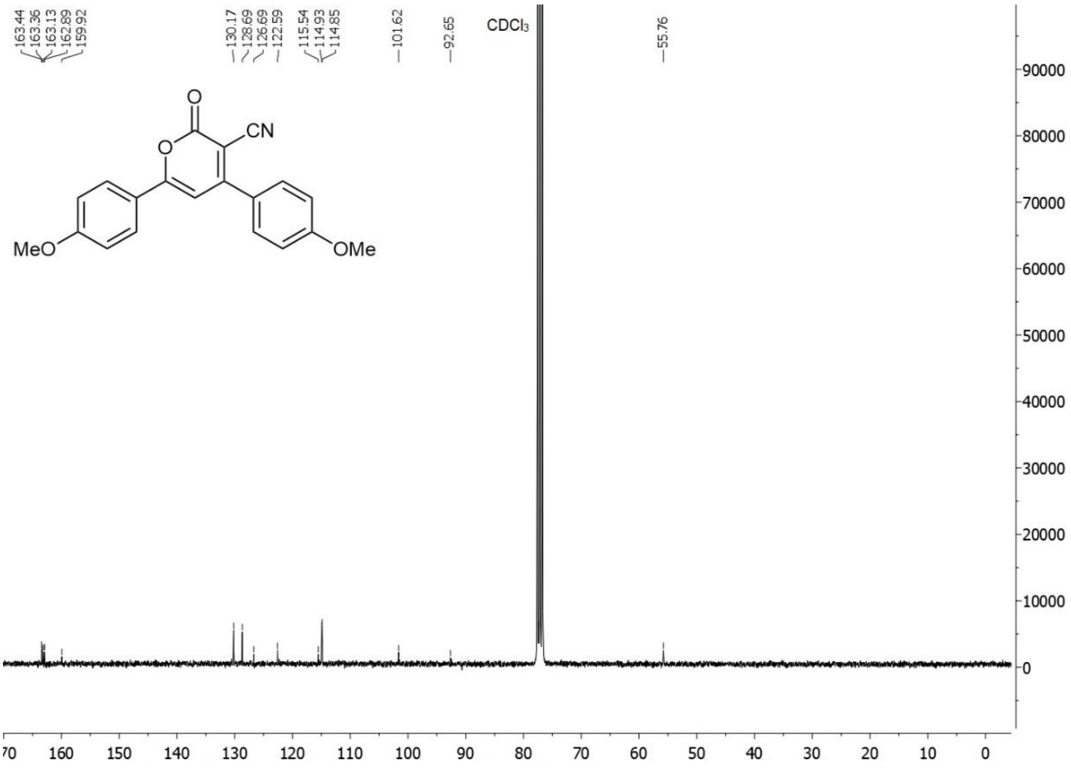

^{13}C NMR spectrum of compound **6f** ($CDCl_3$, 75 MHz, 293 K).

4.7 4-[4-(Dimethylamino)phenyl]-2-oxo-6-[4-(trifluormethyl)pheny]-2*H*-pyran-3-carbonitrile (6g)

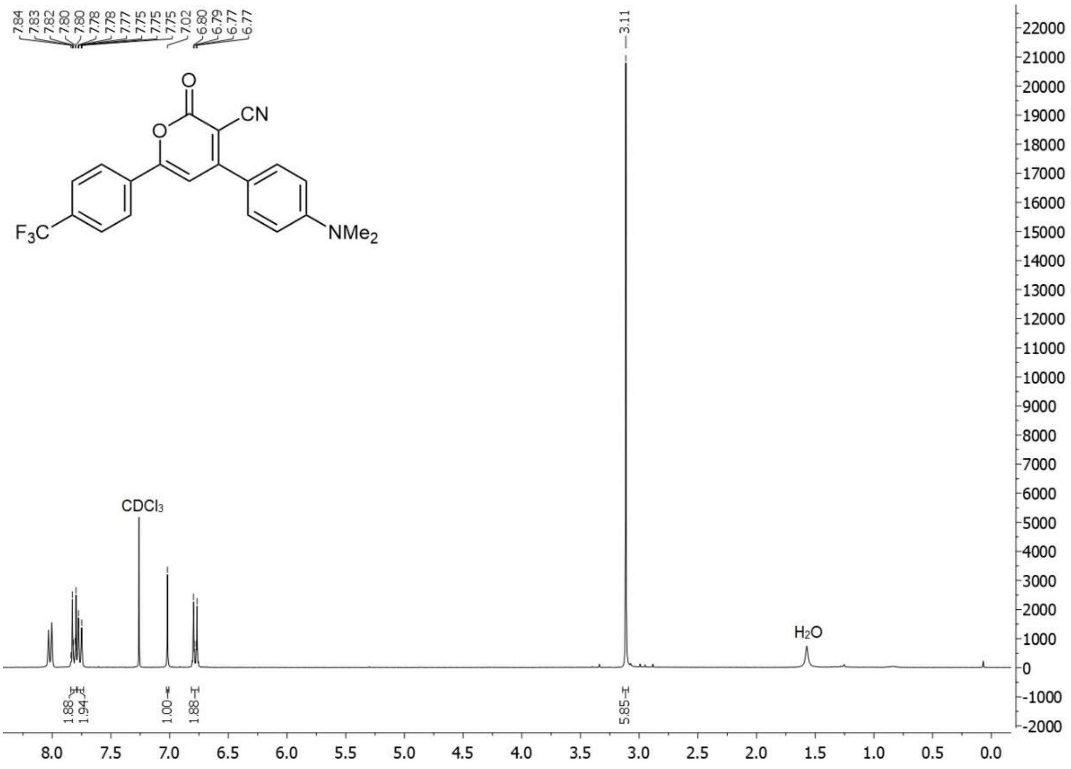

^{1}H NMR spectrum of compound **6g** ($CDCl_3$, 300 MHz, 293 K).

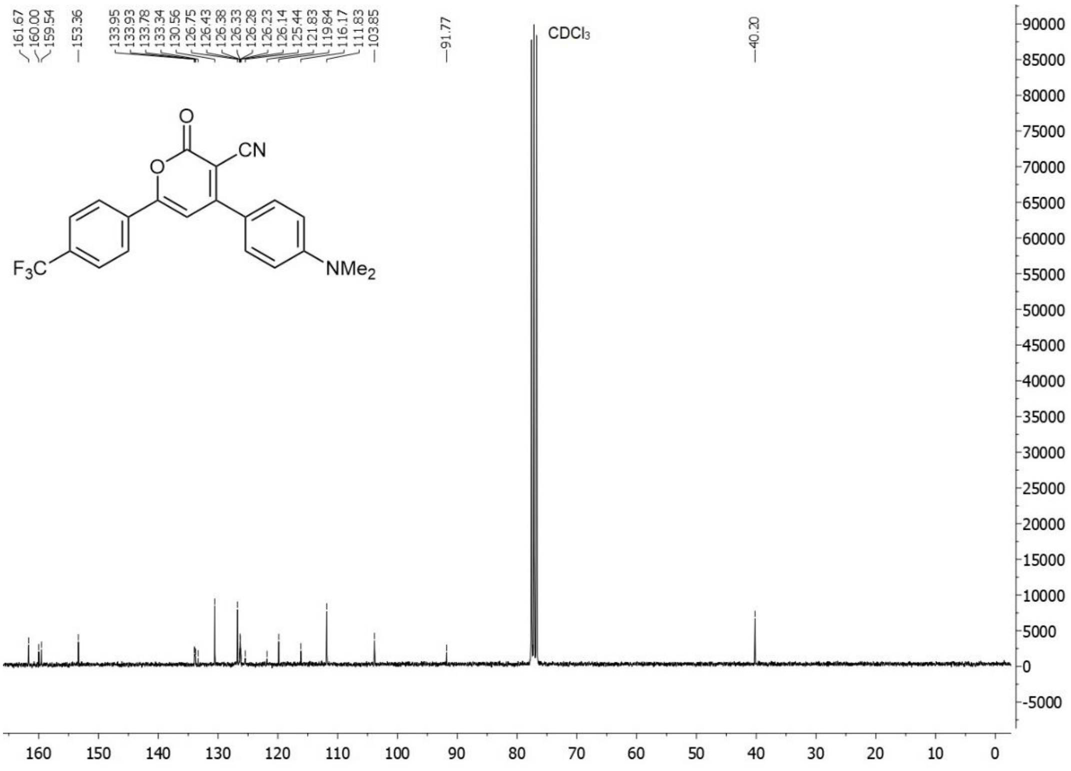

^{13}C NMR spectrum of compound **6g** ($CDCl_3$, 75 MHz, 293 K).

5 ^{1}H and ^{13}C NMR spectra of 1*H*-pyridines 8

5.1 Ethyl (*Z*)-2-(1-cyano-2-ethoxy-2-oxoethylidene)-4,6-diphenyl-1,2-dihydropyridin-3-carboxylate (8a)

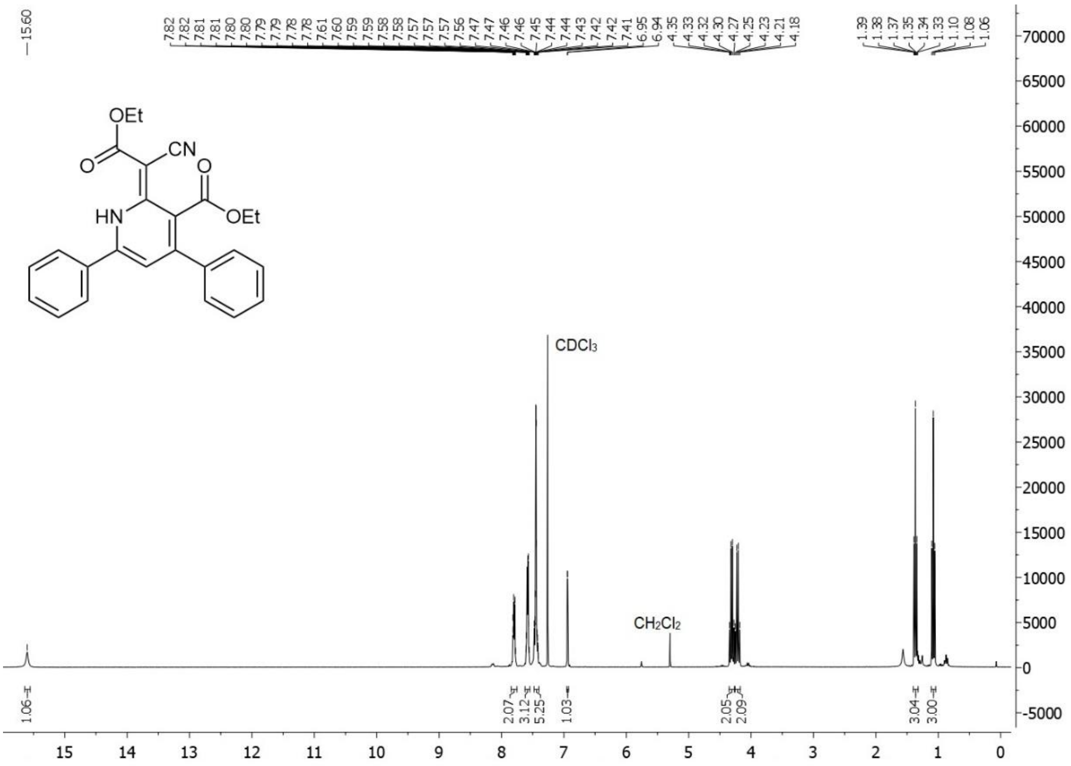

^{1}H NMR spectrum of compound **8a** ($CDCl_3$, 300 MHz, 293 K).

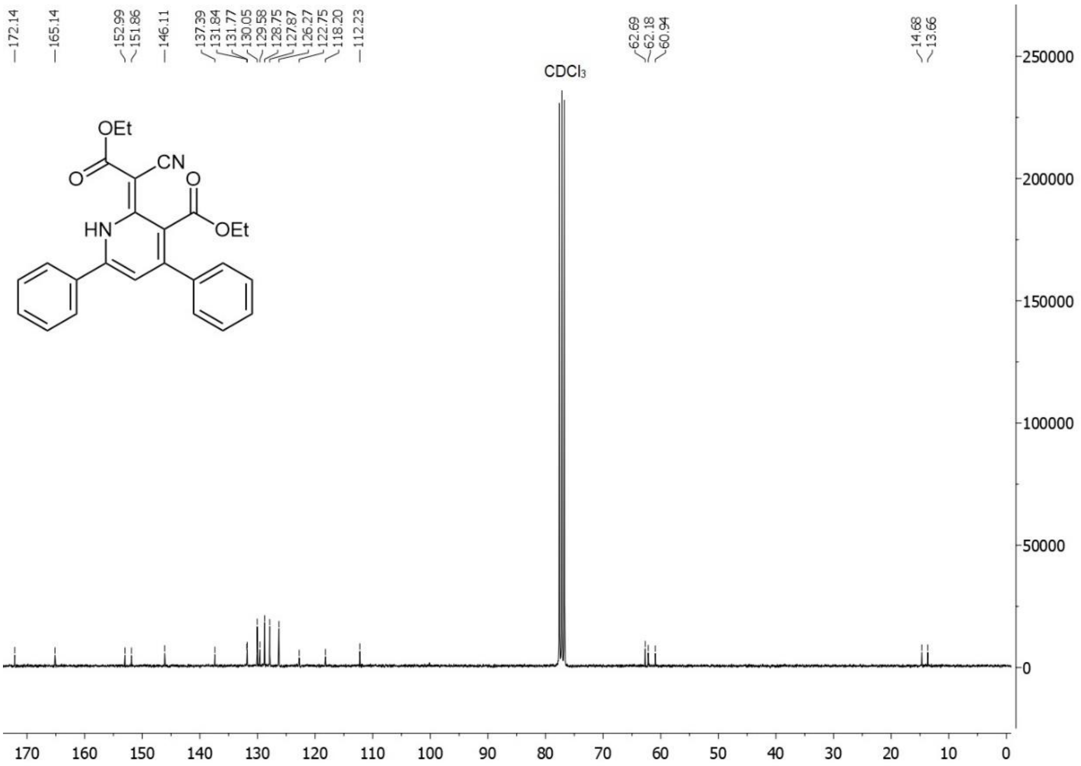

^{13}C NMR spectrum of compound **8a** ($CDCl_3$, 75 MHz, 293 K).

5.2 Ethyl (*Z*)-2-(1-cyano-2-ethoxy-2-oxoethyliden)-6-(4-methoxyphenyl)-4-phenyl-1,2-dihydropyridine-3-carboxylate (8b)

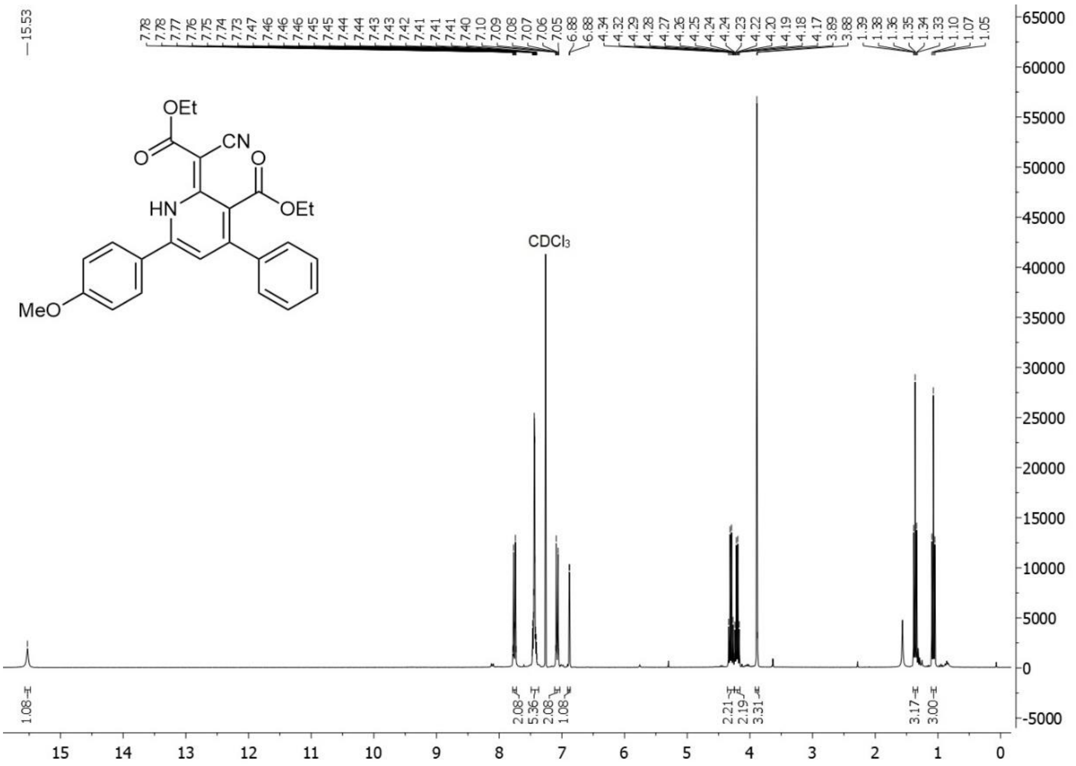

^{1}H NMR spectrum of compound **8b** ($CDCl_3$, 300 MHz, 293 K).

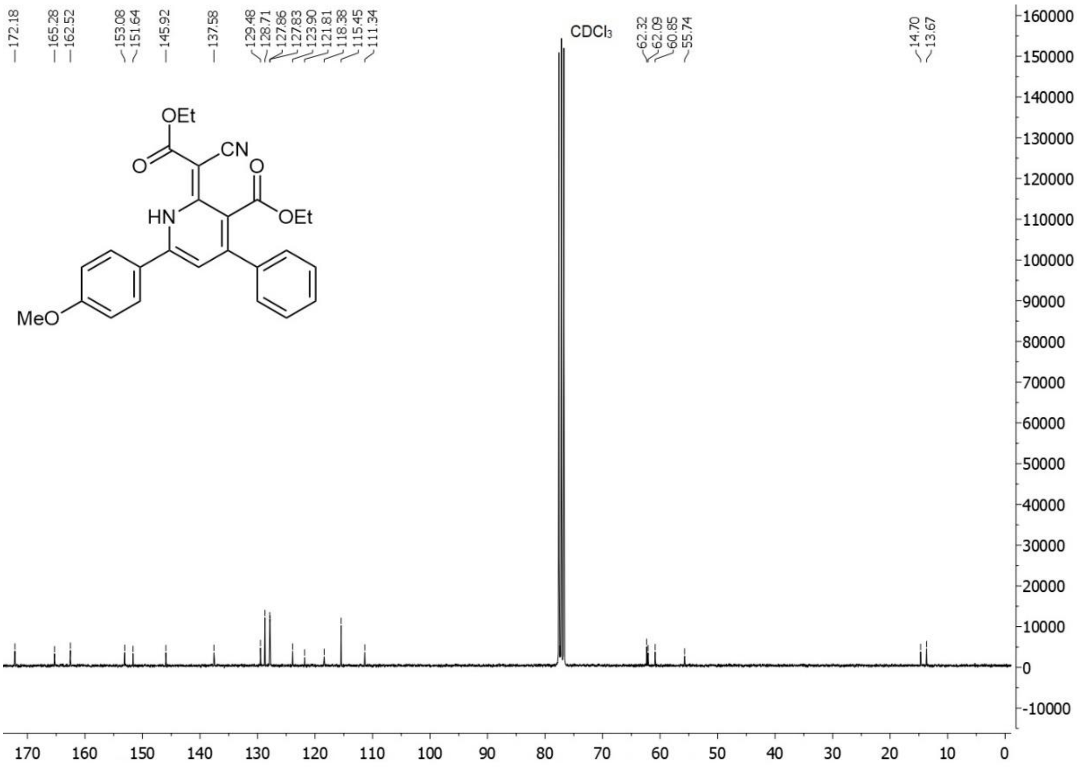

^{13}C NMR spectrum of compound **8b** ($CDCl_3$, 75 MHz, 293 K).

6 Absorption and emission spectra of 1*H*-pyridine 5

6.1 Ethyl (*Z*)-2-cyano-2-(4,6-diphenyl-1*H*-pyridin-2-ylidene)acetate (5a)

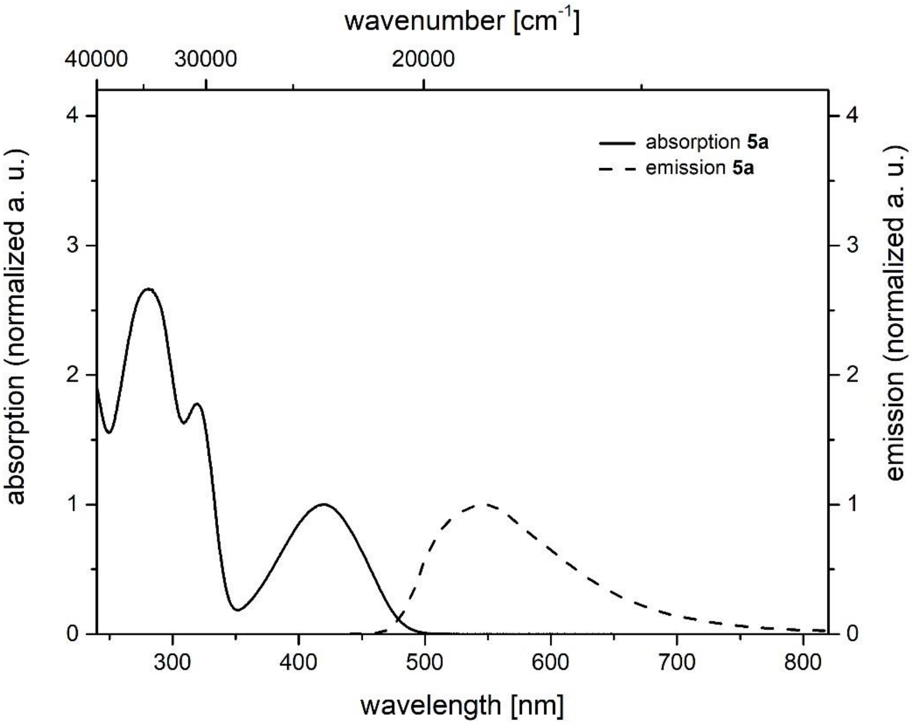

Recorded in dichloromethane at T = 293 K (λ_{exc} = 420 nm, c_o (absorption) = 10^{-6} M, c_o (emission) = 10^{-7} M).

6.2 Ethyl (*Z*)-2-cyano-2-{4-phenyl-6-[4-(trifluoromethyl)phenyl]-1*H*-pyridin-2-ylidene}acetate (5b)

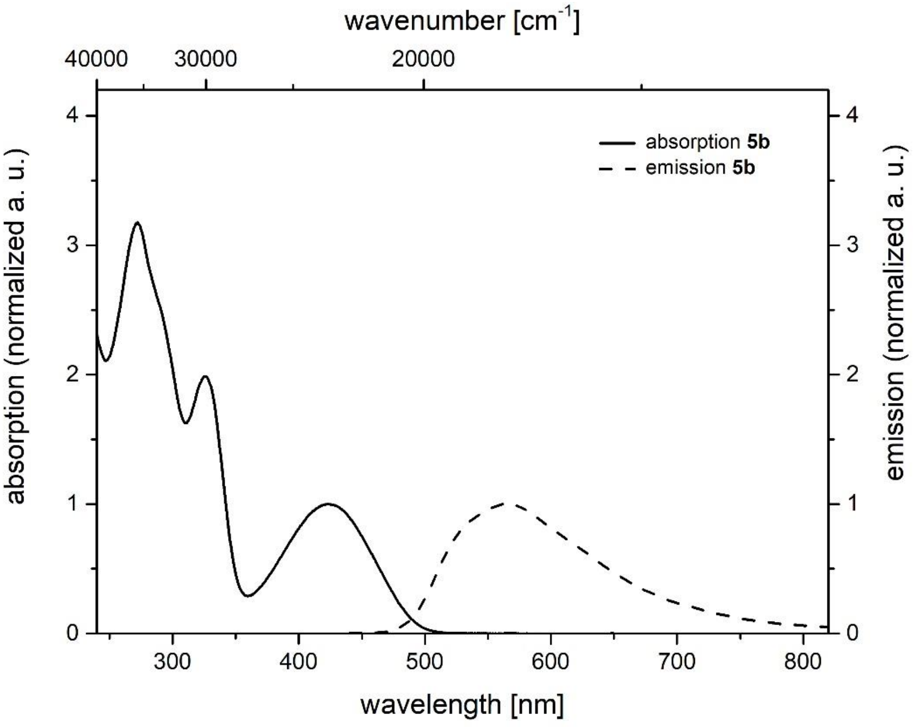

Recorded in dichloromethane at T = 293 K (λ_{exc} = 420 nm, c_o (absorption) = 10^{-6} M, c_o (emission) = 10^{-7} M).

6.3 Ethyl (*Z*)-2-cyano-2-[6-(4-cyanophenyl)-4-phenyl-1*H*-pyridin-2-ylidene]acetate (5c)

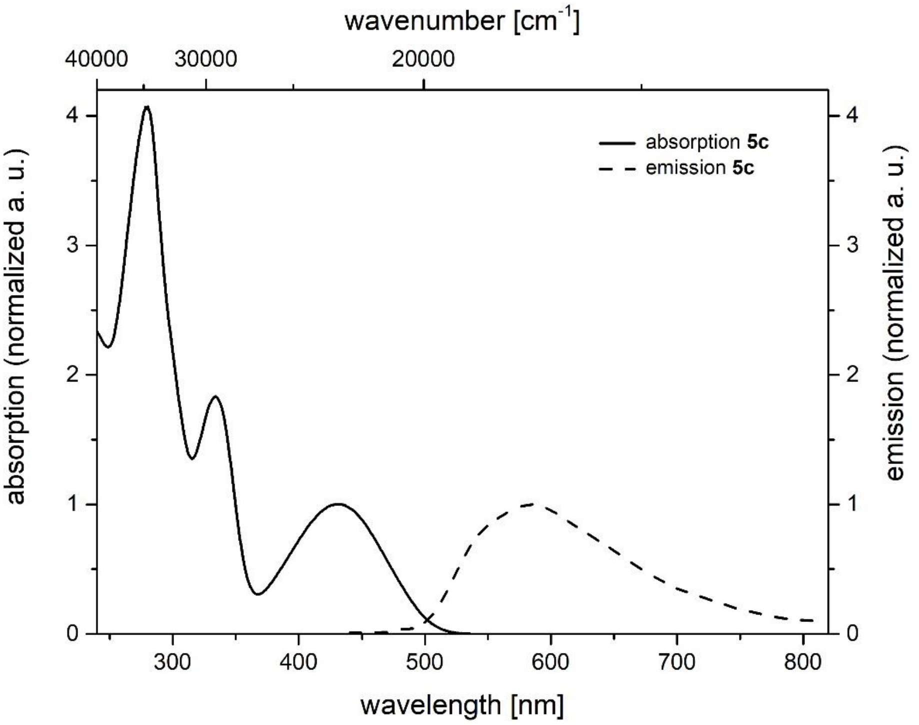

Recorded in dichloromethane at T = 293 K (λ_{exc} = 420 nm, c_0 (absorption) = 10^{-6} M, c_0 (emission) = 10^{-7} M).

6.4 Ethyl (*Z*)-2-cyano-2-{4-phenyl-6-[4-(trifluoromethyl)phenyl]-1*H*-pyridin-2-ylidene}acetate (5d)

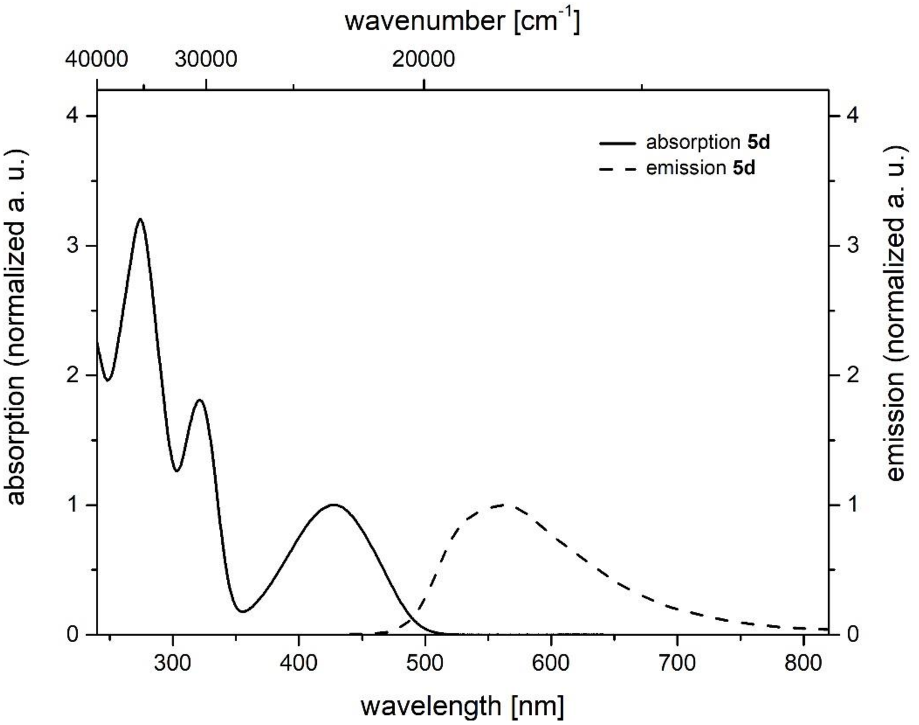

Recorded in dichloromethane at T = 293 K (λ_{exc} = 420 nm, c_0 (absorption) = 10^{-6} M, c_0 (emission) = 10^{-7} M).

6.5 Ethyl (*Z*)-2-cyano-2-[6-(4-cyanophenyl)-4-phenyl-1*H*-pyridin-2-ylidene]acetate (5e)

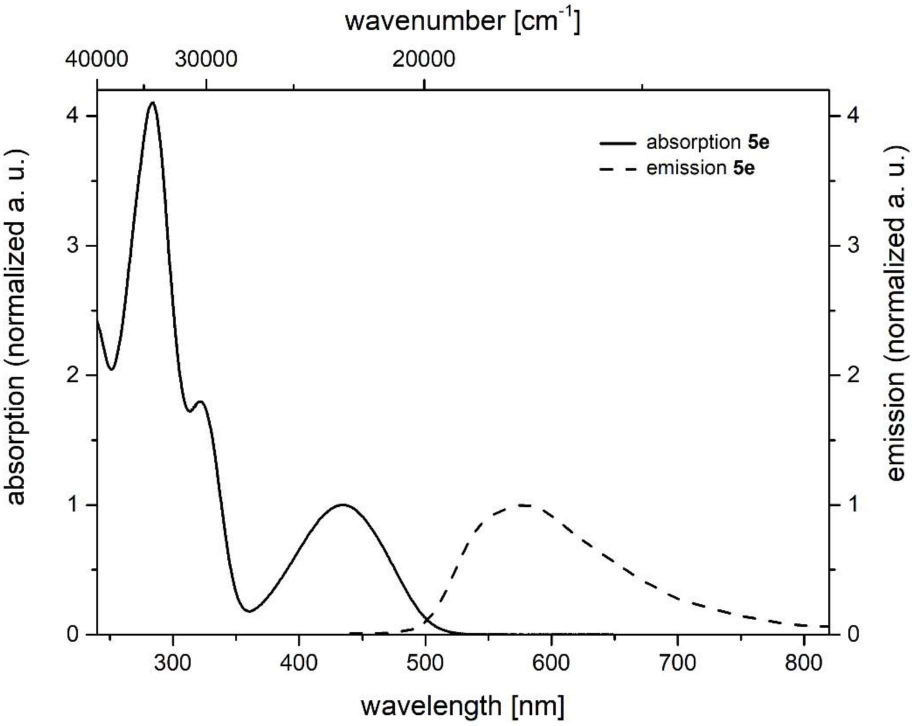

Recorded in dichloromethane at T = 293 K (λ_{exc} = 420 nm, c_o (absorption) = 10^{-6} M, c_o (emission) = 10^{-7} M).

6.6 Ethyl (*Z*)-2-cyano-2-[4-(4-methoxyphenyl]-6-[4-(trifluoromethyl)phenyl]pyridin-2(1*H*)-ylidene)acetate (5f)

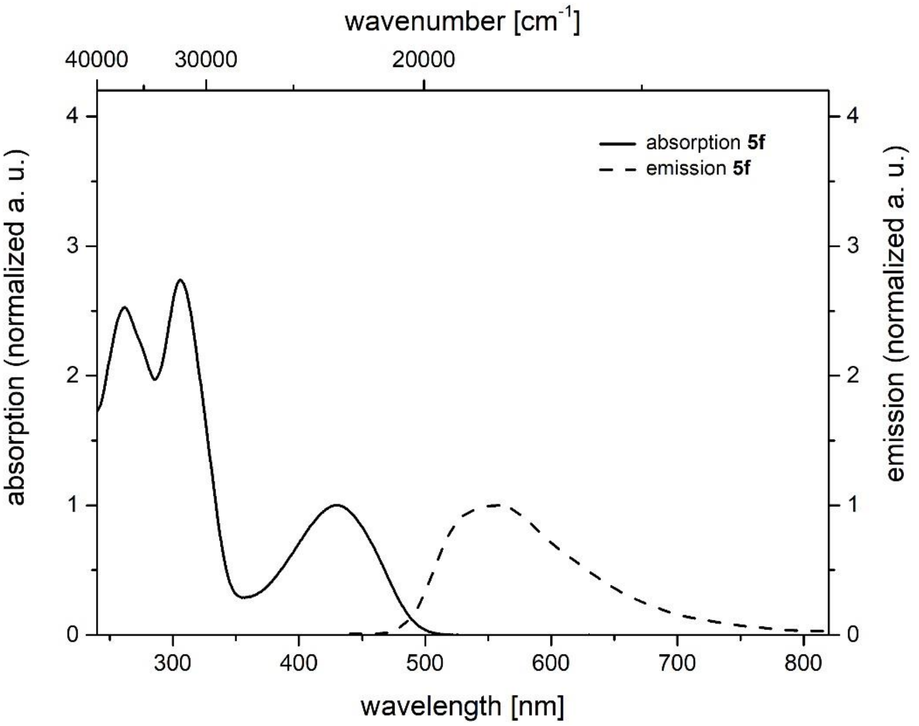

Recorded in dichloromethane at T = 293 K (λ_{exc} = 420 nm, c_o (absorption) = 10^{-6} M, c_o (emission) = 10^{-7} M).

6.7 Ethyl (*Z*)-2-cyano-2-{4-(4-methoxyphenyl)-6-[4-(trifluoromethyl)phenyl]pyridin-2(1*H*)-ylidene}acetate (5g)

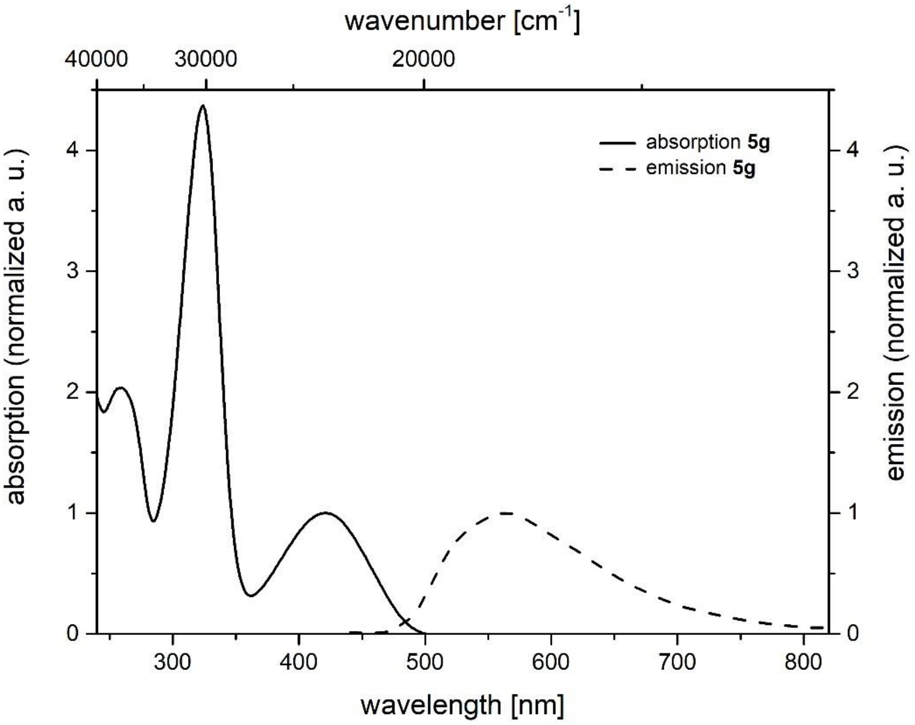

Recorded in dichloromethane at *T* = 293 K (λ_{exc} = 420 nm, c_0 (absorption) = 10^{-6} M, c_0 (emission) = 10^{-7} M).

6.8 Ethyl (*Z*)-2-cyano-2-[4-phenyl-6-(thiophen-2-yl)pyridin-2(1*H*)-ylidene]acetate (5h)

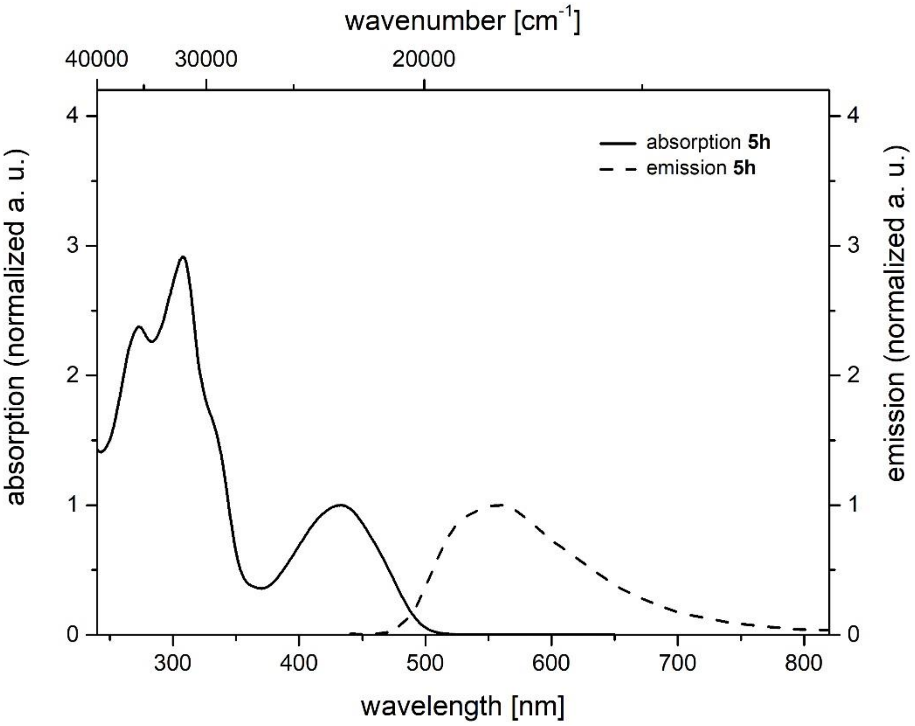

Recorded in dichloromethane at *T* = 293 K (λ_{exc} = 420 nm, c_0 (absorption) = 10^{-6} M, c_0 (emission) = 10^{-7} M).

7 Absorption and emission spectra of α-pyrones 6

7.1 2-Oxo-4,6-diphenyl-2*H*-pyran-3-carbonitrile (6a)

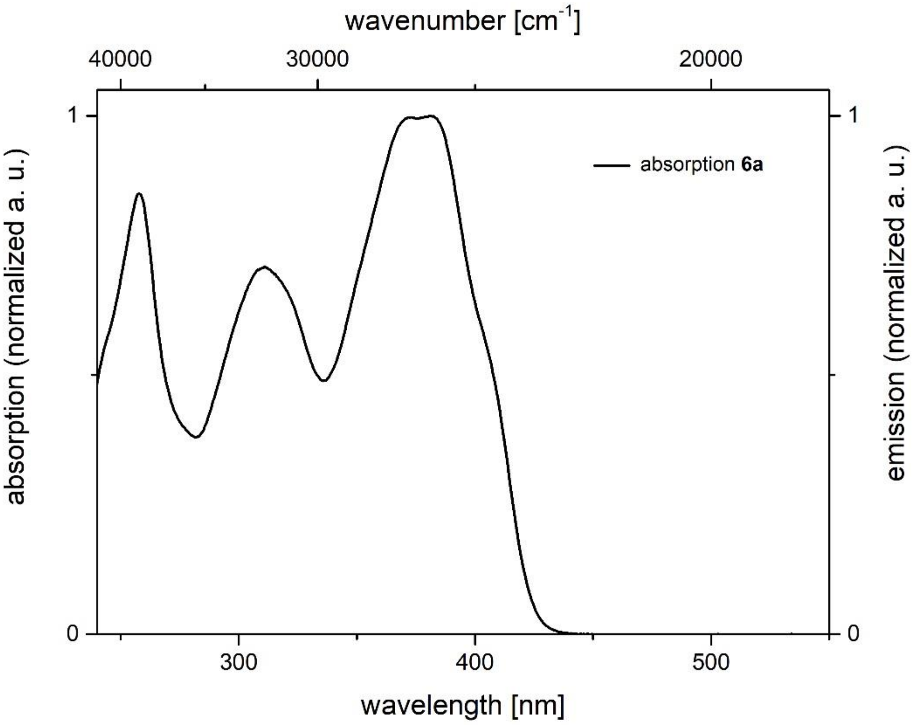

Recorded in dichloromethane at T = 293 K (λ_{exc} = 465 nm, c_0 (absorption) = 10^{-6} M.

7.2 6-(4-Methoxyphenyl)-2-oxo-4-phenyl-2*H*-pyran-3-carbonitrile (6b)

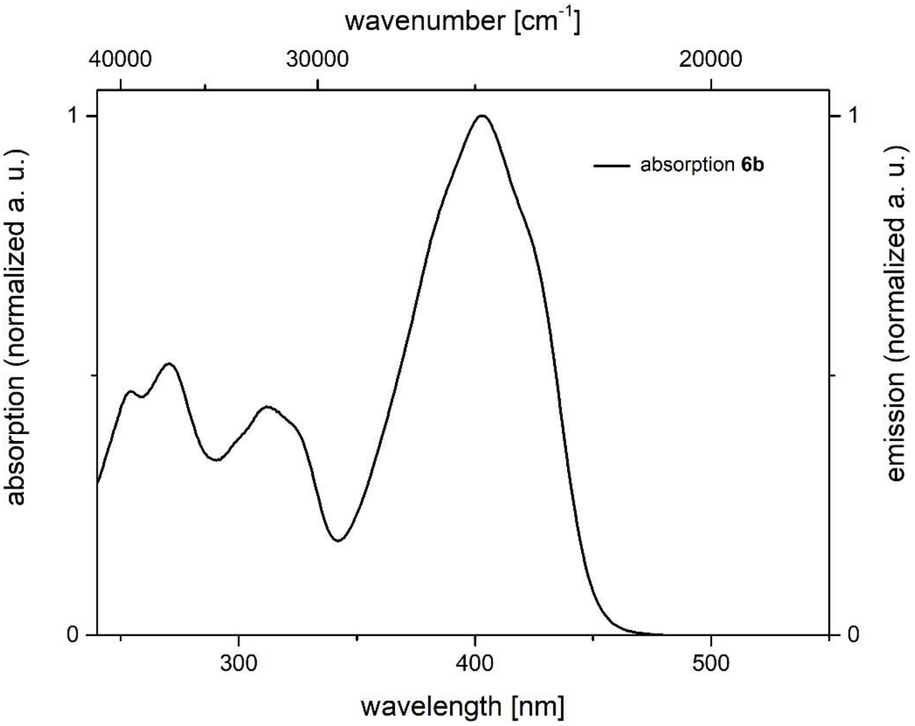

Recorded in dichloromethane at T = 293 K (λ_{exc} = 465 nm, c_0 (absorption) = 10^{-6} M.

7.3 6-[4-(Dimethylamino)phenyl]-2-oxo-4-phenyl-2*H*-pyran-3-carbonitrile (6c)

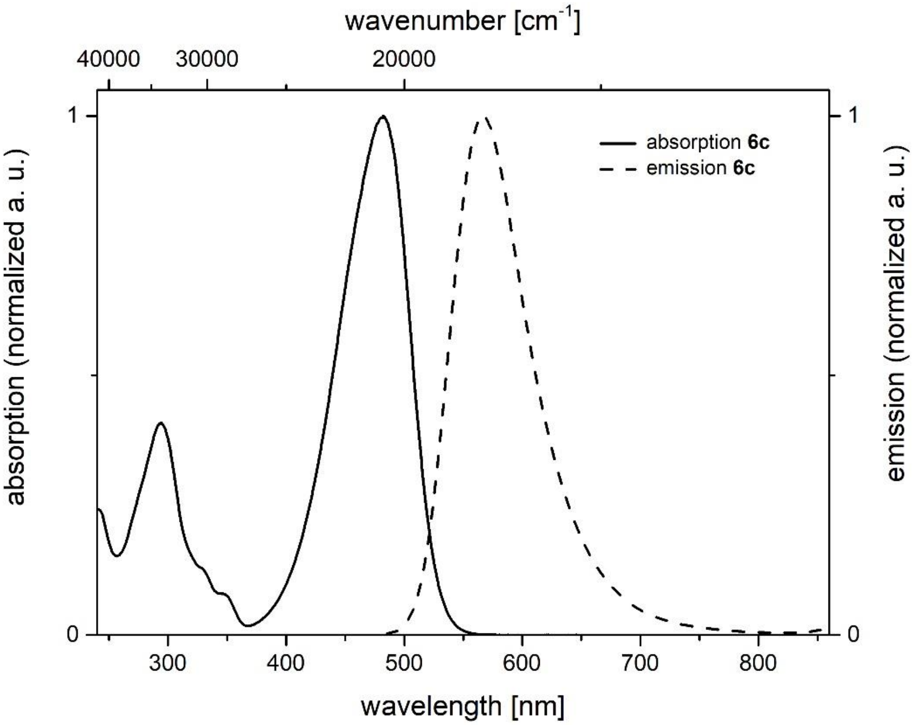

Recorded in dichloromethane at T = 293 K (λ_{exc} = 465 nm, c_o (absorption) = 10^{-6} M, c_o (emission) = 10^{-7} M).

7.4 4-(4-Methoxyphenyl)-2-oxo-6-phenyl-2*H*-pyran-3-carbonitrile (6d)

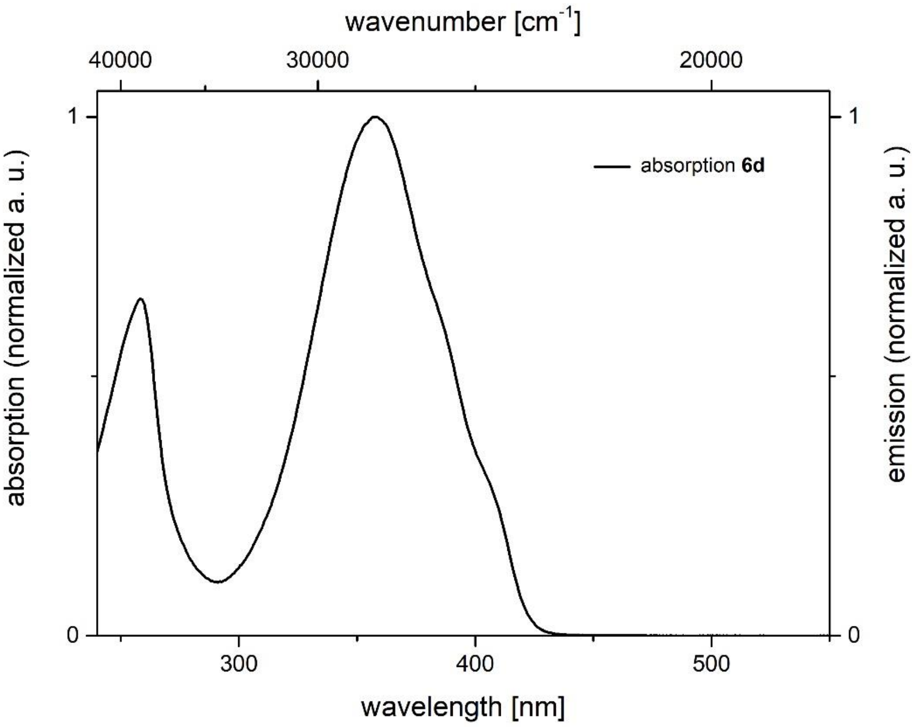

Recorded in dichloromethane at T = 293 K (λ_{exc} = 465 nm, c_o (absorption) = 10^{-6} M.

7.5 4-[4-(Dimethylamino)phenyl]-2-oxo-6-phenyl-2*H*-pyran-3-carbonitrile (6e)

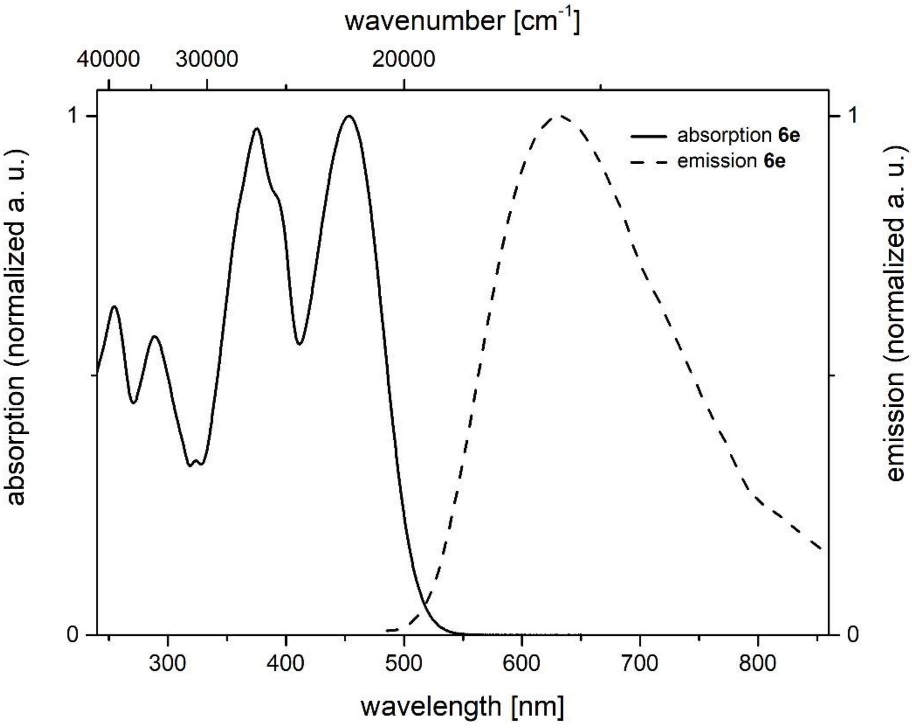

Recorded in dichloromethane at T = 293 K (λ_{exc} = 465 nm, c_o (absorption) = 10^{-6} M, c_o (emission) = 10^{-7} M).

7.6 4,6-Bis(4-methoxyphenyl)-2-oxo-2*H*-pyran-3-carbonitrile (6f)

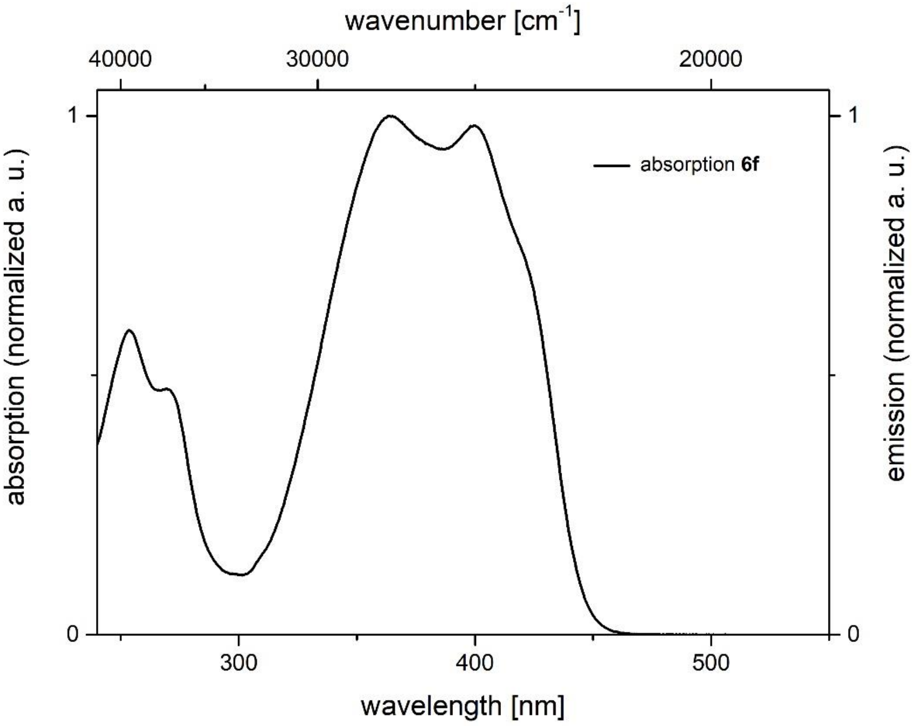

Recorded in dichloromethane at T = 293 K (λ_{exc} = 465 nm, c_o (absorption) = 10^{-6} M.

7.7 4-[4-(Dimethylamino)phenyl]-2-oxo-6-[4-(trifluormethyl)pheny]-2*H*-pyran-3-carbonitrile (6g)

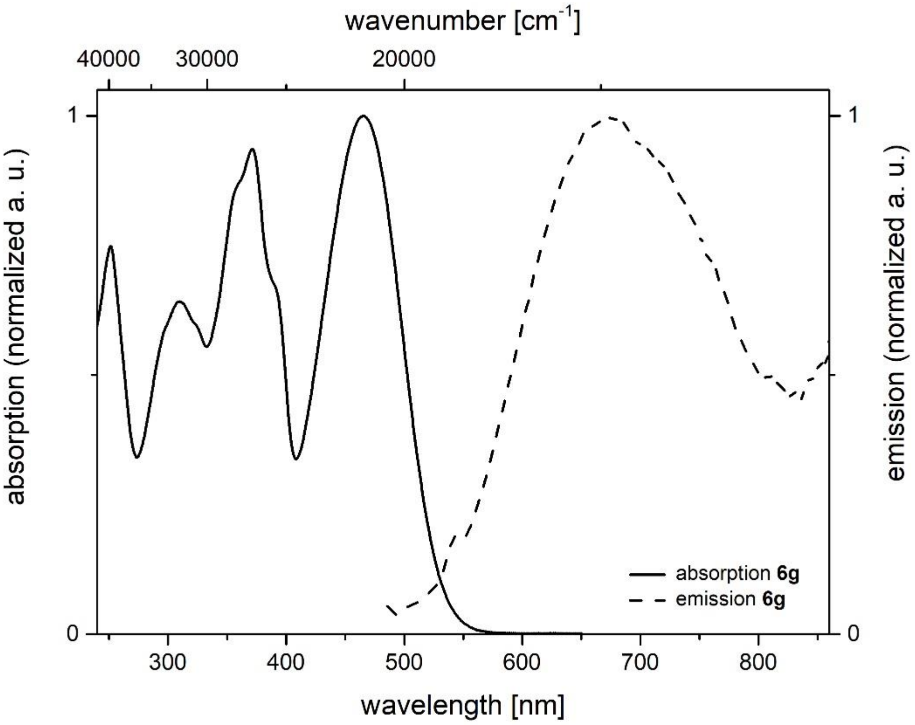

Recorded in dichloromethane at T = 293 K (λ_{exc} = 465 nm, c_0 (absorption) = 10^{-6} M, c_0 (emission) = 10^{-7} M).

7.8 Solvatochromism of 6-[4-(dimethylamino)phenyl]-2-oxo-4-phenyl-2*H*-pyran-3-carbonitrile (6c)

Table S3. Fluorescence of compound **6c** with variable solvent polarity.

Solvent	Δf	$\lambda_{max,abs}$ [nm][a] (ε [L·mol^{-1}·cm^{-1}])	$\lambda_{max,em}$ [nm][b]	Stokes shift $\Delta\tilde{\nu}$ [cm^{-1}]
toluene	0.0132	291, 470 (36200)	529	2400
ethyl acetate	0.1996	289, 469 (40400)	565	3600
acetone	0.2843	476 (43000)	598	4300
DMF	0.2744	293, 485 (41400)	616	4400
DMSO	0.2634	291, 490 (39700)	628	4500

7.9 Aggregation-induced enhanced emission of 4-[4-(dimethylamino)phenyl]-2-oxo-6-phenyl-2*H*-pyran-3-carbonitrile (6e)

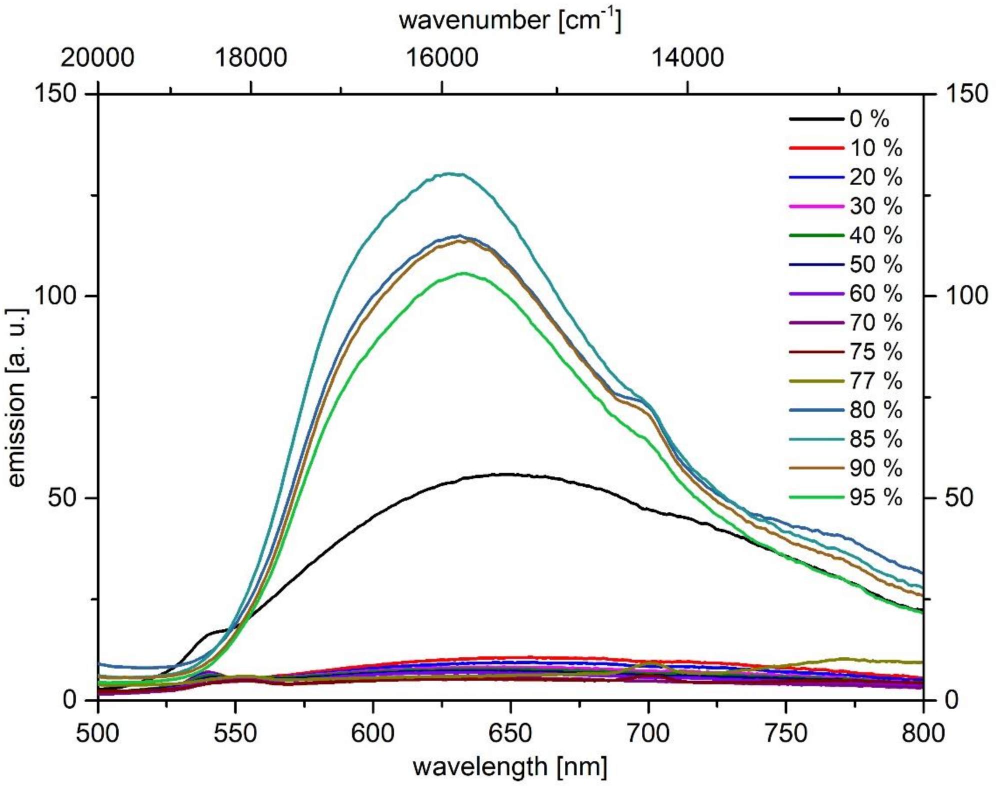

Figure 1: Emission spectra of α-pyrone **6e** in THF/water mixtures containing different water fractions (recorded at T = 298 K).

8 Absorption and emission spectra of 1*H*-pyridine 8

8.1 Ethyl (*Z*)-2-(1-cyano-2-ethoxy-2-oxoethylidene)-4,6-diphenyl-1,2-dihydropyridine-3-carboxylate (8a)

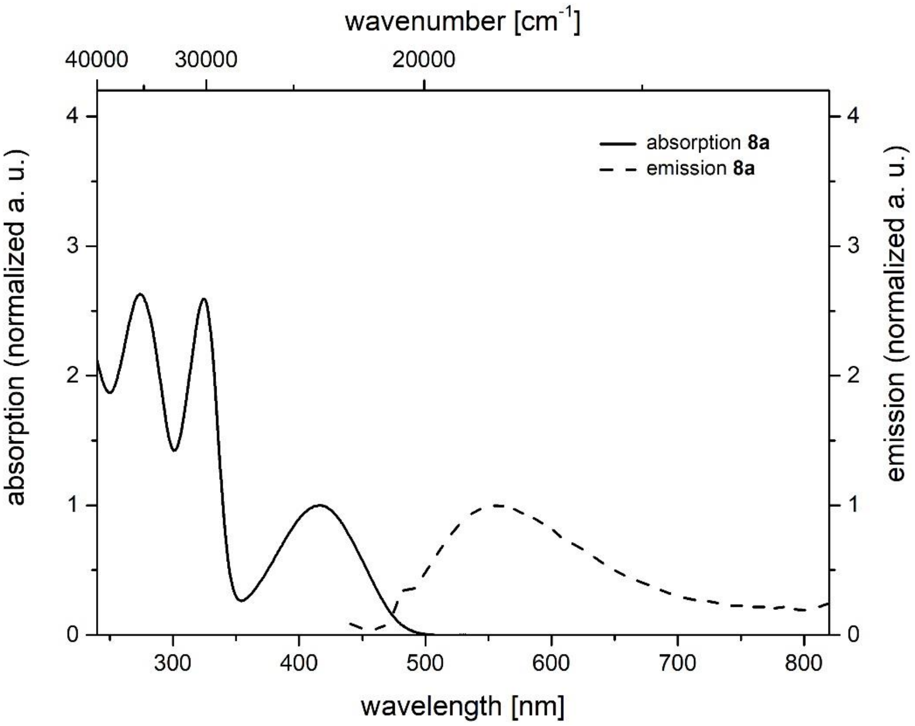

Recorded in dichloromethane at T = 293 K (λ_{exc} = 420 nm, c_0 (absorption) = 10^{-6} M, c_0 (emission) = 10^{-7} M).

8.2 Ethyl (*Z*)-2-(1-cyano-2-ethoxy-2-oxoethylidene)-6-(4-methoxyphenyl)-4-phenyl-1,2-dihydropyridine-3-carboxylate (8b)

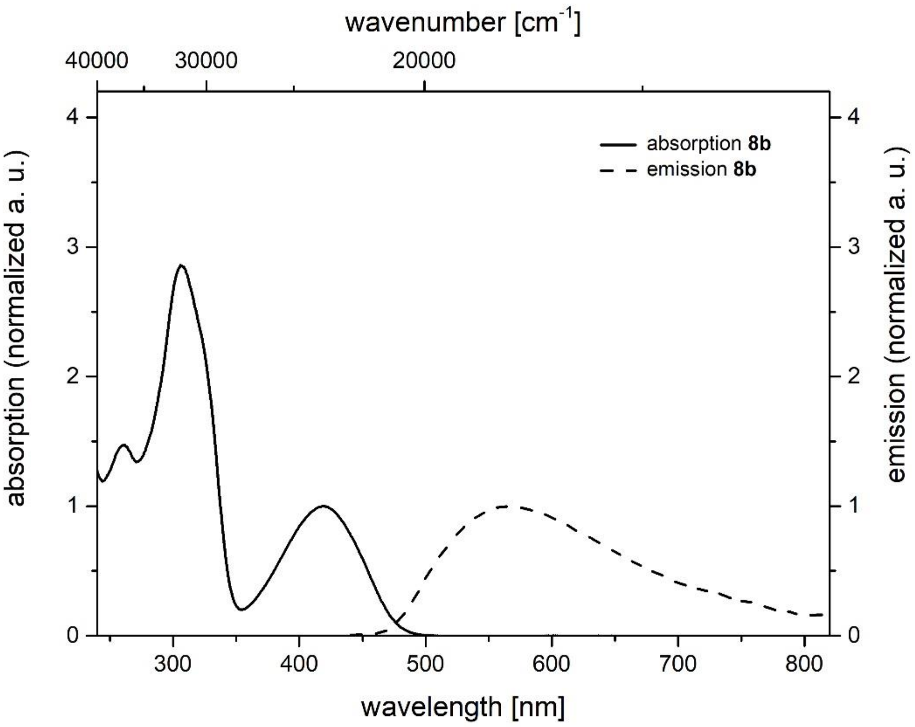

Recorded in dichloromethane at T = 293 K (λ_{exc} = 420 nm, c_0 (absorption) = 10^{-6} M, c_0 (emission) = 10^{-7} M).

9 Emission spectra of 1*H*-pyridines 5 in the solid state

9.1 Ethyl (*Z*)-2-cyano-2-(4,6-diphenyl-1*H*-pyridin-2-ylidene)acetate (5a)

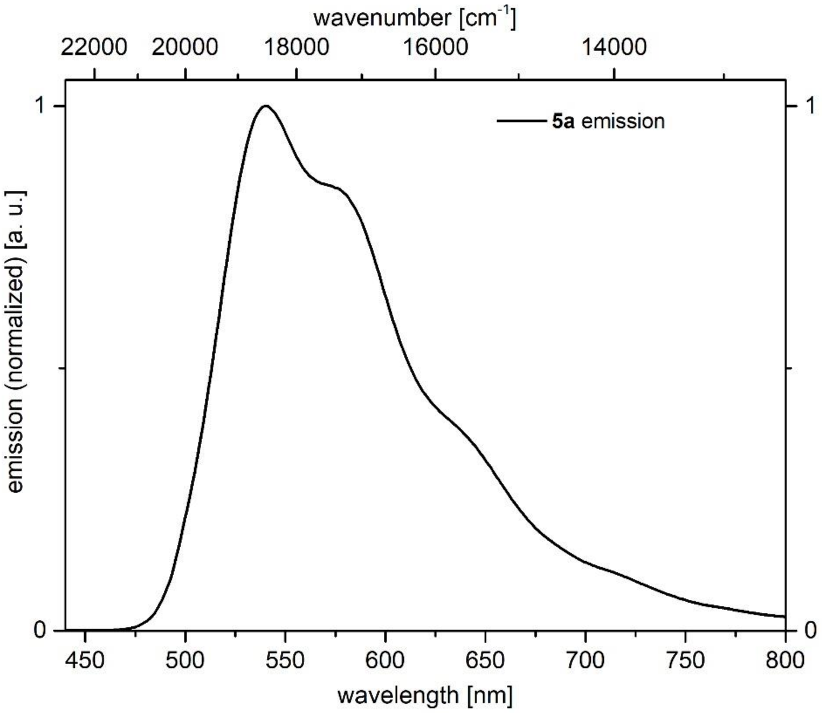

λ_{exc} = 420 nm

9.2 Ethyl (*Z*)-2-cyano-2-{4-phenyl-6-[4-(trifluoromethyl)phenyl]-1*H*-pyridin-2-ylidene}acetate (5b)

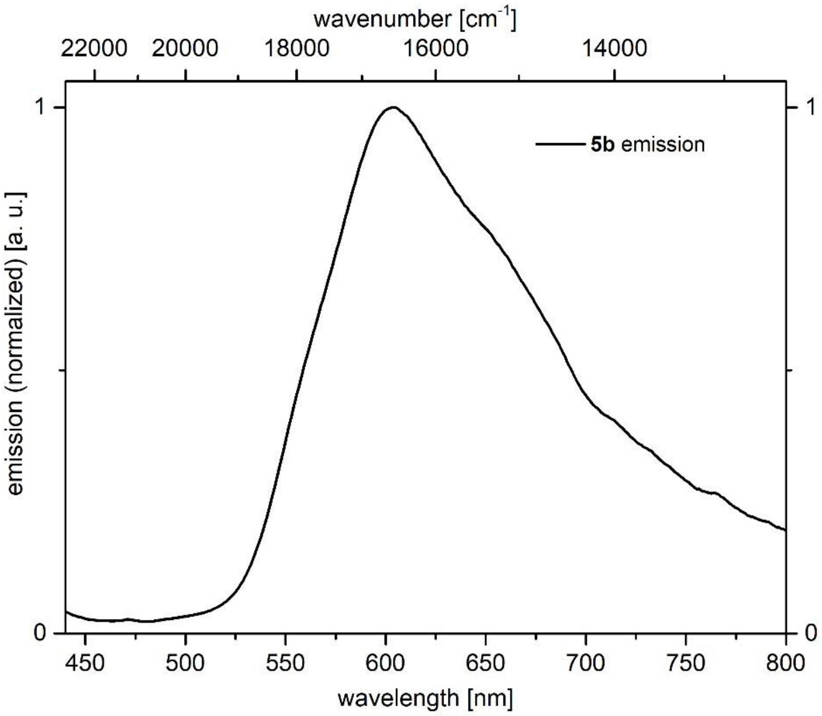

λ_{exc} = 420 nm

10 Emission spectra of α-pyrones 6 in the solid state

10.1 2-Oxo-4,6-diphenyl-2*H*-pyran-3-carbonitrile (6a)

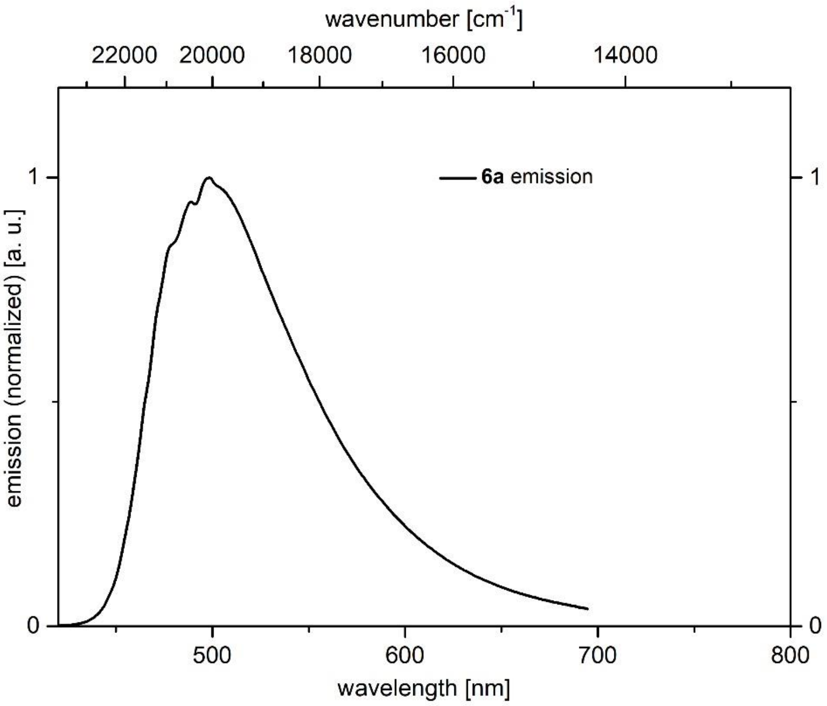

λ_{exc} = 380 nm

10.2 6-(4-Methoxyphenyl)-2-oxo-4-phenyl-2*H*-pyran-3-carbonitrile (6b)

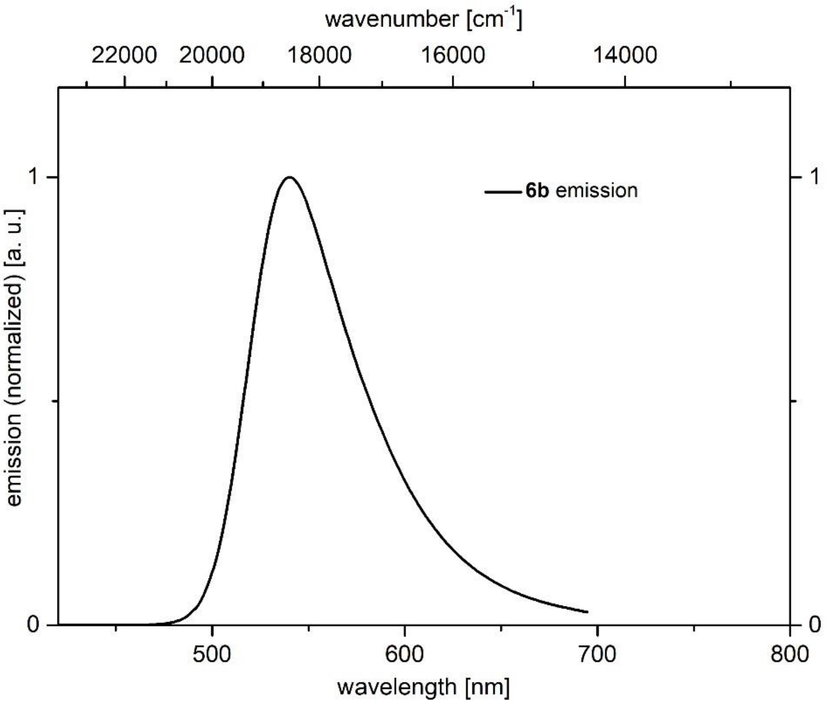

λ_{exc} = 380 nm

10.3 6-[4-(Dimethylamino)phenyl]-2-oxo-4-phenyl-2*H*-pyran-3-carbonitrile (6c)

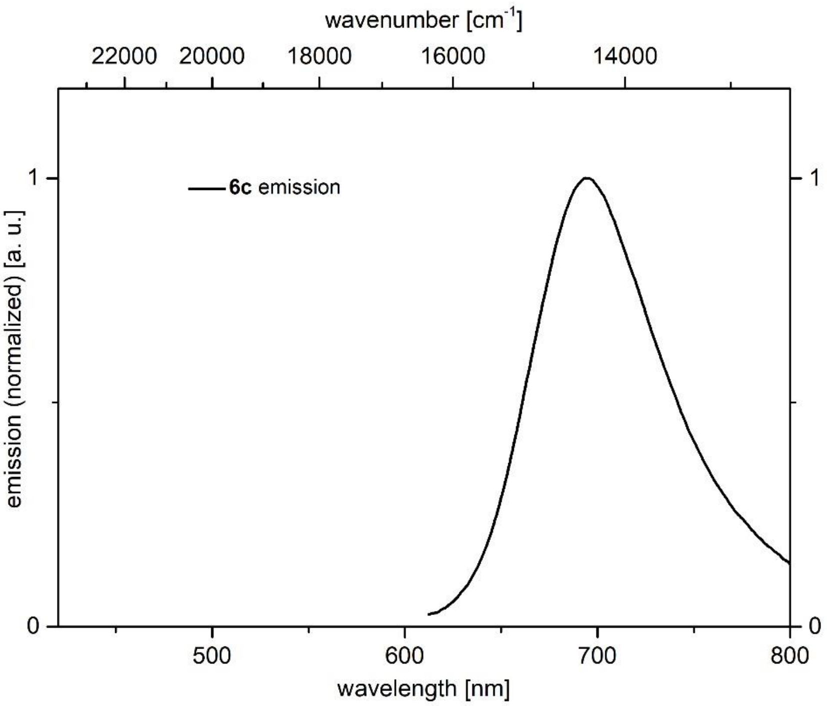

λ_{exc} = 480 nm

10.4 4-(4-Methoxyphenyl)-2-oxo-6-phenyl-2*H*-pyran-3-carbonitrile (6d)

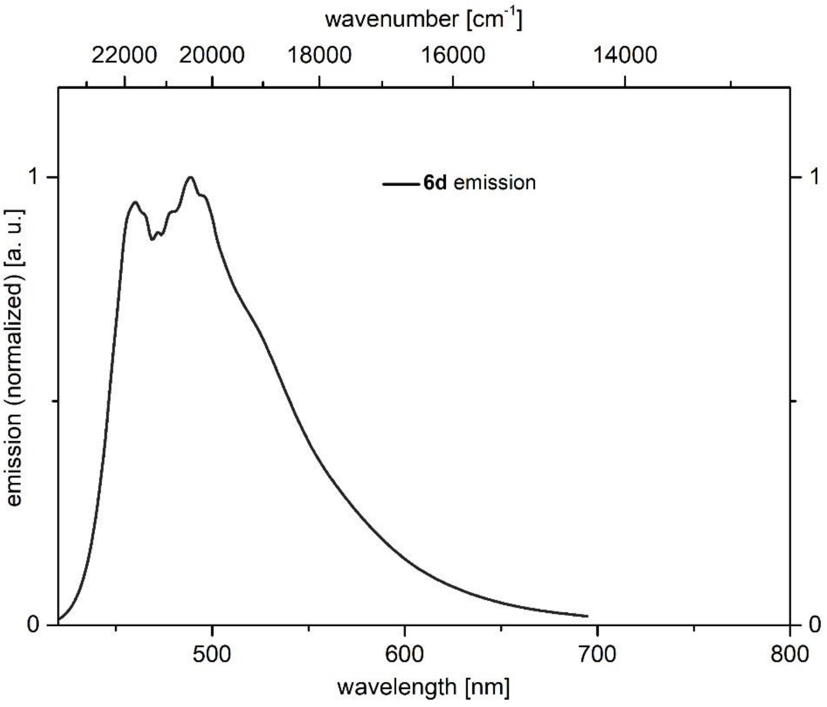

λ_{exc} = 380 nm

10.5 4,6-Bis(4-methoxyphenyl)-2-oxo-2*H*-pyran-3-carbonitrile (6f)

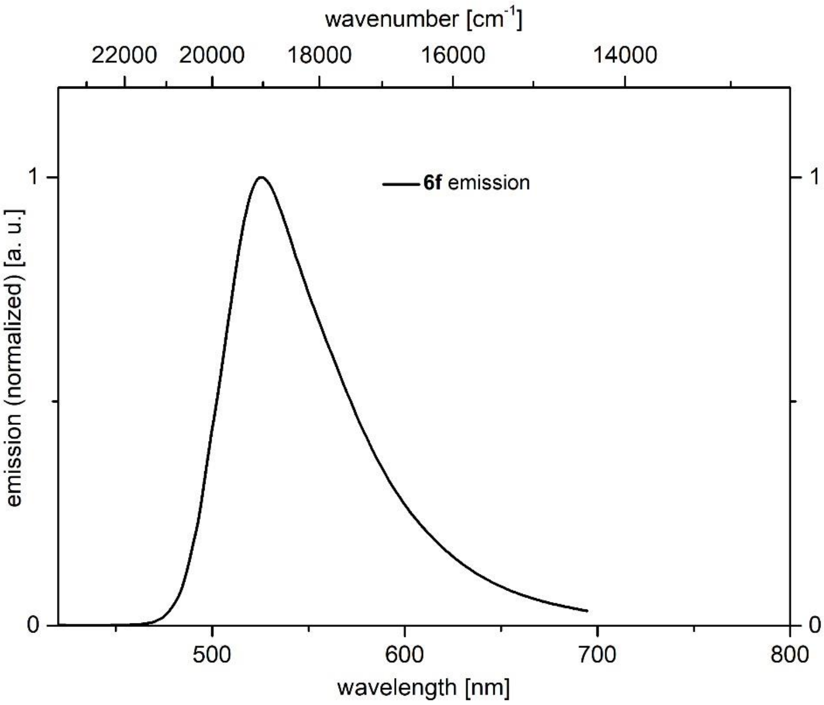

λ_{exc} = 380 nm

11 X-ray structural data of compound 5a

X-Ray crystallography. Single crystals of **5a** suitable for X-ray diffraction were crystallized from methanol at room temperature. A single crystal was mounted on a loop under a polarizing microscope. Data collection: Bruker Kappa APEX2 CCD X-ray diffractometer with microfocus tube, MoKα radiation (λ = 0.71073 Å) at 100 ± 2 K, multilayer mirror system, ω- and ϕ-scan; data collection with APEX2 [13], cell refinement and data reduction with SAINT [14]**Fehler! Textmarke nicht definiert.**, experimental absorption correction with SADABS [15]. *Structure Analysis and Refinement:* The structure of **5a** was solved by direct methods using SHELXS-97; refinement was done by full-matrix least squares on F^2 using the SHELXL-97 program suite [16]. All non-hydrogen positions were refined with anisotropic displacement parameters. Hydrogen atoms on carbon were positioned geometrically (with C–H = 0.95 Å for aromatic CH, C–H = 0.98 Å for CH_3, and C–H = 0.99 for CH_2) and refined using riding models (AFIX 43, 137, and 23 respectively) with $U_{iso}(H) = 1.2\ U_{eq}$ and $U_{iso}(H) = 1.5\ U_{eq}$.q.

Crystal data and details on the structure refinement are given in Table S4. Graphics were drawn with DIAMOND [17]. Analyses on the supramolecular C–H···O, C–H···π, and π–π-stacking interactions were done with PLATON for Windows [18]. CCDC 1944699 contains the supplementary crystallographic data for this paper. These data can be obtained free of charge via http://www.ccdc.cam.ac.uk/conts/retrieving.html.

Crystal data and structure refinement for 5a.

Table S4 Crystal data and refinement details.

Identification code	**5a**	
Empirical formula	$C_{22}H_{18}N_2O_2$	
Formula weight	342.38 g·mol^{-1}	
Temperature	100 (2) K	
Wavelength	0.71073 Å	
Crystal system	Monoclinic	
Space group	$P2_1/c$	
Unit cell dimensions	a = 12.2031 (6) Å	α = 90°
	b = 20.7530 (11) Å	β =106.948 (3)°
	c = 7.1994 (4) Å	γ = 90°
Volume	1744.07 (16) Å^3	
Z	4	
Density (calculated)	1.304 Mg·m^{-3}	
Absorption coefficient	0.084 mm^{-1}	
F(000)	720	
Crystal size	0.02 × 0.02 × 0.02 mm^3	
Theta range for data collection	5.251 to 64.880°	
Index ranges	-15<=h<=15, -25<=k<=25, -8<=l<=8	

Reflections collected	33791
Independent reflections	3470 [R_{int} = 0.0353]
Completeness to theta = 64.88°	99.9 %
Absorption correction	multi-scan (SADABS; Sheldrick, 1996)
Refinement method	Full-matrix least-squares on F^2
Data / restraints / parameters	3470/ 0/ 240
Flack parameter (Absolute structure parameter)	-
Goodness-of-fit on F^2	1.034
Final R indices [I>2sigma(I)]	R1 = 0.0338, wR2 = 0.0939
R indices (all data)	R1 = 0.0403, wR2 = 0.0894
Largest diff. peak and hole (max and min)	0.226 e·Å^{-3} and -0.219 e·Å^{-3}

Bond lengths [Å] and angles [°] for 5a.

O1—C20	1.2330 (14)	C6—C7	1.4013 (17)
O2—C20	1.3474 (14)	C7—C8	1.3834 (17)
O2—C21	1.4456 (14)	C8—C9	1.3882 (18)
N1—C5	1.3596 (15)	C9—C10	1.3867 (18)
N1—C1	1.3639 (15)	C10—C11	1.3889 (17)
N2—C19	1.1500 (16)	C12—C17	1.3971 (17)
C1—C2	1.3696 (17)	C12—C13	1.3985 (17)
C1—C6	1.4728 (16)	C13—C14	1.3889 (18)
C2—C3	1.4133 (16)	C14—C15	1.3872 (19)
C3—C4	1.3777 (17)	C15—C16	1.3885 (19)
C3—C12	1.4840 (16)	C16—C17	1.3903 (17)
C4—C5	1.4115 (17)	C18—C19	1.4195 (16)
C5—C18	1.4184 (16)	C18—C20	1.4333 (16)
C6—C11	1.3962 (16)	C21—C22	1.5046 (16)

Torsion angles [°] for 5a.

C20—O2—C21	116.19 (9)	C9—C10—C11	120.49 (11)
C5—N1—C1	124.46 (10)	C10—C11—C6	120.05 (11)
N1—C1—C2	118.45 (11)	C17—C12—C13	119.04 (11)
N1—C1—C6	116.98 (10)	C17—C12—C3	120.72 (11)
C2—C1—C6	124.57 (11)	C13—C12—C3	120.22 (11)
C1—C2—C3	120.16 (11)	C14—C13—C12	120.30 (12)
C4—C3—C2	119.35 (11)	C15—C14—C13	120.31 (12)
C4—C3—C12	120.65 (11)	C14—C15—C16	119.78 (11)
C2—C3—C12	119.99 (10)	C15—C16—C17	120.24 (12)
C3—C4—C5	120.50 (11)	C16—C17—C12	120.32 (11)

N1—C5—C4	117.06 (10)	C5—C18—C19	118.04 (10)
N1—C5—C18	118.67 (10)	C5—C18—C20	121.70 (10)
C4—C5—C18	124.27 (11)	C19—C18—C20	120.24 (10)
C11—C6—C7	119.15 (11)	N2—C19—C18	177.69 (12)
C11—C6—C1	119.60 (10)	O1—C20—O2	121.95 (11)
C7—C6—C1	121.25 (11)	O1—C20—C18	125.71 (11)
C8—C7—C6	120.25 (11)	O2—C20—C18	112.34 (10)
C7—C8—C9	120.40 (11)	O2—C21—C22	106.25 (9)
C10—C9—C8	119.65 (11)		
C5—N1—C1—C2	0.32 (17)	C4—C3—C12—C17	38.07 (17)
C5—N1—C1—C6	-179.93 (10)	C2—C3—C12—C17	-141.67 (12)
N1—C1—C2—C3	0.79 (17)	C4—C3—C12—C13	-143.65 (12)
C6—C1—C2—C3	-178.93 (11)	C2—C3—C12—C13	36.61 (17)
C1—C2—C3—C4	-0.75 (17)	C17—C12—C13—C14	0.14 (18)
C1—C2—C3—C12	179.00 (11)	C3—C12—C13—C14	-178.17 (11)
C2—C3—C4—C5	-0.37 (17)	C12—C13—C14—C15	0.57 (19)
C12—C3—C4—C5	179.88 (10)	C13—C14—C15—C16	-0.9 (2)
C1—N1—C5—C4	-1.40 (17)	C14—C15—C16—C17	0.51 (19)
C1—N1—C5—C18	178.04 (11)	C15—C16—C17—C12	0.20 (19)
C3—C4—C5—N1	1.40 (17)	C13—C12—C17—C16	-0.52 (18)
C3—C4—C5—C18	-178.02 (11)	C3—C12—C17—C16	177.79 (11)
N1—C1—C6—C11	153.78 (11)	N1—C5—C18—C19	-179.78 (10)
C2—C1—C6—C11	-26.48 (18)	C4—C5—C18—C19	-0.37 (17)
N1—C1—C6—C7	-26.90 (16)	N1—C5—C18—C20	1.96 (17)
C2—C1—C6—C7	152.83 (12)	C4—C5—C18—C20	-178.64 (11)
C11—C6—C7—C8	-0.13 (18)	C21—O2—C20—O1	6.53 (17)
C1—C6—C7—C8	-179.45 (11)	C21—O2—C20—C18	-172.60 (10)
C6—C7—C8—C9	0.56 (19)	C5—C18—C20—O1	0.19 (19)
C7—C8—C9—C10	-0.31 (19)	C19—C18—C20—O1	-178.04 (11)
C8—C9—C10—C11	-0.37 (19)	C5—C18—C20—O2	179.28 (10)
C9—C10—C11—C6	0.79 (18)	C19—C18—C20—O2	1.05 (16)
C7—C6—C11—C10	-0.53 (18)	C20—O2—C21—C22	178.28 (10)
C1—C6—C11—C10	178.80 (11)		

Hydrogen-bond geometry (Å, º) for 5a.

D—H⋯*A*	*D*—H	H⋯*A*	*D*⋯*A*	*D*—H⋯*A*
N1—H1⋯O1	0.899 (16)	1.868 (16)	2.6237 (13)	140.3 (14)

Significant π-stacking show rather short centroid-centroid contacts (<3.8 Å), near parallel ring planes (α < 10° to ≈ 0° or even exactly 0° by symmetry), small slip angles (β, γ < 25°) and vertical displacements

(slippage <1.5 Å) which translate into a sizable overlap of the aryl-plane areas (Scheme S1). Significant intermolecular C–H···π contacts are less than 2.7 Å for the (C-)H···ring centroid distances with H-perp below 2.6–2.7 Å and C–H···Cg > 145°.

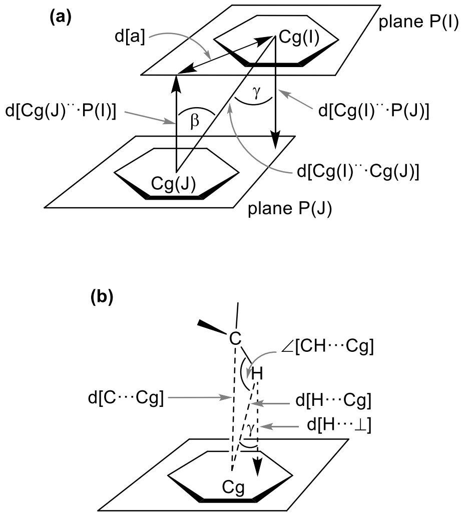

Scheme S1 Graphical presentation of the parameters used for the description of (a) π–π stacking and (b) CH-π interactions.

Table S5. Packing Analysis for 1*H*-pyridine **5a** for possible π-π interactions.[a]

==

Analysis of Short Ring-Interactions with Cg-Cg Distances < 6.0 Ang., Alpha < 20.000 Deg. and Beta < 60.0 Deg.

==

- Cg(I) = Plane number I (= ring number in () above)
- Alpha = Dihedral Angle between Planes I and J (Deg)
- Beta = Angle Cg(I)-->Cg(J) or Cg(I)-->Me vector and normal to plane I (Deg)
- Gamma = Angle Cg(I)-->Cg(J) vector and normal to plane J (Deg)
- Cg-Cg = Distance between ring Centroids (Ang.)
- CgI_Perp = Perpendicular distance of Cg(I) on ring J (Ang.)
- CgJ_Perp = Perpendicular distance of Cg(J) on ring I (Ang.)
- Slippage = Distance between Cg(I) and Perpendicular Projection of Cg(J) on Ring I (Ang).
- P,Q,R,S = J-Plane Parameters for Carth. Coord. (Xo, Yo, Zo)

Cg(I) Res(I) Cg(J) [ARU(J)]	Cg-Cg	Alpha	Beta	Gamma	CgI_Perp	CgJ_Perp	Slippage
Cg(1) [1] -> Cg(1) [4564.01]	4.6623(7)	3.61(5)	41.6	45.0	3.2977(5)	-3.4842(5)	3.098
Cg(1) [1] -> Cg(1) [4565.01]	4.6623(7)	3.61(5)	45.0	41.6	-3.4842(5)	3.2977(5)	3.296
Cg(3) [1] -> Cg(2) [3666.01]	4.6922(8)	33.29(6)	29.4	62.7	2.1523(5)	4.0861(5)	
Cg(3) [1] -> Cg(3) [4564.01]	4.9699(8) 7	8.57(6)	11.6	83.9	0.5289(5)	-4.8682(5)	
Min or Max	4.662	0.0	11.6	83.9	-4.463	-4.868	

[4564] = X,3/2-Y,-1/2+Z
[4565] = X,3/2-Y,1/2+Z
[3666] = 1-X,1-Y,1-Z

[a] The Table presents a selection of the Cg-Cg distances calculated by PLATON [18], here chosen according to the criteria of centroid-centroid contacts (<3.8 Å), near parallel ring planes (α < 10° to ~0° or even exactly 0° by symmetry), small slip angles (β, γ < 25°) (Scheme).

The Cg(I) refer to the Ring Centre-of-Gravity numbers with atoms#

Cg(1) = N1-C1-C2-C3-C4-C5
Cg(2) = C6-C7-C8-C9-C10-C11
Cg(3) = C12-C13-C14-C15-C16-C17

Table S6. Packing Analysis for 1*H*-pyridine **5a** for possible C-H-π interactions.

==

Analysis of X-H...Cg(Pi-Ring) Interactions (H..Cg < 3.0 Ang. - Gamma < 30.0 Deg)

==

- Cg(J) = Center of gravity of ring J (Plane number above)
- H-Perp = Perpendicular distance of H to ring plane J
- Gamma = Angle between Cg-H vector and ring J normal
- X-H..Cg = X-H-Cg angle (degrees)
- X..Cg = Distance of X to Cg (Angstrom)
- X-H, Pi = Angle of the X-H bond with the Pi-plane (i.e.' Perpendicular = 90 degrees, Parallel = 0 degrees)

X--H(I)	Res(I)	Cg(J)	[ARU(J)]	H..Cg	H-Perp	Gamma	X-H..Cg	X..Cg	X-H,Pi
C(9) -H(9)	[1] ->	Cg(3)	[3666.01]	2.84	2.81	8.29	129	3.5232(14)	33
C(17) -H(17)	[1] ->	Cg(3)	[4565.01]	2.78	-2.78	1.37	152	3.6474(14)	63
C(22) -H(22B)	[1] ->	Cg(2)	[4565.01]	2.61	-2.61	1.98	162	3.5603(14)	74
	Min or Max	2.610		-2.779	1.4	162.00	3.523	74.00	

[3666] = 1-X,1-Y,1-Z

[4565] = X,3/2-Y,1/2+Z

12 Quantum chemical calculations

12.1 Computed xyz-coordinates of 1*H*-pyridines 5

Table S7 TDDFT Calculations (B3LYP/6-311G**) of the absorption maxima for 1*H*-pyridines **5**.

Compound	R^1	R^2	$\lambda_{max,abs}$ exp. [nm] [a]	$\lambda_{max,abs}$ calcd. [nm]	Oscillator strength	most dominant contributions
**5a**	H	H	420	417	0.197	HOMO → LUMO (98 %)
			320	321	0.359	HOMO → LUMO+1 (95 %)
			282	293	0.590	HOMO-1 → LUMO (95 %)<br>HOMO-2 → LUMO (3 %)
**5b**	CF_3	H	423	426	0.167	HOMO → LUMO (98 %)
			326	333	0.334	HOMO → LUMO+1 (98 %)
			273	299	0.424	HOMO-1 → LUMO (98 %)
**5c**	CN	H	433	446	0.167	HOMO → LUMO (98 %)
			334	345	0.287	HOMO → LUMO+1 (98 %)
			279	310	0.403	HOMO-1 → LUMO (98 %)
**5d**	H	CF_3	428	429	0.188	HOMO → LUMO (98 %)
			322	325	0.256	HOMO → LUMO+1 (95 %)<br>HOMO-1 → LUMO (2 %)
			275	290	0.382	HOMO-1 → LUMO (77 %)<br>HOMO → LUMO+2 (11 %)<br>HOMO → LUMO+3 (10 %)
**5e**	H	CN	434	445	0.176	HOMO → LUMO (98 %)
			322	337	0.141	HOMO → LUMO+1 (95 %)
			284	300	0.333	HOMO-1 → LUMO (58 %)
				295	0.874	HOMO → LUMO+2 (46 %)<br>HOMO-1 → LUMO (39 %)<br>HOMO-2 → LUMO (12 %)

**5f**	OMe	CF_3	429	430	0.241	HOMO → LUMO (98 %)
			306	319	0.437	HOMO → LUMO+1 (77 %)<br>HOMO-1 → LUMO (18 %)
			261	274	0.462	HOMO-1 → LUMO+1 (67 %)<br>HOMO-2 → LUMO (23 %)<br>HOMO → LUMO+3 (5 %)<br>HOMO-3 → LUMO (3 %)
**5g**	CF_3	OMe	420	426	0.161	HOMO → LUMO (98 %)
			324	349	0.368	HOMO-1 → LUMO (98 %)
				334	0.469	HOMO → LUMO+1 (98 %)
			260	291	0.155	HOMO-1 → LUMO+1 (92 %)
				276	0.248	HOMO → LUMO+3 (70 %)<br>HOMO-2 → LUMO (10 %)<br>HOMO-3 → LUMO (10 %)<br>HOMO → LUMO+4 (7 %)
				268	0.162	HOMO → LUMO+4 (44 %)<br>HOMO-3 → LUMO (35 %)<br>HOMO → LUMO+3 (14 %)<br>HOMO-2 → LUMO (5 %)

[a] Recorded in dichloromethane, T = 293 K, c_0(**5**) = 10^{-6} M.

12.1.1 Ethyl (*Z*)-2-cyano-2-(4,6-diphenyl-1*H*-pyridin-2-ylidene)acetate (5a) (B3LYP)

C 0 -1.232643 -1.215146 -0.071483
C 0 -1.954326 -0.037827 0.002318
C 0 -1.253688 1.196853 0.014665
C 0 0.118041 1.205183 -0.045965
N 0 0.783393 0.014614 -0.125729
C 0 0.182506 -1.215846 -0.137496
C 0 0.953526 -2.410898 -0.227966
C 0 2.397373 -2.534956 -0.315934
O 0 3.003702 -3.591154 -0.387369
C 0 -3.434999 -0.055908 0.082958
C 0 0.936517 2.435199 0.002702
C 0 -4.197323 0.939076 -0.548296
C 0 -5.587943 0.917057 -0.482059
C 0 -6.240547 -0.092482 0.225091
C 0 -5.493391 -1.082660 0.863023
C 0 -4.103383 -1.067028 0.790702
C 0 2.145494 2.467077 0.714245
C 0 2.900865 3.635573 0.767002
C 0 2.462616 4.784418 0.109507
C 0 1.262100 4.760884 -0.600709
C 0 0.502974 3.595848 -0.654804
C 0 0.256352 -3.639951 -0.235825
N 0 -0.334020 -4.640034 -0.240145
O 0 3.050135 -1.327855 -0.320276
C 0 5.131300 -1.427396 0.984720
C 0 4.505096 -1.339825 -0.395249
H 0 -1.742464 -2.166951 -0.106421
H 0 -1.783171 2.130523 0.131849
H 0 1.798590 0.009634 -0.212588
H 0 -3.704479 1.720109 -1.115234
H 0 -6.161470 1.686352 -0.986229
H 0 -7.322995 -0.106540 0.280040
H 0 -5.992376 -1.865249 1.422856
H 0 -3.534238 -1.830621 1.307497
H 0 2.484587 1.591945 1.257226
H 0 3.826796 3.650148 1.329919
H 0 3.052385 5.692750 0.150792
H 0 0.920015 5.648066 -1.120674
H 0 -0.417170 3.579821 -1.226515
H 0 6.219598 -1.373431 0.891309
H 0 4.802162 -0.599569 1.617347
H 0 4.877124 -2.369656 1.473249
H 0 4.809275 -2.170564 -1.030118
H 0 4.752411 -0.400218 -0.888897
SCF Done: E(RB3LYP) = -1109.45629970 A.U. after 16 cycles
Sum of electronic and zero-point Energies= -1109.088838
Sum of electronic and thermal Energies= -1109.066364
Sum of electronic and thermal Enthalpies= -1109.065419
Sum of electronic and thermal Free Energies= -1109.142971

12.1.2 Ethyl (*Z*)-2-cyano-2-{4-phenyl-6-[4-(trifluoromethyl)phenyl]-1*H*-pyridin-2-ylidene}acetate (5b) (B3LYP)

C 0	2.666619	-0.277697	-0.066843
C 0	2.118599	-1.546475	-0.011661
C 0	0.704879	-1.681258	0.013327
C 0	-0.091708	-0.562882	-0.020795
N 0	0.495421	0.666726	-0.073479
C 0	1.847051	0.874665	-0.097116
C 0	2.347598	2.207860	-0.129579
C 0	1.467460	3.353241	-0.132277
O 0	0.231786	3.288001	-0.117031
C 0	2.982925	-2.750791	0.012385
C 0	-1.570237	-0.611814	-0.038305
C 0	2.621008	-3.875258	0.770191
C 0	3.438850	-5.001584	0.803743
C 0	4.625613	-5.028473	0.071783
C 0	4.991922	-3.919710	-0.691248
C 0	4.180231	-2.789273	-0.718861
C 0	-2.310723	0.288637	-0.816410
C 0	-3.699268	0.225083	-0.841501
C 0	-4.362381	-0.742438	-0.087580
C 0	-3.637371	-1.642663	0.695435
C 0	-2.250490	-1.576682	0.718108
C 0	3.743568	2.406447	-0.150938
N 0	4.896820	2.544054	-0.169363
O 0	2.123672	4.529193	-0.158859
C 0	1.026588	6.192888	1.278026
C 0	1.338071	5.752207	-0.141674
C 0	-5.865058	-0.787449	-0.072323
F 0	-6.410922	-0.266232	-1.193621
F 0	-6.383226	-0.082847	0.970162
F 0	-6.340483	-2.048879	0.050166
H 0	3.738573	-0.140112	-0.059670
H 0	0.246292	-2.658682	-0.001074
H 0	-0.062080	1.531332	-0.061655
H 0	1.710480	-3.861947	1.357738
H 0	3.150575	-5.856219	1.404878
H 0	5.259527	-5.907406	0.094372
H 0	5.907664	-3.936467	-1.270851
H 0	4.466697	-1.942490	-1.331273
H 0	-1.809146	1.025721	-1.431538
H 0	-4.258180	0.917575	-1.457277
H 0	-4.150081	-2.390103	1.287438
H 0	-1.696431	-2.266292	1.342298
H 0	0.500095	7.151142	1.252276
H 0	0.389158	5.466858	1.786112
H 0	1.944823	6.323480	1.855304
H 0	1.971651	6.477017	-0.652205
H 0	0.430233	5.595715	-0.723538

SCF Done: E(RB3LYP) = -1446.61070520 A.U. after 15 cycles

Sum of electronic and zero-point Energies= -1446.242058

Sum of electronic and thermal Energies= -1446.215987
Sum of electronic and thermal Enthalpies= -1446.215042
Sum of electronic and thermal Free Energies= -1446.302635

12.1.3 Ethyl (*Z*)-2-cyano-2-[6-(4-cyanophenyl)-4-phenyl-1*H*-pyridin-2-ylidene]acetate (5c) (B3LYP)

C	0	-1.968267	0.377945	0.034528
C	0	-1.941752	-1.004514	0.014153
C	0	-0.684130	-1.664598	-0.012575
C	0	0.476335	-0.930149	-0.023554
N	0	0.398581	0.431283	-0.009181
C	0	-0.773504	1.135448	0.016170
C	0	-0.731536	2.558591	0.018860
C	0	0.517858	3.283824	-0.006054
O	0	1.636875	2.755090	-0.028547
C	0	-3.198126	-1.791728	0.031923
C	0	1.826733	-1.533471	-0.034614
C	0	-3.273418	-3.003712	0.735522
C	0	-4.458282	-3.734313	0.765217
C	0	-5.583814	-3.273532	0.082762
C	0	-5.518386	-2.074590	-0.627134
C	0	-4.337544	-1.337904	-0.650514
C	0	2.885251	-0.937863	0.668006
C	0	4.148010	-1.513167	0.666735
C	0	4.371650	-2.700020	-0.044929
C	0	3.321041	-3.302301	-0.752770
C	0	2.062465	-2.719933	-0.745236
C	0	-1.947433	3.272666	0.034543
N	0	-2.961753	3.838447	0.048079
O	0	0.363145	4.619762	-0.003932
C	0	1.137203	6.882629	-0.027262
C	0	1.567979	5.430944	-0.032088
C	0	5.671645	-3.296205	-0.050762
N	0	6.721877	-3.779188	-0.055152
H	0	-2.907873	0.909636	0.084498
H	0	-0.630487	-2.742945	-0.019071
H	0	1.240745	1.021146	-0.051458
H	0	-2.413071	-3.365738	1.286049
H	0	-4.502919	-4.661232	1.325291
H	0	-6.504616	-3.844997	0.102820
H	0	-6.385668	-1.714411	-1.168304
H	0	-4.294378	-0.417704	-1.221127
H	0	2.724906	-0.034454	1.243542
H	0	4.956285	-1.051857	1.219457
H	0	3.496093	-4.212934	-1.311126
H	0	1.264922	-3.178656	-1.315579
H	0	2.022711	7.522885	-0.050030
H	0	0.524699	7.113292	-0.901676
H	0	0.565477	7.119894	0.872622
H	0	2.175082	5.183251	0.840873
H	0	2.137127	5.177944	-0.928741

SCF Done: E(RB3LYP) = -1201.73140651   A.U. after   16 cycles
Sum of electronic and zero-point Energies=   -1201.365273
Sum of electronic and thermal Energies=   -1201.340990
Sum of electronic and thermal Enthalpies=   -1201.340045
Sum of electronic and thermal Free Energies=   -1201.421926

12.1.4 Ethyl (*Z*)-2-cyano-2-{4-phenyl-6-[4-(trifluoromethyl)phenyl]-1*H*-pyridin-2-ylidene}acetate (5d) (B3LYP)

C 0	-0.016080	-1.189245	-0.090330
C 0	-0.715981	0.001134	-0.080925
C 0	-0.001168	1.226326	-0.093459
C 0	1.371876	1.212212	-0.119789
N 0	2.019326	0.009420	-0.133949
C 0	1.401719	-1.212916	-0.121598
C 0	2.154527	-2.420246	-0.145006
C 0	3.599739	-2.567959	-0.174161
O 0	4.190113	-3.634467	-0.202791
C 0	-2.199682	0.004860	-0.037328
C 0	2.207766	2.431229	-0.122104
C 0	-2.887483	0.949647	0.737885
C 0	-4.276031	0.949162	0.792409
C 0	-4.998128	0.007516	0.058002
C 0	-4.329401	-0.936231	-0.722108
C 0	-2.940178	-0.935591	-0.767123
C 0	3.402797	2.484611	0.611243
C 0	4.175231	3.642989	0.614533
C 0	3.768502	4.759451	-0.115085
C 0	2.582432	4.713754	-0.847981
C 0	1.805591	3.559177	-0.852303
C 0	1.438435	-3.638802	-0.148935
N 0	0.832667	-4.629404	-0.151674
O 0	4.270299	-1.371863	-0.173058
C 0	6.290177	-1.518953	1.222896
C 0	5.727410	-1.408295	-0.182600
C 0	-6.497450	-0.021091	0.151928
F 0	-7.027476	1.211348	0.333761
F 0	-6.927561	-0.777380	1.199062
F 0	-7.076998	-0.540335	-0.954345
H 0	-0.540129	-2.133263	-0.051585
H 0	-0.522329	2.171877	-0.076548
H 0	3.036849	-0.013310	-0.185813
H 0	-2.337817	1.676473	1.323099
H 0	-4.791480	1.679801	1.402769
H 0	-4.886878	-1.661173	-1.301118
H 0	-2.431726	-1.658267	-1.393221
H 0	3.717843	1.636471	1.208755
H 0	5.089835	3.675343	1.194897
H 0	4.371927	5.659676	-0.112604
H 0	2.265131	5.575012	-1.424146
H 0	0.897321	3.524581	-1.441822
H 0	7.382094	-1.478040	1.178254

H 0	5.943423	-0.694025	1.849874
H 0	6.002623	-2.463070	1.688354
H 0	6.045685	-2.238142	-0.811863
H 0	6.011000	-0.468543	-0.655786

SCF Done: E(RB3LYP) = -1446.60544658 A.U. after 16 cycles
Sum of electronic and zero-point Energies= -1446.232486
Sum of electronic and thermal Energies= -1446.206293
Sum of electronic and thermal Enthalpies= -1446.205349
Sum of electronic and thermal Free Energies= -1446.293524

12.1.5 Ethyl (*Z*)-2-cyano-2-[6-(4-cyanophenyl)-4-phenyl-1*H*-pyridin-2-ylidene]acetate (5e) (B3LYP)

C 0	-0.722004	-1.199185	-0.064412
C 0	-1.427226	-0.012231	-0.044951
C 0	-0.719344	1.216883	-0.064821
C 0	0.653391	1.209732	-0.108521
N 0	1.306627	0.010190	-0.131227
C 0	0.695740	-1.215522	-0.111810
C 0	1.453769	-2.418879	-0.143289
C 0	2.899686	-2.559306	-0.185105
O 0	3.494818	-3.622900	-0.216520
C 0	-2.909912	-0.016391	0.018628
C 0	1.482400	2.433253	-0.120809
C 0	-3.591211	0.926518	0.803263
C 0	-4.976730	0.920696	0.880007
C 0	-5.709417	-0.031446	0.156793
C 0	-5.040974	-0.975137	-0.636648
C 0	-3.654871	-0.964183	-0.699306
C 0	2.689475	2.491096	0.592240
C 0	3.455251	3.653850	0.586406
C 0	3.029782	4.770361	-0.132339
C 0	1.831649	4.720310	-0.845070
C 0	1.061485	3.561311	-0.840301
C 0	0.743451	-3.640867	-0.140762
N 0	0.142121	-4.634095	-0.138237
O 0	3.563732	-1.359827	-0.192552
C 0	5.594037	-1.491063	1.189707
C 0	5.021041	-1.388402	-0.212224
C 0	-7.137762	-0.038497	0.227244
N 0	-8.292472	-0.043121	0.284359
H 0	-1.240490	-2.145978	-0.020074
H 0	-1.244712	2.159894	-0.040648
H 0	2.323667	-0.007524	-0.194475
H 0	-3.036213	1.655425	1.380633
H 0	-5.491694	1.644253	1.499030
H 0	-5.606358	-1.705293	-1.201498
H 0	-3.150092	-1.686134	-1.329044
H 0	3.019715	1.642926	1.181423
H 0	4.379409	3.689498	1.151206
H 0	3.627959	5.674066	-0.136976
H 0	1.499734	5.581640	-1.412816

H 0 0.143563 3.523614 -1.414511
H 0 6.685335 -1.443536 1.137394
H 0 5.246478 -0.666273 1.816450
H 0 5.315658 -2.435451 1.660196
H 0 5.339342 -2.218719 -0.840836
H 0 5.296136 -0.448797 -0.690670
SCF Done: E(RB3LYP) = -1201.72541521 A.U. after 16 cycles
Sum of electronic and zero-point Energies= -1201.354864
Sum of electronic and thermal Energies= -1201.330553
Sum of electronic and thermal Enthalpies= -1201.329609
Sum of electronic and thermal Free Energies= -1201.411604

12.1.6 Ethyl (*Z*)-2-cyano-2-[4-(4-methoxyphenyl]-6-[4-(trifluoromethyl)phenyl]pyridin-2(1*H*)-ylidene)acetate (5f) (B3LYP)

C 0 -0.485533 -1.464174 -0.086645
C 0 -1.052246 -0.202316 -0.038051
C 0 -0.211257 0.935229 -0.002889
C 0 1.158609 0.781862 -0.034850
N 0 1.667760 -0.482631 -0.094205
C 0 0.917456 -1.626201 -0.116062
C 0 1.573359 -2.890633 -0.173965
C 0 3.012204 -3.004172 -0.210696
O 0 3.792855 -2.043976 -0.196842
C 0 -2.527786 -0.039181 -0.008622
C 0 2.115527 1.901057 -0.001065
C 0 -3.122569 0.956105 0.780438
C 0 -4.503323 1.108197 0.811550
C 0 -5.310942 0.270032 0.041281
C 0 -4.735691 -0.724177 -0.750212
C 0 -3.354016 -0.875389 -0.772580
C 0 3.378013 1.766647 0.606757
C 0 4.266473 2.826511 0.646214
C 0 3.922861 4.059877 0.073256
C 0 2.672922 4.210189 -0.539151
C 0 1.786966 3.137844 -0.569871
C 0 0.784483 -4.058771 -0.206302
N 0 0.110777 -5.004882 -0.231199
O 0 3.444622 -4.279306 -0.270338
C 0 5.454921 -4.599285 1.106258
C 0 4.878486 -4.515105 -0.296567
C 0 -6.805238 0.410706 0.108601
F 0 -7.195395 1.697451 0.270284
F 0 -7.335495 -0.279952 1.155273
F 0 -7.421828 -0.051421 -1.002645
O 0 4.858986 5.036939 0.161205
C 0 4.570594 6.323932 -0.395229
H 0 -1.104919 -2.349632 -0.081477
H 0 -0.632282 1.927729 0.052903
H 0 2.680789 -0.647120 -0.152493
H 0 -2.507516 1.603457 1.393202
H 0 -4.946948 1.876976 1.431636

H 0 -5.358425 -1.369627 -1.356134
H 0 -2.916312 -1.634762 -1.408714
H 0 3.664202 0.835309 1.081638
H 0 5.232221 2.722987 1.125582
H 0 2.385139 5.144506 -1.000665
H 0 0.835155 3.268212 -1.070466
H 0 6.517578 -4.850888 1.046237
H 0 5.358865 -3.646765 1.630789
H 0 4.952490 -5.376090 1.687295
H 0 4.977798 -5.463801 -0.823453
H 0 5.354417 -3.728201 -0.881033
H 0 5.450896 6.932080 -0.199545
H 0 4.404640 6.257280 -1.473893
H 0 3.699585 6.774290 0.088441
SCF Done: E(RB3LYP) = -1561.17095519 A.U. after 16 cycles
Sum of electronic and zero-point Energies= -1560.768204
Sum of electronic and thermal Energies= -1560.739497
Sum of electronic and thermal Enthalpies= -1560.738552
Sum of electronic and thermal Free Energies= -1560.832041

12.1.7 Ethyl (*Z*)-2-cyano-2-{4-(4-methoxyphenyl)-6-[4-(trifluoromethyl)phenyl]pyridin-2(1*H*)-ylidene}acetate (5g) (B3LYP)

C 0 1.948937 1.103526 -0.129865
C 0 1.999415 -0.280452 -0.086213
C 0 0.775288 -1.003513 -0.072946
C 0 -0.422229 -0.332389 -0.102550
N 0 -0.417439 1.030667 -0.146280
C 0 0.717251 1.794368 -0.159827
C 0 0.599896 3.214070 -0.224973
C 0 -0.683922 3.872004 -0.280793
O 0 -1.774358 3.286155 -0.275904
C 0 3.290114 -0.998286 -0.044472
C 0 -1.738836 -1.006045 -0.054870
C 0 3.439161 -2.261359 -0.647646
C 0 4.650775 -2.930123 -0.623809
C 0 5.759265 -2.360897 0.019908
C 0 5.630478 -1.109673 0.633502
C 0 4.408332 -0.444556 0.592367
C 0 -2.811419 -0.441195 0.648451
C 0 -4.042674 -1.085409 0.698893
C 0 -4.215482 -2.301543 0.039930
C 0 -3.155758 -2.875607 -0.665491
C 0 -1.927518 -2.230255 -0.712833
C 0 1.777334 3.989401 -0.242227
N 0 2.762611 4.604898 -0.253073
O 0 -0.592010 5.214913 -0.348174
C 0 -2.329576 6.310587 0.999150
C 0 -1.823715 5.984738 -0.395557
O 0 6.902128 -3.093553 -0.002822
C 0 8.073354 -2.564006 0.626074
C 0 -5.527930 -3.029953 0.121913

F	0	-6.558363	-2.213391	0.432793
F	0	-5.508506	-4.004084	1.072077
F	0	-5.846833	-3.644948	-1.041839
H	0	2.858371	1.685669	-0.171707
H	0	0.774535	-2.079253	0.019573
H	0	-1.289509	1.572027	-0.210629
H	0	2.605121	-2.715583	-1.169305
H	0	4.763120	-3.894627	-1.104150
H	0	6.463200	-0.651181	1.148615
H	0	4.326561	0.512927	1.092835
H	0	-2.687909	0.492342	1.183703
H	0	-4.859993	-0.640404	1.251104
H	0	-3.290891	-3.813508	-1.189226
H	0	-1.119651	-2.668836	-1.284773
H	0	-3.213950	6.949540	0.923356
H	0	-2.608670	5.404320	1.540028
H	0	-1.569925	6.845888	1.573467
H	0	-1.547243	6.888500	-0.938076
H	0	-2.563351	5.431142	-0.973186
H	0	8.850991	-3.309450	0.474718
H	0	8.375316	-1.620694	0.162863
H	0	7.911653	-2.416694	1.697498

SCF Done: E(RB3LYP) = -1561.17022617 A.U. after 15 cycle
Sum of electronic and zero-point Energies= -1560.767625
Sum of electronic and thermal Energies= -1560.738901
Sum of electronic and thermal Enthalpies= -1560.737957
Sum of electronic and thermal Free Energies= -1560.831290

12.2 Computed xyz-coordinates of 1*H*-pyridines 8

Table S8. TDDFT Calculations (B3LYP/6-311G**) of the absorption maxima for 1*H*-pyridines **8**.

**8a**	H	H	CO_2Et	402	417	0.201	HOMO → LUMO (98 %)
				315	324	0.432	HOMO → LUMO+1 (95 %)<br>HOMO-2 → LUMO (3 %)
				290	274	0.297	HOMO-1 → LUMO (95 %)<br>HOMO-2 → LUMO (2 %)
**8b**	OMe	H	CO_2Et	419	409	0.266	HOMO → LUMO (98 %)
				307	327	0.256	HOMO-1 → LUMO (54 %)<br>HOMO → LUMO+1 (42 %)
					311	0.571	HOMO → LUMO+1 (52 %)<br>HOMO-1 → LUMO (42 %)
				261	287	0.128	HOMO-2 → LUMO (79 %)<br>HOMO → LUMO+2 (12 %)<br>HOMO → LUMO+3 (2 %)
					275	0.244	HOMO-1 → LUMO+1 (90 %)<br>HOMO → LUMO+2 (4 %)<br>HOMO → LUMO+3 (3 %)

[a] Recorded in dichloromethane, T = 293 K, c_0(**8**) = 10^{-6} M.

12.2.1 Ethyl (*Z*)-2-(1-cyano-2-ethoxy-2-oxoethylidene)-4,6-diphenyl-1,2-dihydropyridine-3-carboxylate (8a) (B3LYP)

C	0.841943	-0.363401	-0.235547
C	1.427199	0.893776	-0.166266
C	0.615740	2.046699	-0.022427
C	-0.751225	1.922191	0.025786
N	-1.285787	0.674785	-0.032471
C	-0.580154	-0.499432	-0.138269
C	-1.326065	-1.709624	-0.120814
C	-2.776451	-1.693443	-0.077862
O	-3.480712	-0.674050	-0.049082
C	2.901793	1.102605	-0.251107
C	-1.687268	3.063618	0.111742
C	3.560928	1.774872	0.787794
C	4.930608	2.012729	0.720783
C	5.658313	1.598220	-0.394106
C	5.008914	0.940843	-1.436553

C	3.639852	0.689273	-1.367537
C	-2.955332	3.003927	-0.485713
C	-3.823183	4.089127	-0.408153
C	-3.442628	5.246952	0.267100
C	-2.186446	5.313990	0.868860
C	-1.315971	4.232214	0.793280
C	1.718815	-1.569934	-0.471173
O	1.947302	-2.008871	-1.571625
C	-0.695323	-2.976116	-0.085502
N	-0.203716	-4.024043	-0.029215
O	-3.322814	-2.918883	-0.073016
C	-5.135770	-4.468772	-0.034794
C	-4.767406	-2.999235	-0.031075
O	2.226204	-2.037264	0.669786
C	3.472691	-3.611465	1.974950
C	3.034748	-3.247515	0.572617
H	1.072350	3.025213	-0.025840
H	-2.308653	0.523747	0.032478
H	3.000242	2.092402	1.660092
H	5.429099	2.520243	1.538963
H	6.724269	1.787983	-0.449484
H	5.566167	0.622309	-2.310200
H	3.143609	0.178050	-2.182441
H	-3.264818	2.118771	-1.029122
H	-4.796672	4.029260	-0.880729
H	-4.121140	6.090041	0.327831
H	-1.887535	6.206524	1.406371
H	-0.352687	4.285651	1.286291
H	-6.223749	-4.573800	-0.003922
H	-4.766247	-4.961728	-0.936339
H	-4.714327	-4.979392	0.833552
H	-5.121480	-2.489336	0.867878
H	-5.173448	-2.472473	-0.897742
H	4.081433	-4.519052	1.940927
H	4.069774	-2.813983	2.423514
H	2.607699	-3.804125	2.612696
H	2.412896	-4.020984	0.119633
H	3.879969	-3.040568	-0.087188

SCF Done: E(RB3LYP) = -1376.71980130 A.U. after 16 cycles
Sum of electronic and zero-point Energies= -1376.285014
Sum of electronic and thermal Energies= -1376.256580
Sum of electronic and thermal Enthalpies= -1376.255636
Sum of electronic and thermal Free Energies= -1376.347370

12.2.2 Ethyl (*Z*)-2-(1-cyano-2-ethoxy-2-oxoethylidene)-6-(4-methoxyphenyl)-4-phenyl-1,2-dihydropyridine-3-carboxylate (8b) (B3LYP)

C	0	-1.385269	-0.191069	-0.245424
C	0	-0.883398	-1.491473	-0.191814
C	0	0.506304	-1.706934	-0.093640
C	0	1.373007	-0.635337	-0.077350
N	0	0.840242	0.614190	-0.112084

C	0	-0.494996	0.918602	-0.164470
C	0	-0.850151	2.302767	-0.096741
C	0	0.159092	3.343981	-0.075924
O	0	1.382707	3.153737	-0.110021
C	0	-1.761500	-2.694286	-0.264765
C	0	2.839403	-0.758150	-0.043638
C	0	-1.699182	-3.652549	0.756860
C	0	-2.488487	-4.798169	0.699811
C	0	-3.336047	-5.011546	-0.387466
C	0	-3.391923	-4.071234	-1.414958
C	0	-2.613065	-2.916539	-1.354741
C	0	3.670943	0.193175	-0.663715
C	0	5.047717	0.059917	-0.637109
C	0	5.641024	-1.029114	0.018526
C	0	4.828853	-1.982161	0.644826
C	0	3.445597	-1.840381	0.606429
C	0	-2.863391	0.017223	-0.450113
O	0	-3.346535	0.320728	-1.517852
C	0	-2.191196	2.716547	0.046231
N	0	-3.282957	3.084354	0.191745
O	0	-0.355379	4.583859	-0.012598
C	0	-0.232636	6.969737	0.094467
C	0	0.580592	5.694288	0.024777
O	0	6.995858	-1.071660	-0.007057
C	0	7.666214	-2.158673	0.639504
O	0	-3.555461	-0.210860	0.665038
C	0	-5.570109	-0.345222	1.961181
C	0	-5.005812	-0.053446	0.587986
H	0	0.893808	-2.714524	-0.108191
H	0	1.446277	1.448767	-0.058807
H	0	-1.044469	-3.490638	1.605731
H	0	-2.440507	-5.523885	1.503635
H	0	-3.946092	-5.906344	-0.434567
H	0	-4.038622	-4.235825	-2.269181
H	0	-2.654604	-2.198903	-2.164617
H	0	3.243375	1.033230	-1.198501
H	0	5.684665	0.786624	-1.126331
H	0	5.258026	-2.825101	1.168188
H	0	2.835816	-2.576919	1.115582
H	0	0.443511	7.827901	0.125903
H	0	-0.877321	7.072776	-0.781108
H	0	-0.855319	6.990291	0.991552
H	0	1.227408	5.575221	0.896184
H	0	1.202225	5.656397	-0.871801
H	0	8.728253	-1.982527	0.484261
H	0	7.450353	-2.171149	1.711280
H	0	7.384658	-3.115703	0.191944
H	0	-6.657118	-0.235287	1.933415
H	0	-5.337084	-1.364914	2.275582
H	0	-5.175185	0.351561	2.703596
H	0	-5.210792	0.967898	0.264593
H	0	-5.381291	-0.744923	-0.168442

SCF Done: E(RB3LYP) = -1491.28008650   A.U. after   16 cycles
Sum of electronic and zero-point Energies=   -1490.810465
Sum of electronic and thermal Energies=   -1490.779350
Sum of electronic and thermal Enthalpies=   -1490.778405
Sum of electronic and thermal Free Energies=   -1490.876360

12.3 Calculated equilibrium ground-state structures of 1*H*-pyridines 5a and 8a

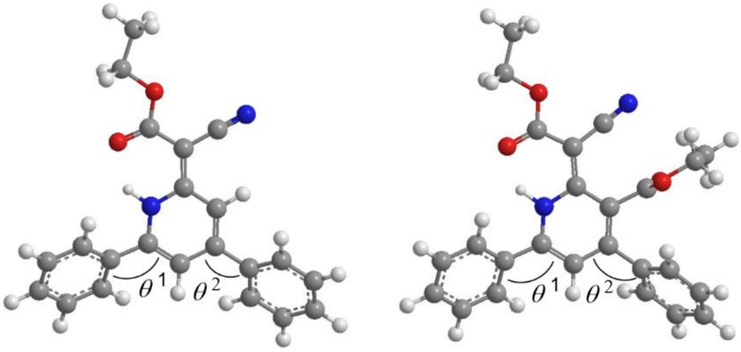

Figure 2: Optimized ground-state geometry at the B3LYP 6-311G** level of DFT theory for 1H-pyridine **5a** (left) and **8a** (right).

The calculated equilibrium ground-state structures of 1*H*-pyridines show that with the additional ester substituent in 3-position (**8a**) the aryl substituent in 4-position is sterically hindered and the torsional angle increases to 59° in comparison to 36° in the 1*H*-pyridine **5a**.

Table S9. Calculated equilibrium ground-state and solid-state dihedral angles for aryl substituents in 4- and 6-position of 1*H*-pyridine **5a** and **8a**.

Compound	R^1	R^2	R^3	θ^1	θ^2
**5a**	H	H	H	38°	36°
**5a** - exp., solid state	H	H	H	27	38
**8a**	H	H	COOEt	32°	59°

12.4 Computed xyz-coordinates of α-pyrones 6

Table S10. TDDFT Calculations (B3LYP/6-311G**) of the absorption maxima for α-pyrones **6**.

Compound	R^1	R^2	$\lambda_{max,abs}$ exp. [nm][a]	$\lambda_{max,abs}$ calcd. [nm]	Oscillator strength	most dominant contributions
**6a**	H	H	381	377	0.645	HOMO → LUMO (98 %)
			311	333	0.250	HOMO-1 → LUMO (98 %)
			258	262	0.256	HOMO → LUMO+1 (77 %)<br>HOMO-4 → LUMO (15 %)
**6b**	OMe	H	404	408	0.849	HOMO → LUMO (98 %)
			312	330	0.203	HOMO-1 → LUMO (95 %)<br>HOMO-3 → LUMO (2 %)
			271	278	0.161	HOMO → LUMO+1 (87 %)<br>HOMO-4 → LUMO (5 %)<br>HOMO-3 → LUMO (3 %)
			254	254	0.089	HOMO → LUMO+2 (82 %)<br>HOMO-4 → LUMO+1 (6 %)<br>HOMO-4 → LUMO (5 %)<br>HOMO → LUMO+3 (3 %)
**6c**	NMe_2	H	483	466	1.003	HOMO → LUMO (98 %)
			294	314	0.296	HOMO → LUMO+1 (56 %)<br>HOMO-2 → LUMO (35 %)<br>HOMO-4 → LUMO (5 %)
**6d**	H	OMe	358	394	0.329	HOMO → LUMO (82 %)<br>HOMO-1 → LUMO (17 %)
				376	0.665	HOMO-1 → LUMO (82 %)<br>HOMO → LUMO (17 %)
			258	270	0.321	HOMO → LUMO+1 (95 %)

**6e**	H	NMe_2	453	481	0.484	HOMO → LUMO (98 %)
			375	371	0.614	HOMO-1 → LUMO (98 %)
			289	317	0.180	HOMO → LUMO+1 (63 %)
						HOMO-2 → LUMO (34 %)
				314	0.109	HOMO-2 → LUMO (63 %)
						HOMO → LUMO+1 (34 %)
						HOMO → LUMO+3 (2 %)
			254	259	0.167	HOMO-1 → LUMO+1 (72 %)
						HOMO-4 → LUMO (15 %)
						HOMO → LUMO+2 (2 %)
						HOMO → LUMO+5 (2 %)
**6f**	OMe	OMe	400	407	0.941	HOMO → LUMO (92 %)
						HOMO-1 → LUMO (8 %)
			364	383	0.216	HOMO-1 → LUMO (98 %)
						HOMO → LUMO (8 %)
			254	276	0.176	HOMO → LUMO+1 (85 %)
						HOMO-4 → LUMO (5 %)
						HOMO-1 → LUMO+1 (5 %)
						HOMO-2 → LUMO (3 %)
				264	0.184	HOMO-1 → LUMO+1 (92 %)
						HOMO → LUMO+1 (2 %)
**6g**	CF_3	NMe_2	465	503	0.449	HOMO → LUMO (100 %)
			372	369	0.651	HOMO-1 → LUMO (98 %)
			309	332	0.289	HOMO → LUMO+1 (95 %)
			251	261	0.139	HOMO-1 → LUMO+1 (56 %)
						HOMO-4 → LUMO (25 %)
						HOMO-6 → LUMO (6 %)
						HOMO-5 → LUMO (3 %)
						HOMO → LUMO+6 (3 %)

[a] Recorded in dichloromethane, T = 293 K, c_0(**6**) = 10^{-6} M.

12.4.1 2-Oxo-4,6-diphenyl-2*H*-pyran-3-carbonitrile (6a) (B3LYP)

C 0 1.049842 0.163782 -0.017468
C 0 1.073179 1.551260 -0.104137
C 0 -0.155560 2.321735 -0.124302
O 0 -1.332382 1.575082 -0.077612
C 0 -1.371355 0.224783 -0.012659
C 0 -0.208880 -0.491163 0.022729
O 0 -0.268665 3.522631 -0.185516
C 0 -2.733822 -0.316654 0.033877
C 0 2.279904 -0.658588 0.038123
C 0 2.388440 -1.802198 -0.768826
C 0 3.534346 -2.590056 -0.722438
C 0 4.575121 -2.261428 0.146344
C 0 4.468158 -1.136847 0.963834
C 0 3.332387 -0.334136 0.906294
C 0 -3.827530 0.538525 0.252232
C 0 -5.119592 0.026802 0.304789
C 0 -5.342072 -1.339377 0.134848
C 0 -4.262634 -2.195562 -0.090641
C 0 -2.969097 -1.691735 -0.141520
C 0 2.267861 2.309704 -0.239647
N 0 3.219058 2.956094 -0.365616
H 0 -0.242819 -1.564028 0.125747
H 0 1.587328 -2.060029 -1.451716
H 0 3.614057 -3.459942 -1.363805
H 0 5.463830 -2.880478 0.187892
H 0 5.268327 -0.883664 1.649308
H 0 3.254058 0.528315 1.556260
H 0 -3.660093 1.598593 0.386109
H 0 -5.953653 0.696217 0.479065
H 0 -6.350100 -1.735528 0.174053
H 0 -4.429993 -3.256591 -0.232693
H 0 -2.148145 -2.370295 -0.334497
SCF Done: E(RB3LYP) = -897.973056447 A.U. after 16 cycles
Sum of electronic and zero-point Energies= -897.718076
Sum of electronic and thermal Energies= -897.702463
Sum of electronic and thermal Enthalpies= -897.701518
Sum of electronic and thermal Free Energies= -897.762078

12.4.2 6-(4-Methoxyphenyl)-2-oxo-4-phenyl-2*H*-pyran-3-carbonitrile (6b) (B3LYP)

C 0 1.786866 0.135635 -0.023787
C 0 2.036906 1.505645 -0.069462
C 0 0.949314 2.461038 -0.079508
O 0 -0.332890 1.911206 -0.051542
C 0 -0.593810 0.583042 -0.024800
C 0 0.444844 -0.311180 -0.012680
O 0 1.026604 3.666816 -0.115261
C 0 -2.018260 0.272696 0.003481
C 0 2.872146 -0.872398 0.017459
C 0 2.821941 -1.988135 -0.832482
C 0 3.829505 -2.947480 -0.795004

C	0	4.886699	-2.817907	0.105885
C	0	4.935479	-1.720808	0.965230
C	0	3.939821	-0.749013	0.917987
C	0	-2.977963	1.305851	0.049973
C	0	-4.328599	1.020030	0.079507
C	0	-4.772135	-0.312159	0.062657
C	0	-3.832719	-1.352335	0.013940
C	0	-2.477779	-1.054551	-0.015144
C	0	3.339470	2.062734	-0.173607
N	0	4.385491	2.548213	-0.271948
O	0	-6.111032	-0.491203	0.095292
C	0	-6.638353	-1.823804	0.085657
H	0	0.243443	-1.368949	0.043484
H	0	2.007068	-2.091134	-1.539602
H	0	3.788842	-3.795256	-1.468920
H	0	5.667065	-3.569387	0.139249
H	0	5.748224	-1.620567	1.675005
H	0	3.980009	0.091382	1.599757
H	0	-2.653328	2.337255	0.065071
H	0	-5.063811	1.814452	0.116934
H	0	-4.145661	-2.386761	-0.002659
H	0	-1.778710	-1.879779	-0.056563
H	0	-7.719334	-1.710173	0.119389
H	0	-6.353190	-2.350579	-0.828574
H	0	-6.300752	-2.383946	0.961342

SCF Done: E(RB3LYP) = -1012.53396741 A.U. after 17 cycles
Sum of electronic and zero-point Energies= -1012.244367
Sum of electronic and thermal Energies= -1012.225194
Sum of electronic and thermal Enthalpies= -1012.224249
Sum of electronic and thermal Free Energies= -1012.294106

12.4.3 6-[4-(Dimethylamino)phenyl]-2-oxo-4-phenyl-2*H*-pyran-3-carbonitrile (6c) (B3LYP)

C	0	2.134009	0.140613	-0.027007
C	0	2.374584	1.517798	-0.068907
C	0	1.279345	2.459309	-0.081382
O	0	0.001435	1.898226	-0.057217
C	0	-0.252162	0.565722	-0.034642
C	0	0.803809	-0.319463	-0.021616
O	0	1.341603	3.667951	-0.115774
C	0	-1.660318	0.240097	-0.011669
C	0	3.232470	-0.854831	0.017777
C	0	3.208967	-1.962808	-0.842958
C	0	4.226093	-2.912143	-0.797917
C	0	5.267976	-2.779378	0.120086
C	0	5.291537	-1.688804	0.988795
C	0	4.285493	-0.727857	0.934709
C	0	-2.643179	1.252715	0.020606
C	0	-3.989681	0.956589	0.046761
C	0	-4.446719	-0.389390	0.041010
C	0	-3.454743	-1.408937	0.004162
C	0	-2.112978	-1.097130	-0.020402
C	0	3.671849	2.083842	-0.167738

N 0 4.716282 2.575637 -0.260414
N 0 -5.774410 -0.691287 0.069894
C 0 -6.218080 -2.082759 0.059424
C 0 -6.774950 0.371791 0.114300
H 0 0.612793 -1.379364 0.031652
H 0 2.405688 -2.067913 -1.562982
H 0 4.204767 -3.754527 -1.479586
H 0 6.056135 -3.522489 0.158874
H 0 6.092793 -1.585180 1.711169
H 0 4.306611 0.108105 1.622877
H 0 -2.336174 2.290103 0.027232
H 0 -4.698189 1.771970 0.072566
H 0 -3.743740 -2.449982 -0.004771
H 0 -1.404893 -1.915763 -0.049970
H 0 -7.304800 -2.107293 0.080727
H 0 -5.882849 -2.603254 -0.843472
H 0 -5.846982 -2.628531 0.933009
H 0 -7.765036 -0.076146 0.147374
H 0 -6.653905 0.997751 1.004122
H 0 -6.719557 1.012797 -0.771600
SCF Done: E(RB3LYP) = -1031.98593175 A.U. after 17 cycles
Sum of electronic and zero-point Energies= -1031.654937
Sum of electronic and thermal Energies= -1031.634591
Sum of electronic and thermal Enthalpies= -1031.633647
Sum of electronic and thermal Free Energies= -1031.706192

12.4.4 4-(4-Methoxyphenyl)-2-oxo-6-phenyl-2*H*-pyran-3-carbonitrile (6d) (B3LYP)

C 0 0.319483 0.528880 -0.080222
C 0 0.082972 1.902364 -0.107717
C 0 -1.263837 2.434846 -0.062809
O 0 -2.285517 1.487522 -0.015743
C 0 -2.076199 0.150420 -0.011278
C 0 -0.804189 -0.341885 -0.042636
O 0 -1.595230 3.597316 -0.072244
C 0 -3.317400 -0.630813 0.053754
C 0 1.670002 -0.059247 -0.082015
C 0 1.940736 -1.208853 -0.850778
C 0 3.199723 -1.779270 -0.862366
C 0 4.229754 -1.232751 -0.080399
C 0 3.973262 -0.104649 0.708089
C 0 2.708278 0.473649 0.693309
C 0 -4.546580 0.013274 0.276229
C 0 -5.724459 -0.723192 0.345944
C 0 -5.697560 -2.108673 0.191419
C 0 -4.482265 -2.757134 -0.035945
C 0 -3.301965 -2.027933 -0.105176
C 0 1.108379 2.875340 -0.253018
N 0 1.915379 3.693959 -0.387109
O 0 5.425726 -1.864467 -0.150805
C 0 6.526524 -1.350892 0.607980
H 0 -0.642995 -1.406379 0.011839
H 0 1.164249 -1.639919 -1.471545

H 0	3.411218	-2.648666	-1.472817
H 0	4.741258	0.325928	1.335314
H 0	2.528569	1.334044	1.325025
H 0	-4.573685	1.087437	0.398420
H 0	-6.664362	-0.213415	0.521848
H 0	-6.616644	-2.680546	0.245069
H 0	-4.454905	-3.832826	-0.163183
H 0	-2.373865	-2.551780	-0.294078
H 0	7.366128	-2.005677	0.386106
H 0	6.767841	-0.328834	0.304458
H 0	6.311746	-1.380928	1.679443

SCF Done: E(RB3LYP) = -1012.53327506 A.U. after 17 cycles
Sum of electronic and zero-point Energies= -1012.244248
Sum of electronic and thermal Energies= -1012.226013
Sum of electronic and thermal Enthalpies= -1012.225069
Sum of electronic and thermal Free Energies= -1012.291591

12.4.5 4-[4-(Dimethylamino)phenyl]-2-oxo-6-phenyl-2*H*-pyran-3-carbonitrile (6e) (B3LYP)

C 0	-0.015671	0.684902	-0.073640
C 0	-0.375047	2.036420	-0.150046
C 0	-1.759897	2.454909	-0.134031
O 0	-2.705679	1.432071	-0.071408
C 0	-2.380406	0.117154	-0.027748
C 0	-1.075293	-0.269664	-0.026136
O 0	-2.186212	3.587135	-0.182013
C 0	-3.551482	-0.766389	0.050300
C 0	1.364795	0.207483	-0.025985
C 0	1.724401	-1.012490	-0.636154
C 0	3.019233	-1.490746	-0.609621
C 0	4.047780	-0.782194	0.067076
C 0	3.679956	0.430151	0.708838
C 0	2.385715	0.907499	0.647484
C 0	-4.810308	-0.243893	0.390829
C 0	-5.918126	-1.080716	0.481289
C 0	-5.790637	-2.446004	0.228070
C 0	-4.545901	-2.972416	-0.121838
C 0	-3.435079	-2.142366	-0.211833
C 0	0.556503	3.092062	-0.334150
N 0	1.284134	3.976417	-0.505697
N 0	5.329911	-1.248058	0.103670
C 0	6.367840	-0.501090	0.807432
C 0	5.675315	-2.513227	-0.536467
H 0	-0.838558	-1.316028	0.079920
H 0	0.980991	-1.582972	-1.180621
H 0	3.240107	-2.416307	-1.121768
H 0	4.412716	0.995543	1.266569
H 0	2.156988	1.825722	1.172440
H 0	-4.914090	0.814016	0.591272
H 0	-6.881913	-0.665172	0.751162
H 0	-6.655290	-3.095831	0.297438
H 0	-4.442237	-4.030405	-0.332039
H 0	-2.483047	-2.566701	-0.504889

H 0 7.317396 -1.017250 0.687801
H 0 6.476919 0.509823 0.402170
H 0 6.154121 -0.423332 1.879162
H 0 6.735003 -2.706760 -0.388167
H 0 5.112875 -3.349148 -0.106837
H 0 5.482676 -2.483674 -1.614071
SCF Done: E(RB3LYP) = -1031.98382243 A.U. after 15 cycles
Sum of electronic and zero-point Energies= -1031.653267
Sum of electronic and thermal Energies= -1031.632201
Sum of electronic and thermal Enthalpies= -1031.631257
Sum of electronic and thermal Free Energies= -1031.705513

12.4.6 4,6-Bis(4-methoxyphenyl)-2-oxo-2*H*-pyran-3-carbonitrile (6f) (B3LYP)

C 0 -1.092381 0.682780 0.075482
C 0 -1.098149 2.079387 0.072610
C 0 0.136160 2.832536 0.023500
O 0 1.306042 2.073102 -0.005368
C 0 1.333276 0.719394 0.018024
C 0 0.159099 0.017305 0.060226
O 0 0.266468 4.035103 0.013234
C 0 2.684077 0.168118 -0.021196
C 0 -2.324756 -0.126811 0.090599
C 0 -2.405695 -1.278813 0.897457
C 0 -3.548075 -2.057427 0.917597
C 0 -4.644590 -1.723364 0.107485
C 0 -4.575143 -0.593489 -0.715583
C 0 -3.428275 0.194306 -0.710039
C 0 3.805295 1.021287 -0.090582
C 0 5.087501 0.509078 -0.125517
C 0 5.298058 -0.878522 -0.092477
C 0 4.195532 -1.742272 -0.023178
C 0 2.911232 -1.217429 0.011652
C 0 -2.276927 2.862156 0.191974
N 0 -3.215075 3.531346 0.302792
O 0 -5.714856 -2.551048 0.186858
C 0 -6.872223 -2.263801 -0.605986
O 0 6.586981 -1.284481 -0.131126
C 0 6.877565 -2.687294 -0.103217
H 0 0.181748 -1.060354 0.040314
H 0 -1.575708 -1.549046 1.539602
H 0 -3.615829 -2.929853 1.556053
H 0 -5.397361 -0.321309 -1.362515
H 0 -3.390347 1.052997 -1.368054
H 0 3.661358 2.092740 -0.117186
H 0 5.946752 1.166323 -0.179126
H 0 4.327689 -2.814695 0.004091
H 0 2.081939 -1.910925 0.066467
H 0 -7.589965 -3.046562 -0.371131
H 0 -7.293983 -1.289535 -0.345421
H 0 -6.634203 -2.293468 -1.672661
H 0 7.961806 -2.761831 -0.141812
H 0 6.511063 -3.144421 0.819500

H 0 6.444003 -3.193662 -0.969435
SCF Done: E(RB3LYP) = -1127.09400340 A.U. after 16 cycles
Sum of electronic and zero-point Energies= -1126.770457
Sum of electronic and thermal Energies= -1126.748653
Sum of electronic and thermal Enthalpies= -1126.747709
Sum of electronic and thermal Free Energies= -1126.823984

12.4.7 4-[4-(Dimethylamino)phenyl]-2-oxo-6-[4-(trifluormethyl)pheny]-2*H*-pyran-3-carbonitrile (6g) (B3LYP)

C 0 2.524434 -0.987287 -0.334900
C 0 3.796170 -1.540205 -0.355474
C 0 4.907775 -0.739798 -0.081384
C 0 4.742081 0.612752 0.213289
C 0 3.467305 1.166207 0.229884
C 0 -3.826404 0.605941 -0.660198
C 0 -4.972675 -0.162290 -0.691393
C 0 -5.043540 -1.406653 -0.009576
C 0 -3.874679 -1.831910 0.677421
C 0 -2.728321 -1.064079 0.673969
C 0 2.341106 0.372731 -0.036291
C 0 -2.665907 0.186273 0.022113
O 0 0.008771 4.299314 0.054825
C 0 -0.143688 3.099398 0.034007
O 0 1.013053 2.321053 -0.012425
C 0 0.995089 0.966724 -0.002109
C 0 -0.181383 0.289128 0.036706
C 0 -1.436982 0.973855 0.040016
C 0 -1.396271 2.372817 0.058346
C 0 -2.545700 3.195244 0.197982
N 0 -3.456046 3.897885 0.332123
C 0 6.278195 -1.357476 -0.061259
F 0 6.411837 -2.340099 -0.982664
F 0 6.560421 -1.925209 1.143217
F 0 7.257986 -0.457556 -0.297740
N 0 -6.178783 -2.161951 -0.019127
C 0 -6.215282 -3.450378 0.666190
C 0 -7.363024 -1.707878 -0.742472
H 0 1.679822 -1.619811 -0.573810
H 0 3.922461 -2.587561 -0.597965
H 0 5.601147 1.236915 0.422465
H 0 3.343722 2.216501 0.454479
H 0 -3.821961 1.534231 -1.216056
H 0 -5.820118 0.198265 -1.256626
H 0 -3.869823 -2.766550 1.219591
H 0 -1.871803 -1.427608 1.229303
H 0 -0.168037 -0.788601 0.028212
H 0 -7.205227 -3.884791 0.549937
H 0 -5.483762 -4.150174 0.248019
H 0 -6.016585 -3.339945 1.737088
H 0 -8.159042 -2.436834 -0.610688
H 0 -7.717783 -0.744213 -0.363420

H 0 -7.165777 -1.607209 -1.815208
SCF Done: E(RB3LYP) = -1369.13226199 A.U. after 15 cycles
Sum of electronic and zero-point Energies= -1368.796655
Sum of electronic and thermal Energies= -1368.772697
Sum of electronic and thermal Enthalpies= -1368.771753
Sum of electronic and thermal Free Energies= -1368.853615

12.5 Calculated equilibrium ground-state structures of α-pyrones 6

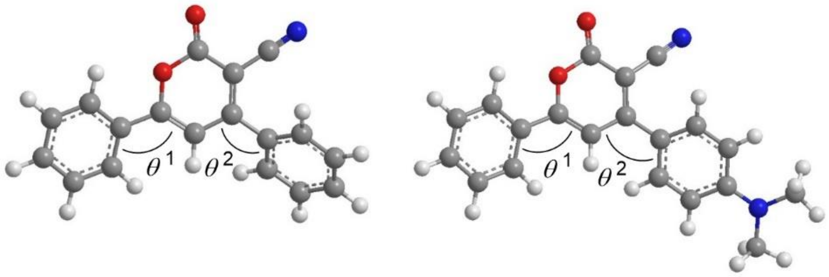

Figure 3: Optimized ground-state geometry at the B3LYP 6-311G** level of DFT theory for α-pyrone **6a** (left) and **6e** (right).

The calculated equilibrium ground-state structures of α-pyrones **6** show that for (dimethylamino)phenyl-substituted compound **6c** the aryl substituent in 6-position is almost planar with an angle of 2° (Table S11). The other (dimethylamino)phenyl derivate **6e** shows a smaller torsional angle for the substituted aryl substituent in 4-position. With the introduction of an additional electron-accepting group (**6g**) the angles are not influenced.

Table S11. Calculated equilibrium ground state torsional angles of α-pyrones **6.**

Compound	R^1	R^2	θ^1	θ^2
**6a**	H	H	13°	46°
**6c**	NMe_2	H	2°	48°
**6e**	H	NMe_2	17°	35°
**6g**	CF_3	NMe_2	15°	33°

13 References

[1] Rurack, K.; Spieles, M. *Anal. Chem.* **2011**, *83*, 1232-1242. doi.org/10.1021/ac101329h

[2] a) D'Souza, D. M.; Müller, T. J. J. *Nat. Protoc.* **2008**, *3*, 1660. DOI: 10.1038/nprot.2008.152; b) Karpov, A. S.; Müller, T. J. J. *Org. Lett.* **2003**, *5*, 3451-3454. DOI: 10.1021/ol035212q

[3] Wu, X. F.; Neumann, H.; Beller, M. *Chem. Eur. J.* **2010**, *16*, 12104-12107. DOI: 10.1002/chem.201001864

[4] Levashov, A. S.; Aksenov, N. A.; Aksenova, I. V.; Konshin, V. V. *New J. Chem.* **2017**, *41*, 8297-8304. DOI: 10.1039/C7NJ01376K

[5] Ogiwara, Y.; Kubota, M.; Kurogi, K.; Konakahara, T.; Sakai, N. *Chem. Eur. J.* **2015**, *21*, 18598-18600. DOI: 10.1002/chem.201504255

[6] Zhang, C.; Liu, J.; Xia, C. *Org. Biomol. Chem.* **2014**, *12*, 9702-9706. DOI: 10.1039/C4OB01878H

[7] Götzinger, A. C.; Müller, T. J. J. *Org. Biomol. Chem.* **2016**, *14*, 3498-3500. DOI: 10.1039/C6OB00483K

[8] Atobe, S.; Masuno, H.; Sonoda, M.; Suzuki, Y.; Shinohara, H.; Shibata, S.; Ogawa, A. *Tetrahedron Lett.* **2012**, *53*, 1764-1767. DOI: 10.1016/j.tetlet.2012.01.105

[9] Kim, W.; Park, K.; Park, A.; Choe, J.; Lee, S. *Org. Lett.* **2013**, *15*, 1654-1657. DOI: 10.1021/ol4004349

[10] Liu, J.; Zhang, X.; Peng, H.; Jiang, H.; Yin, B. *Adv. Synth. Catal.* **2015**, *357*, 727-731. DOI: 10.1002/adsc.201401014

[11] Breuer, N.; Müller, T. J. J. *Synthesis* **2018**, *50*, 2741–2752. DOI: 10.1055/s-0037-1610129

[12] Karlsen, H.; Songe, P. H.; Sunsby, L. K.; Hagen, L. C.; Kolsaker, P.; Rømming, C. *J. Chem. Soc. Perkin Trans. 1* **2001,** 497–507. DOI: 10.1039/B009175H

[13] APEX2, data collection program for the CCD area-detector system; SAINT, data reduction and frame integration program for the CCD area-detector system. Bruker analytical X-ray.

[14] APEX2. SAINT, Data Reduction and Frame Integration Program for the CCD Area-Detector System, Bruker Analytical X-ray Systems; Data Collection program for the CCD Area-Detector System: Madison, WI, USA,1997–2006.

[15] Sheldrick, G. Program SADABS: Area-detector absorption correction, University of Göttingen, Germany, **1996**.

[16] a) Sheldrick, G. *Acta Crystallogr. A* **2008**, *64*, 112-122. doi.org/10.1107/S0108767307043930 b) Hübschle, C. B; Sheldrick, G. M.; Dittrich, B. ShelXle: A graphical user interface for SHELXL. *J. Appl. Cryst.* **2011**, *44*, 1281-1284. doi.org/10.1107/S0021889811043202

[17] Brandenburg, K. *DIAMOND; version 4; Crystal and Molecular Structure Visualization; Crystal Impact—K.* Brandenburg & H. Putz Gbr: Bonn, Germany, **2009**.

[18] a) Spek, A. L. *PLATON-A Multipurpose Crystallographic Tool.* Utrecht, The Netherlands **2005**. b) Spek, A. L. Structure validation in chemical crystallography. *Acta Crystall. Section D: Biol. Crystal*. **2009**, *65*, 148-155. DOI: 10.1107/S090744490804362X
